# Understanding and Challenges of Community Nursing Practicums After COVID‐19: A Qualitative Study

**DOI:** 10.1155/nrp/6657747

**Published:** 2026-02-12

**Authors:** Sumi Lee

**Affiliations:** ^1^ Department of Nursing Science, Howon University, Gunsan-Si, Republic of Korea, howon.ac.kr

**Keywords:** community, COVID-19, nursing, nursing students, practicums, public health, qualitative research

## Abstract

**Background:**

The COVID‐19 pandemic highlighted the critical role of community health services, yet little is known about how nursing students perceive and engage with public health centers during their community nursing practicums in this evolving landscape.

**Purpose:**

To explore the community nursing practicum experiences of Korean nursing students at public health center after the COVID‐19 pandemic.

**Methods:**

This qualitative study was conducted in the Republic of Korea. It employed purposive sampling and thematic analysis to examine the practicum experiences of 29 nursing students. The data were collected using daily reflective journals written by students during their practicum, with research consent obtained after all grades were finalized.

**Results:**

Three themes were identified: (a) Understanding and engagement with public health centers, including limited prior awareness of community health services and expanded recognition of diverse public health initiatives. (b) Practicing and overcoming challenges in community nursing involves understanding the role of community nurses in preventive care, experiencing visiting nursing services, enhancing knowledge and skills, and clarifying career options. (c) Leading health promotion projects as a nursing professional entails roles as community health assessors, specialized practitioners, and collaborating with other experts.

**Conclusions:**

This study highlights how community nursing practicum experiences after the COVID‐19 pandemic contributed to enhancing students’ understanding of public health roles and practical skills, influencing some students’ career choices. To maximize outcomes, programs should implement prepracticum preparation and strengthen site collaboration for active student engagement in assessments. Through well‐designed practicum experiences, nurses might be better prepared to address future community health challenges as health assessors and collaborators within multidisciplinary teams.

## 1. Introduction

The COVID‐19 pandemic has had a profound impact on global healthcare systems, resulting in a heightened emphasis on managing infectious diseases. The pandemic led to significant shifts to in‐person nursing education, especially in community health nursing practicums. As a result, nursing students experienced practical training that was replaced by distance learning in digital environments or simulations conducted in school nursing laboratories [1], limiting traditional in‐person practicums. As in‐person community nursing practicums resumed postpandemic, it became imperative to understand the experiences of nursing students who had limited prior in‐person exposure and whose perception of public health centers (PHCs) was predominantly restricted to COVID‐19 infectious disease management. By exploring and understanding the experience of community nursing practicum that resumed after postpandemic, we can improve the quality of community nursing education and contribute to the development of competent community health nursing professionals.

The PHCs in Korea are governmental public health institutions established nationwide under the Local Public Health Law, serving as a core public health infrastructure [2]. Pivotal in infectious disease management, they provide comprehensive services including infection prevention and control, maternal and child care, healthcare for vulnerable populations, health promotion, and environmental hygiene management [3]. By offering these services, PHCs act as the cornerstone of community healthcare and play a vital role in the primary healthcare sector, ensuring health equity [4]. Across Korea, 2,682 nurses work in PHCs, representing 49.7% of the public health workforce [5]. Practicums, which were conducted prior to the pandemic, were completely suspended during the peak of the pandemic but have since resumed. With the resumption of practicums, most fourth‐year nursing students in Korean universities currently undertake their community nursing practicums at PHCs. This ongoing practice offers unique opportunities to bridge the gap between theory and practice, engage with healthcare services, and experience community nursing [6, 7].

Before the pandemic, experienced nurses who worked collaboratively with professionals from various fields and had a high level of autonomy were found to have a positive community nursing learning experience [7]. During the COVID‐19, efforts were made to enhance self‐reflection and critical thinking skills among nursing students through experiential learning programs such as those involving older adults, pregnancy, and infant care [8]. There was a strong emphasis on verifying the effectiveness of alternative nursing education methods. Meanwhile, community nursing practice aimed at strengthening professionalism and exploring nursing students’ perceptions of community health [6].

The importance of reflective journals in nursing education is well documented [9]. These journals, designed to engage clinical reasoning skills, deep reflection, and professional self‐development, help students analyze their learning experiences and promote ongoing education [9]. As a self‐evaluative educational tool, the reflective journal helps students clarify their thoughts, analyze behaviors, and develop self‐awareness, which are crucial for their learning and growth [10, 11]. Analyzing these journals provides valuable data, helping to identify evolving needs for nurse educators, practitioners, and policymakers. Reflective journals provide a rich source of data for nurse educators to evaluate student learning outcomes in community nursing practice, offering insights into students’ evolving self‐awareness for PHC, experience in the role of community nurse, and development of professional nursing skills. Nursing students’ reflective journals reveal that nursing practice fosters self‐reflection, reducing anxiety and enhancing proficiency, while the act of writing these reflections particularly enhances their sense of achievement and strengthens their nursing identity [12]. Community nursing practice influences students’ perceptions of community nursing, enhances their understanding of the community nurse’s role, and offers valuable professional experiences [6, 9]. Finally, the effect of nursing practices during the COVID‐19 pandemic and the use of online, noncontact methods have been documented [8].

Despite extensive research on nursing education in the United States and Europe, there are limited studies in Asia, particularly on community nursing. Although practical experiences in basic, adult, and management nursing have been reported, community nursing research remains limited. The pandemic has significantly altered the healthcare delivery and nursing education, underscoring the need to understand community nursing practice.

This study aims to explore the meaning of community nursing practice experiences at PHCs for nursing students by analyzing their self‐reflective journals in the postpandemic era. By examining these experiences from the students’ perspectives, we can gain valuable insights into how they perceive community nursing. These insights will not only support the development of community nursing professionals but also contribute to cultivating future community nurses equipped with practical knowledge and skills.

## 2. Methods

### 2.1. Study Design and Sample

This qualitative study employed purposive sampling and thematic analysis to examine the practicum experiences of Korean nursing students in PHC during the post‐COVID‐19 period. Thematic analysis was chosen due to its suitability in identifying and analyzing patterns through reflective journals, making it ideal for capturing the diversity of student experiences during their placements in a public health setting.

Participants were selected using purposive sampling based on specific criteria. Students were eligible if they: (1) were enrolled as fourth‐year nursing students, (2) had completed the 2‐week community nursing practicum at a PHC, and (3) voluntarily agreed to participate in the study. Students who withdrew consent at any point during the study period were to be excluded from the analysis; however, no participants withdrew their consent during the study.

Senior nursing students at Howon University in Gunsan, Republic of Korea, complete a 2‐week practicum at a PHC. This is their first hands‐on experience following community nursing theoretical coursework, during which they explore departmental functions and engage in various projects. Gunsan City Public Health Center operates under the leadership of its director and comprises departments responsible for health administration, infectious disease management, health promotion, clinical services, and home‐based care. The center serves as a primary institution delivering comprehensive and specialized public health services aimed at promoting community health and preventing disease among local residents.

### 2.2. Data Collection

Data were collected from students’ daily reflective journals written during their practicum. Reflective journaling allows students to critically examine their learning experiences, new knowledge acquisition, challenges, and meaning‐making. Consent was obtained from study participants from June 9 to September 7, 2024, after their reflective journals were completed and their grades were finalized. Of 60 students who completed the practicum, 29 (6 men, 23 women; mean age 27.6 years, range 23–58 years) agreed to participate and provided their journals for analysis. These documents offered in‐depth narratives of students’ thoughts and insights, providing valuable understanding of how they navigated community practice challenges in the postpandemic period.

The reflective journals were collected daily during the 2‐week practicum period. Each journal consists of five questions that prompt students to self‐evaluate their learning each day through community nursing practicum. These questions include: “What was meaningful about your practical experience?”, “How did you feel and think during practice?”, “What new things did you learn through practice?”, “How can what you learned be applied to your future nursing practice?”, and “What went well in practice, and what could be improved?”.

### 2.3. Rationale for Thematic Analysis

Thematic analysis was chosen for its flexibility and effectiveness in uncovering personal and subjective experiences from reflective journals [13]. This approach allows for a comprehensive examination of students’ learning processes, perceptions, and challenges, providing deep insights into their experiences during the community nursing practicum in the post‐COVID‐19. Unlike more rigid qualitative methods, thematic analysis enables both inductive and deductive coding, allowing the research team to integrate theoretical concepts while remaining open to unexpected findings [14]. This adaptability makes it particularly suitable for capturing the diverse experiences of students and identifying common patterns in their learning journey.

### 2.4. Thematic Analysis Process

This study employed thematic analysis on students’ reflective journals, following Braun and Clarke’s six‐step process [13]. (1) Familiarization with data—thoroughly reading journals, noting key insights and patterns. (2) Generating initial codes—using inductive and deductive approaches to categorize significant phrases and concepts. (3) Searching for themes—grouping codes into broader themes, with three main themes emerging. (4) Reviewing themes—iteratively refining themes for accurate data representation through peer debriefing with fellow nursing instructors to enhance analytical objectivity. Data saturation was reached after analyzing 22 journals, with the final seven confirming no new themes emerged. (5) Defining and naming themes—clearly labeling each theme, informed by the confirmed saturation point, and employing member checking with participants to verify that interpretations accurately reflected their experiences. (6) Producing the report—organizing themes into a cohesive narrative with extensive direct quotes from journals to provide an audit trail demonstrating the link between raw data and findings.

Data were manually managed and analyzed through a systematic thematic analysis process, without the use of qualitative analysis software. The researcher developed a thematic framework, indexed the dataset against it, and iteratively presented the analysis to fellow instructors for feedback. This process, including review by participants, was repeated to enhance the credibility and trustworthiness of the findings. The SRQR checklist for this study is presented in supporting table S1.

### 2.5. Translation and Cross‐Linguistic Validation

In cross‐language qualitative research, translation accuracy and cultural equivalence are crucial, particularly when translating Korean reflective journals into English. This study followed a rigorous process based on a guideline for qualitative translation [15]. First, a bilingual expert translated the journals, focusing on preserving meaning and cultural nuances. A second bilingual researcher conducted a back‐translation to identify discrepancies. Any inconsistencies were addressed through consensus meetings involving the translator and the researcher, ensuring both linguistic and conceptual alignment.

### 2.6. Ethical Statement

Ethical approval for this study was granted by the Institutional Review Board (IRB) of Howon University (No. 1041585‐202402‐HR‐003‐02). Students were informed about the study’s purpose, data usage, and their right to withdraw. Informed consent was obtained before data collection. This approach received IRB approval, deemed essential given that the reflective journals constituted an evaluative component of the course and an inherent relationship existed between the researcher and participants. This ensured all participation was voluntary and had no impact on their grades. All identifying information was anonymized using pseudonyms to protect participant confidentiality.

## 3. Results

**FIGURE 1:**
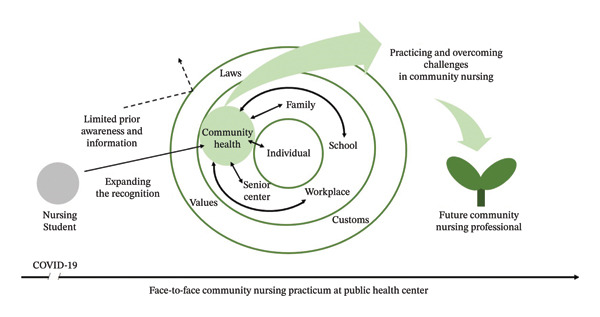
Illustration of results on nursing students’ experience in community after the pandemic.

This study showed a focus on understanding and engagement with PHC, practicing and overcoming challenges in community nursing, and leading health promotion projects as a nursing professional. This study identified three main themes and nine subthemes, which are presented in Table 1. The conceptual structure and thematic relationships in nursing students’ postpandemic community experiences are shown in Figure 1.

**TABLE 1:** Theme and subtheme analysis of nursing students’ community health nursing practicum experiences in public health center postpandemic.

Theme	Subtheme
1. Understanding and engagement with public health center	1.1. Limited prior awareness of community health services
	1.2. Expanded recognition of diverse public health initiatives

2. Practicing and overcoming challenges in community nursing	2.1. Understanding the role of community nurses in preventive care
	2.2. Experiencing visiting nursing services
	2.3. Enhancing knowledge and skills
	2.4. Clarifying career options

3. Leading health promotion projects as a nursing professional	3.1. Community health assessors
	3.2. Specialized practitioners
	3.3. Collaborate with other experts

### 3.1. Theme 1: Understanding and Engagement With PHC

#### 3.1.1. Subtheme 1.1. Limited Prior Awareness of Community Health Services

During the COVID‐19 pandemic, many students became familiar with PHC primarily through media coverage of infection control activities. However, most had little direct experience with these centers, aside from visits for COVID‐19 testing or childhood vaccinations. Students observed the extensive services offered including vaccination programs, maternal and child health services, *tuberculosis* management, and dementia care.

*I found that quite a few people visit to take advantage of the various programs and services. Maternal and child health, tuberculosis management, and the dementia care center*… (P2).

*Public health center aren’t somewhere I’ve gone often. Before my practicum, the only times I’d been were to get a COVID test and for vaccines back in elementary school. I didn’t really know what they did there, but I learned a lot during the practicum. I was surprised to see so many more people using the center than I’d expected.* (P12).

*I had never used a public health center before, so I was surprised to learn about the various specialized services inside.* (P21).

These observations highlighted the students’ limited prior awareness and knowledge regarding community health services.

#### 3.1.2. Subtheme 1.2. Expanded Recognition of Diverse Public Health Initiatives

Students initially knew PHCs mainly for infection control but discovered that these centers offer comprehensive services, such as health promotion, prenatal care, nutritional support, and chronic disease management, unlike hospitals’ treatment‐focused approach.

*I learned about the Red Circle Campaign through a program called ‘Learn about Myocardial Infarction’ at the visiting health management center. This campaign raises awareness among citizens about their vascular age and teaches them prevention strategies for cardiovascular diseases.* (P15).

*I learned about preventing dental caries, malocclusion, and periodontal disease at the Oral Health Center. Recognizing that individuals with disabilities and other vulnerable groups face greater challenges in maintaining oral health than the general public, I participated in a free treatment program to support their needs.* (P15).

*A 40-year-old man who had quit smoking for over six months visited the smoking cessation counseling room. To celebrate, he received a gift set with useful tools and expressed happiness about his achievement, reaffirming his commitment to a smoke-free. However, many other participants who had not yet quit felt excluded from receiving gifts.* (P21).

*The health promotion room launched a mobile healthcare initiative to prevent metabolic syndrome. This six-month program included health consultations, basic checkups, nutritional guidance, and personalized exercise plans. During the initial visit, participants had their height, weight, vital signs, blood glucose levels, and lipid profiles assessed, followed by a thorough health consultation.* (P12).

Students observed tools such as smartwatches and health management apps used to help citizens track their health. These experiences helped students recognize how PHCs promote preventive care through community health management.

### 3.2. Theme 2. Practicing and Overcoming Challenges in Community Nursing

#### 3.2.1. Subtheme 2.1. Understanding the Role of Community Nurses in Preventive Care

Students developed a deeper understanding of the multifaceted nature of community nursing, particularly its focus on preventive care. They observed how community nurses serve as key agents in disease prevention and health promotion, experiencing this role firsthand through their involvement in vaccination programs and health education initiatives.

*It was a day when I could learn about the difference between public health centers and hospitals.… Unlike hospitals, it was good that there were various programs that allowed people at risk or healthy people to check their health in advance and take preventive measures through public health center projects.* (P2)

*Home health management is to improve health behaviors and manage health problems. The goal is to improve health behaviors management by recognizing health status, encouraging healthy lifestyle practices, and improving health knowledge.* (P21).

*During my observation of the pneumonia vaccination process, I completed a questionnaire and had my temperature checked. I received a comprehensive explanation of the typical course of the BCG vaccination, including the natural healing process and the expected appearance of swelling, rash, nodules, ulcers, scabs, and scar formation at the injection site.* (P22).

These experiences helped students recognize both the scope and significance of preventive care in community nursing practice.

#### 3.2.2. Subtheme 2.2. Experiencing Visiting Nursing Services

Students gained valuable experience to home visit nursing services, a cornerstone of community nursing that delivers healthcare directly to patients in their homes. Prior to each visit, nurses confirmed appointments and clarified the visit’s purpose.

*Most of the people receiving home nursing care are older adults, so they don′t seem to care much about their health, wondering how much longer they will live. This made me think that it would be good to have more services for the older adults, such as education, exercise, and activities to improve their health, especially in cities with a large older adults population.* (P3).

*It was my first time learning about theory and seeing it applied in practice. I observed how visiting nursing operates, the criteria for selecting patients, and the types of treatment provided. Many of the elderly patients were health-conscious, often reflecting on how much longer they might live. Given the large senior population in the area, I believe it would be beneficial to implement more activities focused on health education, exercise, and healthy lifestyle habits.* (P17).

*Visiting nursing differs from hospital-based care, as it requires nurses to individually attend to each patient, making the work time-consuming and physically demanding. I noticed that many visits targeted elderly and disabled individuals living in rental housing. It is essential to call in advance to confirm the visiting schedule and exact address before each visit.* (P22).

Students came to recognize the critical importance of health education for this population. These experiences provided students with a comprehensive understanding of visiting nurse services.

#### 3.2.3. Subtheme 2.3. Enhancing Knowledge and Skills

Participation in home visiting services, particularly, proved invaluable for students’ professional development. These experiences deepened their understanding of diverse health needs and nursing services while allowing them to apply classroom knowledge to real‐world situations. Through direct patient interactions, students learned to tailor health information to individual circumstances. They also strengthened their grasp of preventive healthcare principles.

*The instructor educated mothers and their families about the benefits of breastfeeding. The session covered its effects, proper techniques, guidance for breastfeeding, and how to diagnose and address individual issues. In addition to breastfeeding education, information was also provided about other programs and initiatives for mothers.* (P3).

*I realized that a visiting nurse needs to be proficient in a wide range of nursing skills, as well as have good driving skills. Strong communication skills are necessary, as visiting nurses engage in one-on-one conversations to assess health conditions and monitor reactions.* (P12).

*Today is June 9th, Oral Health Day. I learned about the purpose, target audience, and key services of the oral health program. I gained experience with oral care methods, such as proper brushing, toothbrush storage, fluoride application, tartar removal, and at-home tooth filling, as well as oral examinations and health consultations. Since I was not very familiar with oral health topics, everything felt new to me. I now feel confident in educating my family and others about the importance of oral care and helping them improve their oral health.* (P22).

Overall, community nursing experiences enhanced students’ knowledge, skills, and personal growth, better preparing them for their future nursing careers.

#### 3.2.4. Subtheme 2.4. Clarifying Career Options

The practicum helped fourth‐year nursing students reflect on their career aspirations and shaped their interest in community nursing. While the practicum provided important opportunities for reflection, career decisions were often reinforced by existing preferences for hospital environments, which offer diverse clinical experiences. As a result, many students concluded that community nursing was not the right fit for them and preferred hospital‐based nursing instead.

*I observed direct nursing, administrative tasks, telephone work, and human resources management, and the more I practiced, the more inspired I became to pursue the profession. This experience broadened my perspective on career paths beyond the hospital and motivated me to create a new future.* (P12).

*Observing the duties of visiting nurses made me realize that there are numerous fields where I can utilize my nursing license.* (P12).

*My practicum experience took place at a hospital, making the public health center feel unfamiliar. Although the atmosphere was more relaxed than a hospital, it didn’t align with my interests, and I did not find the role of a nurse in a community setting appealing.* (P20).

Students cited several reasons for preferring hospital‐based settings: access to diverse clinical environments, higher earning potential, and opportunities to work with patients requiring intensive nursing care. While a few students solidified their commitment to community nursing, most clarified their preference for other settings based on their professional self‐concept.

### 3.3. Theme 3. Leading Health Promotion Projects as a Nursing Professional

#### 3.3.1. Subtheme 3.1. Community Health Assessors

Students learned to evaluate the unique characteristics and needs of communities where nurses practice. They identified key factors such as aging populations, seasonal workforce shifts, and sociodemographic and economic conditions. These assessments provided valuable insights into specific health priorities and challenges, highlighting the importance of tailoring healthcare services to each community’s structure and lifestyle.

*I became aware of the vulnerable groups around us, especially older adults living alone. Many were not managing their health effectively and often took medications without proper guidance.* … *Before starting the project, I recognized the need to understand their health issues in detail.* (P3).

*As it is currently the farming season, many adults are particularly busy and have been unable to visit the mobile clinic. The public health center has noted that tick-borne infections often begin to occur in April. They are identifying common diseases in this rural area.* (P17).

*The community nursing report was discussed during the conference, providing an opportunity to learn more about specific issues in the city area. I gained objective information through statistics and survey data on health issues I hadn’t previously considered, such as smoking cessation, dementia, and tsutsugamushi mites.* (P17).

This experience enabled students to develop intervention plans that address residents’ needs, ultimately aiming to improve healthcare accessibility and advance health equity for all community residents.

#### 3.3.2. Subtheme 3.2. Specialized Practitioners

Students stressed the importance of specialized knowledge, particularly for breastfeeding education and vaccinations, as well as strong communication skills for engaging diverse groups. They gained experience delivering age‐appropriate health education and linking members to resources.

*The oral health teacher uses a model of a tooth and compares it to a cross-section of an apple to simplify the explanation. I realized that I had learned how to use accessible language, but I wasn’t applying it effectively.* (P1).

*I saw children aged 5–7 at a daycare center come to the oral health center to receive education on cavity prevention. I observed them watching a cavity prevention video through a cartoon called “Kongsooni” that was appropriate for the children’s level. I saw that they were operating various health education programs appropriate for their age…* (P2).

*To prevent severe fever with thrombocytopenia syndrome (SFTS), a tick-borne infectious disease, we educated people to wear bright-colored, long-sleeved clothing, hats, socks, and gloves, as well as to apply tick repellent before engaging in outdoor activities. Additionally, we instructed them on infectious disease management practices, such as removing their clothing immediately after outdoor activities, washing the clothes, and taking a shower.* (P15).

These experiences underscored the multifaceted expertise needed to meet community health needs, emphasizing specialized knowledge, practical skills, and adaptable communication strategies.

#### 3.3.3. Subtheme 3.3. Collaborate With Other Experts

Students emphasized the importance of interprofessional collaboration, observing community nurses working with specialists to provide holistic care and improve service quality. This collaboration was evident in multiple settings. Nurses assessed needs, provided counseling, set goals, and monitored health indicators.

*We joined a mobile healthcare project to prevent chronic diseases, involving coordinators, nurses, doctors, nutritionists, and exercise specialists. Nurses assessed users′ needs, provided counseling, set goals, monitored health indicators, and referred users to doctors when needed. Each expert fulfilled their role, and we observed effective teamwork.* (P12).

*The Dementia Care Center provides counseling, registration, early screening, and cognitive enhancement programs. Nurses, social workers, and occupational therapists collaborate to deliver comprehensive dementia support services.* (P12).

These experiences revealed the range of skills needed in community health: clinical expertise, practical abilities, flexible communication, and the ability to engage in interprofessional collaboration.

## 4. Discussion

Most students entered the practicum with limited PHC experience or interest. Fourth‐year students showed limited basic knowledge of community nursing despite completing the required theoretical coursework. This suggests a gap between theoretical education and practical preparation [16]. However, the 2‐week placement meaningfully improved their understanding of PHC’s role in promoting health equity and community health. Through PHC exposure, students gained a broader perspective on nursing that includes community healthcare needs. These findings are consistent with previous studies that reported similar challenges regarding students’ limited knowledge and interest in community care [6, 15]. Educational improvements are needed to better connect theory and practice, beyond traditional content‐focused instructional approaches. Students need earlier and more sustained exposure to community care environments throughout the curriculum, rather than exposure limited to final‐year practicums [16, 17]. This systematic approach to curriculum design facilitates the development of core competencies for community‐centered practice. These competencies support future nurses in addressing increasingly complex public health requirements and inform curriculum redesign and practical training.

This study examined students’ post‐COVID‐19 community nursing experiences, focusing on preventive care, visiting nursing services, and public health initiatives. While previous studies approached practicum from a treatment‐centered perspective [18, 19], this study offers unique insights into prevention‐centered community health nursing education. Prior research study that nursing students often hold negative views of community care, perceiving it as chronic disease management for older adults with limited complexity [15]. However, the findings show that students recognized the essential role community nurses play in health promotion. They observed how immunizations and screenings prevent disease outbreaks and enable early detection [15], and witnessed visiting nurses supporting vulnerable populations [20]. Reflective journals have proven effective for exploring nursing students’ learning experiences and fostering their professional identity in community nursing [9, 11, 12]. The findings of this study further underscore the value of reflective journaling for understanding community nursing practice realities.

Community nursing practice provided a valuable opportunity for students to clarify their career aspirations. While a few developed new interest in this field, most reaffirmed their preference for hospital‐based roles by comparing their experiences across the two practice settings. Previous studies have shown that clinical exposure, preparedness, and perceived work importance influence nursing students’ career preferences [15, 21]. These findings suggest that community practice contributes to students’ professional self‐understanding by allowing them to evaluate their clinical fit and reflect on the reasons underlying their career choices. According to a previous review, although students generally value community nursing, many continue to favor hospital positions because of broader clinical exposure, higher salaries, and access to advanced medical technologies [15]. Further research using surveys and interviews is needed to examine how specific aspects of community nursing experiences shape career decision‐making. The importance of community nursing has grown considerably, especially during the pandemic. It is essential to address the longstanding hospital‐centered orientation of nursing education. Students need broader and more systematic exposure to community settings, including health promotion projects, home visits, school health programs, and workplace health management [15, 22].

These findings about students serving as health assessors and practitioners are consistent with previous community nursing research [6, 15]. Hands‐on experience in real‐world community health settings helps students develop essential skills, learn to communicate in culturally sensitive ways, and understand preventive care [23–25]. However, students often perceive community settings as lacking opportunities to practice complex technical skills, viewing this work as “not real nursing”—something they believe is better learned in hospitals [15]. These results underscore the growing importance of community nursing during the COVID‐19 [26]. Nursing education institutions need to redesign their curricula to better prepare students for pandemic emergencies and the complex health challenges communities face. This means adding modules on health promotion, pandemic preparedness, and community health issues, while also making sure students get enough practicum hours. Postpandemic curricula should give students the nursing knowledge and skills they need to respond effectively to emerging health situations in community settings.

Students recognized that they could potentially lead health promotion initiatives as expert assessors and practitioners. However, research shows that workplace constraints—including organizational limitations and heavy workloads—often prevent public health nurses from fully carrying out their intended community nursing roles [27]. This finding points to an important opportunity. Current community health nurses should see students as their future colleagues and provide them with strong role modeling and mentorship. By actively engaging with students, practitioners can reinforce the value of community health nursing and help develop competent professionals.

The study’s findings about collaborative professionals in community settings underscore the critical role of teamwork in improving community health, which is consistent with the interdisciplinary approach to health care [28–30]. Students’ reflections emphasized how valuable it is to collaborate with professionals involved in community care. This holistic approach shows how an integrated care model can improve service quality by addressing the multifaceted needs of individuals with chronic health conditions and their families [31]. When family support and community resource utilization are incorporated into community nursing theory and practice, this deepens students’ understanding of collaborative practice. By strengthening team‐based interdisciplinary learning and expanding field‐related training, programs can help students collaborate more effectively across disciplines, thereby preparing them for future challenges in community nursing.

### 4.1. Limitations

First, this study relied solely on students’ reflective journals, which may not fully capture the complexity of their practice experiences. While reflective journaling provides rich qualitative data, it is inherently subjective and may miss key themes that other data collection methods could reveal.

Second, there is a methodological limitation because reflective journals were assigned for academic credit, which may have led students to write what they thought their instructors wanted to see. Knowing their journals would be graded may have prompted students to emphasize positive experiences or hide negative ones, introducing social desirability bias that could compromise the authenticity and validity of the findings [32]. Using these evaluation‐driven reflective journals for research without getting explicit consent upfront for secondary use presents a challenge, since the data may not truly reflect authentic experiences. Future consent forms should clearly explain that reflective journals may be used for both academic grading and research. It is important to make clear that participating in research will not affect students’ grades and to include specific consent for using journals they have already submitted.

Finally, this study had limitations in sample representativeness, as less than half of the practicum students participated in reflective journal analysis. This means potential selection bias cannot be ruled out [32], as nonparticipating students may have had different perspectives or experiences.

Despite these limitations, this study provides valuable insights into how students experienced and perceived community nursing practicums after COVID‐19, providing foundational data for exploring where community nursing education should head in the changed educational landscape following the pandemic.

## 5. Conclusions

This study examined students’ experiences during post‐COVID‐19 community nursing practicums, revealing insights into their understanding, engagement, and professional growth. The findings can inform practical strategies to address students’ limited awareness of community nursing roles and support their professional development. Practicum experiences influenced some students’ career preferences by providing opportunities to observe community health nurses leading health promotion initiatives as health assessors and professional practitioners working with interdisciplinary teams. Programs should also strengthen collaboration closely with practicum sites to ensure students have more opportunities to actively engage in hands‐on practice by collecting, summarizing, and analyzing data with health assessment tools, rather than observation. This approach to community nursing practicum education will help develop nurses prepared for future community health challenges.

## Funding

This work was supported by Research Fund of the Howon University.

## Consent

Consent from participants was obtained solely for the purpose of this specific research during the IRB approval process, precluding external sharing or broader dissemination.

## Conflicts of Interest

The author declares no conflicts of interest.

## Supporting Information

The supporting data accompanying this manuscript include additional table. Table S1 provides Reporting Standards for Qualitative Research (SRQR) to enhance the transparency and quality of health research. Additionally, the Research Funding Agreement and Research Data are provided to offer further evidence regarding the funding support and the transparency of the qualitative data obtained from the research participants.

This supporting information is designed to provide additional context and deeper insight to support the conclusions outlined in the main text.

## Supporting information


**Supporting Information** Additional supporting information can be found online in the Supporting Information section.

## Data Availability

The data supporting the findings of this study are not publicly available due to ethical restrictions.
