# Supplementary material for: Understanding and Challenges of Community Nursing Practicums After COVID‐19: A Qualitative Study
Source: Nurs Res Pract. 2026 Feb 12;2026:6657747. doi: 10.1155/nrp/6657747 (PMC12901696; doi:10.1155/nrp/6657747)
Supplement: Supplementary file 1 — Supporting Information Additional supporting information can be found online in the Supporting Information section. [file NRP-2026-6657747-s001.zip › 20241231_Research Funding Agreement_Public Health Center.pdf]

연 구 협 약 서

☐ 연구과제명 : 코로나19 이후 간호대학생의 보건소 실습 경험: 주제분석
☐ 계약당사자 : (갑) 호원대학교 총장
(을) 호원대학교 이수미

상기 연구과제에 대하여 (갑)과 (을)은 다음과 같이 연구계약을 체결한다.

제1조(목적) 교내학술연구비의 효율적인 관리를 통해 연구계약서의 연구목적을 달성하는데 있다.

제2조(연구비 지급) ① 연구비는 전액 (갑)이 부담하며 (갑)은 아래와 같이 (을)에게 지급한다.

제3조(연구기간) 본 연구과제의 연구기간은 2025년 01월 02일부터 2026년 01월 01일까지로 한다.

제4조(연구과제수행) (을)은, 「교내연구비 지원 계획서」를 준수하여 성실히 연구를 수행하여야 한다.

제5조(연구결과보고서 제출) (을)은 (갑)이 지정한 기간 이내에 본 연구과제의 게재 의무지에 게재한 최종결과보고서(게재예정증명서 제외) 1부를 (갑)에게 제출하여야 한다.

제6조(계약의 해석) 연구수행에 필요한 사항으로서 본 계약서에 기재되어 있지 않은 사항은 (갑)이 정하는 「교내연구비 지원 계획서」에서 정하는 사항을 따르되, 해석상 의문이 있을 경우 (갑)의 해석에 따른다.

제7조(계약의 변경) (을)이 연구수행 중 부득이한 사유가 발생할 경우, (갑)과 (을)이 상호 협의하여 연구과제명은 변경할 수 있으나 연구기간 및 연구책임자는 변경할 수 없다.

제8조(계약의 효력) 본 계약은 (갑)과 (을)이 서명·날인한 날로부터 연구결과보고가 완료될 때까지 유효하다.

첨 부 : 연구계획서 1부.

2024년 12월 31일

(갑) 호원대학교 총 장 강 희 성 (인)

(을) 호원대학교 소속: 간호학과 직위: 조교수 성명: 이수미 [signature]
