# Supplementary material for: Understanding and Challenges of Community Nursing Practicums After COVID‐19: A Qualitative Study
Source: Nurs Res Pract. 2026 Feb 12;2026:6657747. doi: 10.1155/nrp/6657747 (PMC12901696; doi:10.1155/nrp/6657747)
Supplement: Supplementary file 1 — Supporting Information Additional supporting information can be found online in the Supporting Information section. [file NRP-2026-6657747-s001.zip › Research DATA_Public Health Center.pdf]

1. 오늘 실습경험의 의미 있는 점은 무엇인가?

오늘은 보건소 첫날이여서 선생님들이 OT 해주셨는데, 보건소의 건물 구조나 보건소 내 있는 부서들에 대해서 알려주셨다. 또한 어떤 사업을 진행하는거라 여러 캠페인과 감염병의 위험성에 대해서 알려주셔서 의미있었다.

2. 오늘 실습을 하는 동안 나의 생각과 느낌은 어떠했는가?

오늘 실습하면서 지역사회 간호학 이론에서만 보았던 건강보험공단이나 노인장기요양 보험을 실제로 보았을때 지역 사회에서 보건 정책이 어떻게 녹아들어가있는지 체감할수 있어서 신기했다.

3. 오늘 실습을 통해 새롭게 알게 된 것은?

오늘은 건강보험공단에서는 국민의 질병, 부상에 대한 예방, 진단, 치료, 재활과 출산, 사망에 대하여 보험급여를 실시함으로서 국민보건을 향상시키는 목적으로. 모든 국민이 지역가입자나 직장가입자로 나뉘어서 의무적으로 가입 해야한다. 지급형태로는 현물급여, 현금급여, 건강관리제공이 있다. 또한 노인장기요양보험도 공단에서 5등급을 나누어 지원한다는 것도 알게되었다.

4. 오늘 실습을 통해 새롭게 배운 것들이 향후 간호실무에 어떻게 적용될 수 있겠는가?

SFTS라는 진드기매개감염병에 대해서 알게되었다. 병원에서 아닌 지역사회에서 자주 나타날수 있는 주제로 강의를 해주셔서 색달랐다. 진드기에 감염 시 증상으로는 고열, 오한, 구토, 검은딱지가 있다. 예방으로는 긴옷, 농작업복을 입고 일이 끝난후 바로 샤워하도록 한다. 치매안심센터에서는 치매의 종류와 치매 초중 말기단계의 변화등과 치매파트너에 대해서 배울수있었다.

5. 오늘 실습에서 좋았던 점과 아쉬웠던 점은 무엇인가?

오늘 오티하면서 다들 친절하시고 자세하게 설명해주셔서 좋았다. 하나 아쉬웠던 점은 오늘은 이동 진료 팀 실습이였지만 출장일이 없어서 실제로 어떤 일을 해내는지에 대해서는 따로 찾아 보는 수밖에 없어서 그런 점은 아쉬웠다.

1. 오늘 실습경험의 의미 있는 점은 무엇인가?

오늘은 방문 건강관리 팀에 갈수 있었다. 직접 경로당에 가서 어르신들 V/S이나 BST를 재면서 지역사회 속에서 어떻게 간호가 제공되는 지 직접 볼수 있는 시간 들이어서 의미있었던 것 같다.

2. 오늘 실습을 하는 동안 나의 생각과 느낌은 어떠했는가?

오늘 실습하는 동안 선생님들도 다들 친절하시고 자세히 알려주려고 하셔서 좋았던것같다. 방문 건강관리는 보통 집에서 하는 줄 알았는데, 그게 아닌 노령 사람이 많이 몰려있는 경로당에서 하게 된다는게 신기했던 것 같다.

3. 오늘 실습을 통해 새롭게 알게 된 것은?

- 방문건강관리 사업이란: 보건소에 소속된 방문간호사가 각 가정을 방문하여 가족과 건강 문제를 가진 가구원을 발견하여 질병예방 관리, 건강증진을 위해 건강 서비스를 제공하는 것
- 대상: 기초수급자, 독거노인, 차상위계층, 장애인, 독거노인, 타기관에서 의뢰한 건강 문제가 있는 대상자.
- 목표: 건강행태개선(건강상태인식, 건강생활 실천유도, 지식향상), 건강문제관리(건강문제 정기적 스크리닝 증상조절, 치료 순응 향상)

4. 오늘 실습을 통해 새롭게 배운 것들이 향후 간호실무에 어떻게 적용될 수 있겠는가?

- 간호사분들 14명으로 이루어지고 각 한분씩 읍면 동을 맡아 경로당을 돌면서 혈압, 혈당을 재고 혈압이나 혈당 관리에 대한 주의점들을 교육시키고 요실금 예방을 위한 케겔운동이나, 환절기 때 주의사항들에 대해서도 간단한 강의를 하는 것을 볼수있었다

5. 오늘 실습에서 좋았던 점과 아쉬웠던 점은 무엇인가?

방문 건강관리 팀 선생님들이 다들 친절하셔서 좋았다. 방문 간호에서 어떤 일을 하시고 구성은 어떻게 되어있는지랑, 관할의 동, 면 단위까지 가 볼수 있어서 좋았던 것같다. 직접 지역사회에 나가서 간호를 제공해볼수 있는 것이 실질적으로 정책들이 어떻게 이루어지고 또한 제공되는지 알수 있는 시간이였던 것 같다.

1. 오늘 실습경험의 의미 있는 점은 무엇인가?

오늘은 보건소 행사진행되어 모든 부서의 특성에 맞춰서 행사가 진행되었다. 직접적으로 지역사회에서 사람들에게 보건 교육이나 간단한 검사들을 제공해 주어서 이러한 행사에 많은 사람들이 오고 보고 간다는 것이 신기하였다.

2. 오늘 실습을 하는 동안 나의 생각과 느낌은 어떠했는가?

오늘은 보건소 안에서가 아닌 외부 공원에서 주민들이 많이 지나가는 곳에서 본격적 혈당도 재고 혈압을 재면서 간단한 정상수치나 생활 습관에 대해서 배울수 있었다. 이런 행사도 하면서 적극적으로 주민들에게 다가가는 방식이 신선하고 꽤나 효과적인 것을 알수 있었다.

3. 오늘 실습을 통해 새롭게 알게 된 것은?

오늘은 지역사회 내에서 보건소의 홍보전략으로 여러 부스에서 다양한 경품 제공과 함께 조폭자들 주면서 지역사회 보건지식을 알려주는 것을 배웠다. 구강보건, 혈관관리 뿐아니라 금연 캠페인, 정신 건강 관련된 설문지 작성이나 리플렛 측정등을 주민들이 보건소 내에서 아닌 밖에서 쉽게 접하는 것을 보고 이런 행사들이 보건 관리에 향상될 수 있다고 생각되었다.

4. 오늘 실습을 통해 새롭게 배운 것들이 향후 간호실무에 어떻게 적용될 수 있겠는가?

여러 캠페인을 통해 지역 주민이 보건 교육을 많이 접하다 보면 쉽게로 실천하는 부분들도 늘어날 것이고. 이런 현상들이 많아지면 효과적인 지역사회 속 보건 교육및 관리들이 향상될것 같다고 생각한다.

5. 오늘 실습에서 좋았던 점과 아쉬웠던 점은 무엇인가?

오늘은 행사 하느라 바빴지만 그래도 우리도 직접 행사에 참여도 해보고 행사진행도 하면서 몰랐던 보건 교육도 받아보고 해서 많은 것을 배운 것 같다. 나름 유익한 정보들도 많아서 좋았었다.

1. 오늘 실습경험의 의미 있는 점은 무엇인가?

오늘도 행사를 하면서 오늘은 혈압과 혈당을 재면서 당뇨 환자들의 당 초과 수치에 대해서만 배웠었는데 당수치의 일반적인 경향 주의 권고 수치와 같이 세분화된 수치에 대해서 새롭게 알게 된 내용이었다.

2. 오늘 실습을 하는 동안 나의 생각과 느낌은 어떠했는가?

오늘은 V/S, 혈당 등에 잡일들을 많이 해서 조금은 바쁘고 힘들었었다. 또 집담회에서 지역사회 과제를 하면서 잘 몰랐던 부분이나 지역사회 과제 내 잘못된 부분으로 과제를 작성했던 것에서 수정하고 알수 있게 해주셔서 좋았다.

3. 오늘 실습을 통해 새롭게 알게 된 것은?

지역사회 사례 보고서에 관련 문제요인과 진단에 대한 연관성이나, 자료 수집도와 같은 부분들이 지도를 통해 수정할수 있었고, 행사에서 혈당의 정상범위는 120 이하고 식후 2시간 이후엔 140 이하까지 정상이고 200 이상이면 당뇨병의 위험이 있다고 배울수 있었다.

4. 오늘 실습을 통해 새롭게 배운 것들이 향후 간호실무에 어떻게 적용될 수 있겠는가?

오늘은 지역사회내 보건 교육 제공하는 법에 대해서 배울수 있었다. 또한 바이탈과 혈당을 재면서 위험 수치가 높으신분들은 보건소내 당뇨 혈압 교실과도 연계 시켜 주는 것을 보고 보건 교육뿐만이 아닌 직접적인 지역사회 내 고위험 진단을 받는 환자들도 찾아낼수 있는게 매우 기발한 방법이었던 것 같다

5. 오늘 실습에서 좋았던 점과 아쉬웠던 점은 무엇인가?

오늘 행사하면서 내가 직접 해보기도 하고 행사 진행도 해보았는데, 구강보건이나 금연시 폐 기능에 대한 정보들도 실제로 볼수 있어서 유익했다. 오늘은 선생님들이 아이스크림도 주고 물도 주셔서 힘들었는데 고마웠었다.

1. 오늘 실습경험의 의미 있는 점은 무엇인가?

오늘은 접수실에서 실습을 하였다. 이곳에 오니 원무과일도 하시고 길 안내도 해서 보건소내에 어떤 일들을 하는 것인지 좀 더 자세하게 알수 있었다. 보건소는 보통 결핵 검사나 예방접종, 코로나 검사만 한것을 보았는데 생각보다 많은 부분들을 담당해서 신기하였다.

2. 오늘 실습을 하는 동안 나의 생각과 느낌은 어떠했는가?

보건소 내에서 생각보다 많은 일들을 하고 있는 것을 알수 있었다. 구강보건이나 전염병 예방접종 뿐만 아니라 행정적인 부분이나 정신건강 사업 금연 치료, 당뇨 검사나 암 난임 희귀난치, 영유아 건강검진 지원도 해주는것을 볼수 있었다.

3. 오늘 실습을 통해 새롭게 알게 된 것은?

보건소 내에서는 예방접종 뿐만 아니라 A형 B형 항체 검사나 건강 클리닉 이나 영양 플러스 사업. 치매나 아토피 지원도 해주고 한방진료나 물리 치료 진료도 봐주고. 보건소 내 진료도 봐주시고 또한 사전 연명 중단 동의서도 보건소 내에서 작성할수 있다는 것을 보고 신기하였다.

4. 오늘 실습을 통해 새롭게 배운 것들이 향후 간호실무에 어떻게 적용될 수 있겠는가?

오늘 실습을 통해 보건소 내의 여러 부서들이 있고 여러 사업들을 하고 있다는 것을 알수 있었다. 이처럼 임상 밖에서도 간호사가 할수 있는 일들이 여러가지 다양하다는 것을 알 수 있었다.

5. 오늘 실습에서 좋았던 점과 아쉬웠던 점은 무엇인가?

오늘은 접수실에서 실습하면서 보건소에 오시는 주민분들에게 안내해주는 역할을 했는데 보건소 내 어떤 업무 곳이 어디에 있는지 잘 몰라서 조금은 당황 했던 것이 있었다.

1. 오늘 실습경험의 의미 있는 점은 무엇인가?

오늘은 예방접종실에서 실습을 하였다. 보건소 내에서 진행하는 접종 종류에 대해서 알게 되었고 같은 질병이여도 백신의 종류가 다르다는 것을 알게 되었다. 또한 국가에서 정책적으로 관리해야 할 질병들이 꽤나 많은 것을 알 수 있었다.

2. 오늘 실습을 하는 동안 나의 생각과 느낌은 어떠했는가?

생각한 것 이상으로 예방접종 지원 대상이 많아졌고 해당되는 감염병 종류들도 많아졌던 것을 보고 놀랐었다. 이러한 부분들이 의료보험의 혜택이 돌아가는 구나라고 생각하면서 우리나라 의료 보험 서비스가 잘되어 있는 구나라는 생각이 들었다.

3. 오늘 실습을 통해 새롭게 알게 된 것은?

- 국가 필수 예방접종은 만 12세 이하 아동이 해당되고 아이의 나이에 따라 차례대로 접종해야 하는 부분도 되게 한눈에 잘되어있는 것을 볼 수 있었다. 예전에는 자궁경부암 같은 것은 보험이 안되어 있었지만 지금은 일정 나이 일시 국가에서 무료접종도 제공해준다는 것을 알게되었다. 또한 무료접종으로 어르신 폐렴이나 고위험군 A형 간염검사, 및 A형 간염 접촉자 등에게도 무료로 접종을 제공한다는 것을 배웠다.

4. 오늘 실습을 통해 새롭게 배운 것들이 향후 간호실무에 어떻게 적용될 수 있겠는가?

폐렴 접종 후 말해야 할 것들은 샤워가 가능하고, 2-3일 정도 근육통이 있을 수 있음을 설명하고 오늘 하루정도는 푹 쉬어야 한다는 것을 말해드린다. 또한 근육통이 심할 시 타이레놀을 먹어도 된다는 것들을 말해드려야 한다는 것을 배웠다.

5. 오늘 실습에서 좋았던 점과 아쉬웠던 점은 무엇인가?

오늘 실습에서 좋았던 점은 진료실과 예방접종실에서는 어떤 접종과 접종 기간에 대해서 자세하게 나와있어서 약명, 용량, 경로 등에 대해서 알 수 있어서 좋았다. (수두, MMR, 일본뇌염 → SC, BCG → ID, B형, DTap, 뇌수막염 → IM으로)

1. 오늘 실습경험의 의미 있는 점은 무엇인가?

오늘은 건강보험 공단에서 실습을 하였다. 건강보험공단에서 어떤일을 하는지와 건강보험공단 채용 공고에 대해서도 자세하게 알려줘서 의미있었다. 우리가 하는 일은 미검진자가 검사하도록 독려하는 전화를 하는 일이였다. 나는 여러 검사를 공부할수 있어서 의미있었다.

2. 오늘 실습을 하는 동안 나의 생각과 느낌은 어떠했는가?

오늘은 실습 때 전화 하도록 하셔서 조금은 떨렸었다. 하지만 선생님이 매뉴얼도 알려주시고 어떻게 하는지 시범도 먼저 보여주셔서 감사했다. 처음에는 떨렸지만 계속하면서 선생님들이 많이 도와줘서 익숙해 질수 있었던 것 같다.

3. 오늘 실습을 통해 새롭게 알게 된 것은?

오늘은 간암 상반기 검진을 독려하는 전화를 했는데 간암은 보통 40세 이상 간암 발생 고위험군이 대상으로 검진비용은 10% 본인부담이다. 간암은 6개월 주기로 상반기 하반기로 나누어져있어서 간초음파와 표지자검사로 혈액검사를 한다고 한다.

4. 오늘 실습을 통해 새롭게 배운 것들이 향후 간호실무에 어떻게 적용될 수 있겠는가?

오늘은 간암 검사 독려에 대해서 알수 있었고 기본적으로 민원인과의 응대 태도에 대해서 배울수 있었다. 또한 건강보험 공단에서는 보험금여 심사나 노인장기요양보험, 건강검진, 금연치료 등을 제공한다는 것을 알 수 있었다.

5. 오늘 실습에서 좋았던 점과 아쉬웠던 점은 무엇인가?

선생님들도 다 친절하시고 편하게 대해주셔서 고마웠다. 처음에는 전화 하는게 조금 떨렸지만 그래도 매뉴얼이 다 있어서 안심하고 잘 응대할수 있었던 것 같다.

1. 오늘 실습경험의 의미 있는 점은 무엇인가?

오늘도 건강보험공단에서 실습하였다. 채용 정보에 대해서 실질적인 조언들도 들을수 있었고 또한 건강보험공단에서 제공하는 검사에 대해서도 어떤 종류가 있는지 알수 있어서 좋았다.

2. 오늘 실습을 하는 동안 나의 생각과 느낌은 어떠했는가?

오늘은 실습 때 계속 전화를 돌리다보니 받는 사람들이 모두 다르다는 답변을 하는게 신기하다고 느꼈다. 여러 궁금한 것을 묻는 사람도 있었고 단답만 하는 사람도 있었다. 오히려 내가 모르는 부분도 물어보셔서 그런 부분에 대해서 이후에 자세하게 알수있어서 좋았다

3. 오늘 실습을 통해 새롭게 알게 된 것은?

국가건강검진은 일반검진, 대장암, 간암, 자궁경부암, 위암 등을 받을수 있다. 각 장기들마다 검진을 받을수 있는 연령대가 다른데 나의 경우에는 자궁경부암을 받을수 있었다. 건강보험공단 내에서 받을수있는 검사가 생각보다 많고 자세하다는 것을 알게되었다.

4. 오늘 실습을 통해 새롭게 배운 것들이 향후 간호실무에 어떻게 적용될 수 있겠는가?

오늘은 건강검진 채용에 대해서 잘 알려주셨는데 병원에 안다녀도 바로 지원 가능할 수 있고 직업교육을 받아야 하며 또한 이제는 저출생으로 건강보험공단에서 채용이 거의 안뽑을수 있다고도 말씀해 주셨다.

5. 오늘 실습에서 좋았던 점과 아쉬웠던 점은 무엇인가?

오늘도 다른 선생님이 우리를 맡으셨는데 너무 잘해주시고 건강 보험 공단에 대해서 실질적인 조언들도 해주셔서 병원 외에도 취업할수 있는 곳들은 여러개 있구나라고 생각이 들었다.

P2

1. 오늘 실습경험의 의미 있는 점은 무엇인가?

오늘은 "건강증진실"에서 실습을 하게 되었다. 보건소 내에 건강증진실이라는 곳에서 어떤일을 하는지 알게 된게 의미 있었다. 건강증진실은 시민들에게 보다 나은 건강생활 실천 서비스를 드리고자 운영되는 곳이다. 예약을 통해 오신 분들에게 체성분검사 (인바디)를 측정하고 최소 2개월후에 재측정을 하여 건강상담을 하고 있다.

2. 오늘 실습을 하는 동안 나의 생각과 느낌은 어떠했는가?

보건소에서 처음 실습을 해보는데 보건소에서는 정확히 무슨일을 하는지 그동안은 잘 몰랐었다. 지역사회간호 이론에서 들었던 걸로 대충 짐작만 했었는데 담당 선생님께서 해주신 얘기를 듣고 생각보다 다양한 사업들을 운영하고 있다는 사실을 알게 되었다. 지역의 시민들의 건강을 예방하고, 증진 시키기 위해 영양관리, 건강증진, 이동진료, 구강보건 등 다양한 사업이 있다는 것은 지역의 많은 시민들에게 알리는 것도 중요한것 같다고 생각하게 되었다.

#보건소 인식

1-1

3. 오늘 실습을 통해 새롭게 알게 된 것은?

보건소 모바일 헬스케어 사업을 운영하고 있다는 것을 새롭게 알게 되었다. 예약을 통해 온 사람들에게 피검사, 혈당검사, 인바디등의 검사를 하여 개인별로 체중, 근육, 체지방 등을 분석해주고, 식습관 개선 교육과 운동에 대해 교육을 해준다. 그리고 스마트 워치를 제공하여 집중상담, 식단사진, 활동량계 정보, 운동, 식사일기 등을 등록하여 어플과 연결하여 정보를 제공 받는다. 한국건강증진 개발원과 보건소에서 제공하는 건강 정보들을 통해 대상자 들을 건강 증진 시켜주는 이러한 사업이 있다는 것을 알게 되었다.

4. 오늘 실습을 통해 새롭게 배운 것들이 향후 간호실무에 어떻게 적용될 수 있겠는가?

보건소 모바일 헬스케어 라는 사업은 비만, 고혈압, 고혈당 등 대사 증후군의 위험이 높은 일반인에게 널리 알려 많은 분들이 미리미리 건강 예방을 할 수 있도록 적용하면 좋을것 같다. 그리고 대상자들에게 맞게 사전 검진을 바탕으로 운동, 식습관 등에 대해 교육 하면 될것 같다

2-1

5. 오늘 실습에서 좋았던 점과 아쉬웠던 점은 무엇인가?

보건소에서 어떤 일들을 하는지 궁금했는데 실습을 하면서 직접 현장 에서 보게 되어 좋았다. 병원과는 다르게 위험군에 있거나 건강한 사람들이 보건소의 사업들을 통해 미리 건강을 점검하고, 예방 할수 있는 프로그램들을 다양하게 알게 되어 좋았다.

예방중심

1. 오늘 실습경험의 의미 있는 점은 무엇인가?

건강보험공단에서 실습을 하게 되었는데 건강보험공단에서 하는 업무 중 암검진 유선독려를 하게 되었다 업무 중 하나를 직접 해보게 되면서 조금이라도 건강보험공단이 어떤일을 하는지 몸으로 느끼게 되어 의미 있었다.

2. 오늘 실습을 하는 동안 나의 생각과 느낌은 어떠했는가?

암검진 대상자들에게 전화를 통해 검진을 받으라고 설명해 주면서 나라에서 국민들을 위한 혜택들이 우리가 알고 있는 것보다 훨씬 다양함을 알수 있었고, 평소에 나에게도 이런 전화가 오면 무심코 받거나. 귀찮아 했던 경우가 많은데 별 일이 아니라고 생각 했지만 현장에서는 대상자들을 위해 많이 노력하고 있는 것을 보면서 앞으로는 좀 더 신경써서 전화에 응답해야 겠다고 느꼈다

3. 오늘 실습을 통해 새롭게 알게 된 것은?

병원에서 대상자들이 건강검진을 받으면 병원에서 공단으로 검진한 항목을 신고해줘서 공단에서는 그러한 데이터를 바탕으로 국가건강검진 대상자들을 관리하고 있음을 알게 되었다. 나이별로, 위험군별로 받아야 하는 항목들을 보고 대상자들에게 앞으로의 건강 검진에 대해서도 안내하고 있음을 알수 있었다.

4. 오늘 실습을 통해 새롭게 배운 것들이 향후 간호실무에 어떻게 적용될 수 있겠는가?

국가 건강검진의 종류와 검진 대상자들의 나이별, 위험군별을 알고 대상자들에 맞는 검진을 빠지지 않고 잘 받아서 질병을 예방할수 있도록 적용 하면 좋을것 같다.

5. 오늘 실습에서 좋았던 점과 아쉬웠던 점은 무엇인가?

건강보험공단에서 하는 일 중 작은 업무를 직접 해보게 된 점이 좋았다. 지역사회간호 실습은 어떤 업무들을 하는지 궁금했는데 이렇게 다양한 일들이 많이 있음을 직접 눈으로 보고, 설명 듣고, 해볼수 있어서 좋았다.

1. 오늘 실습경험의 의미 있는 점은 무엇인가?

국민건강 보험공단에서 무슨일을 하는지 배우게 되었다. 직렬은 행정직, 건강직, 요양직, 전산직이 있으며 행정직은 공인 어학성적(TOEIC, NEW TEPS, TOEFL, OPIC, TOEI speaking, G-TELP)이 있거나 공단, 일산병원 중 한 기관에서 130일 이상 근무하거나 청년인턴으로 근무한 사람이 가능하며 간호사 면허가 있으면 면허를 이용하여 건강직, 요양직에 가산점을 받아 지원이 가능하다고 한다.

2. 오늘 실습을 하는 동안 나의 생각과 느낌은 어떠했는가?

국민건강보험이 병원하고 얼마나 연관이 있고, 우리 에게 어느정도 의미가 있는지 그동안은 생각해 본적이 없는데 국민건강 보험공단에서 실습하면서 선생님들께서 업무 하시는 것을 조금 관찰하고, 들어보니 우리 삶에서 우리가 다치거나, 아플때 반드시 필요하고 우리에게 많은 도움을 준다는 것을 알게 되었다. 또한 병원하고도 계속 연락 하고 연계되어서 많은 사람들이 보험을 제대로 받을수 있도록 많은 도움을 주는 곳임을 깨닫게 되었다.

3. 오늘 실습을 통해 새롭게 알게 된 것은?

노인장기 요양보험에 대해 이론에서 보다 더 자세히 알게 되었다. 노인장기 요양보험은 65세이상 노인 또는 치매, 뇌혈관성 질환 등 노인성 질병이 있는 65세 미만의 자가 6개월 이상동안 혼자서 일상생활을 수행하기 어려워 수급자로 판정 받은 경우, 장기 요양기관 으로부터 신체활동, 또는 가사활동, 인지활동 지원등의 장기 요양급여를 받을수 있는 제도로 장기요양 등급 판정 위원회에서 총 6등급으로 등급 판정을 한다. 장기요양 1등급은 심신의 기능상태 장애로 일상생활에서 전적으로 다른사람의 도움이 필요한 자로서 장기요양인정 점수가 95점 이상인자로 가장 도움이 필요한 등급이며 장기요양 인지지원등급은 치매환자로서 장기 요양 인정 점수가 45점 미만인자이다.

4. 오늘 실습을 통해 새롭게 배운 것들이 향후 간호실무에 어떻게 적용될 수 있겠는가?

나중에 대상자가 노인장기 요양보험에 대해 잘 알지 못해서 장기 요양 급여를 못받고 있으면 오늘 배운 내용을 토대로 대상자에게 보험에 대해 설명해주고, 안내해 줄수 있을것 같다. 수급자에게 장기 요양 급여를 제공하는 기관을 설명 해주어 많은 대상자들이 우리나라에서 제공하는 많은 혜택을 누릴수 있도록 실무에서 도움을 줄수 있을것 같다.

5. 오늘 실습에서 좋았던 점과 아쉬웠던 점은 무엇인가?

병원에서 간호사 경력이 있던 선생님들과 대화를 나누며 병원과 국민건강보험공단 에 대해 현장의 내용을 직접 들을수 있어서 좋았다. 또한 국민건강 보험공단의 채용에 대해 설명해 주셔서 간호사 면허로 지원 가능한 업무에 대해 설명해 주셔서 앞으로의 취업등에 직접적인 도움을 받을수 있게 된것 같다.

P2

1. 오늘 실습경험의 의미 있는 점은 무엇인가?

치매 보건소에서 실습을 하게 되었는데 직접 눈으로 보진 않고, 옆에 앉아서 듣게 되었는데 어르신들께서 치매 조기검진을 받으러 오시는 것을 듣게 되었다. 치매검진은 대부분 병원 같은 의료기관에서 자세히 이루어 지는 줄 알았는데 이렇게 보건소에서 조기검진을 할수 있는 곳이 있다는 것을 알게되고, 검진 받는걸 보게되어 의미있었다

보건소 인식

2. 오늘 실습을 하는 동안 나의 생각과 느낌은 어떠했는가?

갈수록 고령화 사회가 되면서 어르신들이 많아지는데 고령이신 분들이 가장 두려워 하는 병이 치매라고 한다. 이런 치매를 보건소에서 언제든지 60세 이상 이시면 검사를 받아서 조기검진을 할수 있다는 게 정말 좋은것 같다는 생각이 들었다. 치매도 조기에 치료를 시작하면 충분히 늦추고, 관리가 가능하다고 해서 많은 어르신들이 이런 보건소 프로그램을 알고, 이용해서 모두가 건강하게 생활하면 좋은것 같다고 생각되었다.

3. 오늘 실습을 통해 새롭게 알게 된 것은?

치매 안심센터 에서 60세 이상 관내 어르신이면 누구든지 인지선별검사(CIST)를 받을수 있는데, 학력, 연세에 따른 기준 점수에 의하여 정상, 인지저하로 나눠진다. 인지저하 판정을 받으면 정밀검사 안내 및 연계를 해주게 된다. 정밀검사 (SNSB)는 기억력, 지남력, 주의력, 시공간 기능, 집행기능, 언어기능 등의 영역 검사를 문답식으로 시행하는데 결과로 경도인지 장애가 나오면 1년마다 재검사를 시행하고, 치매 의심되면 감별검사를 연계해준다 감별검사는 병원에서 실시하는 치매 진단을 위해 MRI 또는 혈액검사를 한다고 한다.

4. 오늘 실습을 통해 새롭게 배운 것들이 향후 간호실무에 어떻게 적용될 수 있겠는가?

나중에 60세 이상의 어르신 인 대상자를 만났을 때 치매에 대해 두려워하고, 의심이 조금 되면 보건소에 치매 안심센터 에 이런 프로그램이 있다는 것을 안내해 줄수 있을 것 같다. 그렇게 해서 많은 대상자들이 조기검진을 통해 치매를 예방할수 있도록 하고, 조기진단이 되면 질병을 늦추거나 치료해서 많은 분들이 건강하게 살수 있도록 하면 좋을것 같다.

5. 오늘 실습에서 좋았던 점과 아쉬웠던 점은 무엇인가?

치매 조기검진을 받는 모습을 직접 보진 못하고, 옆에서 듣기만 해서 조금 아쉬웠다. 대상자들이 어떤 질문들을 통해 점수를 받는지 세세하게 보면 더 기억에 남을것 같은데 그러지 못했던 점이 아쉬웠다.

1. 오늘 실습경험의 의미 있는 점은 무엇인가?

오늘은 방문 건강관리실에서 실습을 하게 되었다. 방문건강관리 사업이란 보건소에 소속된 방문간호사가 각 가정을 방문하여 가족과 건강 문제를 가진 가구원을 발견하여 질병예방 및 관리, 건강증진을 위하여 건강 서비스를 제공하는 사업 이라고 한다. 방문건강관리의 목표는 건강행태 개선, 건강문제 관리 여서 이러한 목표에 맞춰 방문건강 관리를 한다고 알게 되었다.

2. 오늘 실습을 하는 동안 나의 생각과 느낌은 어떠했는가?

오늘 오전에 교수님과 함께 집담회를 해서 방문건강관리실에서의 실습이 늦게 시작 되었다. 점심 이후에 와서 이미 오늘은 방문건강을 하러 나간 상태에서 아쉽게도 현장에 나가지 못하였다. 대신 집담회를 하면서 지역사회 간호에 대해 조금 더 자세히 알수 있었고, 지역사회 간호의 PI이나 진단들을 어떠한 방식으로 내려야 하는지 배울수 있게 되어 좋았다.

3. 오늘 실습을 통해 새롭게 알게 된 것은?

방문간호의 대상은 기초수급자, 독거노인, 차상위 계층, 장애인, 독거노인, 타기관에서 의뢰한 건강 문제가 있는 대상자, 재가암 환자 관리, 재가 장애인, 집단서비스로 경로당 등이 있다고 한다. 그동안 알고 있던 대상보다 훨씬 더 다양한 대상들 방문간호 한다는 사실을 알게 되었으며 방문관리의 목표는 건강 행태 개선으로 건강상태 인식, 건강 생활 실천 유도, 건강 지식 향상이 있으며, 건강문제 관리로는 건강문제 정기적 스크리닝, 증상 조절, 치료 순응 향상이 있다.

4. 오늘 실습을 통해 새롭게 배운 것들이 향후 간호실무에 어떻게 적용될 수 있겠는가?

나중에 병원의 간호사가 되어서 의료기관에서 치료를 계속 받지 못하는 대상자가 있다면 보건소의 이러한 방문건강 관리 사업에 대해 소개를 해주어서 많은 취약 계층의 대상자들이 치료를 지속적으로 받을수 있도록 적용하면 좋을것 같다. 또한 나중에 보건소에서 일을 하게 되면 이번에 배운 내용을 바탕으로 사업을 적용해도 좋을것 같다.

5. 오늘 실습에서 좋았던 점과 아쉬웠던 점은 무엇인가?

집담회를 오전에 하고, 오후에 늦게 방문건강관리실에서 실습이 시작되어서 방문건강에 이미 선생님들이 나가신 후라 같이 현장에 따라가지 못하게 된 점이 아쉬웠다. 오늘 하루만 방문건강관리실에서 실습하는게 아니고, 다음날에도 할 예정이라 그때 적극 참여 해야 겠다는 생각이 들었다.

P3

1. 오늘 실습경험의 의미 있는 점은 무엇인가?

방문건강관리실에서 실습을 하면서 방문 간호사 선생님과 가정방문 하면서 어르신들의 건강을 관리한 점이 의미 있었다. / 가정방문해서 어떤 건강 관리를 접하는지 궁금했는데 방문간호 선생님과 함께 이동하면서 현장을 보고, 겪게 되어 좋았다.

#방문간호 전문적?

2. 오늘 실습을 하는 동안 나의 생각과 느낌은 어떠했는가?

소외계층과 취약 계층이 우리 주변에 너무 많다는 사실이 몸소 느껴졌다. 독거노인도 정말 많고, 어르신들이 자가 관리도 많이 안되어서 아무 약이나 그냥 막 드시는 것을 직접 보게 되어 도움이 필요한 곳이 정말 많음을 알게 되었다. 앞으로는 더욱더 고령사회가 될거라고 하는데 정말 아끼 사회운제 임을 다시한번 느끼게 되었고, 나부터 조금씩 우리 주변을 돌봐야 겠다는 생각이 들었다.

#인구특성 고령화

3-1

3. 오늘 실습을 통해 새롭게 알게 된 것은?

방문간호사 선생님들께서 담당 하시는 구역이 다 다르고, 선생님들이 맡으신 구역에 기초수급자, 독거노인, 차상위 계층, 장애인, 타기관에서 의뢰한 건강 문제가 있는 대상자, 재가암 환자 관리, 재가 장애인, 집단서비스(경로당) 등의 대상자들의 건강을 관리한다는 것을 알게 되었다 // 가정방문의 정해진 횟수는 없고, 좀더 건강관리가 필요한 분들께 전화를 한뒤 방문하여 혈압, 혈당을 재고 어르신들의 전반적인 건강에 대해 여쭙고 말동무도 되어 드리는 일을 하셨다. / 병원처럼 진료, 직접적인 간호를 하지는 않고, 질병을 예방하거나 건강 생활을 실천 하도록 교육하는 일을 한다는 것을 알수 있었다.

#방문간호

4. 오늘 실습을 통해 새롭게 배운 것들이 향후 간호실무에 어떻게 적용될 수 있겠는가?

우리 주변의 소외계층이나 취약계층인 다양한 대상자들이 의료 사각지대에 있어서 제대로된 건강한 생활이 이루어 지지 않고, 교육이 이루어 지지 않고 있다는 사실을 알게 된다면 그분들에게 보건소에 있는 방문건강서비스 사업에 대해 안내해 드릴수 있을 것 같다. 또한 내가 나중에 방문건강 간호에 관심이 생겨 취약한 대상자를 돕고 싶다면 이러한 사업에 대해 관심갖고 많은 분들에게 제공하려고 노력할수 있을 것 같다.

5. 오늘 실습에서 좋았던 점과 아쉬웠던 점은 무엇인가?

방문간호 선생님과 함께 가정방문 하며 현장을 돌아다니며 직접 현장을 볼수 있고, 혈압 · 혈당도 재드리며 방문간호사의 역할이 어떤것인지 조금이라도 알수 있게 되어서 좋았다. / 어르신들께 건강하게 지낼수 있도록 생활에 대한 교육이나 음식 등에 대해 자세히 설명해 드리며 교육하는 모습을 볼수 있어서 좋았다.

★ 방문간호 #전문적

P3

1. 오늘 실습경험의 의미 있는 점은 무엇인가?

오늘은 이동진료팀에서 원래 실습하는 날인데 농번기로 인하여 경로당의 노인분들이 바쁘시다고 하여 출장에 나갈 수 없었다. 그래서 수유실에서 수유실에 배치 되어 있는 다양한 모자건강에 대한 내용에 대해 공부 하게 되었다. 모유수유 에 대한내용과 태아, 0-3세의 육아 등에 대해 알수 있었다.

2. 오늘 실습을 하는 동안 나의 생각과 느낌은 어떠했는가?

이동진료팀에서는 어떤일을 하는지 궁금했는데 그것을 겪어보지 못해서 아쉬웠다. 대신 수유실에서 이번에 실습하지 않는 모자보건에 대해 공부해서 또 다른점으로는 좋았다. / 이동진료나 방문간호 등은 병원과 다르게 우리가 환자나 대상자를 찾아가는 시스템이어서 항상 변수가 생긴다는것을 감안해야 된다는 사실도 알게 되었다. 보건소와 병원의 차이에 대해 알수 있던 날이었다.

보건소인식

3. 오늘 실습을 통해 새롭게 알게 된 것은?

모유수유에 대해 공부하였는데 모유수유는 신생아 및 영유아의 건강에 중요한 공급원이며 세계 보건기구 에서도 신생아 초기의 완전모유수유를 핵심 지표로 삼고 있다고 한다. 모유수유는 아기 뿐만 아니라 어머니의 건강에도 다양한 이점이 있으며, 사회경제적으로도 이득이 많다고 한다. 대부분의 엄마들과 아기들은 모유수유가 가능하며 모유수유 교육은 엄마와 아기에게도 도움이 될수 있다. 모유수유의 교육 대상자는 산모뿐만 아니라 보건소 모유수유 담당자, 산후조리원 모유수유 담당자 등도 대상자라고 한다.

4. 오늘 실습을 통해 새롭게 배운 것들이 향후 간호실무에 어떻게 적용될 수 있겠는가?

나중에 내가 분만실이나 신생아실등 산모와 관련된 대상자를 만나서 간호하게 된다면 오늘 공부한 모유수유 내용을 조금이라도 안내 해 드릴수 있을 것 같다. 무작정 모유가 좋아서 모유수유를 해야 한다는 것보다 구체적으로 왜 모유수유가 좋은지 안내해 드리면 좋을것 같다. 모유수유에 대한 지식이 없는 산모들에게 보건소에 있는 다양한 프로그램에 대해 안내를 할수도 있을 것 같다.

효과적 교육.

5. 오늘 실습에서 좋았던 점과 아쉬웠던 점은 무엇인가?

오늘 이동진료팀이 하는 일을 직접 보지 못해서 아쉬웠다. 보건소에서 무슨일을 하는지 다 배우고 갈 수는 없지만 배정 받은 실습지 에서는 최선을 다해 배우고 가고 싶지만 아쉽게도 오늘 출장이 없어서 수유실 안에만 있게 되었지만 수유실에서 또 다른 내용들에 대해 공부하게 된 점은 좋았다.

P3

7-2 외국인

1. 오늘 실습경험의 의미 있는 점은 무엇인가?

접수실에서 실습을 하면서 보건소에 있는 다양한 서비스에 대해 알수 있었던 점이 좋았다. 매일 다른곳을 실습하면서 그곳에 대해 자세히 아는것도 좋지만 이렇게 접수실에서 실습하면서 민원 안내를 해드리며 다양한 서비스가 있다는 것을 알고, 보건소 내부의 전반적인 위치를 파악한 점도 좋았던것 같다.

2. 오늘 실습을 하는 동안 나의 생각과 느낌은 어떠했는가?

접수실에 외국인이 난민검사를 하고 싶다고 왔는데 접수실에 영어를 할수 있는 선생님이 한분도 안계셔서 꽤 오랜시간 서로 의사소통이 안되서 힘들어 하는 모습을 보게 되었다. 외국인도 자신이 받아야 하는 검사에 대해서도 전혀 설명하지 못하고, 접수실 선생님들도 설명을 못해서 기본적인 영어를 배워야 겠다는 생각이 들었다. 점점 더 글로벌한 사회가 되어가는데 우리나라에 온 외국인들의 소통이 되지 않아서 의료서비스를 제대로 받지 못하면 안될것 같기에 나도 기본적인 영어를 공부해야 겠다는 생각이 들었다.

3. 오늘 실습을 통해 새롭게 알게 된 것은?

접수실에서 실습하면서 보건소에 다양한 프로그램과 서비스를 이용하기 위해 꽤 많은 사람들이 방문한다는 사실을 알게 되었다. 대부분은 보건증을 만들기 위해 많이 방문하고, 예방접종을 위해 방문하는 사람들도 많다는것을 알게 되었다. 보건소에서 65세 어르신들은 폐렴구균 예방접종을 하러 오셨다. 아토피 대상자 들은 로션을 지원도 해주고, 금연 프로그램을 받기 위해 금연 상담실을 이용하는 분들도 많이 오셨다. 그동안 나는 보건소보다 병원을 많이 이용해서 보건소에 이렇게 다양한 프로그램을 이용하러 많은 사람들이 온다는 사실을 실습 하면서 처음 알게 되었다.

4. 오늘 실습을 통해 새롭게 배운 것들이 향후 간호실무에 어떻게 적용될 수 있겠는가?

보건소에 있는 다양한 활동을 안내해 드릴수 있을 것 같다. 사전연명, 임산부, 의학처치, 영유아 건강검진 지원, 전염병, 정신건강 상담, 아토피 지원, 치매상담, 결핵상담, 자동차 적성검사, 당뇨, 콜레스테롤, B형 간염, 간기능, 빈혈, 혈액형 검사, 건강클리닉 운영, 금연프로그램, 영양플러스 사업, 예방접종, 산후도우미 신청, 유축기 대여, 신고검사, 엽산제, 철분제 지원, 코로나 관련 등의 보건소 활동에 대해 알게 되었으니 이에 적절하게 대상자에게 안내 하고, 설명해 줄수 있을 것 같다.

5. 오늘 실습에서 좋았던 점과 아쉬웠던 점은 무엇인가?

접수실 입구에서 다양한 민원인들을 만나며 보건소 내부에 대해 안내해 드려 재미있고, 좋았다. 민원인 분들이 헤매시고 어디로 가야 하는지 잘 모르실때 내가 아는 부분에 대해 안내 해 드려서 알게되고, 가시는 모습이 뿌듯했다. 그리고 외국인 대상자가 왔을때 의사소통이 되지 않은 점은 매우 아쉬웠다. 앞으로 외국인이 많은데 기본적인 영어를 배워서 필요한 영어는 알아둬야 겠다는 생각이 들었다.

1. 오늘 실습경험의 의미 있는 점은 무엇인가?

예방접종실에서 실습하면서 제대혈에 대해 자세히 알게 된 점이 의미 있었다.
제대혈이란 출산시 단 한번만 얻을수 있는 탯줄과 태반에 존재하는 혈액을 말하며
기증된 제대혈은 제대혈 속에 혈액을 만드는 줄기세포인 조혈모세포가 들어있는데 기증된 제대혈과
조건이 일치하는 환자에게 이식하여 백혈병, 재생불량성 빈혈등 난치성 혈액질환을 치료하는데 사용한다는
사실을 배우게 되었다.

2. 오늘 실습을 하는 동안 나의 생각과 느낌은 어떠했는가?

65세이상 어르신들께서 보건소에서 무료로 접종해주는 폐렴구균에 매우 관심이
많으시다는 사실을 알게 되었다. 다른 진료를 보러 오시다가 "65세 이상 폐렴구균 무료접종"
내용을 보시고 예방접종실에 오시는 경우가 많았는데 어르신들은 이미 맞으시거나
다른 종류를 맞으시거나 의 등등 이유로 다음에 맞아야 하는 경우가 많았는데 이런 경우를
매우 못갈려 하심을 보게 되었다. 어르신들께 최대한 이해가 가도록 예방접종에 대해
설명해 드리지만 지금 보다 더 쉽고, 어른들이 알아 들으실수 있는 내용 설명이 필요함을 깨닫게 되었다.

3. 오늘 실습을 통해 새롭게 알게 된 것은?

HPV 예방접종 전·후 주의사항에 대해 자세히 알게 되었다. 건강 상태가 좋은날,
접종기관을 방문하고 미성년자는 안전한 예방 접종을 위해 보호자와 함께 방문 하도록 한다
접종전 예진표를 꼼꼼히 작성하고, 접종시 등받이가 있는 의자에 앉거나 누워서
접종받고, 예방접종 후 20~30 분간 의료기관에 앉아 있거나 누워서 경과를 관찰하도록 한다.
접종후 심한 긴장으로 인해 일시적으로 정신을 잃고 넘어질수 있지만, 이는 다른 예방접종후에도
발생할수 있는 상황으로 충분히 예방 가능하다고 한다. 또한 국가에서 1995년부터 예방접종후 이상반응이
발생하면 예방접종과의 관련성을 심의하여 진료비등을 보상하는 「예방접종 피해 국가 보상 제도」를 운영하고 있다고 한다.

4. 오늘 실습을 통해 새롭게 배운 것들이 향후 간호실무에 어떻게 적용될 수 있겠는가?

병원에서 여러가지 예방접종과 관련된 간호 업무를 할때 오늘 새롭게 알게된
예방접종 관련 주의 사항들을 참고하여 대상자에게 예방 접종도 해주고,
주의사항에 대해 교육해 줄수 있을 것 같다. 주의사항 들을 정확히 알려주어
대상자들에게 예방접종으로 인한 문제가 발생하지 않도록 예방하고 혹시나 예방접종으로 인한
이상반응이 나타나게 된다면 「예방접종 피해 국가보상제도」를 알려주어 국가보상을
받을수 있도록 해드리면 좋을것 같다.

5. 오늘 실습에서 좋았던 점과 아쉬웠던 점은 무엇인가?

예방 접종실에 있는 여러 냉장고 안에 백신을 볼수 있어서 좋았다.
냉장고 마다 온도가 다 다르고 그 온도에 맞는 백신별로 나눠서 보관하고
있다는 점도 신기했다. 또한 민간 의료기관에서 코로나 백신을 보건소에서
가져가고, 반납하고 있다는 사실도 처음 알게 되었다. 보건소에서 많은 것을
민간의료기관에 관여 하고 있다는 점을 알게 되어 좋았던 실습이었다.

P2

1. 오늘 실습경험의 의미 있는 점은 무엇인가?

구강보건센터에서 실습하게 되었는데 구강보건센터에서는 무슨일들을 하는지 알게 되어 의미 있었다. 노인의치 보철사업, 학교구강 보건 사업, 어린이 충치 예방 사업, 노인불소도포, 스켈링 사업, 사회복지시설 구강건강관리 사업, 장애인 및 취약계층 우선진료, 불소용액 양치사업, 구강 보건교육, 양치체험교실운영, 미취학 어린이 구강건강관리 사업 등 다양한 구강관련 사업을 하고 있음을 알게 된 점이 의미 있었다.

2. 오늘 실습을 하는 동안 나의 생각과 느낌은 어떠했는가?

어린이집에서 어린이들이 (5-7세) 구강 보건센터에 와서 충치 예방을 위한 교육을 듣는 것을 보게 되었다. 어린이들의 수준에 맞도록 (콩순이)라는 만화를 통해 충치예방 영상을 보는것을 관찰하게 되었다. 보건소에서 이렇게 연령에 맞는 다양한 보건교육 프로그램을 운영하는 것을 보니 내가 그동안 보건소에서 하는 다양한 사업, 교육을 많이 알지 못했다는 사실을 알게 되었다. 지역사회를 위한 더 많은 사업과 교육이 어떤것들이 있는지 찾아봐야 겠다는 생각이 들게 되었다.

3. 오늘 실습을 통해 새롭게 알게 된 것은?

우리나라 식품의약품 안전처에서는 불소가 들어있는 치약에 불소정보를 제시하고 있다고 한다. 주요성분에는 일불소인산나트륨, 플루오르화나트륨, 플루오르화석, 플루오르화아민 297 등 4가지 중 한가지 성분이 있는지 확인해야 한다고 한다. 또한 내가 쓰는 치약의 정보는 식품의약품 안전처 홈페이지 에서 의약품 제품 정보에서 볼수있다고 한다. 치석제거 (스케일링) 이란 잇몸병의 가장 큰 원인인 치아 표면에 붙어있는 치석과 세균을 제거하고, 치아표면을 매끄럽게 하여 세균 부착을 방지하기 위한 목적의 예방 처치 이다. 스케일링은 만 19세 이상 성인 부터 대상자 라고 한다.

4. 오늘 실습을 통해 새롭게 배운 것들이 향후 간호실무에 어떻게 적용될 수 있겠는가?

구강관련 대상자에게 구강 건강에 대해 설명해주고, 구강검진에 대해 교육해줄수 있을것 같다. 일반 사람이라면, 관심이 크게 없는 사람 이라면 잘 알지 못하는 불소의 성분에 대해 교육하여 그 성분이 있는 치약을 쓸수 있도록 대상자에게 알려주고 치석제거 (스케일링) 이란 무엇인지 설명하여 대상자들이 구강 건강을 지키도록 건강교육에 적용하면 좋을것 같다. 구강 관련된 건강 홈페이지를 소개 해주는 방법도 좋을것 같다.

5. 오늘 실습에서 좋았던 점과 아쉬웠던 점은 무엇인가?

그동안 일상생활에서 불소양치를 제대로 하고 있지 않았다는 사실을 알게 되었다. 구강보건센터에서 불소양치용액을 직접 만들고, 용법에 대해 설명법을 알게 된 점이 좋았다. 불소양치 용액은 하루에 한번, 칫솔질을 깨끗이 하고 10cc 정도의 불소양치용액을 작은 양치컵에 따라 1분가량 입안에 머금은 후 뱉는다. 양치 후에는 30분 가량 음식이나 물로 입안을 헹구지 않는다고 한다. 이러한 용법을 제대로 배워 일상에 적용할수 있게 되어 좋았다

1. 오늘 실습경험의 의미 있는 점은 무엇인가?

실습 시작 전 전체적인 오리엔테이션을 통해 보건소, 치매안심센터, 건강보험공단, 감염병관리 등에 대해 교육을 받았고 어떤 기관이고, 어떤 일 등을 하는지에 대해 알게 되었습니다.
오리엔테이션 후 '건강증진실'에서 실습하며 건강증진실의 업무나 사업 등에 대해 알게 되었습니다.
또한 건강증진실에서 시민분들이 오셔서 건강 프로그램을 상담하시고 영양 선생님께서 생활습관에 대해 교육해주시는 것을 관찰할 수 있었습니다.

2. 오늘 실습을 하는 동안 나의 생각과 느낌은 어떠했는가?

지역사회 간호학의 이론수업을 들으면서 보건소에서는 어떤 업무를 하는지 궁금하고 경험해보고 싶었는데 일반적으로 생각하는 것과 달리 많은 사업과 프로그램 등이 구성되어 있는 것을 알게 되었습니다.
보건소에서 지역사회 시민들을 위해 건강, 영양, 출산, 치매 관리 등 다양한 사업을 진행 중인데 더 많은 시민분들이 알고 참여하지 못하는 것 같아서 조금 더 많은 홍보를 통해 편안한 시민 모두 사업에 참여하여 삶의 질이 향상되었으면 좋겠다는 생각을 하게 되었습니다.

3. 오늘 실습을 통해 새롭게 알게 된 것은?

감염병 교육을 통해 털진드기로 인해 'SFTS'에 감염될 수 있고 조기에 검진하지 못하고 적절한 치료를 받지 못하면 사망에 이를 수도 있다는 것을 새롭게 알 수 있었습니다.
건강증진실에서는 건강증진실 이용 여부, 알게된 경로, 1일 30분 이상 운동을 하는지, 만약 한다면 어떤 종목을 하는지, 일주일 동안의 운동 실천 일수, 운동을 하지 않는 이유, 개선사항 등의 항목을 묻는 설문지를 작성한 후 인바디나 혈압, 혈당 등을 측정하여 이를 바탕으로 대상자의 건강 상태를 파악하며 식습관과 생활습관, 운동 등에 대해 교육하며 '채움건강'이라는 이름을 통해 보건소에서 대상자를 주기적으로 관리하는 것을 새로 알게 되었습니다.

4. 오늘 실습을 통해 새롭게 배운 것들이 향후 간호실무에 어떻게 적용될 수 있겠는가?

보건소의 건강증진사업에 참여하는 대상자들에게 각각 건강상태에 적절한 식습관과 운동습관을 알고 교육해주는 데에 적용할 수 있을 것같고, 주기적으로 관리하기 위해 중간검진을 시행하는 것과 이때 참여 전의 상태와 비교하여 개선하고 교정해야 할 사항들을 교육하는 데에 적용할 수 있을 것 같습니다. 또, 보건소에서 제공하는 전자우기를 통해 운동과 식사 일기를 기록하고 자신의 건강 상담과 커뮤니티를 통해 다른 사람들과 정보도 공유할 수 있는 점들을 알고 적용하여 지역사회 시민들의 삶의 질을 개선해 나가는 데 도울 수 있을 것 같습니다.

5. 오늘 실습에서 좋았던 점과 아쉬웠던 점은 무엇인가?

실습을 시작하기 전에 전체적인 오리엔테이션을 통해 전반적인 보건소의 체계나 사업 등에 대해 알 수 있어 좋았고, 매일 다른 과로 실습을 하게 되는데 오늘은 건강증진실에서 어떠한 업무를 하는지 충분히 배울 수 있었던 시간이어서 좋았습니다. 또, 일주일에 세번 헬스 강사분께서 오셔서 진행하는 헬스 수업에도 직접 참여하게 해주셔서 시민분들이 제공받는 프로그램을 같이 해볼 수 있어 좋았습니다.

1. 오늘 실습경험의 의미 있는 점은 무엇인가?

오늘은 보건소 내에 위치한 과에 배정받은 것이 아니라 건강보험공단으로 따로 실습을 나오게 되었는데 실습이 아니면 경험해보지 못할 곳이기 때문에 의미 있는 경험이었습니다.
또한 직원분들께서 일하시는 모습을 지켜보는 것만으로 배우는 것이 아니라 직접 저희에게 자궁경부암 검진 전화 안내 업무를 시켜주셔서 새로운 경험을 할 수 있는 기회였습니다.

2. 오늘 실습을 하는 동안 나의 생각과 느낌은 어떠했는가?

평소 저에게도 건강보험공단에서 건강검진 안내에 대한 문자나 전화 등의 연락이 오곤 했었는데 검진을 받을 때까지 연락이 오는 것에 조금은 귀찮기도 했는데 직접 전화로 안내를 하려고 보니 별거 아닌 일 같지만 정말 많은 사람에게 전화를 해서 검진에 대해 안내를 하고 다른 질문들에도 대응하다보니 나라에서 제공해주는 이러한 서비스가 있다는 것에 감사하며 안내전화에도 친절하게 답해드려야 겠다고 느꼈습니다.

3. 오늘 실습을 통해 새롭게 알게 된 것은?

출생연도에 따라 짝·홀수를 기준으로 2년마다 20세 이상의 여성에게 자궁경부암 검진을 시행하는 것을 정확하게 알게 되었고, 만약 임신 중이거나 자궁적출술 같은 자궁관련 수술을 받았거나, 성 경험이 없거나, 암치료 중 또는 치료 중인 경우 검진 제외 신청을 하여 해당연도에 더 이상 연락이 가지 않도록 할 수 있다는 것을 새롭게 알게 되었습니다.

4. 오늘 실습을 통해 새롭게 배운 것들이 향후 간호실무에 어떻게 적용될 수 있겠는가?

만약 건강보험공단에서 일하게 된다면 오늘 실습하는동안 배운 내용들을 바탕으로 기본적인 검진 안내 전화는 어떻게 처리해야 하는지를 알고 적용할 수 있을 것 같습니다.
또 다른 직원분들께서 처리하는 교통사고나 산재 등에 대한 일을 어떻게 처리하는지 조금이나마 듣고 익혀 실무에 적용할 수 있을 것 같습니다.

5. 오늘 실습에서 좋았던 점과 아쉬웠던 점은 무엇인가?

실습학생이라고 해서 마냥 직원분들이 일하시는 것을 지켜보기만 하는 것이 아니라 직접 검진 전화 안내 업무를 할 수 있도록 해주셔서 좋았습니다.
또 처음 해보는 일이니 전체적으로 어떻게 해야하는지, 주의사항이 무엇인지 등 자세하게 설명을 해주셔서 훨씬 수월하게 해낼 수 있었던 것 같습니다.

1. 오늘 실습경험의 의미 있는 점은 무엇인가?

오늘도 어제에 이어서 건강보험공단에서 실습을 하게 되었는데 실습학생을 담당해주시는 선생님께서 건강보험공단에 취업하는데에 필요한 자격증과 조건, 그리고 최근 채용공고 자료를 주셔서 설명해주셔서 건강보험공단에 대해 더 자세히 알 수 있게 되었습니다.

2. 오늘 실습을 하는 동안 나의 생각과 느낌은 어떠했는가?

그동안 여러 병원에서 실습을 해봤지만 건강보험공단에서의 실습은 병원실습과는 전혀 다른 경험을 할 수 있어서 좋았고, 꼭 병원으로의 취업만을 생각하지 않던 상황에 건강보험공단에 직접 근무하고 계신 선생님들의 이야기와 조언, 장단점 등을 들을 수 있어서 앞으로의 진로에 대해 한번 더 생각하는 계기가 되었습니다.

3. 오늘 실습을 통해 새롭게 알게 된 것은?

오늘은 노인장기요양보험에 대한 내용이 담긴 책자를 찾아보았는데 장기요양등급 판정을 받은경우 복지용구인 이동변기, 성인용보행기, 지팡이, 요실금 팬티, 욕창예방 방석 등을 구입하는 급여를 지원받을 수 있고, 수동휠체어나 이동욕조, 배회감지기, 전동·수동 침대, 욕창 예방 매트리스 등을 대여 받아 이용이 가능하다는 것을 알게 되었습니다. 또 장기요양 인정 유효기간은 최소 1년 6개월에서 최대 4년 6개월까지 (갱신신청의 경우) 판정되기 때문에 급여를 계속하여 이용하려면 유효기간이 끝나기 90일 전부터 30일 전까지 갱신신청을 해야 가능하다는 것을 새로 알게 되었습니다.

4. 오늘 실습을 통해 새롭게 배운 것들이 향후 간호실무에 어떻게 적용될 수 있겠는가?

건강보험공단의 건강직과 요양직 각 직렬에서 어떠한 업무를 하는지 알고 건강직의 경우 국민건강보험법, 요양직의 경우 노인장기요양보험법에 대해 더 공부하고 익혀 실무에 적용할 수 있을 것 같습니다. 또한 담당선생님께서 제공해주신 자료들을 통해 알게된 내용들을 바탕으로 건강보험공단에 대해 알고 취업을 준비하는 데에 적용할 수 있을 것 같습니다.

5. 오늘 실습에서 좋았던 점과 아쉬웠던 점은 무엇인가?

담당선생님과 다른 선생님들께서도 너무 친절하게 대해주시고 실습 중인 학생이라 아무리 편하게 있하라고 하셔도 눈치보게 되는데 직접 같이 휴게실에 데려가주셔서 진로나 병원에서 일할 때의 힘들었던 점, 장점, 이곳에서의 업무는 어떤지 등 다양한 이야기를 해주시고 많은 조언을 해주셔서 좋았습니다.

1. 오늘 실습경험의 의미 있는 점은 무엇인가?

오늘은 보건소의 접수실에서 실습을 하게 되어서 민원인분들께 안내를 해드리는 일을 해볼 수 있었습니다. 일반적으로 보건소에 방문하시는 대부분의 사람들이 제일 먼저, 그리고 많이 이용하시는 접수실이다보니 보건소에서 어떠한 업무를 하는지 전체적으로 알 수 있게 되었고, 민원인분들이 자신이 어디로 가야하는지 질문하시는 것에 대해 안내를 해드릴 수 있다는 점이 재미있는 경험이었습니다.

2. 오늘 실습을 하는 동안 나의 생각과 느낌은 어떠했는가?

지역사회 간호학 실습을 하면서 병원에 비하면 보건소는 정말 편해보이기만 했는데 오늘 하루동안 접수실에서 직접 경험해보니 계속해서 민원인을 상대하는 업무를 하는 것 또한 마냥 쉽지만은 않은 일이라는 것을 느끼게 되었습니다.
또 민원인분들이 보건소에 들어와 자신이 무엇을 해야하고, 어디로 가야할지 모를 때 가장 쉽게 접근할 수 있는 곳이 접수실이기 때문에 보건소의 전체 업무와 위치를 알고 전부 안내를 해드리는 접수실 선생님들이 대단하다고 생각되었습니다.

3. 오늘 실습을 통해 새롭게 알게 된 것은?

보건소에서 사전연명중단 신청과 운전면허 적성검사, 병·의원 관련신고, 한방진료, 물리치료, 치매상담, 결핵상담, 아토피 로션 지급, 산후도우미 신청, 유축기 대여, 산전검사, 그리고 암, 난임, 희귀난치, 영유아 검진에 대해 지원해주는 업무 등을 하고 있다는 것을 새롭게 알게 되었습니다.
또 빗물을 먹는 물로 바꾸기 위해 소독하는 급수약도 제공해드리는 것도 알게 되었습니다.
그리고 보건증을 발급받을 때에는 3.000원의 비용이 발생하는데 장기기증 신청을 해놓은 장기기증 신청자는 이 비용을 감면받을 수 있다는 것을 새롭게 알게 되었습니다.

4. 오늘 실습을 통해 새롭게 배운 것들이 향후 간호실무에 어떻게 적용될 수 있겠는가?

보건소의 전체적인 담당 업무와 담당과를 알고 보건소에서 일하게 된다면 이를 적용하여 조금 더 수월하게 일하는 데에 적용할 수 있을 것 같고.
접수실에서 가장 많이 하는 업무인 보건증 발급의 방법과 절차에 대해 배운 것들을 바탕으로 간호실무에 적용할 수 있을 것 같습니다.

5. 오늘 실습에서 좋았던 점과 아쉬웠던 점은 무엇인가?

접수실에서 일하시는 선생님들의 옆에서 마냥 보는것만으로 배우는 것이 아니라 간단한 민원 안내 업무라도 저에게 직접 가르쳐주시고 해볼 수 있는 기회를 만들어주셔서 좋았습니다. 또 대부분의 민원인분들이 보건증 발급을 하러 와서 조금 다른 업무들 하시는 것도 보고 싶었지만 계속해서 보건증 발급에 대해 듣다보니 이것 하나만큼은 정확하게 배우게 된 것 같아 의미있는 경험이었습니다.

1. 오늘 실습경험의 의미 있는 점은 무엇인가?

오늘 실습부서는 방문건강관리실이었는데 오전에는 교수님과 컨퍼런스를 진행하느라 방문간호를 직접 다녀와보지는 못했습니다. 하지만 컨퍼런스에서 다른 학생들의 사례보고서 발표를 들으면서 제가 사례보고서를 작성할 때 미처 생각하지 못하고 놓쳤던 부분이나, 작성하는 방법 또는 추가할 내용 등을 발견할 수 있었고 교수님의 피드백을 들으면서 다시 한번 지역사회의 간호과정 방법에 대해 배울수 있었습니다.

2. 오늘 실습을 하는 동안 나의 생각과 느낌은 어떠했는가?

지역사회간호학 1 이론 수업의 과제로 지역사회 대상 사례보고서를 한번 작성해봤었지만 일반적으로 많이 해봤던 사람 대상도 아니고 간호진단을 내리는 기준도 NANDA가 아닌 오마하 간호진단을 사용해서 쓰려다보니 과제를 하면서 의문점도 많았고 어려운 점도 있었는데 오늘 컨퍼런스를 통해 다른 학생들은 어떤 식으로 작성했는지 보면서 작성 기준을 조금은 세울 수 있게 되었고, 교수님의 피드백을 통해 지역사회 사례보고서는 물론이고 예비 간호사로서 어떠한 마음가짐과 태도를 지녀야 할지 생각할 수 있어 의미있는 시간이었습니다.

3. 오늘 실습을 통해 새롭게 알게 된 것은?

새롭게 알게 된 것은 아니지만 지역사회를 대상으로 하든, 사람을 대상으로 하든 간호과정에서 가장 먼저 해야하고 중요한 일은 대상에 대해 파악하고 이를 분석하여 해결해야 할 간호문제가 무엇이 있는지 파악하는 것이 중요하다는 것을 다시 한번 배울수 있었습니다.
또 매번 실습을 할 때 실습도 하면서 시험도 보고 과제도 해야하는 상황에 처하기 때문에 사례보고서를 작성할때 단지 시간 내에 완성하기 바빴는데 간호사를 준비하는 마음을 가지고 이러한 과제가 주어졌을 때 많은 자료들을 찾아보고 공부하면서 미리 준비하는 것이 필요하다는 것을 배울 수 있었습니다.

4. 오늘 실습을 통해 새롭게 배운 것들이 향후 간호실무에 어떻게 적용될 수 있겠는가?

만약 지역사회간호사로 일하게 된다면 지역사회를 대상으로 할때 기본적으로 사정해야 하는 부분들과 더 자세하게 분석할 필요가 있는 부분들은 알고 이를 적용하여 한 지역사회가 가지고 있는 간호문제를 찾아내는 데에 적용할 수 있을 것 같습니다.
또 상황이 주어졌을 때 배우기 위해 최선을 다하는 자세로 매순간 임하면서 간호사가 되었을때 환자들, 다른 의료진들에게 인정받고 일을 잘 해낼 수 있도록 노력하는 태도를 가지는 것에 적용할 수 있을 것 같습니다.

5. 오늘 실습에서 좋았던 점과 아쉬웠던 점은 무엇인가?

혼자서 사례보고서를 쓰려고 할때에는 막막한 마음도 들었고 이렇게 하는게 맞는건지 싶은 생각도 하곤 했었는데 다른 학생들의 사례보고서를 보면서 배울 점이나 주의할 점 등을 알 수 있어서 좋았고, 교수님께서 사례보고서에 대한 피드백만 해주시는 것이 아니라 이를 바탕으로 간호사로서 일할 때의 적용할 점까지 많은 조언들을 해주셔서 좋았습니다.

1. 오늘 실습경험의 의미 있는 점은 무엇인가?

오늘은 방문건강관리센터에서 실습을 하게 되었는데 지난 화요일에 컨퍼런스를 하느라 선생님들과 같이 방문간호를 못나가서 아쉬웠는데 오늘은 오전부터 직접 방문간호를 나가서 경험해볼 수 있었습니다. 이론에서만 배우고 실제로는 이렇게 가까이에서 방문간호를 하시는 모습은 처음 보는거라 개인적으로 경험이 되고, 방문간호를 받으시는 분들의 기준과 어떤 처치 등을 해드리는지 보고 해볼 수 있어서 의미있었습니다.

2. 오늘 실습을 하는 동안 나의 생각과 느낌은 어떠했는가?

제가 사는 지역이 군산이라서 방문간호를 가게 된 집들을 한번씩 또는 여러번 많이 지나치기도 했던 곳들이었는데 그렇게 무심코 생각없이 지나치기만 했던 곳들에 방문간호가 필요하신 어르신들이 살고 계신다는 것을 깨닫게 되었습니다. 또 방문간호를 받으시는 분들 대부분이 연세가 많으시다보니 자신이 살면 얼마나 더 살겠냐며 건강에 크게 신경을 쓰려고 하지 않는 모습을 보면서 특히 고령인구가 많은 도시인만큼 노인분들을 위한 교육이나 운동, 건강 개선을 위한 활동같은 서비스가 더 많아지면 좋을 것 같다는 생각을 하게 되었습니다.

3. 오늘 실습을 통해 새롭게 알게 된 것은?

방문건강관리 사업이란 보건소에 소속된 방문간호사가 각 가정을 방문하여 가족과 건강문제를 가진 가구원을 발견하여 질병예방 및 관리, 건강증진을 위하여 건강서비스를 제공하는 것이라는 내용과 방문간호의 대상은 기초수급자, 독거노인, 차상위계층, 장애인, 타기관에서 의뢰한 건강문제가 있는 대상자, 재가암 환자관리, 재가 장애인, 집단서비스(경로당)를 대상으로 하고, 방문건강관리의 목표는 건강상태를 인식하고 건강생활을 실천하도록 유도, 건강지식을 향상시키는 건강행태 개선과 건강문제를 정기적으로 스크리닝하고 증상을 간호하며 자가 순응을 향상시키는 건강문제 관리가 있는 것을 알게 되었습니다.

4. 오늘 실습을 통해 새롭게 배운 것들이 향후 간호실무에 어떻게 적용될 수 있겠는가?

보건소에 소속된 방문간호사로 일하게 되었을 때 오늘 직접 경험해보고 배운 내용들을 바탕으로 방문간호를 제공받을 수 있는 대상자 선정과 방문간호 시 어떠한 처치들을 해드리는지 알고 적용할 수 있을 것 같고. 방문간호의 목표인 방문간호 대상의 건강상태를 미리 파악하고 건강생활을 실천할 수 있도록 건강지식에 대해 교육하고 유도하는데에 적용할 수 있을 것 같습니다. 또한 방문간호사가 아니더라도 방문간호가 필요하고 서비스를 제공받을 수 있는 대상에게 방문간호서비스를 연계해주는 데에 적용할 수 있을 것 같습니다.

5. 오늘 실습에서 좋았던 점과 아쉬웠던 점은 무엇인가?

보건소 안에서만 있는 것이 아니라 밖에 나가서 직접 방문간호 대상자들의 집에 방문하며 방문간호 선생님께서 어떠한 처치를 해드리고 대상자분들께 어떤 내용을 교육해드리는지 직접 경험해볼 수 있어서 좋았습니다. 또 직접 방문간호를 나가보니 저희 가까이에 있는 생각보다 많은 분들이 건강관리를 하는데에 도움이 필요하다는 것을 느낄 수 있어서 좋았습니다.

1. 오늘 실습경험의 의미 있는 점은 무엇인가?

☆ 농번기

오늘의 실습부서는 이동진료팀이었는데 담당 선생님께서 요즘은 농번기라 어르신들이 다 바쁘시다보니 환자가 없다고 하셔서 이동진료는 나가보지 못했습니다. 그래서 수유실에서 공부하라고 하셔서 수유실에 구비되어 있는 모유수유 교육 프로그램, 뇌졸중 후 운동 방법, 모바일 헬스케어 사업 안내서 와 같은 책자들을 읽으며 보건소 내 사업에 대한 내용에 대해 공부할 수 있었습니다.

2. 오늘 실습을 하는 동안 나의 생각과 느낌은 어떠했는가?

이동진료팀에서의 실습은 하루밖에 안하는데 하필 농번기 시기와 겹쳐서 이동진료를 경험해볼 수 없어서 아쉬웠지만 다른 부서에서 실습할 때 보지 못했던 보건소에서 시행하고 있는 여러 사업들에 대한 내용이 담긴 책자들을 볼 수 있어서 좋았습니다. 이러한 것들을 보면서 그동안 알지 못했던 보건소에서 시행중인 사업이 정말 많다는 것을 알게 되었고, 이런 서비스를 좀 더 많은 홍보를 통해 더 많은 시민들에게 서비스를 제공해드리면 좋겠다는 생각을 하게 되었습니다

3. 오늘 실습을 통해 새롭게 알게 된 것은?

다학제

모바일 헬스케어 사업을 운영하기 위해 보건소 내에 필수로 있어야 할 인력에는 코디네이터, 의사, 간호사, 영양사, 운동전문가가 있고 사업에 참가하기 위한 대상자 요건은 만 19세 이상 성인, 해당 보건소 관할지역 내 거주자, 현재 관련 질환을 진단받거나, 해당 질환의 치료를 위해 약물처방을 받지 않는 사람에 한해서만 참여할 수 있다는 것을 알게되었습니다.
또 중간 검진과 최종 검진을 통해 건강관리를 도울 수 있고, 모바일 앱을 통해 지속적으로 모니터링하며 영양, 운동 등에 대해 집중적으로 관리를 해준다는 것을 알게 되었습니다.

4. 오늘 실습을 통해 새롭게 배운 것들이 향후 간호실무에 어떻게 적용될 수 있겠는가?

보건소에서 시행하는 사업들에 대해 알고 지역사회 시민들에게 필요한 서비스가 무엇이 있는지 찾아보고 현재 진행중인 사업이 있다면 연계해주고 부족한 사업이 있다면 어떠한 사업을 통해 문제를 해결할 수 있는지 계획하는 데에 적용할 수 있을 것 같습니다.
또 보건소에서 시행하고 있는 사업 중 지역사회 시민들에게 필요하고 많은 도움이 되는 서비스임에도 불구하고 참여자가 많이 없는 경우 어떻게 홍보를 해서 더 많은 시민분들의 삶의 질을 향상시킬 수 있는지에 대한 계획을 세우는 데에 적용할 수 있을 것 같습니다.

5. 오늘 실습에서 좋았던 점과 아쉬웠던 점은 무엇인가?

이동진료팀은 하루밖에 나가지 않는데 하필 농번기라 어르신들이 바쁘셔서 이동진료를 직접 나가보지 못해서 아쉬웠지만 수유실에 구비되어 있는 책자들을 통해 보건소에서 진행중인 사업 또는 교육 프로그램 등이 무엇이 있는지 알 수 있어서 좋았습니다.
또 현재 불각 중인 모바일 헬스케어 사업에 대한 사업안내서가 있어 사업 목적, 운영방식, 예산, 성과관리 등에 대해 더 자세한 내용을 알게 되어 좋았습니다.

1. 오늘 실습경험의 의미 있는 점은 무엇인가?

오늘 실습하게 된 부서는 치매안심센터였는데 치매를 예방하기 위해 조기검진을 받으러 오시는 분들과 이미 치매진단을 받은 치매환자의 보호자분들이 오셔서 치매조기검진을 받으시는 것과 보호자분들께 상담을 해드리며 치매환자분이 제공받을 수 있는 여러 서비스를 소개해드리고 필요로 하는 서비스는 연계해드리는 것을 볼 수 있어서 의미있었습니다. 또 치매환자와 보호자. 가족이 지원받을 수 있는 서비스에 대한 것도 새롭게 알게되어 의미있는 경험이었습니다.

2. 오늘 실습을 하는 동안 나의 생각과 느낌은 어떠했는가?

실습을 하는동안 정말 많은 분들이 치매 조기검진 중 인지선별검사(CIST)를 받으러 오셨는데 제가 들을 때는 쉽게 따라할 수 있고, 해결할 수 있는 문제이지만 나이가 있으신 어르신들께는 어려운 질문이라는 것을 깨닫게 되었습니다. 또 답을 잘 못하시는 분들을 보며 이정도면 치매를 의심해보는 정도가 아닌가하는 생각이 들기도 했는데 선생님께서 말씀해주시는 결과로는 정상인 것을 들었을 때 나이가 들어가면서 정말 인지능력이 감소하는 것을 실감하게 되었고. 만약 제가 치매검진 대상이더라도 치매에 대한 인식때문에 쉽게 검진받으러 오지 못할 것 같은데 그래도 예방을 위해 꾸준히 조기 검진을 받으시는 어르신분들이 대단하시다고 생각하게 되었습니다.

3. 오늘 실습을 통해 새롭게 알게 된 것은?

치매의 조기검진 대상은 60세 이상 관내 어르신으로 종류로는 학력, 연세에 따른 기준점수에 의하여 '정상 / 인지저하'로 나뉘지며 인지저하의 경우 정밀검사를 안내하고 연계해주는 인지선별검사(CIST)와 인지선별검사 실시 결과 '인지저하'가 나오신 분들을 대상으로 실시하는 정밀검사, 병원(신경과)에서 실시하는 치매 진단을 위한 MRI 또는 혈액검사를 하는 감별검사가 있다는 것을 알게되었습니다. 이러한 검진을 통해 치매로 진단받게 된 치매환자와 보호자를 대상으로 치매치료비 지원과 거동이 불편하신 분에게 기저귀나 물티슈 등 조호물품 지원. 그리고 실종을 예방하기 위한 지문등록, 배회인식표배부, 배회감지기 배부 등의 서비스를 제공해드리는 것을 알게 되었고, 치매환자를 돌보는 가족을 대상으로 돌봄교육 및 스트레스 해소 프로그램을 진행하는것을 알게 되었습니다.

4. 오늘 실습을 통해 새롭게 배운 것들이 향후 간호실무에 어떻게 적용될 수 있겠는가?

60세 이상의 어르신께 치매 조기검진을 권장하여 치매를 예방하거나 조기에 신속한 치료를 받을 수 있도록 돕고, 치매인식 개선교육과 치매파트너 양성교육 등을 통해 치매에 대한 기본정보를 제공해드리고 인식개선을 위한 교육을 실시하는 데에 적용할 수 있을 것 같습니다.
또 치매환자로 진단받은 어르신에게는 치매 치료비와 약제비는 지원받고, 방문이나 전화 상담을 통해 약 관리나 필요서비스를 연계받고, 지문을 등록하고 배회인식표와 배회감지기를 배부받을 수 있는 서비스를 소개해드려 치매로 인해 실종이나 경제적인 어려움을 겪지 않도록 도와드리는데에 적용할 수 있을 것 같습니다.

5. 오늘 실습에서 좋았던 점과 아쉬웠던 점은 무엇인가?

치매를 진단하는 검진에는 어떤 검사들이 있는지 선생님께서 실습을 시작하기 전에 설명을 해주셔서 알게 되었는데 가장 많이 하는 인지선별검사(CIST)를 직접 하시는 것을 여러번 볼 수 있었습니다. 이를 통해 인지선별검사를 할 때 질문하는 항목에 시간과 장소에 대한 지남력을 확인하는 것과 선생님께서 말씀하시는 문장. 숫자를 똑같이 말해보는 것. 주어진 단어를 거꾸로 말하기. 1분동안 알고있는 과일과 채소 종류를 최대한 많이 말하기 등이 있는 것을 자세히 알 수 있게되어 좋았습니다.

P3

1. 오늘 실습경험의 의미 있는 점은 무엇인가?

오늘 실습하게 된 부서는 예방접종실이었는데 어릴 때나 병원 실습 전에 직접 예방접종을 하러 간 경험은 있지만 잠깐 주사만 맞고 바로 나오는 경우가 대부분이다보니 직접 접종하는 백신의 종류가 아니면 다른 종류에는 어떠한 것들이 있는지 잘 몰랐고 백신 보관중인 냉장고나 냉장고 속에 어떻게 백신들을 배치해 두는지 자세히 볼 수 있는 기회가 없었는데 하루동안 예방접종실에서 백신보관방법과 온도, 유의사항 백신의 종류, 각 백신마다 접종의 필요성 등을 알 수 있어 의미있었습니다.

2. 오늘 실습을 하는 동안 나의 생각과 느낌은 어떠했는가?

항상 예방접종을 하러 가면 접종 접수를 하고 주사 한대만 맞고나면 끝나기 때문에 예방접종실에서 직접 실습해보기 전까지는 다른 부서에 비해서 업무가 쉬울 거라고 생각했는데 막상 하루종일 예방접종실에 있으면서 경험해보니 같은 종류의 예방접종이더라도 매번 오시는 분들마다 같은 내용을 반복해서 설명해주시고, 접종 후에 주의해야 할 점들도 똑같은 내용을 매번 반복하시는 것이 지겹고 지칠수도 있는데 항상 친절하게 많은 내용을 설명해드리려고 하는 모습이 정말 대단하신 것 같다고 생각하게 된 경험이었습니다.

3. 오늘 실습을 통해 새롭게 알게 된 것은?

백신을 보관하는 장치의 온도는 일반적으로 냉장고는 2~8°C (평균 5°C), 냉동고는 -50 ~ -15°C 의 온도를 유지하여야 하고, 백신의 올바른 취급과 보관을 위해 온도계는 필수적으로 구비해야 하며 자동온도기록장치는 사용하기도 하는 것을 알게 되었습니다. 자동온도기록장치는 일정 간격마다 온도변화를 자동기록하고, 최고/최저 온도, 설정온도 범위 이탈시간 정보 및 경보 알람의 기능을 포함하고 있어 설정온도를 이탈하는 즉시 설정된 담당자에게 알람이 갈 수 있고 이에 백신 담당자는 바로 확인해야 한다는 것을 새롭게 알게되었습니다. 또 비슷해 보이거나 유사한 포장을 사용하거나, 소아 또는 성인용 전제분을 구분해야 하는 백신은 인접하게 보관하는 경우 오접종사례가 발생할 수 있으므로 가능한 인접하지 않게 각기 다른 선반에 보관하는 등 혼동하지 않도록 주의해야 하며, 유효기간이 짧은 백신 및 희석용제를 앞줄에 배치하여 먼저 사용할 수 있도록 하는것을 알게 되었습니다.

4. 오늘 실습을 통해 새롭게 배운 것들이 향후 간호실무에 어떻게 적용될 수 있겠는가?

백신을 관리하는 담당자로서 일하게 되었을 때 백신이 변질되지 않도록 하는 데에 가장 중요한 백신보관장치의 온도관리에 대한 방법을 정확하게 알고 관리하는데에 적용할 수 있을 것 같습니다. 또 온도계 뿐만 아니라 자동온도기록장치라는 것에 대해 알고 이를 사용하여 더 정확하고 신속하게 온도 알람을 듣고 즉시 백신 보관이 제대로 된 방법으로 행해질 수 있도록 하는데에 적용할 수 있는 것 같습니다. 또한 오접종을 예방하기 위해서 비슷하게 생긴 모양이거나 포장을 사용하는 백신이나 소아나 성인용을 구분해야 하는 경우 꼭 다른 선반에 배치하여 혼동하지 않도록 하고 제일 앞줄에 먼저 사용해야 하는 백신을 배치하여 폐기하는 일이 없도록 하는데에 적용할 수 있을 것 같습니다.

5. 오늘 실습에서 좋았던 점과 아쉬웠던 점은 무엇인가?

백신을 보관하고 있는 여러 종류의 백신보관 냉장고와 다양한 백신의 종류를 볼 수 있었고, 폐렴구균이나 HPV, 결핵(BCG), A형간염, B형간염에 대한 접종의 필요성, 접종 후 주의사항 등에 대해 설명해주는 책자를 많이 보고 새롭게 배울 수 있었던 내용이 많이 있어서 좋았습니다. 또 나라에서 비용을 지원해줌으로써 무료로 접종이 가능한 백신의 종류도 볼수 있었고 이에 대한 내용을 해당 대상자들에게 문자로 안내를 하여 생각보다 많은 분들이 예방접종을 하러 오신다는 것을 보고 알수 있어서 좋았습니다.

1. 오늘 실습경험의 의미 있는 점은 무엇인가?

내가 실습하게 된 부서는 구강보건센터였는데 보건소에 있는 구강보건센터는 한번도 와본적이 없어 어떠한 일들을 하는지 잘 몰랐던 것들을 알게 되었고, 한 어린이집에서 아이들이 단체로 견학을 와서 양치와 치아건강에 대한 교육을 듣고 직접 양치체험도 해보고 의사선생님께 진료를 받는 프로그램을 볼수 있었고, 일반인들이 와서 무료로 받아갈 수 있는 불소를 직접 용기에 채우는 일도 해보았습니다.

2. 오늘 실습을 하는 동안 나의 생각과 느낌은 어떠했는가?

초등학교에 다닐때 수요일마다 보건실에서 불소 용액을 받아와서 불소양치를 했던 경험이 있는데 그때 당시에는 불소용액이 무엇인지, 왜 하는 것인지도 잘 모른채 그냥 따라만했었는데 이번 실습을 통해 불소양치가 왜 필요한지 알수 있게 되었고, 불소용액도 직접 용기에 담아보는 작업을 해보니 신기하기도 하였습니다. 또 어린이집 아이들이 단체로 와서 구강보건 교육을 애니메이션 영상을 보면서 배우고 직접 양치해보는 모습들을 보면서 저도 어린나이부터 이러한 교육을 받고 잘 실천했다면 지금보다 치아가 건강했지 않을까 하는 생각도 하게 되었습니다.

3. 오늘 실습을 통해 새롭게 알게 된 것은?

구강보건센터에서 하는 일은 노인의치보철사업, 장애인 및 취약계층 무료진료, 학교구강보건사업 및 어린이 충치예방사업, 불소용액 양치사업, 노인불소도포 및 스케일링 사업, 구강보건교육 및 양치체험교실 운영, 사회복지시설 구강건강관리사업, 미취학 어린이 구강건강관리 사업 등을 도맡아 하고 있다는 것을 알게되었습니다. 또 유치가 빠지는 아이들도 구강검진을 받아야 하는 이유가 빠지게 될 유치이더라도 건강해야 음식을 잘 씹어서 섭취할 수 있으며 제대로 기능하지 못할 경우, 아이의 성장과 발육은 물론 심리상태에도 영향을 미칠 수 있기 때문이라는 것을 새롭게 알게 되었습니다. 또 불소는 치아를 구성하는 '수산화인회석' 성분과 결합하며 치아를 더 튼튼하게 만들고, 충치로부터 치아를 보호해주기 때문에 사용해야 한다는 것을 알게되었습니다.

4. 오늘 실습을 통해 새롭게 배운 것들이 향후 간호실무에 어떻게 적용될 수 있겠는가?

치과 검진이 어려운 장애인이나 취약계층에게 보건소의 구강보건센터를 소개해줌으로써 차별없이 모두가 구강건강관리를 할 수 있도록 하고, 불소를 왜 사용하고, 어떻게 사용해야하는지 배우게 된것을 바탕으로 소아나 청소년은 6개월에 한번씩 불소도포를 받아 충치를 예방할 수 있도록 하고, 성인이나 어르신은 불소도포를 받아 치아뿌리의 충치를 예방하고, 치아가 시린증상을 완화할 수 있도록 하는데에 적용할 수 있을 것 같습니다. 또한 아직 영구치가 아닌 유치가 있는 어린아이들이더라도 치아건강에 신경써야 제대로 된 성장과 발육을 할수있다는 것을 알고 교육할수 있을 것 같습니다.

5. 오늘 실습에서 좋았던 점과 아쉬웠던 점은 무엇인가?

어린이집 아이들이 구강보건교육과 양치체험교실을 할 때 양치하는 것을 옆에서 도와주고 검진 후 받아가는 선물과 구강관리 수첩을 나눠주는 일을 경험해볼 수 있어서 좋았습니다. 또 직접 불소용액을 빈용기에 담아 채우고 불소를 어떻게 사용하는지 배웠으며 저희가 만든 불소용액을 가져갈 수 있어 재미있었고, 구강보건센터에서 어떠한 일을 하는지와 구강보건센터 내의 치과처럼 되어있는 시설들을 실제로 볼 수 있어 의미있는 경험이었습니다.

1. 오늘 실습경험의 의미 있는 점은 무엇인가?

오늘 실습하게 된 부서는 구강보건센터였는데 보건소에 있는 구강보건센터는 한번도 와본적이 없어 어떠한 일들을 하는지 잘 몰랐던 것들을 알게 되었고. 한 어린이집에서 아이들이 단체로 견학을 와서 양치와 치아건강에 대한 교육을 듣고 직접 양치체험도 해보고 의사선생님께 진료를 받는 프로그램을 볼수 있었고. 일반인들이 와서 무료로 받아갈 수 있는 불소를 직접 용기에 채우는 일도 해보았습니다.

2. 오늘 실습을 하는 동안 나의 생각과 느낌은 어떠했는가?

초등학교에 다닐때 수요일마다 보건실에서 불소용액을 받아와서 불소양치를 했던 경험이 있는데 그때 당시에는 불소용액이 무엇인지, 왜 하는 것인지도 잘 모른채 그냥 따라만했었는데 이번 실습을 통해 불소양치가 왜 필요한지 알수 있게 되었고, 불소용액도 직접 용기에 담아보는 작업도 해보니 신기하기도 하였습니다. 또 어린이집 아이들이 단체로 와서 구강보건 교육을 애니메이션 영상을 보면서 배우고 직접 양치해보는 모습들을 보면서 저도 어린나이부터 이러한 교육을 받고 잘 실천했다면 지금보다 치아가 건강했지 않을까 하는 생각도 하게 되었습니다.

3. 오늘 실습을 통해 새롭게 알게 된 것은?

구강보건센터에서 하는 일은 노인의치보철사업, 장애인 및 취약계층 무료진료, 학교구강보건사업 및 어린이 충치예방사업, 불소용액 양치사업, 노인불소도포 및 스케일링 사업, 구강보건교육 및 양치체험교실 운영, 사회복지시설 구강건강관리사업, 1유아 어린이 구강건강관리 사업 등을 도맡아 하고 있다는 것을 알게되었습니다. 또 유치가 빠지는 아이들도 구강검진을 받아야 하는 이유가 빠지게 될 유치이더라도 건강해야 음식을 잘 씹어서 섭취할 수 있으며 제대로 기능하지 못할 경우, 아이의 성장과 발육은 물론 심리상태에도 영향을 미칠 수 있기 때문이라는 것을 새롭게 알게 되었습니다. 또 불소는 치아를 구성하는 '수산화인회석' 성분과 결합하여 치아를 더 튼튼하게 만들고, 충치로부터 치아를 보호해주기 때문에 사용해야 한다는 것을 알게되었습니다.

4. 오늘 실습을 통해 새롭게 배운 것들이 향후 간호실무에 어떻게 적용될 수 있겠는가?

치과 검진이 어려운 장애인이나 취약계층에게 보건소의 구강보건센터를 소개해줌으로써 차별없이 모두가 구강건강관리를 할 수 있도록 하고, 불소를 왜 사용하고, 어떻게 사용해야하는지 배우게 된것을 바탕으로 소아나 청소년은 6개월에 한번씩 불소도포를 받아 충치를 예방할 수 있도록 하고, 성인이나 어르신은 불소도포를 받아 치아뿌리의 충치를 예방하고, 치아가 시린증상을 완화할 수 있도록 하는데에 적용할 수 있을 것 같습니다. 또한 아직 영구치가 아닌 유치가 있는 어린아이들이더라도 치아건강에 신경써야 제대로 된 성장과 발육을 할수있다는 것을 알고 교육할수 있을 것 같습니다.

5. 오늘 실습에서 좋았던 점과 아쉬웠던 점은 무엇인가?

어린이집 아이들이 구강보건교육과 양치체험교실을 할 때 양치하는 것을 옆에서 도와주고 검진 후 받아가는 선물과 구강관리 수첩을 나누어주는 일을 경험해볼 수 있어서 좋았습니다. 또 직접 불소용액을 빈용기에 담아 채우고 불소를 어떻게 사용하는지 배웠으며 저희가 만든 불소용액을 가져갈 수 있어 재미있었고, 구강보건센터에서 어떠한 일을 하는지와 구강보건센터 내의 치과처럼 되어있는 시설들을 실제로 볼 수 있어 의미있는 경험이었습니다.

P4

1. 오늘 실습경험의 의미 있는 점은 무엇인가?

- 빈 통에 불소를 넣고 상자에 정리하기.
- 구강검진을 하러 온 환자분 치료하는 것 관찰.
- 감염병 매개 활동, 치매, 결핵 전체 OT 듣기.

2. 오늘 실습을 하는 동안 나의 생각과 느낌은 어떠했는가?

보건소 실습하는 것 자체가 이번에 두 번째라서 많이 떨리기도 하고, 처음했던 보건소와 같은 행동을 해야되지 않을까라는 생각이 들었다. 하지만 오늘 실습하면서 여러 부서를 갈 수 있게 해주시면서 보건소에 대해서 친절하게 알려주셔서 처음 갔던 보건소와는 다른 느낌이 들었던 하루였던 것같다.

3. 오늘 실습을 통해 새롭게 알게 된 것은?

- 불소양치용액.
- 효과: 치아우식증(충치) 예방., 시린 효과
- 방법: 하루에 한 번, 칫솔질을 깨끗이하고 10cc 정도의 불소양치용액을 작은 양치컵에 따라 1분가량 입안에 머금은 후 뱉는다.
  ↳ 양치 후에는 30분 가량 음식이나 물로 헹구지 않는다.
- 성분: 0.05% NaF(불화나트륨) 용액

4. 오늘 실습을 통해 새롭게 배운 것들이 향후 간호실무에 어떻게 적용될 수 있겠는가?

만약 대상자가 불소양치용액에 대한 설명을 한다면은 충치와 시린 효과를 예방하기 위해서 사용하는 것이며, 하루에 한 번 사용하지만 칫솔질을 깨끗히 한 후에 10cc를 작은 양치컵에 따라 1분 가량 머금은 후 뱉어야 하고, 양치 후 30분 가량 음식이나 물로 헹구지 않아야 함을 설명할 것이다.

Q 공식적 사업명?

5. 오늘 실습에서 좋았던 점과 아쉬웠던 점은 무엇인가?

좋았던 점: 구강보건센터 실습을 하면서 오전에는 불소용액을 가져갈려고 하시는 분들이 많아 오후에도 똑같겠다라는 생각이 들었는데, 2시쯤 넘어서 한 두명씩 구강검진을 하러 온 대상자가 있어서 구강검진을 하는 모습을 관찰 수 있어서 좋았다.

1. 오늘 실습경험의 의미 있는 점은 무엇인가?

건강증진실에서 실습하면서 건강증진실에서는 혈압, 혈당, 인바디, 체력단련실 등이 이루어지는 곳이다. 또한 체력단련실에서는 월, 수, 금동안에는 프로그램도 운영한다고 한다. 여러 회원분들께서 오셔서 인바디나 체력단련실 이용, 운동 어플에 대한 물음에 대해서 많이 찾아오셨는데 그때마다 어떻게 대응해야 되는지에 대해서 볼 수 있어서 좋았다.

2. 오늘 실습을 하는 동안 나의 생각과 느낌은 어떠했는가?

건강증진실에 오시는 회원분들께서 많이 계셨는데 그때마다 회원분들에게 상담을 해주시는 선생님께서 달랐다. 알고 보니 선생님들께서는 영양선생님, 운동치료사, 간호사 등으로 이루어져 있어서 놀랐다. 대부분 병원에서는 서로 다른 부서에 존재하여 간단한 의사소통만 하는데, 여기서는 아예 한 부서로 같이 서로 도와주면서 일을 하시는 것을 보아 되게 신기하였다.

3. 오늘 실습을 통해 새롭게 알게 된 것은?

①. 건강생활실천 클리닉 운영

- 건강생활을 실천하고자 하는 시민에게 체성분 분석 및 상담을 통해 개인별 특성에 맞는 운동과 영양상담 서비스.

②. 건강생활실천 프로그램 운영

- 신체 증가 및 강화로 건강생활을 실천하여 비만탈출 및 대사증후군 예방관리

4. 오늘 실습을 통해 새롭게 배운 것들이 향후 간호실무에 어떻게 적용될 수 있겠는가?

만약 회원님께서 상담을 통해 고민이 되는 부분을 해결할 수 있게 하는 프로그램을 소개시켜준 뒤 이 프로그램에서는 어떤 활동으로 이루어지고 언제 활동을 하는지에 대해서 설명시켜줄 것이며, 꾸준히 활동할 수 있도록 격려도 함께 해줄 것이다.

5. 오늘 실습에서 좋았던 점과 아쉬웠던 점은 무엇인가?

아쉬웠던 점: 체력단련실에서 프로그램 체험을 오전과 오후로 나뉘어지는데 오전에는 컨퍼런스와 오후에는 구강보건센터에서 일손을 도와주느라 따로 참여하지 못했다는 것이 아쉬웠던 것 같다.

1. 오늘 실습경험의 의미 있는 점은 무엇인가?

건강보험공단에서 실습하면서 여러 대상자에게 건강검진이 얼마 남지 않았으니 검진을 할 수 있도록 전화를 하는 것 자체가 가장 의미 있는 것이라고 생각한다. 간단한 업무더라도 실습생에게 전화업무를 믿고 맡겨주셨더라는 생각이 들었기 때문이다.

2. 오늘 실습을 하는 동안 나의 생각과 느낌은 어떠했는가?

공단에서 실습하는 동안 여러 대상자에게 건강검진을 6월30일까지 해야되는 것에 대해 안내를 해주는 것을 하였는데, 전화하면서 친절하게 대답을 해주시는 반면 버럭 화를 내시는 분도 계시기도 하였다. 하루종일 전화를 하는 동안 다른 곳에서 만약 나에게 전화를 주시면 친절하게 이야기를 해야겠다라는 생각이 많이 들었던 하루였던 것 같다.

3. 오늘 실습을 통해 새롭게 알게 된 것은?

◦간암(6개월주기).
- 검진비용 10% 본인부담.
- 40세 이상 간암발생 고위험군 남녀.
- 간 초음파검사 + 혈청알파태아단백검사.

※ 고위험군.
◦ 해당연도 2년간 보험급여내역 중 간암발생 고위험 질병으로 진료받은 내역있는 자
◦ 과거연도 B형간염표면항원 또는 C형간염항체 검사 결과 '양성'으로 확인된 자

4. 오늘 실습을 통해 새롭게 배운 것들이 향후 간호실무에 어떻게 적용될 수 있겠는가?

간암 건강검진은 1년에 왜 2번씩 해요?, 이번 왜 또 해야되나요? 라는 질문을 많이 하신다. 그럴 경우에 간암 건강검진은 6개월에 한 번씩 진행하며 상반기, 후반기 나누어서 진행이 되고 있다고 답변할 것이다. 또한 만약 간암 건강검진은 무슨 검사를 해야되나요? 에 대한 질문에서는 간 초음파 검사와 피 검사를 진행해야된다고 이야기할 것이다.

5. 오늘 실습에서 좋았던 점과 아쉬웠던 점은 무엇인가?

좋았던 점은 대상자에게 건강검진 안내에 대해 설명한 점이고
아쉬웠던 점은 대상자분께서 건강검진을 제외하고 다른 것에 대해 질문을 하였을 때에 아직 정보가 없어서 자세히 알려주지 못했다는 점이다.

1. 오늘 실습경험의 의미 있는 점은 무엇인가?

어제와 똑같이 회원분들에게 간암 예방접종을 받으시라고 안내하는 것을 이어하는 시간을 가졌다. 어제와 이어서 해서 받은 종이는 똑같았으며, 총 300명정도를 이틀에 나눠서 했었는데 시간내에 300명정도의 회원분들께 연락을 할 수 있었다는 점이 좋았다.

2. 오늘 실습을 하는 동안 나의 생각과 느낌은 어떠했는가?

지역사회 실습이 벌써 한 주가 지났다는 것이 놀라웠다. 그만큼 시간도 정말 빨리 가고 즐거웠던 실습이었던 것 같다. 다음주에는 또 다른 부서를 가서 어떠한 내용을 배울지에 대해서 궁금하다. 또한 공단에 실습을 이틀 째 하면서 느낀 것은. 업무를 하다가 중간 중간에 전화가 엄청 많이 와 업무 중간에도 전화를 받고 상담해주시고 전화 끝나면 다시 업무를 하시는 모습을 보고 스트레스를 받을 것 같으면서도 대상자의 상담 내용이 해결되었다는 뿌듯함을 느껴 양가감정을 느꼈을 ☆ 것이라는 생각이 들었다.

3. 오늘 실습을 통해 새롭게 알게 된 것은?

공단에 지원하기 위해서는 학교교육, 직업교육, 경험, 경력, 면허 등을 가지고 있어야 지원응시 자격이 부여되며, 이를 합격할 경우에는 필기시험을 본다. 시험 내용에서는 직업기초능력 응용모듈 60문항을 보게 되고 이를 합격할 경우 인성시험 후 면접을 보게 된다는 점이다.

↳ 만약 행정직으로 가고 싶을 경우에는 800점의 토익이 필요하다.
지역인재 특채로는 강원도에만 존재한다.

4. 오늘 실습을 통해 새롭게 배운 것들이 향후 간호실무에 어떻게 적용될 수 있겠는가?

만약 처음부터 공단에 들어가고 싶을 경우에는 대학교를 다니면서 직업교육 및 한국사 자격증, 컴퓨터 자격증 등을 미리 따야하고, 토익 같은 경우에도 미리 준비해야 된다는 점이다. 또한 만약 자격응시에서 합격할 경우를 대비하여 직업 기초능력에 대해서 미리 조금씩 공부해놓을 것이다.

5. 오늘 실습에서 좋았던 점과 아쉬웠던 점은 무엇인가?

좋았던 점 : 국민보험 공단에서 첫날 실습할 때 선생님께서 전화를 해야할 대상자의 명단표를 주셨을 때 이걸 어떻게 이틀 안에 마치지라는 생각이 들었는데 이틀 날 교습인 오늘 선생님께서 주신 명단에 있는 대상자에게 모두 다 전화를 할 수 있어 좋았다.

1. 오늘 실습경험의 의미 있는 점은 무엇인가?

오늘 실습한 장소는 예방접종실이다. 대상자분께서 오셔서 A형, B형 간염, 폐렴구균 예방접종을 하시러 많이 오셨다. 그래서 대상자분께서 접종을 받기 전에 예진표 작성하시는 것을 도와드렸으며, 접종을 받는 모습 등을 관찰할 수 있어서 의미있었던 것 같다.

2. 오늘 실습을 하는 동안 나의 생각과 느낌은 어떠했는가?

예방접종실이라서 어린 아이들이나 나이가 드신 대상자들만 오실줄 알았는데, 생각 외외로 젊은 대상자분들이 오셔서 예방접종을 하셔서 조금 신기하였다. 또한 접종을 하기 전에 대상자분께서는 의사 상담을 받고 있는 동안에 선생님들께서 약물에 대한 설명과 어떤 대상자마다 바늘 굵기 등을 사용해야 되는지에 대해 자세히 설명해주셔서 좋았다.

3. 오늘 실습을 통해 새롭게 알게 된 것은?

폐렴 예방접종

- 폐렴에 있는 질병 예방하기 위해 접종
- 23가와 19가의 예방접종이 존재하지만, 23가는 65세 이상 대상자분들께 보건소에서 무료로 1번 접종 중이며, 19가의 경우에는 면역력이 약한 분들께서 추천하며, 병원에 가셔서 유료로 접종해야 한다.

↳ 만약 23가 맞고 19가를 맞고 싶은 경우 1년 뒤에 접종해야 됨.

4. 오늘 실습을 통해 새롭게 배운 것들이 향후 간호실무에 어떻게 적용될 수 있겠는가?

65세 이상 대상자분들께서 폐렴 예방접종을 하러 오셨는데 만약 처음이실 경우에는 대부분 보건소에서 '1차' 무료로 받는다는 점을 설명하고 약물은 23가를 사용할 것이라고 설명할 것이다. 그리고 만약 면역력이 약할 경우에는 23가를 맞고 1년뒤에 병원에 가셔서 19가 예방접종을 하도록 권고할 것이다.

5. 오늘 실습에서 좋았던 점과 아쉬웠던 점은 무엇인가?

좋았던 점은 A형간염, B형 간염, 폐렴구균에 대해 사용하는 약물에 대해서 자세히 설명해주고 예방접종하는 모습까지 관찰할 수 있었다는 점과
반면 아쉬웠던 것은 아이들의 예방접종하는 것을 관찰하고 싶었는데 관찰을 하지 못해서 아쉬웠다.

1. 오늘 실습경험의 의미 있는 점은 무엇인가?

오늘의 방문건강관리실에서 실습하였다. 방문건강관리실에서 선생님께서는 매일 방문 간호 및 교육을 하시느라 출장을 나가야된다는 것에 대해 새로운 사실을 알게 되어서 좋았다.
또한 여러 대상자들을 만나면서 혈압과 혈당을 잴 수 있어서 좋았다는 것 자체가 의미있었다.

2. 오늘 실습을 하는 동안 나의 생각과 느낌은 어떠했는가?

경로당에 방문간호를 가게 되었는데 들어가기 전에 선생님께서 체조를 한 뒤에 혈당과 혈압을 재야된다고 하셔서 무슨 소리인가 했는데 들어가보니 미리 오신 체조 선생님께서 체조를 하고 계셨던 것이다. 그걸을 맞춰서 체조를 10분 정도 하였는데도 힘들었는데, 할머니님들께서는 오시기 전에 이미 체조를 하고 계셨을텐데 지치지 않으시고 열심히 참여하는 모습을 보고 정말 대단하시고, 운동을 더 해야할 것 같다라는 생각이 들었다.

3. 오늘 실습을 통해 새롭게 알게 된 것은?

∘ 방문건강관리사업
- 동 주민센터에 방문간호사를 배치하여 건강관리부터 주민센터 방문 상담까지 다양한 건강 서비스를 제공한다

∘ 방문간호 대상
①. 65세 이상 어르신.
②. 기초생활보장수급자, 차상위 계층, 북한이탈주민, 지역아동센터, 다문화가족, 65세 이상 홀몸어르신, 75세 이상 노인부부 가구, 한부모가족, 조손가족, 암 및 정신질환자 가족

4. 오늘 실습을 통해 새롭게 배운 것들이 향후 간호실무에 어떻게 적용될 수 있겠는가?

만약 방문간호를 신청하러 온 대상자가 있다면 위에 방문간호 대상에 해당이 되는지에 대해 확인해야 하며, 확인을 한 후 대상자가 맞으면 방문간호를 실시할 것이다. 또한 방문을 하여 대상자의 혈당, 혈압 등을 측정하고, 간단한 교육도 실행할 것이다.

5. 오늘 실습에서 좋았던 점과 아쉬웠던 점은 무엇인가?

좋았던 점은. 방문간호를 가게 될 경우 대부분 1번 정도만 가게 되는데, 이번 좋은 기회가 되어서 2번 정도 나가게 되어서 좋았고, 2번 정도 나갈 때 간호사선생님께서도 다르셨는데 자신의 스타일을 이용하여 대상자를 대하는 모습을 관찰할 수 있어서 좋았다.

1. 오늘 실습경험의 의미 있는 점은 무엇인가?

오늘 실습한 구간이 이동진료팀이다. 하지만 오늘 이동진료를 갈 일이 없다고 하셔서 앉아서 공부를 하고 있는 도중에 구강보건센터에서 일손이 필요하다고 하셔서 지원을 가 불소를 만들고 왔다는 것이 의미있었다.

2. 오늘 실습을 하는 동안 나의 생각과 느낌은 어떠했는가?

이동진료를 이러한 친구들한테 이동진료를 물어봤을 때에 이동진료는 그날 나가는 것이 결정이 되어있어서 나갈지도 안 나갈지도 모른다고 하여 아침에 실습지에 도착하고 선생님을 만나기 전까지는 그래도 오늘은 있겠지라는 생각이 많이 들었는데 선생님께서 없다고 하셔서 아쉽다라는 생각이 들었다.

3. 오늘 실습을 통해 새롭게 알게 된 것은?

이동진료.

◦ 대상.

- 의료취약지역 주민, 사회복지시설, 시설아동 등 (진료지는 매년 1월 중 취약지역 중 선정).

◦ 내용

- 일반진료 및 투약
- 한방진료 및 투약
- 혈압측정, 혈액검사, 방사선검사, 건강진단 등.

4. 오늘 실습을 통해 새롭게 배운 것들이 향후 간호실무에 어떻게 적용될 수 있겠는가?

만약 나중에 이동진료팀에 들어갔을 경우에 우선 대상자를 선정하고 진료지를 선정할 것이며, 진료지는 대상자가 편안한 장소이며 다른 사람들에게 피해를 주지 않는 곳으로 선정할 것이다. 또한 이동진료로 하러나가 대상자에게 할 수 있는 활동인 진단에 맞는 약 투약, 혈당, 혈액, 혈압 등을 할 것이다.

5. 오늘 실습에서 좋았던 점과 아쉬웠던 점은 무엇인가?

실습을 하면서 아쉬웠던 점은 이번 실습부서가 이동진료팀이었는데 따로 진료를 하러 나가지 못해서 아쉬웠기도 하였고, 경험도 못했다는 것도 아쉬웠던 것 같다.

1. 오늘 실습경험의 의미 있는 점은 무엇인가?

치매안심센터에서 한 어르신께서 인지선별검사 하는 모습을 관찰하였다. 정신간호학 시간에 배운 내용이랑 비슷하여 다시 인지선별검사에 대해 생각할 수 있어서 좋았다. 또한 실제로는 인지선별검사를 하는 모습도 관찰하지 못하였는데 이번 실습에서 관찰할 수 있어서 좋았던 것 같다.

2. 오늘 실습을 하는 동안 나의 생각과 느낌은 어떠했는가?

치매안심센터에서 근무하시는 선생님께서 치매안심센터에 대해서 설명해주셨는데, 치매안심센터에서 운영하는 프로그램의 종류가 많이 존재하는지에 대해서 처음 알게되었다. 군산시 보건소에서 운영하는 것은 작게 운영이 되고 있는 것이라서 별로 없겠다라는 생각이 많이 들었는데 이번에 설명을 들으면서 많이 프로그램도 있고 지원도 많이 하고 있구나 라는 것을 알게 되어서 좋았다.

3. 오늘 실습을 통해 새롭게 알게 된 것은?

치매안심센터 프로그램.

①. 치매환자 쉼터.
- 치매 환자 대상으로 인지 활동 교육.

②. 치매 예방 교실.
- 치매진단을 받지 않은 60세 이상 어르신들을 대상으로 치매예방을 위한 인지교육

③. 인지 강화 교실.
- 치매선별검사 상 인지저하, 정밀검사 결과 경도인지장애로 진단 받은 어르신들을 위한 인지활동교육

4. 오늘 실습을 통해 새롭게 배운 것들이 향후 간호실무에 어떻게 적용될 수 있겠는가?

치매안심센터에 오신 60세 이상 어르신분께서 만약 프로그램의 활동을 원하신다면 우선 경도인지치매인지 아닌지에 대해 간단한 테스트를 통해서 판별한 후 만약 치매진단을 받지 않을 경우에는 치매 예방 교실을 추천하고 경도인지치매 판정을 받았다면 치매환자 쉼터, 인지강화교실을 추천하며, 가족 및 보호자 대상으로 운영하는 치매 가족 지원 교실도 추천할 것이다.

5. 오늘 실습에서 좋았던 점과 아쉬웠던 점은 무엇인가?

실습을 하면서 아쉬웠던 점은 상담실이 한군데에서 만약 정밀검사를 하러 온 어르신분들이 계시면은 상담실이 실시하는데 거기 공간이 좁아서 관찰을 하지 못했던 것이 아쉬웠던 것 같다.

1. 오늘 실습경험의 의미 있는 점은 무엇인가?

방문건강관리실 실습을 하며 방문간호를 나가보았던 점이 의미 있었다.
방문간호를 나주로 가서 경로당 두곳을 방문했고, BP랑 BST 측정을 할수 있었다.
항상 병원에서만 해오던 것들을 출장을 다니며 한 점이 의미있었고, 방문간호 업무를 체험해본 점이 의미있었다.

2. 오늘 실습을 하는 동안 나의 생각과 느낌은 어떠했는가?

생각보다 DM · HTN을 가진 노인이 많다는 생각을 했다. 그리고 지속적으로 방문해 확인하고 식습관이나 생활습관을 교정해주시지만, 한평생 살아온 습관이 한달에 한번 방문하는 것으로 고쳐지기는 어려울 것 같다는 생각을 했다.
선생님께서 지원물품이 나오지 않아 2월 이후로 방문하지 못했던 것도 있었는데 국가차원에서 노인의 만성질환에 집중해 관리해줄 필요가 있다고 생각했다.

3. 오늘 실습을 통해 새롭게 알게 된 것은?

SFTS라는 진드기 매개 감염에 대해 교육을 받게 되었다.
SFTS는 중증열성혈소판감소증후군이라고 불리며 참진드기에 물린다는 특징이 있다. 쯔쯔가무시와 대비하며 배웠을때 털진드기와 차이가 있었는데 털진드기는 검은가피가 생기는 반면 참진드기는 물린자국만 있다고 배웠다.
확진을 위해 물린자국을 찾아 남기는 것이 중요한 과정 중 하나라고 하셨다

4. 오늘 실습을 통해 새롭게 배운 것들이 향후 간호실무에 어떻게 적용될 수 있겠는가?

SFTS 예방법을 교육하고 실천하도록 해 적용할 수 있다고 생각한다.
야외활동시 풀밭에 옷을 벗어 두거나 눕지 않고, 돗자리도 사용한 뒤 잘 세척해 보관할 필요가 있다. 방문간호를 하며 노인분들과 함께 밭·논일 하시는 분을 자주 봤기 때문에 일상복과 작업복을 구분해 착용하고, 옷소매와 바지 끝을 단단히 여며야 함을 교육해야 할 것 같다.

5. 오늘 실습에서 좋았던 점과 아쉬웠던 점은 무엇인가?

센터에만 있지 않고 차를 타고 30분 정도 이동하며 바깥을 볼 수 있어서 좋았다.

1. 오늘 실습경험의 의미 있는 점은 무엇인가?

지역사회 간호과정을 진행할 지역을 선정하고, 업무시간에 보건소를 둘러보며 여러 사업을 보고 방향성을 잡아갔던 점이 의미있었다. 또한 학기중 과제로 군산시 보건소에 대해 조사했었기 때문에 각 업무와 사업을 읽어볼때 조금 더 이해하고 다가가기 쉬웠던 것이 의미있다고 생각했다.

2. 오늘 실습을 하는 동안 나의 생각과 느낌은 어떠했는가?

교수님께서 오셔서 짧은 면담시간을 가졌는데, 과제의 방향성에 대해 이야기를 나누고 틀을 잡을 수 있어 좋았다. 어제 간단히 주제를 정하고 조사를 했었는데 생각보다 어려울 것 같다는 생각을 했고 한 시에 집중하다 보니 어떻게 해야할지 감이 오지 않았는데 이야기를 나누어 조금은 수월하게 진행할 수 있을 것 같다는 생각을 했다.

3. 오늘 실습을 통해 새롭게 알게 된 것은?

지역사회 간호사가 해야하는 역할이 생각보다 아주 많다는 것을 깨달았다. 특히 과제물을 조사하는 과정 중, 보건진료소에서 근무하는 간호사는 의료자원이 부족하기 때문에 대상자의 문제 파악과 상담자·교육자·정보제공자·의뢰자·직접간호 제공자 등 많은 역할을 동시에 수행해야 함을 알았다.

4. 오늘 실습을 통해 새롭게 배운 것들이 향후 간호실무에 어떻게 적용될 수 있겠는가?

추후 대상자의 문제를 정확히 사정하고 진단에 필요한 중재를 적용한다. 교육 및 정보제공을 통해 대상자의 지식향상을 도모하고 필요한 경우 적극적으로 타 기관에 의뢰하여 필요한 사항을 적절히 공급받을 수 있도록 한다.

5. 오늘 실습에서 좋았던 점과 아쉬웠던 점은 무엇인가?

이동진료팀이지만 출동이 없어 나가지 못한 것이 아쉽다.

1. 오늘 실습경험의 의미 있는 점은 무엇인가?

오늘 보건소 행사를 하면서 구강보건 업무와 혈압 BST 측정 업무를 할수 있어 의미있었다. 유치원생부터 고령의 노인까지 전 연령층에게 다양한 보건 사업을 시도해볼 수 있어 의미있었다.

2. 오늘 실습을 하는 동안 나의 생각과 느낌은 어떠했는가?

각 부스마다 선물을 드리면서 간단한 설문조사, 교육·처방. 활력징후 측정 등을 했는데, 초반에는 선물만 뺏아가려고 하시는 분들이 많아 속상하다는 생각을 했다. 하지만 선물을 드리면서 건강증진 행위를 실천하도록 유도하는 것이 목적이니 설득을 통해 많은 참여를 할수 있었다.

3. 오늘 실습을 통해 새롭게 알게 된 것은?

혈당 수치에 대해서 명확히 알게 되었다. 사실 지금까지 혈당을 측정하여 100mg/dl 안으로 들어오면 정상인 것만 인지하고 측정하기도 했고, 병원 내에서 혈당을 측정할때는 이미 당뇨를 가지고 계신분이 많아 비정상이라고만 설명했는데 일반시민 (건강한 사람) 들의 혈당을 측정하여 공복·식후 혈당의 정상치에 대해 알고 설명을 드리며 배웠다.

4. 오늘 실습을 통해 새롭게 배운 것들이 향후 간호실무에 어떻게 적용될 수 있겠는가?

당뇨병을 가지거나, 가지지 않은 일반 사람들 모두를 대상으로 정확한 혈당측정 및 수치 안내를 할수있다.

- 공복혈당 70 ~ 100mg/dl
- 식후 2시간 혈당 90 ~ 140mg/dl

Q. 선생님께서는 200mg/dl 이하는 정상이라고 안내 하셨는데, 검색해보니 위 기준도 있다고 하는데 해당되나요?

대상자의 식사 여부, 음식 섭취 종류와 관련

5. 오늘 실습에서 좋았던 점과 아쉬웠던 점은 무엇인가?

군산시에 거주하며 시민을 대상으로 해본 것이 많이 없는데, 이번 기회를 통해 시민들에게 무언가를 제공했다는 점이 의미있어 좋았고, 활동적인 프로그램들을 할 수 있어 좋았다.

1. 오늘 실습경험의 의미 있는 점은 무엇인가?

보건소 행사를 하며 구강보건센터 업무를 해본 것이 의미있었다.
어제는 설문조사 위주 였다면, 오늘은 불소 사용법과 잇솔질 교육을 해보았고
치위생사와 관련된 지식이라 배워본 적 없는 내용을 학습해보았던 것이
의미있게 느껴졌다.

2. 오늘 실습을 하는 동안 나의 생각과 느낌은 어떠했는가?

구강세트 배분 업무를 하며 한 민원인 분과 말다툼이 있었다.
받아갔는데 받아가지 않은 척 두번이나 왔기 때문이다. 드렸지 않느냐는
말에 화를 내시며 '그렇게 하지 마요' 라고 하시며 불같이 화를 내셨다.
많은 사람에게 기회를 주는것이 공평하다고 생각했고, 다양한 사람에게
행사를 알리는 목적이라고 생각했기에 억울함을 느꼈던 것 같다.

3. 오늘 실습을 통해 새롭게 알게 된 것은?

이빨이 아닌 '치아' 라는 단어가 맞다는 것을 배우게 되었고,
틀니 착용자를 위한 물품을 배분하며 의치 관리법에 대해 공부하고
많은 노년층에게 이를 교육할 수 있었다. 의치를 빼고 자야하는것은
알았지만 이걸 물에 담궈둬야 하는지 몰랐고, 치약을 사용하지 않고
액체세제를 사용해야 한다는 것을 처음 배우게 되었다.

4. 오늘 실습을 통해 새롭게 배운 것들이 향후 간호실무에 어떻게 적용될 수 있겠는가?

병원에 입원하는 많은 연령층 중 노년이 많은 비율을 차지하고,
의치를 사용하는 사람이 많기 때문에 보관법이나 세척을 교육하여
구강위생을 증진시키는 것으로 적용할 수 있다고 생각한다.

5. 오늘 실습에서 좋았던 점과 아쉬웠던 점은 무엇인가?

부스 활동을 하며 어제보다 더 체계적이고 수월하게 진행할 수 있어
좋았다. 그리고 보건소의 다양한 사업을 한 눈에 볼수 있어 좋았다.

1. 오늘 실습경험의 의미 있는 점은 무엇인가?

구강보건센터에서 실습을 하여 한 어르신을 응대한 것이 의미있었다. 사업에 대해 전반적으로 다 알지 못했는데, 틀니 지원을 받고 싶다고 해서 선생님께 안내해드렸고, 다음주 중으로 검진 예정일도 잡으셨다. 지금까지 해왔던 업무와 달라 색다르고, 의미있었다.

2. 오늘 실습을 하는 동안 나의 생각과 느낌은 어떠했는가?

생각보다 보건소에서 구강보건센터의 하는일이 많다고 생각했다. 그리고 센터 내에 치과와 같은 시스템이 모두 마련되어 있어 신기했다. 치과에서는 자세히 볼수 없었던 것들을 볼 수 있어 색달랐다. 노인의치 보철, 취약계층 무료진료, 학교 및 어린이 충치예방, 노인 불소도포 및 스케일링 등 이보다 더 많은 업무가 있었다는걸 처음봤다.

3. 오늘 실습을 통해 새롭게 알게 된 것은?

근무 하시는 선생님께서 임신을 하신 상태에서 임신기 구강변화에 대해 짧게나마 배울 수 있었다. 임신기에 치아 우식증, 치주병이 생길 확률이 더 높다고 한다. 호르몬 변화와 관련되어 있기도 하지만 임산부들이 잦은 간식과 당분섭취를 하기 때문이라고 한다. 임신시 신체변화에 대해 알았지만 구강 내에도 변화가 있다는걸 배웠다.

4. 오늘 실습을 통해 새롭게 배운 것들이 향후 간호실무에 어떻게 적용될 수 있겠는가?

임산부들을 대상으로 임신기 구강관리에 관심을 기울이고, 구강관리 습관을 생활화하여 치아에 유익한 음식을 가려서 먹어야 함을 교육한다. 또한 장차 태어날 아이의 치아건강을 위해서도 중요함을 알린다.

5. 오늘 실습에서 좋았던 점과 아쉬웠던 점은 무엇인가?

오늘은 검진 날이 아니어서 관련된 업무를 관찰하지 못한 것이 아쉽다.

1. 오늘 실습경험의 의미 있는 점은 무엇인가?

건강보험공단에서 암검진 유선독려를 해본것이 의미있었다. 검진율이 떨어지면 이를 독려해야함을 알았지만, 간호사가 직접 전화를 하여 확인하는 사실은 처음알게 되어 의미있었다. 전화를 통해 수검하겠다는 많은 약속을 받았던 것이 건강증진에 기여한 것이라 생각하니 의미있었다.

2. 오늘 실습을 하는 동안 나의 생각과 느낌은 어떠했는가?

처음 전화업무를 해야함을 들었을 때 굉장히 부담스러웠다. 전화로 기관을 대표해 전화를 하면 나의 실수가 큰 영향을 미칠 수 있다는 생각을 했기 때문이다. 하지만 유선전화를 하다보니 금방 익숙해져 업무를 할 수 있었고, 긍정적인 반응을 보여주셔 뿌듯했다. 반대로 한국인은 너무 바빠서 건강검진을 한번 받기도 힘들구나 라는 생각도 했다.

3. 오늘 실습을 통해 새롭게 알게 된 것은?

국가 6대 암 검진에 대해 알 수 있게 되었다. 특히 오늘은 간암을 독려하게 되었는데, 특이하게 간암은 상반기·하반기로 1년에 두번이나 받아야 함을 배웠다. 이유가 궁금해 찾아봤는데 다른 암에 비해서 간암의 5대 생존율이 39.0%로 낮은 편이기 때문이다. 전라북도의 성별 사망원인중 1위가 암이기 때문에 암검진에 대해 독려하는 것이 중요함을 배웠다.

4. 오늘 실습을 통해 새롭게 배운 것들이 향후 간호실무에 어떻게 적용될 수 있겠는가?

군산시 시민들에게 암 조기검진 – 조기발견 – 조기치료로 이어질 수 있도록 지역사회 간호사의 독려를 통해 검진율을 높인다. 주기적으로 대상자들에게 유선독려를 하여 경각심을 일깨우고, 검진기관과 비용 등에 대해 안내한다.

5. 오늘 실습에서 좋았던 점과 아쉬웠던 점은 무엇인가?

건강보험공단에 생각보다 간호사 선생님이 많아서 직장이나 입사 방법에 대해 자세히 알려주셔서 좋았다.

1. 오늘 실습경험의 의미 있는 점은 무엇인가?

예방접종실에서 실습하여 예방접종 준비부터 투약·추후관리까지 직접 관찰할 수 있어 좋았다. 보통은 준비된 것만 보고 내가 맞았었는데 과정을 다 보니 새로웠고, 장갑을 끼고 깨끗한 상태로 해야하는 것을 알게 되어 의미있었다. (지금까지 장갑끼고 맞은적은 없어서)

2. 오늘 실습을 하는 동안 나의 생각과 느낌은 어떠했는가?

예방접종하는 주사가 일체형으로 들어있는게 신기했다. 일반 투약과 같이 바이알을 녹여서 맞는거라고 생각했는데 처음 포장부터 주사기가 들어있던게 신기하게 다가왔다. 그리고 바늘이 생각보다 너무 길어서 놀랐다.. 맞고 나서 아프다고 생각했는데 이유를 알게된것만 같다..

3. 오늘 실습을 통해 새롭게 알게 된 것은?

보건소에서 운영하는 다양한 예방접종 사업에 대해 출근해서 OT를 받게 되었다. 그리고 막힘없이 예방접종 일정을 다 말씀하시는 모습이 존경스러웠다. 어제 간염·간암 관련 업무를 해서인지 B형 간염 예방접종에 대해 자세히 봤고, 0.1.6개월 단위로 맞는데 이식아의 경우는 0.1.2.6개월로 총 4번을 맞는다는 것을 알게되었다.

4. 오늘 실습을 통해 새롭게 배운 것들이 향후 간호실무에 어떻게 적용될 수 있겠는가?

B형간염 주산기 사업에 대해 홍보하고 교육적으로 개입할 수 있다. 예방처치 일정에 따라 접종·검사를 적기에 완료하도록 도와주고, 항원항체 결과에 따른 추후관리 일정도 함께 교육해야한다.

5. 오늘 실습에서 좋았던 점과 아쉬웠던 점은 무엇인가?

교수님과 컨퍼런스를 하면서 사업에 대해 계획을 짤수 있어서 좋았고, 서로에게 장점·단점을 말하는 방식이 나에게도 도움이 많이 된 것같아 좋았다.

1. 오늘 실습경험의 의미 있는 점은 무엇인가?

치매안심센터에서 근무하며 치매 조기검진을 관찰해볼 수 있어 의미 있었다. 그리고 방문하시는 분들에게 사업안내 등을 해줄수 있어 의미있었다. 생각보다 방문자가 많아 당황했지만, 그만큼 치매에 대해 알고 지원을 받고자 하는 것 같아 다행이라고 생각했다.

2. 오늘 실습을 하는 동안 나의 생각과 느낌은 어떠했는가?

인지치매선별검사(CIST)를 보는데 생각보다 어려워서 당황했다. '민수가 자전거를 타고 야구를 하러갔다' 라는 문장이 있는데 한참 뒤에 다시 물어보니 나도 환자분 옆에서 같이 고민했다. 그리고 생각보다 치매에 걸렸을까 불안해하시는 모습을 자주 봐서 마음이 안쓰럽기도 했다.

3. 오늘 실습을 통해 새롭게 알게 된 것은?

치매안심센터에서 근무하며 보건소 주요사업을 알게되었는데, 특히 보건소가 치매조기검진을 제공하지만 치매진단을 내려줄수 없다는 부분을 새롭게 알게 되었다. 조기검진이 곧 진단으로 이어질 것이라고 생각했는데 알던 것과 다른 부분이 있어 새롭게 느껴졌다. 그리고 조호물품 사업으로 기저귀·물티슈 제공되는데 이게 평생 한번 1회만 제공됨을 알게 되었고, 부족하다는 생각을 하게 되었다.

4. 오늘 실습을 통해 새롭게 배운 것들이 향후 간호실무에 어떻게 적용될 수 있겠는가?

조기검진이 곧 진단으로 이어지지 않더라도, 병원으로 안내해서 진단을 받고 안심센터 내 사업을 이용할 수 있도록 한다. 특히 실종예방서비스에 등록해 지문등록·배회인식표 배부, 배회감지기 배부를 받도록 해서 요즘 많이 일어나는 또래 우리 어르신들이 배회하는 행태를 개선하도록 한다.

5. 오늘 실습에서 좋았던 점과 아쉬웠던 점은 무엇인가?

실습이 끝나서 너무 좋아요 ~~~~ ♡!!!

1. 오늘 실습경험의 의미 있는 점은 무엇인가?

오늘 의미있었던 점은 군산시 보건소의 구조를 파악하고 각 과에서 어떤일을 하는지 알 수 있었다는 점이다.

2. 오늘 실습을 하는 동안 나의 생각과 느낌은 어떠했는가?

보건소 OT를 듣는 동안 학기 중에 군산시 보건소에 대하여 조사한것이 떠올랐다. 지역사회간호학 과제로 군산시 보건소에서 하는 업무, 각부서에 대해 미리 파악할 수 있어서 실습 중 보건소 업무에 무엇이 있는지 알아보는데 도움이 될것 같다.

3. 오늘 실습을 통해 새롭게 알게 된 것은?

오늘 감염관리과 OT를 통해 진드기 매개 감염병의 예방과 조기치료가 중요하다는 것을 깨달았다.

진드기
- 털진드기 : 증상 - 고열, 검정딱지 / 서식지 - 숲 / ex) 쯔쯔가무시
- 참진드기 : 증상 - 고열, 구토, 빨간자국 / 서식지 - 풀밭 / ex) 중증열성 혈소판감소 증후군 (SFTS)

4. 오늘 실습을 통해 새롭게 배운 것들이 향후 간호실무에 어떻게 적용될 수 있겠는가?

진드기 매개 감염병에 걸리는 사람은 주로 농업에 종사하며, 증상은 고열, 오한, 구토, 설사 등이다. SFTS의 경우 치명률이 20%이므로 만약 내가 감염관리과에서 일한다면 예방방법 (진드기 기피제 사용, 전신을 덮도록 옷착용, 일반옷과 분리하여 세탁, 바닥에 앉을땐 돗자리 사용 등)을 사람들에게 홍보할것이다. 응답!

5. 오늘 실습에서 좋았던 점과 아쉬웠던 점은 무엇인가?

좋았던 점은 군산시 보건소를 둘러보고 구조 및 업무를 알 수 있었다는 점이고, 아쉬웠던 점은 오늘 이동진료팀에서 이동일정이 없었다는 점이다.

1. 오늘 실습경험의 의미 있는 점은 무엇인가?

오늘 의미있었던 점은 방문건강관리 사업에 대해 알아봤다는 점이다.
- 방문건강관리사업 : 보건소에 소속된 방문간호사가 각 가정을 방문하여 가족과 건강문제를 가진 가구원을 발견하여 질병예방 및 관리, 건강증진을 위하여 건강서비스를 제공하는것.

2. 오늘 실습을 하는 동안 나의 생각과 느낌은 어떠했는가?

경로당에 처음 방문하는거라서 신기하고 두근거렸다. 들어가자마자 인사했더니 반갑게 맞아주셔서 기분이 좋았다. 어르신들께서 혈압·혈당 측정값에 관심이 많으셔서 결과에 대해 자세히 설명하고 또, 생활습관 개선방안에 대해 교육하는것이 중요하다고 느꼈다.

3. 오늘 실습을 통해 새롭게 알게 된 것은?

- 방문간호대상 : 기초수급자, 독거노인, 차상위계층, 장애인, 독거노인, 타기관에서 의뢰한 건강문제가 있는 대상자, 재가암 환자 관리, 재가 장애인, 집단 서비스 (경로당).
- 방문건강관리의 목표
  - 건강행태개선 : 건강상태 인식, 건강생활 실천 유도, 건강지식향상
  - 건강문제 관리 : 건강문제 정기적 스크리닝, 증상조절, 치료 순응 향상

4. 오늘 실습을 통해 새롭게 배운 것들이 향후 간호실무에 어떻게 적용될 수 있겠는가?

방문간호사가 지역사회 건강증진을 위해 어떻게 건강관리를 돕는지 알게되었다. 관련 교육자료를 보고 대상자에게 어떻게 교육내용을 효과적으로 전달하는지 배웠다. 오늘 배운 것을 토대로 내가 방문 간호를 할 경우, 대상자의 V/S과 혈당을 측정하고 결과값에 대해 대상자가 이해할 수 있는 수준으로 이야기 할 것이다.

5. 오늘 실습에서 좋았던 점과 아쉬웠던 점은 무엇인가?

오늘 좋았던 점은 선생님과 함께 경로당을 방문하여 혈압을 재고 선생님께서 대상자들과 대화하는 방식을 관찰할 수 있었다는 점이고, 아쉬웠던 점은 없었다.

1. 오늘 실습경험의 의미 있는 점은 무엇인가?

오늘 의미있었던 점은 구강의 날을 맞이하여 보건소에서 주최한 행사에 참여한 것이다. 5세 아이부터 노인까지 다양한 연령대에 맞는 부스에 참여하여 의미있었다.

2. 오늘 실습을 하는 동안 나의 생각과 느낌은 어떠했는가?

~~실습은~~ 구강의 날이라고 해서 구강에 대한 부스만 있는줄 알았는데 진드기, 국가암검진, 혈관건강, 정신건강에 대해 지역 사회 주민들에게 홍보할 수 있어서 좋았다.

3. 오늘 실습을 통해 새롭게 알게 된 것은?

오늘 새롭게 알게된 점은 어렸을 때부터 보건교육하는것이 중요하다고 느꼈다는 것이다. 어린이 대상으로 저금통에 금연스티커를 붙여 금연 저금통을 만드는 부스의 진행을 맡았는데 아이들과 이야기하다 보니 자연스럽게 금연의 중요성을 인식시켜주는 일이고 가족의 건강과도 연결되어 있다고 생각했다.

4. 오늘 실습을 통해 새롭게 배운 것들이 향후 간호실무에 어떻게 적용될 수 있겠는가?

아이들에게 말할 때는 또박또박 천천히 이야기해야 하며, 표정을 잘 관찰하여 아이들의 기분이나 생각을 유추하여 간호할 것이다.

아동간호
[illegible]하게 된 새로운

5. 오늘 실습에서 좋았던 점과 아쉬웠던 점은 무엇인가?

좋았던 점은 행사에 참여하여 국가암검진 치매 홍보, 혈당과 혈압측정, 금연 저금통 만들기 등을 할 수 있었다는 점이고, 아쉬웠던 점은 없었다.

1. 오늘 실습경험의 의미 있는 점은 무엇인가?

오늘 의미있는 점은 구강의 날 행사로 만성질환 관리를 위하여 혈압, 혈당을 재기 위한 어르신들의 질서를 유지하여 대기줄이 혼란스럽지 않게 했다는 점이다.

2. 오늘 실습을 하는 동안 나의 생각과 느낌은 어떠했는가?

행사에 참여하는 동안 많은 어르신들이 자신의 건강관리에 관심을 갖고있음을 느꼈다. 대기줄이 길었음에도 불구하고 혈압과 혈당을 재드리고 선물까지 드린다고 하면 줄을 서는 모습을 관찰할 수 있었다. 줄 서는 동안 자신의 혈당이 높게 나올까봐 걱정하는 어르신께 식사는 언제하셨는지, 당뇨약은 드시는 편인지 여쭈어보고 정확한건 혈당을 잰후 알 수 있으니 걱정마시라고 하였다.

3. 오늘 실습을 통해 새롭게 알게 된 것은?

새롭게 알게된 점은 아직 세상은 따뜻하다는 점이다.
부스 대기줄이 길어서 줄서기 어려운 다리가 불편하신 어르신에게 먼저 자리를 양보하시는 분의 모습을 관찰할 수 있어서 마음이 훈훈해졌다.
나는 그 모습을 보고 뒤에 줄 서있는 분들에게 상황을 설명하고 양해를 구하였다.
평소 어르신들과 이야기할 기회가 별로 없고 양해를 구할 일이 없었는데, 기분 나쁘지 않게 양해를 구하는 의사소통 기술을 익혔다.

4. 오늘 실습을 통해 새롭게 배운 것들이 향후 간호실무에 어떻게 적용될 수 있겠는가?

오늘 배운것을 통해 불가피한 상황이 갑자기 생겼을 때 정중하게 상황을 설명하고 양해를 구할것이다. 자신의 활력징후, 혈당검사의 결과를 궁금해하고 걱정하는 대상자에게 정확한 정보를 전달하고 생활습관 개선을 통해 나아질 수 있을 것이라고 설명할 것이다.

5. 오늘 실습에서 좋았던 점과 아쉬웠던 점은 무엇인가?

좋았던 점은 어르신들과 이야기할 기회가 있다는 것이고,

아쉬웠던 점은 대기줄에 이탈하여 다른 부스에 갔다온 후 뒤에 사람에게 내 자리를 맡겨놨다며 새치기를 하는 사람이 있었다는 점이다.

1. 오늘 실습경험의 의미 있는 점은 무엇인가?

오늘 의미있는 점은 치매안심센터(치매보건소)에서 어떻게 인지선별검사(CIST)를 하는지 봤다는 점이다. ① 시간, 장소 물어보기 ② 문장 듣고 말하기 (민수는 자전거를 타고 공원에서 11시부터 야구를 했다.) ③ 숫자 따라 말하기 (6·9·7·3) ④ 금수강산 거꾸로 말하기 ⑤ 제시된 그림 똑같이 그리기 ⑥ 모양배열의 규칙성 찾기 ⑦ 2번에서 말했던 문장 다시 말하기 ⑧ 물건의 이름 말하기 (3가지) ⑨ 1분동안 과일, 채소 이름을 최대한 많이 말하기 → 10~15분 소요

2. 오늘 실습을 하는 동안 나의 생각과 느낌은 어떠했는가?

협조가 잘 안되는 대상자를 인지선별검사 하는 선생님의 모습을 보고 많이 답답할 것 같다는 생각을 했다. 검사할 때 대상자가 정말 몰라서 대답을 못하는 건지, 대답하기 싫어서 안하는건지 구분하는 것이 정말 어렵다고 느꼈다.

3. 오늘 실습을 통해 새롭게 알게 된 것은?

치매안심센터에서 하는 프로그램에 대해 알아보고 목적과 대상자에 차이점이 있다는 것을 알았다.

1. 치매환자쉼터 : 치매환자 대상 / 인지활동교육 ⊕ 미술치료, 원예치료, 운동치료
2. 치매예방교실 : 치매진단 받지않은 60세 이상 어르신 대상 / 치매 예방위한 인지활동교육
3. 인지강화교실 : 치매선별검사 → 인지저하, 정밀검사 → 경도인지장애로 진단된 어르신 / 인지활동 교육
4. 치매가족지원교실 : 치매환자, 경도인지장애 환자를 돌보는 가족 보호자 대상 / 돌봄교육, 스트레스 해소 프로그램

4. 오늘 실습을 통해 새롭게 배운 것들이 향후 간호실무에 어떻게 적용될 수 있겠는가?

치매안심센터에서 하는 프로그램, 조기검사, 지원 서비스에 대해 알고 필요한 대상자에게 설명할 수 있을 것이다. 인지선별검사를 할 때 설명이 필요한 검사문항을 대상자가 이해할 수 있도록 설명할 수 있을 것이다.

5. 오늘 실습에서 좋았던 점과 아쉬웠던 점은 무엇인가?

좋았던 점은 치매안심센터에서 인지선별검사를 하는 모습과 연계하는 모습을 관찰할 수 있었다는 점이고, 아쉬웠던 점은 없었다.

1. 오늘 실습경험의 의미 있는 점은 무엇인가?

오늘 의미있었던 점은 보건소 예방접종실에서 실습하여 예방접종 접수 → 예진표 작성 → 예진의사상담 → 접종하는 절차를 봤다는 점이다. 또, 폐렴구균 예방접종 예진표 작성을 도와드렸다.

2. 오늘 실습을 하는 동안 나의 생각과 느낌은 어떠했는가?

^국가에서 무료접종을 지원하는 항목은 꼭 보건소에서 맞았으면 좋겠다는 생각을 많은 사람들이 했다. 나는 자궁경부암 예방접종 (가다실)을 무료로 하지 못했는데, 무료접종대상인 경우 시기를 놓치지 않고 접종할 수 있도록 나라에서 많이 홍보를 하면 좋을 것 같다.

3. 오늘 실습을 통해 새롭게 알게 된 것은?

접종간격

1년 ↓ ↑ 1년 (최소 8주)

*23 → 23 : 5년

새롭게 알게된 점은 65세 이상 어르신 대상으로 폐렴구균 예방접종무료사업을 하는데, 백신의 종류가 2가지 있다는 점이다.

① 23가 다당질 백신 : 프로디악스 23가, 뉴모 23가 / 침습성 폐렴구균 감염증 50~80% 예방 / 폐렴예방효과 일관성 × / 항체지속기간 최소 5년

② 13가 단백결합백신 ; 프리베나 13 / 침습성 폐렴구균 감염증 75% 예방 /
↳ 면역저하자는 우선 접종권장 폐렴 45% 예방 | 항체지속기간 최소 5년 (면역원성 우수)

4. 오늘 실습을 통해 새롭게 배운 것들이 향후 간호실무에 어떻게 적용될 수 있겠는가?

국가 예방접종사업을 통해 65세이상 어르신들은 폐렴구균 예방접종을 무료로 할 수 있는 것을 알았으니, 주위에 계신 할머니나 할아버지께 여쭈어보고 백신 접종을 권유할 것이다. 예방접종 예진표 작성방법을 알고 작성에 어려움을 겪는 어르신들을 도와 접수할 수 있도록 안내해드릴 수 있다.

5. 오늘 실습에서 좋았던 점과 아쉬웠던 점은 무엇인가?

오늘 좋았던 점은 예방접종실에서 어떤 업무를 주로 하는지 알게되었다는 점이고, 아쉬웠던 점은 딱히 없다.

1. 오늘 실습경험의 의미 있는 점은 무엇인가?

오늘 실습에서 의미있었던 점은 컨퍼런스를 통해 군산시의 지역사회 문제점을 공유하고 어떻게 해결할지 간호계획과 보건교육계획안을 발표하여 내가 생각하지 못했던 부분을 알게되었다는 점이다.

2. 오늘 실습을 하는 동안 나의 생각과 느낌은 어떠했는가?

보건프로그램을 계획할 때 대상자들이 잘 참여할 수 있도록 준비하는 것이 중요하다는 생각이 들었다. 대상자들의 입장에서 생각해보고 참여도를 올릴 방안을 생각해야겠다고 느꼈다.

3. 오늘 실습을 통해 새롭게 알게 된 것은?

군산시의 암검진률은 매번 감소하는 추세인데, 왜 참여를 안할까 궁금하였다. 건강보험공단에서 암검진 독려 전화를 수십통 해보니 너무 바쁘거나 귀찮아서 안하는 사람도 있지만, 정말 내가 대상자인지 모르고 잊어버렸던 사람들도 있음을 알게되었다.

4. 오늘 실습을 통해 새롭게 배운 것들이 향후 간호실무에 어떻게 적용될 수 있겠는가?

보건 교육의 참여도를 높이려면 창의성을 발휘하여 재밌는 프로그램을 작성하고 적극적이게 활동할 수 있도록 상품을 준비하여 보건교육안을 세울것이다.

5. 오늘 실습에서 좋았던 점과 아쉬웠던 점은 무엇인가?

좋았던 점은 다양한 진단의 간호계획과 보건교육안을 공유하였다는 점이고, 아쉬웠던 점은 ~~컨퍼~~ 컨퍼런스 중간에 장소가 바뀌었다는 점이다.

1. 오늘 실습경험의 의미 있는 점은 무엇인가?

오늘 의미있는점은 국민건강보험공단에서 암 검진 독려 전화를 어제 포함하여 200통 넘게 독려하여 지역사회 건강증진에 기여하였다는 점이다.

2. 오늘 실습을 하는 동안 나의 생각과 느낌은 어떠했는가?

여러 사람에게 전화를 하며 검진받은 병원을 물어봐야할 때 "내가 그걸 왜 알려줘요?" 라며 거부하였을 때 굉장히 당황하였다. 끝내 알려주지 않아 아쉬웠다. 검진 독려 전화를 하는 선생님들도 기분상한일이 있었을 것같다.

3. 오늘 실습을 통해 새롭게 알게 된 것은?

암검진 중에서 특히 간암검진을 독려하는 전화를 했다.
간암의 검진주기, 검사항목, 대상기준을 알게되었다.

- 검진주기 : 6개월주기
- 검사항목 : 간초음파검사, 혈청알파태아단백검사
- 대상 : 40세 이상 간암발생 고위험군 남·여

4. 오늘 실습을 통해 새롭게 배운 것들이 향후 간호실무에 어떻게 적용될 수 있겠는가?

국민건강보험공단 실습을 하며, 많은 사람들이 건강검진을 받지않음을 깨달았다. 그래서 내주변에 있는 어른들께 건강검진 시기를 놓치지 말고 해당되는 항목을 검진받으라고 알렸다.

5. 오늘 실습에서 좋았던 점과 아쉬웠던 점은 무엇인가?

좋았던 점은 지역사회 건강증진에 기여했다는 점이고, 아쉬웠던점은 딱히 없다.

1. 오늘 실습경험의 의미 있는 점은 무엇인가?

지역사회간호학 첫 실습을 나와서 보건교육실에서 각 파트별로 선생님들께서 간단하게 OT를 해주셨다. 첫 날은 구강보건센터로 실습을 갔고, 불소 만들기를 하고 데스크에서 명단 작성을 도와드리며 불소를 나누어 드렸다.

2. 오늘 실습을 하는 동안 나의 생각과 느낌은 어떠했는가?

지역사회간호학 교과목 첫 실습이어서 보건소에서 어떠한 일들을 하게 될지 궁금했는데 생각보다 더 많은 업무들이 이루어지고 있다는 것을 느꼈다. 또한 구강보건센터에서 실습을 하면서 불소 외에도 치아 검진을 받으러 오는 것, 어린이집 구강 관련 물품 보내기 등의 활동을 하고 있다는 것을 알아서 놀랐다.

보건소 인식 1-1

3. 오늘 실습을 통해 새롭게 알게 된 것은?

구강보건센터에서 하는 일은 장애인 및 취약계층 무료진료, 불소용액양치사업, 구강보건교육, 양치체험교실운영, 미취학어린이 구강건강관리사업, 노인의치보철사업, 학교구강보건사업, 어린이충치예방사업, 노인불소도포, 스케일링사업, 사회복지시설 구강건강관리 사업을 실시한다고 한다. 생각했던 것보다 더 다양한 사업들을 실시하고 있으며 불소를 만드는 것 또한 신기하였다.

4. 오늘 실습을 통해 새롭게 배운 것들이 향후 간호실무에 어떻게 적용될 수 있겠는가?

우리가 잘 모르는 사업들이 실시되고 있어서 이것들에 대하여 자세한 정보를 알고 홍보하는 방법이 중요한 것 같다. 적극적인 홍보를 통해 모든 시민들이 보건소를 적극적으로 이용할 수 있도록 해야할 것이다. 이를 통해 시민들은 건강한 생활을 할 수 있게 될 것이며 보건소에 더 많은 관심을 가지게 되는 것이다.

보건소 인식 1-1

5. 오늘 실습에서 좋았던 점과 아쉬웠던 점은 무엇인가?

직접 불소를 통에 담아서 [illegible] 수 있는 기회가 있어서 좋았다. 초등학생 때를 마지막으로 불소를 본 기억이 없었는데 구강보건센터에서 실습을 통하여 직접 불소를 볼 수 있고 시민들을 응대해 드릴 수 있어서 좋은 경험이 되었다.

P1) 3-3 협력

1. 오늘 실습경험의 의미 있는 점은 무엇인가?

오늘 실습을 나가게 된 부서는 건강증진실이다. 이곳은 간호사 선생님, 운동 처방사 선생님, 영양사 선생님, 코디네이터 선생님이 한 팀이 되어서 부서를 운영해 나가신다. 시민들의 체중 조절을 위해 다양한 프로그램과 사업을 진행하시는 부서이다.

2. 오늘 실습을 하는 동안 나의 생각과 느낌은 어떠했는가?

간호사 선생님 뿐만이 아니라 다양한 직종이 모여서 한 팀을 이루어 부서를 운영한다는 것이 신기하기도 하였고 흥미로운 곳이었다고 느꼈다. 시민들의 건강을 위해서 체중과 관련한 다양한 프로그램을 운영하고 직접 검사, 상담 등을 통해서 교육을 제공해드리는 모습을 볼 수 있었다.

3. 오늘 실습을 통해 새롭게 알게 된 것은?

군산시 보건소 건강증진실에서는 시민들에게 보다 나은 건강생활실천 서비스를 드리고자 운영되고 있으며 체성분 검사(인바디 검사)를 진행해 드리며 다이어트식품, 체육운동시설 등 상업적인 목적으로 인한 체성분 검사는 금지되고 있다는 점이다. 또한 월수금 오전과 오후로 나누어서 회원분들을 대상으로 운동 프로그램을 진행하고 있으며, 모바일 헬스케어 사업을 통하여 온라인으로 회원분들을 관리해드리고 있다.

4. 오늘 실습을 통해 새롭게 배운 것들이 향후 간호실무에 어떻게 적용될 수 있겠는가?

각자 맡은 부서에서 시민들의 건강을 위하여 실행에 옮길 수 있는 프로그램을 계획하는 과정부터 수행하는 과정까지 모두 우리의 몫이기 때문에 이러한 프로그램을 잘 계획하는 아이디어 또한 중요하다. 시민들의 가장 큰 문제점부터 파악하여 적절한 프로그램 & 사업을 내세워서 모두의 건강을 향상 시킬 수 있도록 해야 할 것이다.

5. 오늘 실습에서 좋았던 점과 아쉬웠던 점은 무엇인가?

수요일이라 운동 프로그램이 있었는데 함께 참여하지 못한 부분이 많이 아쉬웠다. 또한 계속 앉아만 있어서 이 부서에서 하는 일들에 대하여 더 많이 알아볼 수 없었음에 아쉬움이 남는다.

1. 오늘 실습경험의 의미 있는 점은 무엇인가?

건강보험공단 [illegible]지사에 방문하여 실습을 할 수 있었다. 내가 맡게 된 역할은 간암 건강검진 대상자 분들께 전화를 드려서 검진을 독려하는 역할이었다. 다양한 나이대의 대상자분들께 전화를 드려서 검진을 받지 않으셨으면 가까운 병원을 알려드리기도 하고, 검진을 받으신 분들이 계신다면 언제, 어디서 받았는지 질문을 하여 조사를 하였다.

2. 오늘 실습을 하는 동안 나의 생각과 느낌은 어떠했는가?

건강보험공단에서 어떤 일들이 이루어지는지 잘 알지 못하였는데 이번 실습을 통하여 각 부서가 다양하게 나뉘어 있다는 것과 각 부서별로 하는 업무들이 다양한 것을 보니까 신기하고 이론에서 배웠던 단어들을 직접 보게 되니까 반갑고 신기하였다.

3. 오늘 실습을 통해 새롭게 알게 된 것은?

오늘 내가 직접 전화를 통해 검진을 독려한 간암 검진은 6개월 간격으로 상반기와 하반기로 나뉘어 검사가 진행된다는 것을 알게 되었다. 그래서 총 1년에 2번을 실시하게 된다. 또한 6대 암 검진이라고 해서 위암, 간암, 유방암, 대장암, 폐암, 자궁경부암이 있고 각각 검진 주기가 다르다. 위암 = 2년 주기, 간암 = 6개월 주기, 유방암 = 2년 주기, 대장암 = 1년 주기, 폐암 = 2년 주기, 자궁경부암 2년 주기.

4. 오늘 실습을 통해 새롭게 배운 것들이 향후 간호실무에 어떻게 적용될 수 있겠는가?

각각의 암 검진의 주기와 암 별로 어떤 검사가 진행되는지 아는 것도 중요하다고 생각한다. 이런 것을 숙지하여 환자분들의 건강을 위하여 독려할 수 있으며 정확한 정보를 전달할 수 있는 간호사가 될 수 있을 것이다.

5. 오늘 실습에서 좋았던 점과 아쉬웠던 점은 무엇인가?

건강보험공단에 방문하여 직접 간암 검진 대상자분들께 연락을 드리며 검진을 독려해드리는 경험을 해볼 수 있어서 좋았고, 이곳에 또 언제 올 수 있을지 모르겠지만 실습을 통해서 직접 건강보험공단을 짧게 나마 경험해볼 수 있어서 좋았다.

1. 오늘 실습경험의 의미 있는 점은 무엇인가?

건강보험공단에서의 마지막 실습 날이었다. 오늘도 어제와 같이 간암 건강검진 대상자분들께 전화를 드려 검진을 독려하는 일을 하였다. 전화를 드리며 최대한 검진을 받을 수 있도록 이끌며 간암 검진 주기, 검사 방법 등에 대하여 설명을 드리는 역할을 하였다.

2. 오늘 실습을 하는 동안 나의 생각과 느낌은 어떠했는가?

전화를 통해서 간암 건강검진 대상자분들과 대화를 나누어보면 생각보다 암 건강검진에 대하여 잘 알고 있다고 느꼈다. 또한 간암 건강검진 주기가 짧아서 독려를 해드릴때 우리 부모님은 해봤나?라는 생각이 많이 들었다. 건강보험공단에서 실습이 끝나고서 부모님, 할머니, 할아버지께도 간암 건강검진을 받으셨냐고 여쭤봐야겠다. 라고 생각하였다. 그냥 전화만 하는 것이 아니라 되게 여러가지 많은 생각이 들게된 실습이었다.

3. 오늘 실습을 통해 새롭게 알게 된 것은?

위암은 위내시경검사(유소견 시 조직검사), 위장조영 검사로, 간암은 간초음파 검사, 혈청알파태아단백검사로 유방암은 유방 촬영(양측)검사로, 대장암은 분변 잠혈 검사 (반응 있을 시 대장내시경 검사 → 유소견시 조직검사)로, 폐암은 저선량흉부 CT, 사후결과상담, 자궁경부암은 자궁경부세포검사로 건강검진을 실시한다는 것을 새롭게 알게 되었다. 이는 6대 암 검진에 해당하는 사항이다.

4. 오늘 실습을 통해 새롭게 배운 것들이 향후 간호실무에 어떻게 적용될 수 있겠는가?

대상자들에게 암 검진을 독려해드릴 수 있으며, 부모님, 가족, 주변지인들을 더 챙겨줄 수 있을 것이다. 또한 6대 암검진에 대하여 정확히 인지하고 있어 질문을 할 때에도 적절한 답변을 해드리며 도움을 드릴 수 있을 것이다.

5. 오늘 실습에서 좋았던 점과 아쉬웠던 점은 무엇인가?

마지막 날까지 간암 건강검진 대상자분들께 검진을 독려해드릴 수 있어서 보람을 느끼게 되어서 좋았다. 아쉬운 점을 굳이 말하자면 컨퍼런스로 인하여 오전까지만 실습을 해야 해서 아쉬웠다.

1. 오늘 실습경험의 의미 있는 점은 무엇인가?

오늘은 예방접종실에서 실습이 이루어졌다. 오시는 민원인들께 예방접종 예진표 작성을 도와드리고 체온을 측정해드린 뒤 작성한 예진표를 선생님께 드리는 일이다. 또한 어떠한 예방접종을 맞는지에 따라 적절한 예진표를 드린다. 연세가 있으신 분들일 경우에는 옆에서 읽어드리며 하나하나씩 천천히 도움을 드렸다.

2. 오늘 실습을 하는 동안 나의 생각과 느낌은 어떠했는가?

예방접종실에서 했던 일은 이전에 코로나 19 예방접종을 해드렸던 경험이 있어서 어렵지 않게 할 수 있었다. 생각보다 보건소에 다양한 연령대의 민원인들이 방문하시고, 보건소를 잘 이용해서서 놀랐다. 또한 다양한 예방접종에 관한 팜플렛을 보며 알지 못했던 내용과, 알았지만 더 자세하게 학습할 수 있어서 좋았다.

3. 오늘 실습을 통해 새롭게 알게 된 것은?

BCG 예방 접종은 결핵 예방접종이며 피내용과 경피용이 있다. 둘은 접종방법과 특징이 다른데 우선 BCG 피내 접종형은 피부에 약 15도로 바늘사면을 완전히 삽입한 후 백신을 주입하며 BCG 접종량이 상대적으로 일정하고 정확하다. 이에 비해서 BCG 경피접종형은 피부에 주사액을 바른 후 9개 바늘을 가진 주사도구를 이용하여 두 번에 걸쳐 강하게 눌러 접종하고, 접종량이 일정하지 않을 수 있다는 특징이 있다.

4. 오늘 실습을 통해 새롭게 배운 것들이 향후 간호실무에 어떻게 적용될 수 있겠는가?

각 연령에 해당하는 예방접종과, 접종 횟수, 접종 간격, 접종 적정 나이 등을 정확하게 파악하고 매년 표준예방접종 일정표를 파악하는 게 중요하다. 2022년과 달리 2023년에는 2개월, 4개월, 6개월에 로타 바이러스 감염증 예방접종이 추가되었다. 이러한 사항들을 정확히 파악하여 적정 나이에 원활히 예방접종이 이루어질 수 있도록 해야겠다.

5. 오늘 실습에서 좋았던 점과 아쉬웠던 점은 무엇인가?

BCG 예방접종에 대한 내용을 더 자세히 알 수 있어서 좋았으며, 민원인들이 오시면 예진표 작성을 도와드릴 수 있어서 좋은 경험이 되었다고 생각한다.

1. 오늘 실습경험의 의미 있는 점은 무엇인가?

방문간호실에 가서 방문 간호사 선생님들과 함께 지역사회로 나가서 직접 대상자분들을 만나 뵙고 직접 BST. BP를 측정해드리는 경험을 하였다. 오전에는 가정으로 직접 방문하였고, 오후에는 경로당으로 방문을 하여 다양한 대상자분들을 만나고 왔다.

2. 오늘 실습을 하는 동안 나의 생각과 느낌은 어떠했는가?

이론에서 배웠던 것처럼 정말 지역사회 각 구역을 방문하여 건강 관련하여 상담을 해주시고 BST와 BP를 측정해드리면서 이전 결과와 비교하여 맞춤형 건강 교육을 해드리는 부분이 신기하였다. 또한 대상자분들께 교육을 해드리는 것을 옆에서 같이 들을 수 있고, 이를 통해 이론을 다시 한 번 더 상기 시켜볼 수 있는 기회가 있어서 좋은 경험이 되었다.

3. 오늘 실습을 통해 새롭게 알게 된 것은?

지역사회 방문간호사 선생님들은 각각 해당 맡으신 구역이 있으며 매번 방문하는 것이 아니라 1달에 1번 정도 방문을 하신다는 것이다. 또한 각 대상자마다 어떠한 질환이 있으신지 모두 기록을 통하여 변화 양상들을 질문하고 적절한 상담을 자세하게 제공해주셨던 부분이 놀라웠다. 또한 방문 간호사 선생님께서는 요양 등급 판정되신 분들을 대상으로 간호를 제공해드리는 게 아니라는 것이다.

4. 오늘 실습을 통해 새롭게 배운 것들이 향후 간호실무에 어떻게 적용될 수 있겠는가?

지역사회 방문 간호사 역시 나의 대상자들의 질환 등을 정확히 파악하고 있어야 하고 건강 상담과 교육 또한 개별 맞춤형으로 제공해드려야 하기 때문에 제공해드리는 간호와 대상자들의 질환에 대하여 정확히 인지하고 있어야 한다. 그러면 향후 지역사회의 방문간호사가 될 수 있다면 대상자분들을 더 잘 관리해드릴 수 있으며 건강 증진을 도모해드릴 수 있을 것이다.

5. 오늘 실습에서 좋았던 점과 아쉬웠던 점은 무엇인가?

보건소 안에서가 아닌 직접 대상자 분들이 있는 곳으로 출장(?)을 나가게 되어서 좋았고, 그 분들에게 직접 BST와 BP를 측정해드릴 수 있어서 좋은 경험이 되었다. 내가 몰랐을 뿐이지 이렇게 지역사회 대상자분들에게도 지속적으로 관리가 이루어지고 있어서 좋았다.

1. 오늘 실습경험의 의미 있는 점은 무엇인가?

오늘의 실습 부서는 이동진료팀이었다. 그런데 선생님께서 오늘은 이동진료 업무가 없는 날이라고 하시며 수유실에서 각자 자습을 하면된다고 하셔서 선생님들과 떨어져서 자습을 하며, 선생님들이 하시는 일들을 볼 수 조차도 없었던 굉장히 아쉬움이 가득한 날이었다.

2. 오늘 실습을 하는 동안 나의 생각과 느낌은 어떠했는가?

처음에 이동진료팀에서는 어떤 경험들을 하게 될까 하며 기대를 잔뜩 가지고 부서 안으로 들어갔다. 하지만 나의 기대와는 다르게 오늘 이동진료 업무가 없다고 말씀하셔서 되게 아쉬움과 무상함이 공존해 있었던 것 같다. 이동진료팀에서는 어떤 일들이 이루어지는지 경험해보고 싶고 궁금한 것들이 많이 있었는데 경험의 기회가 없던 부분이 제일 아쉬웠다.

3. 오늘 실습을 통해 새롭게 알게 된 것은?

직접 경험을 해보지 못하여서 군산시 보건소 홈페이지에서 찾아본 이동진료팀은 의료취약지 경로당 어르신들을 대상으로 가정간강검진, 보건교육을 찾아가는 의료서비스로 진행하고 있다. 또한 경로당별 담당 주치의제를 운영하고 있다. 다른 사업(?)으로는 말도, 방축도, 명도, 관리도, 두리도 주민을 대상으로 가정검진, 보건교육, 내과진료, 안전상비약 지급 및 복약지도 등, 한방진료가 이루어지고 있다.

4. 오늘 실습을 통해 새롭게 배운 것들이 향후 간호실무에 어떻게 적용될 수 있겠는가?

직접 이동진료를 경험해보진 못하였지만 의료취약지 경로당 어르신들을 찾아내는 것 또한 이 부서에서 해야하는 업무인 것 같다. 그러니 지역사회에 관심을 가지고 대상자분들을 적절히 선정을 해내는 능력도 필요한 것 같다. 향후 지역사회 간호사가 될 수 있다면 나의 지역사회에 관심을 가지고 적절한 보건서비스를 제공 받을 수 있도록 해야겠다.

5. 오늘 실습에서 좋았던 점과 아쉬웠던 점은 무엇인가?

이동진료팀 업무를 직접 관찰하고, 경험해보고 싶었는데 딱 오늘 이동진료 업무가 없어서 직접 경험해볼 수 없었던 부분이 가장 큰 아쉬움으로 남는 것 같다.

1. 오늘 실습경험의 의미 있는 점은 무엇인가?

오늘의 실습 부서는 접수실이었다. 그런데 갑작스럽게 방사선실로 실습을 가게 되었다. 방사선 실에서는 민원인분들이 오시면 탈의실로 안내를 해드리며 목걸이, 속옷 등을 탈의 해주시고 가운으로 갈아입고 나오라고 안내를 해드리는 일을 할 수 있었다.

2. 오늘 실습을 하는 동안 나의 생각과 느낌은 어떠했는가?

방사선 실에서 실습을 해볼 수 있는 기회가 흔치 않아서 이번 실습을 통하여 좋은 경험을 해볼 수 있었다. 또한 민원인 분들께 안내를 해드릴 수 있었으며 촬영 시 속옷이나 금속류 등이 나올 수 있기 때문에 모두 탈의를 해드리라고 설명을 드리는 과정에서 나 또한 한 번씩 더 주의사항을 상기할 수 있게 되어서 좋았다.

3. 오늘 실습을 통해 새롭게 알게 된 것은?

[illegible]

촬영 시 목걸이, 속옷을 탈의하는 것은 알고 있었던 사실인데 단추가 있는 것들은 상관없다는 것이다. 또한 단추가 없어도 옷의 마크가 나온다고도 하는데 정확한 기준이 있는 것 같지는 않았다. 젊은 여성분들이 오시면 항상 임신 가능성 여부와 임신 여부를 확인 하시고 촬영을 한다. 방사선에 노출될 수 있는 환경이기 때문에 촬영 전 꼭 확인을 한다. 또한 방사선이 있는데 선생님께서 가지고 계신 방사선 측정기로 얼만큼 피폭을 받고 있는지 알 수 있다고 하셨다.

4. 오늘 실습을 통해 새롭게 배운 것들이 향후 간호실무에 어떻게 적용될 수 있겠는가?

내가 방사선 촬영을 직접 해드리는 것은 아니지만 환자분들께서 방사선 촬영에 대하여 여쭤보거나 교육이 필요하다면 오늘 민원인분들께 안내를 드린 것과 같이 정보를 제공해 드릴 수 있을 것이다. 방사선실에 대하여 조금 더 정확히 공부를 해야겠지만 지금은 간단한 안내는 충분히 드릴 수 있을 것 같다.

5. 오늘 실습에서 좋았던 점과 아쉬웠던 점은 무엇인가?

흔치 않은 기회로 방사선실에서 직접 실습을 해볼 수 있어서 좋았지만, 원래 배치 부서인 접수실에서 실습을 해보지 못한 부분이 아쉬웠던 것 같다. 그래도 방사선실에 대하여 직접 경험해보고 학습해볼 수 있는 기회가 있어서 뜻깊은 실습이 되었다.

1. 오늘 실습경험의 의미 있는 점은 무엇인가?

보건소에 처음 실습을 나오게 되어 보건소의 구조, 하는 역할 등에 대해 알게 되었고 치매안심센터에서 어떤 일을 하는지 치매가 무엇인지에 대해 알게 되어 의미있었습니다.

2. 오늘 실습을 하는 동안 나의 생각과 느낌은 어떠했는가?

국민건강보험에서 국가 6대 암 검진 대상자를 한명 한명 list를 뽑아서 전화를 돌린다는 것을 처음 알았으며 조기검진을 실시하여 암을 예방해야겠다는 생각이 들었습니다.

3. 오늘 실습을 통해 새롭게 알게 된 것은?

국가 6대 암 검진 중 유일하게 1년에 두번 검진하는 간암 검진이 있다. 간암 검진은 40세 이상 간암발생 고위험군으로 간경변증, B형 간염 바이러스 항원 양성, C형 간염 바이러스 항체 양성, B형 또는 C형 바이러스에 의한 만성 간질환 환자가 대상이다. 검진 비용은 국민건강보험공단이 90%, 수검자가 10% 부담한다. 간초음파검사와 혈청알파태아검사를 시행한다.

4. 오늘 실습을 통해 새롭게 배운 것들이 향후 간호실무에 어떻게 적용될 수 있겠는가?

간암 검진 대상자인 간암발생 고위험군에게 암검진 유선전화를 통해 상반기 검진을 시행하도록 독려해야 한다. 이미 검진을 받았거나 간질환으로 입원 치료 중이거나 타질환으로 입원 중인 경우에는 독려 제외를 처리해 두어야 한다.

5. 오늘 실습에서 좋았던 점과 아쉬웠던 점은 무엇인가?

지역사회간호학실습 중 관내 보건소에서 실습하게 되어서 좋았으며 국민건강보험공단에서 어떤 업무를 하는지에 대해서 알 수 있어서 좋았습니다.

1. 오늘 실습경험의 의미 있는 점은 무엇인가?

건강보험공단 신규직원 채용 공고에 대해 보게 되었는데 간호사 면허증이 있는 경우 건강직과 요양직에 지원할 수 있는 응시자격이 있다는 걸 새롭게 알게 되어 의미있었다.

2. 오늘 실습을 하는 동안 나의 생각과 느낌은 어떠했는가?

건강보험공단에 취업하고 싶을 경우 건강보험 청년인턴경력자 라는 우대사항이 있으며 우대면허(자격증)인 한국사 능력 검정시험 3급이상, 컴퓨터활용능력1급 등이 있다. 간호사라는 직업이 병원에서만 근무하는 것이 아니라 다양한 업무환경에서 다양한 직종으로 일할 수 있다는 것을 다시 한번 느끼게 되었다.

3. 오늘 실습을 통해 새롭게 알게 된 것은?

자궁경부암 검진은 20세 이상 여성 대상으로 2년마다 시행하며 출생연도 짝·홀수 기준으로 적용한다. 자궁적출술을 받았거나 성경험이 없으신 분은 검사 전에 반드시 검진의사와 상담을 해야 한다.

유방암 검진은 40세 이상 여성으로 유방촬영검사를 시행하며 무증상 조기 유방암을 발견하는 가장 기본적인 검사입니다.

4. 오늘 실습을 통해 새롭게 배운 것들이 향후 간호실무에 어떻게 적용될 수 있겠는가?

자궁경부암 검진 대상자에게 최소 검사 48시간 전부터는 질 내 어떠한 물질도 넣지 않아야 하며 성관계 금지, 탐폰 사용 금지, 질세척 금지, 질 내 약물 및 윤활제 사용 금지, 질 내 피임약 사용 금지 등에 대해 설명해야 한다. 유방촬영검사는 유방 압박으로 인한 고통이 수반될 수 있음을 설명해야 한다.

5. 오늘 실습에서 좋았던 점과 아쉬웠던 점은 무엇인가?

건강보험공단에 취업할 수 있는 요건과 우대사항, 우대면허 등에 대해 알게 되어서 좋았습니다.

1. 오늘 실습경험의 의미 있는 점은 무엇인가?

보건소에서 진행하는 캠페인 도와 혈압, 혈당을 재고 줄 안내등을 하여 의미있었으며 캠페인에 참여하여 체험할 수 있어서 의미 있었습니다.

2. 오늘 실습을 하는 동안 나의 생각과 느낌은 어떠했는가?

5월 31일 세계 금연의 날로 금연에 관련하여 캠페인으로 하고 참여시 선물을 나눠준다. 또 구강보건에서 구강교육을 실시하는데 어린이집이나 유치원에서 원아들을 데리고 와서 체험하는 것을 관찰하였다. 어린이들과 동네 주민들에게 캠페인 참여를 통해 건강증진을 할 수 있는 좋은 기회라고 생각을 하게 되었다.

3. 오늘 실습을 통해 새롭게 알게 된 것은?

진드기매개감염병이란 세균이나 바이러스에 감염된 진드기에 물려 발생하는 감염병으로 SFTS, 쯔쯔가무시증, 라임병 등이 있습니다. 최근 5년간 연평균 진드기매개 감염병 환자 233명 중 40명이 사망해 치명률이 17.2% 입니다. 진드기에 물릴 경우 2주 이내에 고열, 구토, 설사, 혈소판 감소 등의 현상이 나타나는데 심한 경우 사망에 이를 수 있다.

4. 오늘 실습을 통해 새롭게 배운 것들이 향후 간호실무에 어떻게 적용될 수 있겠는가?

예방백신과 치료제가 없기 때문에 예방법을 숙지하여 진드기 물림을 예방하는 것이 무엇보다 중요하고 증상이 나타나면 반드시 의료기관을 방문하도록 지도해야 한다. 피부노출을 최소화하고 진드기 기피제 사용, 풀밭, 풀숲 피하기, 귀가 후 씻고 빨래하기 등을 교육한다.

5. 오늘 실습에서 좋았던 점과 아쉬웠던 점은 무엇인가?

보건소에서 진행하는 행사를 도와드릴 수 있어서 좋았으며 군산시민에게 참여할 수 있도록 유도해 보는 기회가 되어 좋았습니다.

1. 오늘 실습경험의 의미 있는 점은 무엇인가?

금연캠페인 부스에서 유치원생들이 금연스티커를 저금통에 붙이는 것을 도와주며 금연을 해야하는 이유에 대해서 설명할 수 있어서 의미 있었다.

2. 오늘 실습을 하는 동안 나의 생각과 느낌은 어떠했는가?

2일동안 캠페인을 참여하면서 사람들이 부스에서 나눠주는 선물만 받아가려는 분들이 눈에 많이 띄었다. 건강증진을 위해서 하는거지만 사람들은 선물에 관심을 가졌다. 과연 이런 캠페인을 통해서 사람들이 어떤 캠페인을 하고 있는건 알고 있는건지에 대해 의문점이 들었다.

3. 오늘 실습을 통해 새롭게 알게 된 것은?

흡연자의 자녀는 폐 기능이 저하되어 성인기에 만성 호흡기 질환의 형태로 지속적인 영향을 받으며 간접흡연에 노출된 학령기 아동(8-13세)은 폐로 가는 기도에 염증이 생겨 천식 발생 위험이 있습니다. 2세 미만의 영아는 중이 질환에 걸릴 수 있으며 청력장애 및 청력 상실로 이어질 수 있기 때문에 금연을 해야 한다.

4. 오늘 실습을 통해 새롭게 배운 것들이 향후 간호실무에 어떻게 적용될 수 있겠는가?

아이들과 함께 있을 때 흡연자가 있는 장소를 피하고 금연구역을 이용하며 흡연의 위험성에 대해 알려준다. 아이들과 함께 하는 시간이 많은 어른이라면 금연을 시행해야 함을 교육한다.

5. 오늘 실습에서 좋았던 점과 아쉬웠던 점은 무엇인가?

금연저금통을 만드는 것을 도와줄 때 어린아이들이 "금연안돼" "담배는 나쁜거야" 이런 이야기를 들어서 금연의 조기교육이 중요하다는 것을 느끼게 되어 좋았습니다.

1. 오늘 실습경험의 의미 있는 점은 무엇인가?

방문건강관리실에서 실습을 하여 암환자에게 영양제를 드리고 집단시설(경로당)에 방문하여 혈당측정 및 혈압측정을 하고 건강체조를 해볼 수 있어 의미있었다.

2. 오늘 실습을 하는 동안 나의 생각과 느낌은 어떠했는가?

방문간호 시 위험상황이 발생할 수 있기 때문에 항시 간호사 자신의 안전을 지키기 위해 문 앞에 앉아야 되겠다는 생각이 들었고 어르신들이 일주일에 한번씩 하는 건강체조 시 흥미를 유발할 수 있는 음악을 틀어 놓고 하면 더 좋을 것 같다는 생각을 가지게 되었다.

3. 오늘 실습을 통해 새롭게 알게 된 것은?

맞춤형 방문관리란 건강 서비스 이용이 어려운 사회·문화·경제적 건강 취약계층을 대상으로 찾아가는 맞춤형 건강관리 서비스 제공 및 보건소 내외 자원과의 연계를 통하여 대상자의 건강상태 유지 및 수준 향상에 기여하는 사업이다.

4. 오늘 실습을 통해 새롭게 배운 것들이 향후 간호실무에 어떻게 적용될 수 있겠는가?

집단시설 건강관리는 영종 관내 경로 이용 어르신들에게 집단시설(경로당)에 가서 기초검진 및 보건교육을 실시한다. 취약가구 방문관리는 영종 기초생활수급자 및 65세 이상 독거노인, 75세이상 노인부부 대상으로 방문간호사가 직접 가정에 방문하여 서비스를 제공한다.

5. 오늘 실습에서 좋았던 점과 아쉬웠던 점은 무엇인가?

방문간호로 가서 어르신들 혈당을 측정해드리고 방문간호 시 어떤 것들을 하는지에 대해서 알게 되어 좋았습니다

1. 오늘 실습경험의 의미 있는 점은 무엇인가?

접수실 실습을 하면서 보건소 방문하는 사람들에게 진료실이나 건강관리과, 적성검사실 등에 대한 민원 안내를 해드릴 수 있는 기회가 있어 의미있었다.

2. 오늘 실습을 하는 동안 나의 생각과 느낌은 어떠했는가?

보건소에 방문한 사람들 중 보건증을 만들기 위해 오는 분들이 많았으며 치매상담과 자동차 적성검사를 하러 오는 분들도 많이 있었다. 사전연명의료의향서를 작성하러 오시는 분들도 있어서 보건소에서 다양한 업무를 하고 있다는 다시 한번 느끼게 되었다.

3. 오늘 실습을 통해 새롭게 알게 된 것은?

보건증이란 식품 · 유흥업 종사자의 경우 식약처 · 복지부 등 법령에 따라 건강진단을 실시하고 있으며, 건강진단의무이행을 위해 보건소 등에서 건강진단 실시 후 발급한다. 검사항목으로는 전염성 피부질환 검사(양손을 앞뒤로 보여주기), 장티푸스 검사, 폐결핵 3가지 검사를 시행한다.

4. 오늘 실습을 통해 새롭게 배운 것들이 향후 간호실무에 어떻게 적용될 수 있겠는가?

검사 실시 후 결과 판정까지 약 5일 내외 소요됨을 설명해야 한다. 발급은 검사한 보건소 방문하여 신분증 제시 후 수령하거나 공공보건포털 사이트에 접속하여 온라인에서도 발급이 가능함을 설명한다.

5. 오늘 실습에서 좋았던 점과 아쉬웠던 점은 무엇인가?

내원객들의 민원에 대해 안내해 드릴 수 있는 기회가 되어서 좋았습니다.

1. 오늘 실습경험의 의미 있는 점은 무엇인가?

예방접종을 하러 온 대상자들에게 체온을 측정하고 간호사 선생님들께서 주의사항을 설명해준 뒤 예방접종을 실시하는 것을 보게 되었고 보건소에서 진행하는 예방접종 사업에 대해 알게되었다. 투석환자는 의사처방에 따라 B형간염예방접종 용량과 횟수가 정해져 투석환자이다 다르는것을 알게 되어 의미있었다.

2. 오늘 실습을 하는 동안 나의 생각과 느낌은 어떠했는가?

A형간염, B형간염, 어르신들 폐렴구균을 많이 맞으러 오시는 걸 볼 수 있었으며 폐렴구균이 2가지 종류가 있는데 한개는 보건소에서 65세이상 어르신들에게 놔주는 것이며 나머지는 병원에서 사비로 맞아야 한다. 여러가지 감염병을 예방하기 위해 국가에서 시행하는 예방접종은 챙겨서 맞는 것이 좋은 것 같다는 생각이 들었다.

3. 오늘 실습을 통해 새롭게 알게 된 것은?

신증후군출혈열이란 고열, 신부전, 출혈 등을 특징으로 하는 급성 열성 질환으로 아시아와 유럽에 존재하는 여러 종류의 한타바이러스에 의한 전신 감염 질환이다. 예방접종을 통해 신증후군출혈열을 예방할 수 있으며 고위험군에게 우선 접종하도록 권장되며 한달 간격으로 2회 접종하고 12개월 뒤에 1회 접종(0, 1, 13개월 일정으로 3회에 걸쳐 접종)을 시행한다. B형 간염 항체를 맞은지 10년 넘은 사람들에게 항체(N)한 경우 1차를 다시 맞은 후 기억세포가 살아날 수 있기 때문에 항체검사 시행하라고 권고한다.

4. 오늘 실습을 통해 새롭게 배운 것들이 향후 간호실무에 어떻게 적용될 수 있겠는가?

예방접종 후에 생길 수 있는 이상반응은 대개 경미하며, 일상생활에 지장을 줄 정도는 아니며 국소이상반응으로 접종 후 가려움증, 색소침착, 발적, 통증, 종창 등이 있으며, 전신반응으로 발열, 권태감, 구역질 등이 있다고 교육해준다. 신증후군출혈열 예방할 수 있기 때문에 고위험군에게는 예방을 위해 접종을 권고해야 한다.

5. 오늘 실습에서 좋았던 점과 아쉬웠던 점은 무엇인가?

선생님들이 A형간염, B형간염 등 예방접종 하는 것을 직접 관찰할 수 있어서 좋았으며 영유아 예방접종을 보지못해 아쉬웠습니다.

1. 오늘 실습경험의 의미 있는 점은 무엇인가?

구강보건센터에서 실습하는 날인 오늘이 구강의 날이여서 실습을 하게 되어 의미 있었고 불소양치용액을 방문객들에게 나누어 드리며 하는 방법에 대해 설명해 드려 의미있었습니다.

2. 오늘 실습을 하는 동안 나의 생각과 느낌은 어떠했는가?

구강보건센터에서 하는 일은 노인의치보철사업, 학교구강보건사업, 어린이충치예방사업, 노인불소도포, 스케일링사업, 사회복지시설 구강건강관리사업, 장애인 및 취약계층 무료진료, 불소용액양치사업, 구강보건교육, 양치체험교실 운영, 미취학어린이 구강 건강관리 사업 등의 다양한 일을 한다. 그래서 인지 구강보건센터안에는 치과의자, 양치실, 교육실 등 다양한 시설이 구비되어 있어서 많은 시민들이 알고 방문하였으면 좋을 것 같다는 생각이 들었다.

3. 오늘 실습을 통해 새롭게 알게 된 것은?

치아홈메우기란 치아의 음식을 씹는 부위에는 좁고 깊은 홈이 있는데 이부위는 음식물이 잘끼고 쉽게 빠져나가지 않으며 칫솔질을 해도 충분히 제거가 되지 않아 세균의 서식처가 되어 충치가 잘 발생하고 특히 충치가 처음 시작되는 부위이다. 치아의 홈에 충치가 발생하기 전에 플라스틱과 유사한 치과용 재료로 미리 막아 음식물이 끼지 않고 세균이 자라지 않게 하는 방법이다. 올바른 칫솔질과 치아홈메우기를 통해 씹는면 충치의 약 65-90% 예방 효과가 있는 것으로 나타나고 있다.

4. 오늘 실습을 통해 새롭게 배운 것들이 향후 간호실무에 어떻게 적용될 수 있겠는가?

영구치가 나오기 시작할 때 시행하는 것이 가장 효과적이며 주대상 연령층은 5세 경부터 15세 무렵(영구치열이 완성되고 1~2년이 지난 후) 까지 이다. 시행 후에는 정기적으로 치아 홈 메우기 상태를 검사하는 것이 좋으며 어린이는 6개월에 한번 이상 치과에 방문하여 구강검사를 받아야 함을 보호자에게 안내해야 한다.

5. 오늘 실습에서 좋았던 점과 아쉬웠던 점은 무엇인가?

구강보건센터에서 다양한 업무를 하는 것에 대해 알게 되어 좋았으며 어린아이들을 대상으로 양치의 중요성이나 치과의 무서움을 줄일 수 있는 다양한 동화책들이 있다는 걸 알게 되어 좋았습니다.

1. 오늘 실습경험의 의미 있는 점은 무엇인가?

오늘 보건소에 첫날 실습하러 왔을때 OT로 치매예방. 매개병에 관한 주제로 다양하게 알려주시면서 어떤 일을 하는곳인지 목적을 정확히 알려주셔서 좋았다.

진료실로 부서로 배정받아서 실습을 하게되었는데 어떻게 일을 하는곳인지 알수있어서 의미있었다.

2. 오늘 실습을 하는 동안 나의 생각과 느낌은 어떠했는가?

오늘은 진료실에만 있어서 전체 보건소에서 하는일을 다 볼수는 없었지만. 그래도 오늘 진료실에서 환자분들이 오면 차트에 체온을 측정한 후 혈압을 재고, 당이 있으신 분들은 혈당검사, 콜레스테롤 검사를 하고 의사 진료를 받으시고 약처방을 받고 귀가하시는것을 볼수 있었다. 보건소에서는 진료받고 약처방 받은적이없었는데 많은분들이 받으시는걸 보고 다시 알게되었다.

3. 오늘 실습을 통해 새롭게 알게 된 것은?

건강보험공단에서 간호사가 어떤일을 하는지 알려주셨다.

건강직은 국민을 대상으로 건강관리 사업을 기획 및 추진 주업무) 공단의 건강검진 사업 및 사후관리 업무 외에, 건강증진 서비스 관련업무, 만성질환 관리 사업 관련 업무 등을 한다.

요양직은 장기요양 급여 비용심사, 방문조사 등 장기요양 업무를 하고 주업무는 장기요양보험 신청자의 면담 및 등급판정 지원업무, 고령의 노인들에게 신체활동 및 가사지원 등의 장기요양급여제공을 한다.

4. 오늘 실습을 통해 새롭게 배운 것들이 향후 간호실무에 어떻게 적용될 수 있겠는가?

오늘 OT로 인해 새로운것을 배우고 알게되서 좋았고 향후에 취업을 하거나 간호사로서 도움이 될것이라고 생각하였다. 그리고 진료실에서도 어떤방식으로 진행되는지 알게되어서 새롭게 배운점이라 생각되고 좋았다.

5. 오늘 실습에서 좋았던 점과 아쉬웠던 점은 무엇인가?

오늘 OT에서 교육 해주셔서 좋았고, 부서를 진료실로 배정받아서 어떤 업무를 하는지 볼수있어서 좋은 경험을 할 수 있어서 좋았다.

아쉬웠던점은 없었다.

1. 오늘 실습경험의 의미 있는 점은 무엇인가?

오늘은 구강보건센터 부서에 들어와서 실습을 하게되었다. 구강보건센터에서 하는일은 노인 의치·보철 사업, 장애인 및 취약계층 무료진료, 학교 구강보건사업, 어린이 충치 예방사업, 불소용액 양치사업, 구강보건 교육, 양치체험 교육 교실, 사회복지시설 구강건강관리사업, 이동식 구강건강관리사업 등 다양한 사업으로 구강보건센터에서 하는일을 알게되어서 의미있었다.

2. 오늘 실습을 하는 동안 나의 생각과 느낌은 어떠했는가?

실습을 하는동안 데스크에 앉아서 불소용액 받으러 오시는 분들에게 명단작성을 하도록 하였고, 구강보건 센터 안은 일반 치과와 같이 깔끔하게 되어 있었고 생각했던 것이랑 달라서 신기했다. 구강보건센터에서 경험할 수 있어서 좋았다.

3. 오늘 실습을 통해 새롭게 알게 된 것은?

보건소에서 구강보건 센터를 들어와본적은 처음인데 다양한 사업을 하고 있다는것을 알게되어서 좋았고 어제와 다른 부서로 경험하게 되니까 어떤일(업무)을 하는지 정확하게 알수 있어서 좋은것 같다. 구강보건 교육실 활용 지역 주민을 대상으로 한 구강건강 교육 홍보도 하며 군산시 보건소에서 운영중이다.

4. 오늘 실습을 통해 새롭게 배운 것들이 향후 간호실무에 어떻게 적용될 수 있겠는가?

구강위생 환경수칙에서 칫솔질 전에 흐르는 물에 비누로 30초 이상 손씻기를 한다. 구강의 위생과 청결을 위해 손을 씻도록한다. 칫솔, 치약, 컵은 개인별로 사용한다. 침으로 발생하는 감염을 예방할수 있도록 다른사람과 함께 사용하지 않는다. 칫솔 간 간격을 두고 보관한다. 여러칫솔을 함께 보관하면 칫솔이 맞닿아 다른 사람의 침이나 혈액이 묻을 수 있으므로 칫솔 간 간격을 두고 가급적 공기가 잘 통하는 곳에 보관하도록 한다. 이런 새롭게 배운것들은 나중에 보건센터에 취업하였을때 익숙하여 일을 수월하게 할 수 있을것 같다.

진로선택.
취업.

5. 오늘 실습에서 좋았던 점과 아쉬웠던 점은 무엇인가?

부서를 다양하게 경험함으로서 배울수 있는것이 많아서 좋은것 같다.

구강보건센터에 대해 알게 되어서 좋았다.

아쉬운점은 없었다.

P9

1. 오늘 실습경험의 의미 있는 점은 무엇인가?

오늘 군산시 치매 안심센터에 와서 무슨 사업운영을 하고 프로그램을 운영하는지 알게되서 좋았고 프로그램에 참여해서 직접 경험을 할수 있어서 의미있었던것 같다. 노인분들이 실종되었을때 지문등록이 되어있으면 더 빨리 찾을수있게 집에복귀하도록 도움을 준다고 하였다.

2. 오늘 실습을 하는 동안 나의 생각과 느낌은 어떠했는가?

실습을 하면서 요즘 고령화 사회인것은 누구나 다 아는 사실이지만 이런 사업과 프로그램이 진행되는것은 대다수가 알지는 못할것 같다는 생각을 했다. 이런 사업도 있구나를 처음 들은것도 있기 때문이다. 따라서 홍보를 더 많이 한다면 노인분들 치매분들이 아니더라도 이런 프로그램을 접하면 좋겠다라는 생각을 했다.

보고도 인식

3. 오늘 실습을 통해 새롭게 알게 된 것은?

치매예방 수칙에는 삼·삼·삼, 3권, 3금, 3행이 있다.

3권 즐길것. 운동 - 일주일에 3번 이상 걷기, 식사 - 생선과 채소를 골고루 챙겨먹기. 독서 - 부지런히 읽고쓰기. 3금 참을것 - 절주 술은 한번에 3잔보다 적게 마시기, 금연 담배는 피우지 않기 뇌손상예방 - 머리를 다치지 않도록 조심하기. 3행 챙길것 - 건강검진 혈압, 혈당, 콜레스테롤 3가지를 정기적으로 체크하기. 소통 - 가족과 친구에게 자주 연락하고 만나기 치매조기 발견 - 매년 치매 안심센터에서 치매 조기검진 받기.

4. 오늘 실습을 통해 새롭게 배운 것들이 향후 간호실무에 어떻게 적용될 수 있겠는가?

오늘 치매 안심센터에서 프로그램이 어떻게 운영되고 센터에서는 무슨일을 하는지 들을수 있었다. 다양한 치매사업과 프로그램이 운영되고 있었다. 노인분들에게 친절히 또박또박 크게 말하며 옆에서 프로그램시 도와주었다. 이런 경험으로 인해서 나중에 보건소 치매쪽으로 가게 된다면 간호업무에 도움이 될것 같다.

5. 오늘 실습에서 좋았던 점과 아쉬웠던 점은 무엇인가?

프로그램을 경험하게 되어서 좋았고, 오늘은 치매안심센터에서 또 날마다 바뀌는 복지로 배우는게 있어서 경험으로 좋았다.

아쉬웠던점은 없었다.

1. 오늘 실습경험의 의미 있는 점은 무엇인가?

오늘은 방문간호 [illegible] 실습을 나왔는데 몇분 어르신들의 집을 방문하여.
혈압이 높은지, 낮은지 확인하고 혈당 유지가 잘되고 있는지 확인하였고.
혈압을 잴수 있도록 해드리면서 어르신들 혈압을 재었다.

2. 오늘 실습을 하는 동안 나의 생각과 느낌은 어떠했는가?

실습하면서 보건소에 수일동안 경험해본 결과 알지 못하는 다양한 사업을 하고 있었고, 어르신들을 위한 사업이 잘 이루어지고 있는것 같아서 좋았다.
몇몇 어르신들의 집을 방문해서 혈압과 혈당체크를 하여 도움을 주고.
낮거나 하면 약조정이 필요하다고 말하면서 병원에 다녀오시라고 한다.
어르신들의 건강을 관리하실을 볼수 있었다.

3. 오늘 실습을 통해 새롭게 알게 된 것은?

AI IOT 기반 어르신 건강관리사업 이란. 어르신의 건강 상태별 필요한 디지털기기를 제공하고 스마트폰 오늘건강 APP을 통해 전문인력이 비대면 방식의 만성질환 관리 및 건강행태 개선을 유도하는 건강관리 사업이다. 사업내용은 사전건강측정 (혈압, 혈당, 신장, 체중 등) 후 개인건강 특성별 디지털 기기 대여. 제공 및 전문인력의 비대면 건강관리 서비스지원. 디지털 기기제공 : 활동량계 (스마트시계) / 대여 : 체중계. 혈압, 혈당 측정기. 대상자별 건강위험요인 고혈압. 당뇨 등에 따라 제공한다.

4. 오늘 실습을 통해 새롭게 배운 것들이 향후 간호실무에 어떻게 적용될 수 있겠는가?

방문건강간호 사업을 본적도 관심을 가져본적도 없었는데 보건소에 와서 경험하게 되서 좋았다. 나중에 보건소에 취업을 하게 되었을때 오늘 보았던 경험을 통해 더 쉽게 다가갈 수 있을것 같다고 생각하였다.
혈압, 혈당을 모바일기기에 연결하여 측정하는것을 보고 신기하였다.

5. 오늘 실습에서 좋았던 점과 아쉬웠던 점은 무엇인가?

오늘 실습은 방문간호였는데 날씨가 하루마다 바뀌어서 새로운 경험을 계속 할수 있어서 좋았다. 점점 최신의료기술이 발달하고 있구나를 느꼈다.
의료기술 발달이 이렇게 되겠구나 하는게 이루어지고 있는 과정을 보니 신기하였다.
아쉬웠던점은 없었다.

P9

보건소 인식

취업 진로 탐색

보건소 인식

1. 오늘 실습경험의 의미 있는 점은 무엇인가?

오늘은 예방접종실에서 경험하게 되었다. B형간염 등 주사를 접종하려고 오시는 분들이 오시면 선생님들께서 진료실로 안내하여 의사선생님을 만나고 다시 접종실에 오면 주의사항 및 부작용에 대해 설명해주시고 언제 항체검사 해보시라는 자세한 사항도 안내해 주셔서 배운점이 있어서 의미있었다.

2. 오늘 실습을 하는 동안 나의 생각과 느낌은 어떠했는가?

실습하는동안 접종실에서 무슨 업무를 하고 접종 받으시는 분들 안내와 선생님이 접종하는것을 볼수 있어서 좋았고. 어떻게 일을 하는지 관찰할수 있어서 경험있는 하루였다. 다양한 보건사업이 있어서 많은것을 볼 수 있었다.

3. 오늘 실습을 통해 새롭게 알게 된 것은?

필수예방접종비용은 국가부담사업이고 사업에 참여하는 가까운 의료기관에서 국가필수 예방접종시 접종 비용을 지원해준다. 결핵, B형간염, DTap (디프테리아 백일해, 파상풍). 등 여러 접종이 있다.

보건소에서 접종이 몇개 없고 한정적인줄 알았는데 여러개의 접종을 지원하고 있어서 새롭게 알게되었다.

4. 오늘 실습을 통해 새롭게 배운 것들이 향후 간호실무에 어떻게 적용될 수 있겠는가?

보건소에서 부서마다 경험을 해보아서 새롭게 배운것도 많았고, 여러 프로그램이 있다는것을 알게되고 직접 참여해본것도 많았는데 나중에 보건소에 취업을 하였을때 이번에 지역실습을 통해 배운것이 도움이 된것이라고 생각했다

5. 오늘 실습에서 좋았던 점과 아쉬웠던 점은 무엇인가?

마지막으로 예방접종실에서 실습을 하였는데 보건소에서 여러부서를 다니면서 경험할 수 있어서 더 좋은 경험인것 같다. 한정적으로 경험하지 않아서 좋았다 아쉬운점은 없었다.

[illegible]

결핵관리실에서 선생님들께서 고객분들을 응대해주시는 모습을 볼 수 있었던 점. 군산시 보건소의 특징을 살펴보고, 이를 들으며 보건소의 업무를 들을 수 있었던 점. 결핵관리실에서 ~~[illegible]~~ 결핵에 대해 설명을 들을 수 있었던 점이 의미 있었다.

2. 오늘 실습을 하는 동안 나의 생각과 느낌은 어떠했는가?

선생님께서 결핵관련해서 설명을 해주셨는데 결핵치료가 약물로 생각보다 많은 약물이 사용되고 있는 것을 알게되었다. 각각 다른 약물에 부작용도 다르고, 약물의 투여방법에 대해 잘 알고 간호수행을 해야겠다는 생각을 하게 되었다

투약 간호

3. 오늘 실습을 통해 새롭게 알게 된 것은?

선생님께서 잠복결핵감염에 대해 설명해주시며, 다시 학습하게 되었다. 잠복결핵감염 이란? 몸속에 들어온 결핵균이 인체 내의 방어면역반응에 의해 증식하지 않고, 결핵으로 진행하지 않는 경우가 있는데 이를 잠복결핵감염이라고 한다는 것을 다시 알게되었다. 잠복결핵감염은 몸 안에 결핵균이 존재하지만 활동 및 증식하지 않아 결핵이 발병하지 않은 상태를 말한다고 한다라는 것을 다시 인지할 수 있었다.

4. 오늘 실습을 통해 새롭게 배운 것들이 향후 간호실무에 어떻게 적용될 수 있겠는가?

향후 간호실무에서 결핵이 어떤 것인지 인지하고, 어떤 간호가 필요한지, 어떤 약물을 써야하는지 잘 인지하고 있어 대상자에게 올바른 간호와 올바른 교육을 제공할 수 있을 것 같다. 대상자가 어떤 증상을 보이고, 어떤 부작용이 있는지 잘 파악해 올바른 간호를 제공할 것이다.

간호사 역할 교육자

5. 오늘 실습에서 좋았던 점과 아쉬웠던 점은 무엇인가?

좋았던점 - 결핵에 대해 어떤 약물이나 어떤 부작용이 있는지 인지할 수 있던점. 보건소의 업무에 대해 선생님들께 들어볼 수 있었던점이 좋았다.

아쉬운점 X.

CamScanner로 스캔하기

대상자분들이 계신 경로당을 방문하여 혈압과 혈당을 측정해 드린점, 대상자분들의 가정집을 방문하여 비타민을 나눠주며 인사드릴수 있던점, 어르신들과 계란 먹으면서 대화할수 있던 점이 의미있었다.

2. 오늘 실습을 하는 동안 나의 생각과 느낌은 어떠했는가?

실습 중 경로당이나 가정집을 방문하며, 방문관리팀에서는 어떤 일을 하는지, 방문하여 어떤 것을 교육하는지 옆에서 관찰하여. 보건소의 업무가 생각보다 광범위 하다는 것을 느끼게 되었다. 굉장히 매력있는 직업이라고 생각했다. 1차간호 인식

3. 오늘 실습을 통해 새롭게 알게 된 것은?

보건소 방문관리팀에서 가정이나 경로당을 방문하여 대화뿐아니라 BP나, 혈당등을 측정하여 대상자들의 기본적인 검진도 시행한다는 것을 알수 있었고, 추가로 결핵, 고지혈증이나, 고혈압등 어르신들이 많이 앓고 계신 질병에 대해 교육한다는 것을 알수 있었다.

4. 오늘 실습을 통해 새롭게 배운 것들이 향후 간호실무에 어떻게 적용될 수 있겠는가?

향후 간호실무에서 임상에서 나와 공무원을 통해 보건소에서 일하게 된다면 방문관리팀에서 업무를 맡아보고 싶은 생각이 들었고, 어르신들이 눈높이에 맞춰 이해하기 쉽게 교육을 잘 진행할 수 있을 것 같다. 의사소통

5. 오늘 실습에서 좋았던 점과 아쉬웠던 점은 무엇인가?

좋은점 - 경로당 방문을 통해 어르신의 BST, BP를 측정하고 소통할수 있던점
-방문관리 팀에서 무슨 일을 하는지 관찰해 볼수 있던점이 좋았다.

아쉬운점 - 가정방문때 대상자가 유방암 환자라 모른채 갔던 것이 아쉽다

1. 오늘 실습경험의 의미 있는 점은 무엇인가?

건강보험공단 군산지사에서 대상자들에게 암검진 유선독려를 해볼 수 있던 점, 암에 대해 깊게 교육받을 수 있던 점, 대상자들에게 검진 안내에 대해 설명하며 소통할 수 있던 점, 6대 암검진에 대해 알아볼 수 있던 점에 의미가 있었다.

2. 오늘 실습을 하는 동안 나의 생각과 느낌은 어떠했는가?

실습을 통해 대상자들에게 암검진에 대해 안내해드리며 소통하는 기회를 가지게 되었는데, 자궁경부암의 경우 임신중이나 성경험이 없을 경우, 자궁적출술을 받거나 치료중인 경우에는 자궁경부암 검진을 받지 않아도 된다는 것을 교육하며, 대상자에게 올바르게 교육하는 것도 중요한 과정중 하나라고 느끼게 되었다.

3. 오늘 실습을 통해 새롭게 알게 된 것은?

6대 암검진에 대해 새롭게 알게되었다. 먼저 위암은 2년주기로 검사하며 위내시경검사, 조직검사를 시행한다고 한다. 대장암은 1년주기로 분변잠혈검사, 대장내시경검사, 조직검사를 시행한다고 한다. 간암은 6개월주기로 간초음파검사, 혈청알파태아단백검사를 하며, 폐암은 2년주기로 저선량흉부CT, 상담등을 시행한다고 한다. 유방암은 2년주기로 유방촬영검사를 시행하며, 자궁경부암은 2년주기로 자궁경부세포검사를 한다고 한다.

4. 오늘 실습을 통해 새롭게 배운 것들이 향후 간호실무에 어떻게 적용될 수 있겠는가?

향후 간호실무에서 대상자에게 암에 대한 올바른 교육을 시행할 수 있으며 어떤 암에는 무슨 검사가 필요한지, 무슨 치료가 필요한지 등 많이 공부하여 대상자가 올바른 회복을 할 수 있도록 할 것 같다.

5. 오늘 실습에서 좋았던 점과 아쉬웠던 점은 무엇인가?

좋았던 점 - 대상자들에게 암검진 유선독려를 해봤던 점, 대상자들에게 암검진에 대해 설명하며 소통할 수 있던 점, 6대 암검진에 대해 알아볼 수 있던 점이 좋았다.

아쉬운 점 - 대상자들에게 전화했을 때 받지 않는 분이나 화난 [illegible] 분께 제대로 알려드리지 못해 아쉽다.

CamScanner로 스캔하기

1. 오늘 실습경험의 의미 있는 점은 무엇인가?

영양플러스 사업이 어떤 사업인지 알 수 있던 점, 영양플러스 사업의 대상자가 어떤 대상자인지 알 수 있던 점. 어떤 방식으로 사업이 진행되는지, 보충식품종류에는 무엇이 있는지 팜플렛을 읽어 볼 수 있던 점이 의미있었다.

2. 오늘 실습을 하는 동안 나의 생각과 느낌은 어떠했는가?

보건소 영양플러스 팀에서 제공하는 서비스에는 1. 영양교육 및 상담서비스 2. 정기적 영양평가 3. 보충식품지원 이 3가지로 구체적으로는 올바른 영양 및 식생활 관리, 모유수유/이유식 방법, 가정방문 교육등으로 영양위험요인이 큰 임산부와 영유아에게 디테일한 사업을 진행한다는 것을 느끼게 되었고, 사업체계가 잘되어 있다는 것을 느끼게 되었다.

3. 오늘 실습을 통해 새롭게 알게 된 것은?

영양플러스 사업중 보충식품 지원 사업이 있는데 보충식품들에는 어떤 종류의 식품들이 있는지 알게되었다. 임산부와 영유아를 대상으로 한 식품이라 그들에게 어떤 음식이 좋은지 알게되었다.
◎ 지원하는 보충식품의 종류로는 분유와 쌀, 우유, 달걀, 검정콩, 당근, 감자, 김, 미역, 참치통조림, 오렌지주스 1.5L 가 보건소 영양플러스 사업에서 지원하는 보충식품 이라고 한다.

4. 오늘 실습을 통해 새롭게 배운 것들이 향후 간호실무에 어떻게 적용될 수 있겠는가?

진로선택?
△

향후 간호실무에서 공무원이 되어 영양사업을 맡는다면 영양위험요인이 큰 임산부와 영유아에게 보충식품 제공 및 체계적인 영양교육 및 상담을 통해 각 임산부마다 개개인의 특성이 있으므로 개인에 맞는 맞춤형 지원을 올바르게 제공할 수 있을 것 같다.

5. 오늘 실습에서 좋았던 점과 아쉬웠던 점은 무엇인가?

좋았던 점 - 영양플러스 사업에 대해 알 수 있었고, 사업의 종류에는 어떤 것이 있는지 구체적으로 살펴볼 수 있었던 점이 좋았다.

아쉬운점 - 오늘은 예정된 프로그램이 없어 직접 사업진행내용을 관찰하지 못한점이 아쉽다.

CamScanner로 스캔하기

1. 오늘 실습경험의 의미 있는 점은 무엇인가?

치매분소에서 치매예방수칙 333에 대해 읽어볼 수 있던점. 치매분소의 업무 리플렛을 읽어 볼 수 있던점, 치매예방을 위한 체조가 어떤 것들이 있는지 확인해볼 수 있던 점이 의미있었다.

2. 오늘 실습을 하는 동안 나의 생각과 느낌은 어떠했는가?

실습을 통해 치매분소에서 어떤 일을 하는지 보았을 때 기본적인 상담이나 인지 선별검사, 정밀검사등과 같이 세밀한 체계가 이루어져 있다는 것을 느끼게 되었고, 생각했던 것보다 많은 프로그램이 진행되고 있다는 사실을 느끼게 되었다.

3. 오늘 실습을 통해 새롭게 알게 된 것은?

치매예방 프로그램에는 치매환자 쉼터나 치매예방교실 정도로만 알고 있었는데 추가로 인지강화교실, 치매가족 지원 교실등도 운영하고 있다는 것을 새롭게 알게되었다. 보건소인식

인지강화교실은 인지저하, 경도인지 장애로 진단을 받은 어르신들을 위한 인지 향상 교육을 말하며, 치매가족 지원 교실은 치매환자를 돌보는 가족을 대상으로 교육 및 스트레스 해소 프로그램이라고 한다.

4. 오늘 실습을 통해 새롭게 배운 것들이 향후 간호실무에 어떻게 적용될 수 있겠는가?

향후 간호실무에서 치매 진단을 받은 대상자가 있으면 올바른 간호를 제공하며, 대상자 뿐만 아니라 가족 간호까지도 고려할 수 있는 간호사가 될 것이다.
또한 '치매'라는 특징에 맞추어 대상자들이 이해하고, 따라하기 쉬운 좋은 프로그램을 운영할 수 있을 것 같다.

5. 오늘 실습에서 좋았던 점과 아쉬웠던 점은 무엇인가?

좋았던 점 - 치매분소에서 무슨 일을 하는지, 어떤 체계로 이루어져 있는지 파악해볼 수 있었던 점
- 사무실에 있는 팜플렛을 통해 치매에 대한 많은 정보를 접할수 있던 것이 좋았다.

아쉬운점 - 없다♡

CamScanner로 스캔하기

1. 오늘 실습경험의 의미 있는 점은 무엇인가?

결핵관리실에서 선생님들께서 고객분들을 응대해주시는 모습을 볼수 있었던 점. 군산시 보건소의 특징을 살펴보고, 얘기 들으며 보건소의 업무를 들을수 있었던 점. 결핵관리실에서 결핵에 대해 설명을 들을 수 있었던 점이 의미 있었다.

2. 오늘 실습을 하는 동안 나의 생각과 느낌은 어떠했는가?

선생님께서 결핵관련해서 설명을 해주셨는데 결핵치료가 약물로 생각보다 많은 약물이 사용되고 있는 것을 알게되었다. 각각 다른 약물이 부작용도 다르고, 약물의 투여방법에 대해 잘 알고 간호수행을 해야겠다는 생각을 하게 되었다.

투약 간호

3. 오늘 실습을 통해 새롭게 알게 된 것은?

선생님께서 잠복결핵감염에 대해 설명해주시며, 다시 학습하게 되었다. 잠복결핵감염 이란? 몸속에 들어온 결핵균이 인체 내의 방어면역반응에 의해 증식하지 않고, 결핵으로 진행하지 않는 경우가 있는데 이를 잠복결핵감염이라고 한다는 것을 다시 알게 되었다. 잠복결핵감염은 몸 안에 결핵균이 존재하지만 활동 및 증식하지 않아 결핵이 발병하지 않은 상태를 말한다고 한다라는 것을 다시 인지할 수 있었다.

4. 오늘 실습을 통해 새롭게 배운 것들이 향후 간호실무에 어떻게 적용될 수 있겠는가?

향후 간호실무에서 결핵이 어떤 것인지 인지하고, 어떤 간호가 필요한지, 어떤 약물을 써야하는지 잘 인지하고 있어 대상자에게 올바른 간호와 올바른 교육을 제공할 수 있을 것 같다. 대상자가 어떤 증상을 보이고, 어떤 부작용이 있는지 잘 파악해 올바른 간호를 제공할 것이다.

간호사 역할 교육자

5. 오늘 실습에서 좋았던 점과 아쉬웠던 점은 무엇인가?

좋았던점 - 결핵에 대해 어떤 약물이나 어떤 부작용이 있는지 인지할 수 있던점. 보건소의 업무에 대해 선생님들께 들어볼수 있던점이 좋았다.

아쉬운점 X.

1. 오늘 실습경험의 의미 있는 점은 무엇인가?

보건소에서 해보는 첫 실습이고 지역간호학 실습 OT가 꼼꼼하게 이루어졌다. 치매바로 알기라고 치매에 대해서 설명해 주시고 국민건강 보험공단에서 국민 건강보험 OT. 진드기 매개 감염병에 대한 OT를 해주셔서 많은 걸 배웠다.

2. 오늘 실습을 하는 동안 나의 생각과 느낌은 어떠했는가?

☆ 보건소 업무

보건소는 코로나 이전에는 아이들 예방접종과 독감 예방접종을 위해 이용해서 별게 없다고 생각이 들었는데 지역간호학 이론에서 배운 것과 같이 치매부터 건강증진까지 참으로 다양한 일을 하고 계신다는 생각이 들었다.
시민들이 다 알지 못해서 이용 못하는 것이 많은 것 같다.

3. 오늘 실습을 통해 새롭게 알게 된 것은?

진드기가 무섭다고는 생각했지만 OT를 통해서 종류와 증상에 대해서 알게 되었다.
참진드기는 SFTS (중증 열성 혈소판 감소 증후군) : 라임병. 진드기 매개 뇌염을 일으키고. 털진드기는 쯔쯔가무시증을 일으킨다.
특히 SFTS는 잠복기는 5일~14일. 환자발생시기는 4월-11월 사이 고열. 오심 구토. 설사: 식욕부진 등의 소화기 증상을 시작으로 의식저하와 혈소판 감소 등을 보인다.

4. 오늘 실습을 통해 새롭게 배운 것들이 향후 간호실무에 어떻게 적용될 수 있겠는가?

SFTS는 뉴스에서도 봤었고 치명률이 높아 생명을 잃기도 한다 진드기에 물렸다면 모르는 경우도 많으나 손으로 터트리거나 잡지말고 보건소 또는 의료기관에 제거를 권하고 산이나 밭에서 일할 때는 작업복을 갖춰 입고 진드기 기피제를 사용할 것을 알려야 한다. 작업복 역시 즉시 세탁해야 하는 것도 잊지 말아야한다

5. 오늘 실습에서 좋았던 점과 아쉬웠던 점은 무엇인가?

건강증진실 배치를 받고 여기저기 둘러보고 싶었는데 담당선생님이 안계셔서 오전 내내 앉아만 있었다.
쉬는 것이 좋기도 하지만 설명이 전혀 없어서 아쉬웠다.
마지막 실습인 만큼 잘 마무리 해야겠다.

CamScanner로 스캔하기

1. 오늘 실습경험의 의미 있는 점은 무엇인가?

오늘은 보건소가 아닌 나운동에 치매안심센터에서 실습을 하였다. 어제도 보건소에서 치매에 대해 설명해 주었는데 오늘은 치매안심센터에서 하고 있는 일을 여러가지로 구분하여 더 자세하게 알 수 있었다.

2. 오늘 실습을 하는 동안 나의 생각과 느낌은 어떠했는가?

부모님이 치매를 걱정하는 나이가 되다보니 치매안심센터에 가보라고만 했지 어떠한 프로그램이 있는지 몰랐다.

선별검사부터 정말 많은 프로그램이 있다는 걸 알고 놀랐다. 걸리면 안되는 무서운 병이지만 치매안심센터를 통해 자신의 경도인지 장애등을 먼저 알고 대처해야겠다는 생각이 들었다.

보건소인식

3. 오늘 실습을 통해 새롭게 알게 된 것은?

치매유병자를 위한 프로그램과 지원에 관한 다양한 OT를 들으면서 치매안심마을이 있다는 것과 치매 예방 수칙 3.3.3이 있다는 걸 알았다.

3권 (즐길것) - 운동. 식사. 독서. 3금 (참을것) : 절주. 금연. 뇌손상예방
3행 (챙길것) : 건강검진. 소통. 치매조기발견

치매안심 마을은 지역사회 구성원들이 치매에 대해 올바르게 이해하고 치매 환자와 가족의 고립을 방지하기 위해 지역특성에 따라 조성한 마을이다.

4. 오늘 실습을 통해 새롭게 배운 것들이 향후 간호실무에 어떻게 적용될 수 있겠는가?

치매 친화적 지역사회 환경이 조성되면 치매예방정보들을 자연스럽게 접할 수 있게 된다.

노화로 인해 어쩔 수 없다고 방치하지 말고 세대별로 젊어서부터 노년기까지 할수 있는 것들을 찾아보고 운동은 평생할 수 있는 것으로, 우울증 꼭 치료하기. 노년기에는 치매 예방체조를 하고 여러 사람들과 자주 어울리고. 치매안심센터에서 치매조기검진을 받아야 한다.

5. 오늘 실습에서 좋았던 점과 아쉬웠던 점은 무엇인가?

월.수에만 프로그램이 있어서 우리는 프로그램에 참여하지는 못했다. 오후에 1층 접수실에서 민원인들이 다양한 물품을 타가는 것을 볼수 있었고. 2층에 어르신들이 직접 만드신 다양한 물품이 전시되어 있어서 구경해 볼수 있어서 좋았다.

부모님께도 서둘러 치매조기검진을 권해 볼 수 있는 계기가 되었다.

보건소 인식

CamScanner로 스캔하기

1. 오늘 실습경험의 의미 있는 점은 무엇인가?

오늘은 방문건강관리실에서 실습을 했다. 오전에 암환자 분들에게 영양제를 나누어주고 오후에는 경로당에 방문해서 혈당과 혈압을 재어드리고 신규 어르신들의 문진과정을 지켜보았다. 시범사업중인 AI 사업으로 어르신 건강관리 사업관리도 관찰할 수 있었다.

2. 오늘 실습을 하는 동안 나의 생각과 느낌은 어떠했는가?

대상자분들께 컨디션과 요즘 근황을 꼼꼼하게 문진하시고 AI로 관리하시는 분들의 문제점과 잘하고 계신 부분은 칭찬하시고 격려하시는 선생님을 보면서 즐겁게 일하고 계신 듯 하였다. 어르신들이 외로우신지 계속 고마워하시고 대화를 하는 것으로도 좋아하셨다. 귀찮기도 할텐데 즐기시는 모습을 보면서 배워야겠다는 생각을 했다.

3. 오늘 실습을 통해 새롭게 알게 된 것은?

방문건강 관리사업은 지역주민의 건강인식 제고, 자가 건강관리 능력향상 건강상태 유지 및 개선이 목적이고 지역보건법을 법적 근거로 하고 있다. 관할보건소에 대상자 등록 후 관리한다.
방문간호는 일상생활을 혼자 수행하기 어려운 노인에게 간호서비스 제공을 통한 부담을 경감시켜주고 노인장기요양 보험법에 의해서 노인 요양 보호 등급판정자가 등급 판정후 요양기관과 서비스를 계약한다.

4. 오늘 실습을 통해 새롭게 배운 것들이 향후 간호실무에 어떻게 적용될 수 있겠는가?

가정간호는 입원환자의 입원 기간 단축, 국민의료비를 절감하고 환자와 가족의 편의를 제공한다. 의료법에 의해 민간 및 국공립 의료기관에 의해 운영된다. 조기 퇴원환자, 외래환자를 대상으로 진료담당의사가 환자와 협의 후 가정간호를 의뢰한다. 세가지가 헷갈리는 경우가 많았는데 방문건강관리팀에서 실습하면서 제대로 비교하고 알 수 있었다.

5. 오늘 실습에서 좋았던 점과 아쉬웠던 점은 무엇인가?

좋은 선생님과 함께 방문건강관리를 할 수 있어서 좋았는데 맛있는 커피 사주신다고 멀리까지 가셔서 커피를 사주셔서 더 좋았다.
시간이 조금더 있다면 여러 케이스를 관찰했다면 하는 아쉬움이 남는다.

CamScanner로 스캔하기

1. 오늘 실습경험의 의미 있는 점은 무엇인가?

오늘의 실습지는 국민건강보험공단였다. 선생님께 건강검진을 위한 안내대본을 받고 자궁경부암을 검진 받지 않으신 분들에게 전화를 했다. 지역사회간호학 실습에서는 병원과 다른 여러가지 경험을 하는 것 같다..

2. 오늘 실습을 하는 동안 나의 생각과 느낌은 어떠했는가?

컨퍼런스를 할 때 친구들의 케이스 발표를 보고 들으면서 같은 걸 보고도 이렇게 다를 수 있구나 하고 많은 걸 배우는 것 같다. 또한 같은 걸 경험해도 부정적인 시선으로 보고 느끼는 사람도 있고 즐거워하는 친구도 있는 걸보면 사람의 생각은 가지가지 인 듯 하다. 항상 긍정적이고 좋을 수는 없겠지만 기쁜 마음으로 모든 일에 임하려고 노력해야 겠다.

3. 오늘 실습을 통해 새롭게 알게 된 것은?

국가건강검진을 해당년도에 매번 검진은 받았는데 나에게 해당되는 정도만 알았는데 위암. 대장암. 자궁경부암 외에도 간암과 폐암검진도 시행되고 있었다.

간암은 : 간경변, C형간염 바이러스 항체 양성, B형간염 바이러스항원 바이러스, B형, C형 간염 바이러스에 의한 만성 간질환을 가진 고위험군 환자를 대상으로 이루어진다.

4. 오늘 실습을 통해 새롭게 배운 것들이 향후 간호실무에 어떻게 적용될 수 있겠는가?

폐암은 문진시 30갑년이상 흡연, 30갑년 이상으로 국가건강검진을 받았던 자로 검진후 금연 15년 이내. 74세 까지 인 자로 저선량 흉부 CT검사 → 검진결과 사후상담 및 금연 상담이 이루어 진다.

몰라서도 못 받는 혜택은 어쩔 수 없지만 홍보도 많이하고 건강을 지키는 국가건강검진은 꼭 혜택을 누리도록 주위분들이라도 알려야겠다.

5. 오늘 실습에서 좋았던 점과 아쉬웠던 점은 무엇인가?

국민건강보험공단에서 왔다갔다 하는 건 번거로웠지만 안내 전화도 해보고 국민 건강보험공단 사무실에서 어떤 일을 하는지 볼수 있어서 좋았다. 마지막 실습의 마지막 컨퍼런스를 하고 내일만 실습하면 끝난다고 생각하니 정말 정말 기쁘다.

CamScanner로 스캔하기

1. 오늘 실습경험의 의미 있는 점은 무엇인가?

오늘은 금연클리닉에서 실습을 했다. 생각보다 금연을 원하는 방문자들이 많았다.
상담을 하면서 문진을 하고 대상자에게 맞는 패치와 여러가지 선물을 주고 있었고 금연유지프로그램까지 10회 상담이 이루어진다는 것이 놀라웠다.

2. 오늘 실습을 하는 동안 나의 생각과 느낌은 어떠했는가?

중독이라는 것이 얼마나 무서운 것인지 다시 찾아오는 분들도 많이 계셨다. 술자리가 있어서, 습관적으로 피우는 담배들이였다.
혼자의 의지로 금연이 어렵다면 금연클리닉의 도움을 받아서 금연에 도전해 보는 것이 좋겠다는 생각을 했다.
우리 가족들이 담배를 피우지 않는 것이 당연하다고 생각했는데 오늘은 감사하다는 생각이 들었다.

3. 오늘 실습을 통해 새롭게 알게 된 것은?

담배냄새가 싫고 간접흡연이 안좋다고 만 생각했는데 간접흡연에 대해서 제대로 알 수 있었다.
담배연기는 흡연자가 흡입한 뒤 내뿜는 주류연(주변공기의 20%)과 담배 끝에서 피어나는 연기인 부류연(주변공기의 80%)로 구성된다.
부류연은 주류연에 비해 암모니아, 타르, 벤젠 등의 독성화학물질의 농도가 높고 담배연기 입자가 더 작아 폐의 깊은 곳까지 영향을 준다.

예방
활동.

4. 오늘 실습을 통해 새롭게 배운 것들이 향후 간호실무에 어떻게 적용될 수 있겠는가?

아이들이 담배연기로 간접흡연에 노출되면, 성인보다 피해의 정도가 크다. 감기나, 천식, 중이염 등에 걸릴 확률도 높아지고 폐기능의 발달도 전반적으로 저하된다. 뇌종양, 림프종, 백혈병, ADHD, 뇌졸중의 위험도 높아진다는 연구가 있다. 흡연자들에게 자신의 건강과 예방을 위해서 금연클리닉의 이용을 적극 홍보해야 할것이다.

5. 오늘 실습에서 좋았던 점과 아쉬웠던 점은 무엇인가?

단 1주일 이지만 보건소에서 실습을 하면서 여러 부서들을 경험해 볼수 있어서 좋았다.
드뎌 교외실습이 끝이나서 다 해낸것 같은 기분이 들어서 행복하다.

1. 오늘 실습경험의 의미 있는 점은 무엇인가?

<이동진료팀>
오전에, 원광대 교수님이 오셔서 '만성질환의 이해" 교육 하시는 것을 수강하였다. 당뇨병, 고혈압, 생활습관에 대해 설명하셨는데 평소에 알던 것에 심화내용까지 있어서 유익한 시간이 된 것 같았다. 그리고 오후에, 이동진료팀 활동으로 경로당에 가서 혈압/혈당 체크를 하는 좋은 경험이 된 것 같았다.

2. 오늘 실습을 하는 동안 나의 생각과 느낌은 어떠했는가?

하루 하루 교육을 통해서 잊었던 혹은 알지 못했던 지식을 알아가고 '이런 것도 있구나' 하며 깨닫는 좋은 경험이 된 것 같다. #실습
오후에는 이동진료팀 활동으로 개정면의 '정수경로당'에 가서 경로당에 계신 어르신분들의 혈압/혈당을 체크하여 건강상태를 확인하였고 한의사분도 가서 어르신께 아픈 부위에 침 놔드리고 하셨다. 보건소에서는 이렇게 방문간호와 같은 대상자분들을 직접 찾아가는 서비스도 한다는 것을 알게되었다.

3. 오늘 실습을 통해 새롭게 알게 된 것은?

평소에 당뇨병, 고혈압에 대해서 알았던 지식과 성인간호학 때도 배웠었지만, 당뇨병은 공복혈당 126mg/dL 이상, 식후 혈당은 200 mg/dL은 항상 기억하였지만 "당화혈색소"가 중요하다는 것을 잊고 있었다. / 당화혈색소 6.5 이상이면 당뇨병이라는 사실을 잊지 말아야겠다. 오후에, 이동진료팀활동으로 경로당을 가서 건강상태를 확인하였는데 대부분이 혈압이 있으시고 혈당도 어르신들의 있으신분이 꽤 있어서 관리가 중요하다는 것을 깨달았다.

4. 오늘 실습을 통해 새롭게 배운 것들이 향후 간호실무에 어떻게 적용될 수 있겠는가?

당뇨병과 고혈압은 완치의 개념이 없기 때문에 평소 식이습관이나 생활습관들이 매우 중요하다는 것을 느꼈다. / 유산소 운동(걷기)와 나트륨이 적은 식이 및 생활요법을 통해 관리하게 되는데 나도 당뇨병과 고혈압 환자에게 관리의 중요성에 대해 설명할 수 있도록 노력해야겠다. 이동진료를 통해 혈압과 당뇨 관리에 대해 어르신께 이해가 쉽도록 설명해드릴 수 있도록 전문 지식을 향상시켜야겠다고 생각했다.

5. 오늘 실습에서 좋았던 점과 아쉬웠던 점은 무엇인가?

어제부터 느끼는 건데, 보건소는 OT와 보건교육들이 체계적으로 진행되는 것 같았다. OT와 교육하시는 분들 다들 설명을 잘 하시고 이해가 쉽게 설명해주셔서 유익한 시간이 되었다.
어르신들을 직접 찾아뵙고 건강상태를 확인하였는데, 병원과는 다른 느낌이 들었다. 이렇게 방문사업도 중요하다는 것을 깨달았고 알아가는 계기가 된 것 같다.

CamScanner로 스캔하기

P12

1. 오늘 실습경험의 의미 있는 점은 무엇인가?

<방문건강관리실>

방문건강관리실 활동으로 외부로, 취약계층(노인, 장애인 등) 대상으로 가정 방문간호사님과 가정방문간호를 수행했다. 방문간호를 나가면 어떤 활동을 하고 어떻게 사정하는지 등을 알게되어서 뜻깊은 경험이 된 것 같다.

2. 오늘 실습을 하는 동안 나의 생각과 느낌은 어떠했는가?

처음으로 가정방문간호 활동을 하였는데, 대상자의 가정(집)까지 가는 데 시간은 좀 걸렸지만 대상자분들은 취약계층인 노인과 장애인분들이었는데 다들 친절하시고 반갑게 맞이해주셨다. 병원에서는 한꺼번에 많은 환자를 돌보지만, 방문간호는 대상자분 한분 한분 찾아뵈어야 하니 발로 뛰어다니는 느낌?이 나고 약간 힘들 것 같다는 생각이 들었다.

전문직

2-2

3. 오늘 실습을 통해 새롭게 알게 된 것은?

'방문건강관리사업'이란, 보건소에 소속된 방문간호사가 각 가정을 방문하여 가족과 건강문제를 가진 구성원을 발견하여 질병예방 및 관리, 건강증진을 위하여 건강서비스를 제공하는 것으로, 방문간호 대상은 기초수급자, 독거노인, 차상위계층, 장애인, 독거노인, 타기관에서 의뢰한 건강문제가 있는 대상자, 재가암 환자관리, 재가장애인 집단서비스 -경로당이 있다. 방문건강관리의 목표는 크게 두가지로, 건강행태개선과 건강문제관리를 목표로 두고있다.

건강상태인식, 건강생활실천유도 등. ↳ 건강문제 정기적 스크리닝, 증상 조절, 치료 순응 향상

2-2

4. 오늘 실습을 통해 새롭게 배운 것들이 향후 간호실무에 어떻게 적용될 수 있겠는가?

병원에서 혼자 간호술기들을 잘 할 수 있도록 경력을 쌓아서 방문간호사로 V/S, BST 뿐만 아니라 다른 간호술기도 적용할 수 있도록 해야겠다는 생각이 들었다. 그리고 대상자와 1:1 대화를 해야되기 때문에 친절하고 상냥한 말투와 치료적 의사소통을 통해 대상자의 상태를 사정할 수 있도록 치료적의사소통을 좀 더 배워야겠다고 생각했다.

진로선택

5. 오늘 실습에서 좋았던 점과 아쉬웠던 점은 무엇인가?

방문간호사가 하는 일은 무엇일까? 궁금했는데 이렇게 직접 실습을 통해 방문간호사가 하는 일을 알게되어 좋았다. 방문간호사가 되기위해 운전과 간호술기들을 다 잘 수 있어야 한다는 점, 대상자의 상태와 반응을 알아보기 위해 최근 근황과 말로 사정한다는 점 등 아직 내가 부족한 것이 많다는 걸 느꼈고 노력해야겠다고 생각했다.

2-2

#진로선택

대면비대면 점검

CamScanner로 스캔하기

P 12

1. 오늘 실습경험의 의미 있는 점은 무엇인가?

〈방문건강관리실〉

오전에 민원인(?) 보건소를 이용하는 분들에게 보낼 우편물 포장하는 작업들을 하고 오후에는 경로당에 가서 어르신들의 V/S과 BST를 측정하여 건강상태를 확인하고 프로그램이 잘 진행 되는지 확인하는 활동을 하였다.

#실습

2. 오늘 실습을 하는 동안 나의 생각과 느낌은 어떠했는가?

보건소는 행정과 여러부서가 있기 때문에 각 각의 맡은 일이 다른데, 확실히 병원보다는 서류작업들이 많아보였다. 오전에 보건소를 이용하시는 분들께 담당 복지사의 명함이 들어 있는 안내문을 넣고 주소가 맞는지 이중확인하고 우편물을 포장하는 작업들을 하였는데 실습중에 서류작업을 하니 약간 색다르게 보였다.

2-1

3. 오늘 실습을 통해 새롭게 알게 된 것은?

어제 방문보건활동을 하면서도 느낀 것이지만, 임대주택에 사시는 취약계층(노인, 장애인 등)분들은 임대주택이 국가의것이기 때문에 이사·이동하라는 공지가 뜨면 이사를 하시는 것 같았다. 어제 어르신도 그 말씀을 하셨고 오늘 서류작업을 하면서 주소가 바뀐 분들도 여럿 있어서 선생님께 주소 확인 후, 작업을 하였다.

2-2 방문 간호

4. 오늘 실습을 통해 새롭게 배운 것들이 향후 간호실무에 어떻게 적용될 수 있겠는가?

간호중재를 하던, 서류작업을 하던, 이중 확인은 꼭 해야겠다. 중요하다고 생각이 되었다. 앞으로도 재차 확인하는 습관을 들여야겠다고 생각했고 오후에, 경로당을 가서 어르신들의 V/S과 BST를 측정하여 건강상태를 확인하고 보건소에서 파견(?)하는 프로그램이 잘 진행되는지, 방문간호사님이 확인한다고 하셔서 방문간호사가 이 업무도 하는구나. 라고 생각했다.

2-1

[illegible]

#보건소

5. 오늘 실습에서 좋았던 점과 아쉬웠던 점은 무엇인가?

오늘은 오전부터 비가와서 약간 마음이 울적했던 것 같은데 그래도 오후에 경로당에 가서 활동을 하니 그나마 좀 나아진 것 같았다. 방문간호사가 하는 일이 V/S, BST, 건강상태 뿐만 아니라 다른 활동들도 있다는 점을 알게되어 새로웠다.

CamScanner로 스캔하기

P12

1. 오늘 실습경험의 의미 있는 점은 무엇인가?

<건강증진실>

집담회를 통해서 지역사회간호PBL을 어떤 식으로 하는지, 가이드라인을 알게 된 것 같았다. 그리고 오후에는 건강증진실에서 실습을 하게 되었는데 모바일 헬스케어 사업이 어떻게 진행되는지 알게 되었으며, 신청인들이 오셔서 어떻게 처음에 시작되는지부터 차근차근 절차에 대해 알게되었다.

2. 오늘 실습을 하는 동안 나의 생각과 느낌은 어떠했는가?

지역사회PBL은 지역사회1 때 조별과제로 하였었는데, 그때도 자료조사 및 간호과정 내리는데 어려웠는데 이번에도 역시 혼자 자료조사하며 간호사정하는 데 어려움을 겪었다. 통일되지 않은 자료들과 추가할 점을 잘 보완해야겠다고 생각했다. 오후에, 건강증진실에서 실습하였는데 혈액검사시 3~5cc씩 채혈하는 것이 아닌 BST처럼 손 끝을 찔러서 스포이드 같은 걸로 피모아서 어떤 키트에 채혈된 혈액을 떨어뜨려서 검사하는데 처음보는 것이라 신기했다.

01-3

간호 활동? 검사 건강에서

3. 오늘 실습을 통해 새롭게 알게 된 것은?

건강증진실에서는 모바일 헬스케어사업을 진행하는데, 6개월간 건강관리해주는 사업으로, 영양관리 및 운동관리를 하며, 보건소 전문가 상담 확인까지 해주는 활동이었다. 처음에 신청인이 오면 인바디, V/S, BST 등을 통해서 기초건강상태를 사정하고 결과에 대해 설명을 통해 현재건강상태를 본인이 알게 되고 모바일 헬스케어에 대해 주의사항 및 설명드리는 것을 보았다. 기초대사량 구하는 방법을 알게 되었다.

[남성 : 66 + (13.7 × 체중kg) + (5×신장cm) − (5.8×연령)
여성 : 66.5 + (9.6 × 체중kg) + (1.7×신장cm) − (4.7×연령)]

1-3

4. 오늘 실습을 통해 새롭게 배운 것들이 향후 간호실무에 어떻게 적용될 수 있겠는가?

일단, 건강증진실에서 처음에 기본적인 검사(V/S, BST, 인바디, 혈액검사 등)을 통해 그결과를 신청인에게 알려드려야 하기 때문에 각 검사의 정상수치와 비정상 수치시 어떤 문제·증상이 일어날 수 있는지 알아야겠다고 생각했다. 중성지방은 150mg/dL 이상 비정상이고 HDL(일명, 좋은 콜레스테롤)은 높을수록 좋지만 최소 남성은 40mg/dL이상, 여성은 50mg/dL이상이여야 한다고 한다.

1-3 #간호활동

5. 오늘 실습에서 좋았던 점과 아쉬웠던 점은 무엇인가?

집담회에서 지역사회 간호과정하는 것이 막막하였는데, 코멘트를 받아 보완하는, 가이드라인과 같이 이해가 가며 잘 보완해야겠다고 생각했다. 건강증진실 실습도 살짝 건강검진센터 느낌이 들었고 간호사와 영양사, 운동관리사, 코디네이터 등 여러 직업군이 있다는 것을 알게되었다.

CamScanner로 스캔하기

P12

1. 오늘 실습경험의 의미 있는 점은 무엇인가?

<접수실>
오늘은 접수실에서 실습하는 날이어서, 접수실에 앉아 보건소에 오시는 민원인? 시민분들이 어떤 일로 오셨는지, 업무를 보러 가야할 곳이 몇층, 어디인지 모를 때 여쭤보시면 안내해드리는 역할을 하게 되었다. 보건소에 어떤 부서들이 있는지, 더 확실하게 알게 된 것 같았다.

2. 오늘 실습을 하는 동안 나의 생각과 느낌은 어떠했는가?

보건소 입구쪽에 앉아 너무 정면이어서 약간 부담스러웠던 것을 제외하고는 괜찮았다. "민원 안내서"가 있어서 민원인분들이 찾는 부서를 안내해드리면 되지만, 내가 직접 가보지 않은 부서를 안내해드리려 하니 '이쪽이 맞나?' 싶은 생각이 들었고 방향이 써있었는데 내기준·민원인기준으로 쓰여 있어서 (내가 방향치이기도 하고) 헷갈렸다. 지도도 같이 있었으면 하는 바램이 있었다. 생각보다 많은 사람들이 보건소에 방문하여 보건증·예방접종(폐렴 등)을 한다는 것을 알게되었다.

1-1

#보건소 인식

3. 오늘 실습을 통해 새롭게 알게 된 것은?

접수실에서 '민원안내서"를 보며 보건소에는 여러부서들이 있고 그말은 바가 다 다르다는 것을 알게되었다. 1층 접수실에서는 번호표를 뽑고 보건증·채용건강검진·A·B형을 접수하는 일을 담당하였고, 예방접종실에서는 폐렴·자궁경부암 등 예방접종을 담당하였다. 또, 1층에는 결핵관리실, 치매안심센터·건강증진센터(인바디) 부서들이 있고, 2층에는 정신건강상담을 하는 정신건강실, 어린이 놀이터?, 어르신·장애인 진료를 하는 구강보건센터, 물리치료실 등의 부서들이 있었으며, 3층에는 암·난임희귀난치·영유아검진 지원관련 사업하는 건강관리과와 병·의원 관련 신고를 하는 보건사업과 의약계, 전 영업 관리계가 있다.

(치매상담)
(결핵상담)

4. 오늘 실습을 통해 새롭게 배운 것들이 향후 간호실무에 어떻게 적용될 수 있겠는가?

보건소에 어떤 부서들이 위치하는 지 알게 되었고 그 부서들의 업무 또한 간략하게 알게되었다. 저번에도 느낀 것이지만, 각 부서에 간호사들이 배치되어 있어 간호사가 꼭 병원의 간호사 뿐만 아니라, 보건소에서 방문건강관리실·건강증진실·예방접종실 등 여러부서에서도 일을 할 수 있다는 것을 알게되었다. 그 전에, 병원에서의 경험이 있다면 더 좋은 것 같다는 생각을 하게 되었고, 병원에서 경력을 쌓고 보건소에 취업하고 싶다는 생각이 들게되었다.

진로 선택

5. 오늘 실습에서 좋았던 점과 아쉬웠던 점은 무엇인가?

사실, 보건소에 가본 경험이 별로 없어서 (초등학생 때 예방접종 맞으러, 코로나19 검사하러 총 2번 가본 경험이 있다) 보건소에서 어떤 업무를 하는지 잘 몰랐었는데 이번 기회로 알게 된 것 같다. 그리고 보건소에 꽤 많은 사람들이 방문하여 업무(보건증·예방접종)을 본다는 사실을 알게 되었고, 내가 모르던 것들을 알게되어 좋은 경험이 된 것 같다. 접수실에서 실습하면서 민원인들을 안내하는 방법을 조금이나마 알게 된 것 같다.

#보건소 인식

1-1

☆

P12
#팀활동

1. 오늘 실습경험의 의미 있는 점은 무엇인가?

<치매분소>
치매안심센터는 사회복지사, 간호사, 작업치료사 이렇게 협력하여 운영하고 있다고 한다. 치매검사를 하시는 어르신을 지켜보며 치매검사가 이루어지는 과정을 관찰하게 되었고 치매안심센터에서 검사(조기검진) 및 지원서비스 등 여러 업무를 한다는 것을 알게되었다.

2. 오늘 실습을 하는 동안 나의 생각과 느낌은 어떠했는가?

3-2
#전문직 의사소통

어제 접수실 실습할 때도 느낀건데, 생각보다 치매검사를 받으시는 분이 많다고 느꼈는데, 오늘 치매안심센터에서 실습하면서 꽤 많은 분이 치매검사 및 치매상담을 받으신다는 것을 알게되었다. 대상자분들이 60세이상 어르신분들이라 상담하시는 분이 목소리를 크고 또박또박한 발음으로 천천히 말씀하시는 것을 보며 대상자가 어떤 연령이냐에 따라 대상자의 눈 높이에 맞춰서 대화·상담을 진행해야겠다고 생각하였다. 치매검사인 인지선별검사(CIST)는 1:1로 지문을 읽고 답하는 형태로 상담하는데 대략 10분정도 소요되는 것 같았다.

3. 오늘 실습을 통해 새롭게 알게 된 것은?

치매안심센터에서는 상담·등록, 조기검진, 인식개선, 프로그램 등으로 업무를 맡고 있다. 상담은 60세 이상 어르신, 치매환자, 보호자를 대상으로 하며. 등록은 환자 등록 및 서비스 연계 업무를 한다. 서비스로는 치매치료비 지원서비스(치매진료비 및 약제비 지원), 조호물품 지원 서비스(거동이 불편하신 분께 기저귀, 물티슈 등 조호물품 제공), 사례관리서비스(방문, 전화상담으로 대상자의 약 관리 및 필요서비스 연계), 실종예방서비스(지문등록, 배회인식표배부)가 있고 조기검진으로는 인지선별검사(CIST)를 실시하여 인지저하시 정밀검사(SNSB)를 실시하여 결과가 경도인지장애시 1년마다 재검사하거나 치매의심시 감별검사를 연계하여 MRI를 검사하게된다.

4. 오늘 실습을 통해 새롭게 배운 것들이 향후 간호실무에 어떻게 적용될 수 있겠는가?

치매안심센터에서 여러활동을 한다는 것을 알게되었고. 제일 관심있게 본 활동은 인지선별검사(CIST)로 치매검사 하는 것이였다. 인지선별검사(CIST)은 치매검사여서 병원에도 어르신 연령대가 많고 치매나 인지장애 있으신 분들이 많을 것 같아, 알아두면 도움이 될 것 같다고 생각했다. 학년/연세에 따른 기준 점수에 의하여 정상과 인지저하로 나뉘며 인지저하시, 의사가 진행하는 정밀검사(SNSB)를 진행하여 더 확실한 결과를 도출할 수 있다고 한다.

5. 오늘 실습에서 좋았던 점과 아쉬웠던 점은 무엇인가?

#보건 [illegible]

치매안심센터에서 실습하면서, 노인요양등급을 받으려면 국민보험공단을 가야한다는 것은 알고 있었으나 보건소안에 치매검사와 상담을 해주는 치매안심센터가 있다는 점을 처음 알게 되었다. (부모님과 친구들한테도 이야기 해보니 처음알게 되었다고 한다.) 아직 모르는 사람이 많은것 같으니 약간의 홍보가 필요한 것 같았다. 그리고 상담해주시는 분들이 다 친절하고 상냥하셔서 나도 저런점을 본받아 환자와 대상자에게 친절하게 대해드려야겠다고 생각했다.

CamScanner로 스캔하기

1. 오늘 실습경험의 의미 있는 점은 무엇인가?

<예방접종실>
예방접종실에서 처음 실습으로, 예방접종에는 무료/유료가 있고, 영유아부터 65세 어르신까지 연령별로 예방접종 주사 종류가 다르다는 것과 보건소에서 하는 예방접종 종류들을 알게되었다. 그리고 예방접종을 하시려는 분이 오시면 신분증, 예진표를 작성을 도와드리며 응대하는 방법들을 알게 되어서 대상자를 대하는 법을 더 배우게 된 것 같다.

2. 오늘 실습을 하는 동안 나의 생각과 느낌은 어떠했는가?

접수실에서 실습할 때, 폐렴구균 예방접종을 맞으러 오시는 분들이 많이 있으셨는데, 오늘 예방접종실 오전에는 어르신 2분과 영유아 2명이 와서 예방접종을 받으셨다. 보건소에 생각보다 많은 외국인들이 온다는 것을 알게 되었고 외국인을 응대할 수 있는 인력도 필요할 것 같다는 생각과 세계공용어인 영어를 배워서 의사소통으로 설명을 할 수 있을 정도의 수준으로 자기개발을 하여야겠다고 생각했다. 그리고 접종시 주의사항과 예방접종의 설명 등 전문적지식을 쌓고 설명할 정도의 수준이 되도록 노력해야겠다고 생각했다.

3. 오늘 실습을 통해 새롭게 알게 된 것은?

예방접종실에서 실습하면서, 무료접종과 유료접종이 있다는 것을 알게되었고, 독감(인플루엔자) 예방접종은 9~10월부터라서 지금은 비시즌이어서 없다고 하셨고, 보통 영유아 2~4개월과 만65세 이상 어르신을 대상으로 한 예방접종이 많다고 하셨다. 만 65세 이상 어르신들은 폐렴 접종하시러 오시는데, 23가와 13가 두 종류가 있어서 만성질환자나 면역저하자이신 분은 13가 먼저 맞으시고 1년 후 23가로 맞으시는 것을 권장하고 건강한 어르신이라면 23가를 권장한다고 하셨다. 그리고, 자궁경부암 예방접종은 만12세 여성 청소년을 대상으로 6개월 간격 2회 접종을 지원을 한다고 한다.

4. 오늘 실습을 통해 새롭게 배운 것들이 향후 간호실무에 어떻게 적용될 수 있겠는가?

보건소의 예방접종실에서 A, B형 간염 예방접종 뿐만아니라, 영유아 예방접종부터 65세 이상의 어르신의 폐렴구균까지 여러 접종을 한다는 사실을 알게되었다. 그리고, 예방접종 하기 전 예진표를 작성하여 오늘 상태와 BT측정하고 복용하시는 약 등을 사정하는 것이 중요하며 폐렴구균은 어떤주사이고 몇 년에 1번씩 맞아야 하며, 예방접종 후 뻐근하거나 살짝 열이 날 수 있음 등 주의사항에 대해 설명드리는 것이 중요하며 가이드라인을 배운 것 같다.

5. 오늘 실습에서 좋았던 점과 아쉬웠던 점은 무엇인가?

예방접종실 실습은 '어떨까..'라고 생각하며 실습에 임하였는데, 접수실에선 예방접종관련해서 여쭤보시는 분이 많아서 바빠보였는데 막상 오늘 실습을 하니 오전에 총 4-5분정도 오셔서 응대해드렸다. 어르신이나 성인, 청소년들이 많을 것으로 예상되었지만, 의외로 영유아 연령대도 있다는 것과 외국인 부모님과 아이도 와서 예방접종을 하러 와서 외국인도 꽤 많다는 것을 느꼈다.

의사소통 다문화

3-2

전문적 가이드라인

다문화

3-2

CamScanner로 스캔하기

P 12

1. 오늘 실습경험의 의미 있는 점은 무엇인가?

<건강보험공단>

건강보험공단(군산)이 어디에 있는지 알게되었고, 건강보험공단이 하는 일에 대해 알게되었다. 대부분은 행정업무와 전화업무를 하시고 외근 나가시는 날도 있어보였다. 대부분 외라벨이 있어보였고 다들 친절하여서 건강보험공단에 취업하고 싶다고 생각이 들었다. 그리고 국가건강검진 외에도 산재보험 등 보험관련 업무도 하신다는 것을 알게되었다.

#진로선택

2. 오늘 실습을 하는 동안 나의 생각과 느낌은 어떠했는가?

처음으로 군산보건소가 아닌, 건강보험공단 군산지사를 가게 되었다. 골목길에 있어서 위치를 잘 못찾을 뻔 하여서.. 아무래도 익숙하지 않은 곳이라 그런 것 같다. 건강보험공단의 분위기는 좋아보였고, 다들 행정업무와 전화업무를 중심으로 일을 하시는 것 같았다. 특히, 전화로 민원인(?) 상담하시며 필요한 서류제출 등 설명하시는 것을 보게되었다. 병원보다 보건소나 건강보험공단에서의 업무가 편해보였고, 더 알고 싶고 흥미가 생겼으며 여기에 취업하고 싶다는 생각이 들었다.

#진로선택

3. 오늘 실습을 통해 새롭게 알게 된 것은?

건강보험공단은 장기요양팀과 보험급여팀으로 나뉘어져 있으며, 국가 6대암 검진을 담당하고 있었다. 대장암검진은 1년주기로, 50세 이상 대상자를 중심으로 하고, 2년 주기로 유방암 검진은 40세이상 여성, 위암검진은 40세이상 대상자로, 자궁경부암 검진만 20세 이상여성으로 6대암검진중 제일 연령이 낮았다. 그리고, 고위험군 기준해당으로 간암은 6개월 주기 / 폐암은 2년 주기로, 간암의 고위험 기준은 간경변증, B·C형 간염 항원·항체 양성이거나 만성 간질환자를 대상으로 하며 폐암검진은 54~74세 폐암발생 고위험군으로 2년내 국가건강검진시 현재 흡연중이며 하루 한갑기준 30년이상 대상자라고 한다.

4. 오늘 실습을 통해 새롭게 배운 것들이 향후 간호실무에 어떻게 적용될 수 있겠는가?

1-2

건강보험공단을 통해 국가암검진이 이렇게 많다는 것을 알게되었고 병원은 이미 걸린 질병의 치료이지만, 건강보험공단은 질병의 조기발견을 위해, 질병을 예방하기 위한 건강검진을 중요시한다고 생각이 들었으며 둘 다 의료와 관련된 기관이지만, 업무는 확실히 다르다고 느꼈다. 대상자에게 건강검진의 중요성을 알리며 건강검진을 받도록 노력하는 모습들을 보며 본인도 저렇게 대상자를 중심으로 간호중재를 적용하는 간호사가 되어야겠다고 생각했다.

#보건소인식 예방중재

#전문직

2-1

5. 오늘 실습에서 좋았던 점과 아쉬웠던 점은 무엇인가?

건강검진의 중요성을 알았으며, 본인도 올해 건강검진대상자인데, 건강검진을 미루지 말고 미리미리 받아야겠다는 생각이 들었다. 병원에서만 실습하다가 지역사회실습으로 보건소와 건강보험공단에서 실습을 하니, 간호사의 길은 많다는 것을 느꼈고 굳이 병원만 생각하지 말고 넓은 방면으로 보아야겠다고 생각했다. 지역사회실습을 통해 새로운 미래(?)를 설계해보며 노력을 해야겠다고 생각했다.

#진로선택

CamScanner로 스캔하기

P12

1. 오늘 실습경험의 의미 있는 점은 무엇인가?

<건강보험공단>

오늘은 집담회가 있어서 오전에만 건강보험공단 실습을 하였다. 어제는 OT와 건강보험공단에 대한 정보 및 채용관련한 것들을 듣고 전화상담(?)을 하였고, 오늘도 오전에는 간암 검진 독려전화를 드렸었다. 전화업무는 처음이라 떨렸는데 하다보니 익숙해져서 나중에는 수월하게 한 것 같다.

2. 오늘 실습을 하는 동안 나의 생각과 느낌은 어떠했는가?

처음으로 전화업무를 하는 것이라 긴장되었고 응대하는 업무는 약간 어려운 것 같아서 걱정이 많았지만, 계속 하다보니 괜찮은 것 같았다. 전화업무를 하면서 간암 검진 안내를 기본으로 말씀드렸는데 본인부담금 10%가 얼마정도인지 등 복잡한 내용들은 선생님께 전화를 돌려드렸다. 선생님께서 친절히 설명해주시는 걸 들으면서 나도 저렇게 많은 정보를 알아, 대상자에게 친절하게 설명할 수 있도록 전문지식을 많이 알고 이해하여 노력해야겠다고 생각했다.

3. 오늘 실습을 통해 새롭게 알게 된 것은?

건강보험공단에서 암검진 관련하여 검진을 받으시도록 독려전화를 한다는 것을 처음 알게되었다. 대상자에게 전화를 걸어서 건강보험공단임을 밝히고 간암 검진 대상이신데, 아직 검진을 받지 않으셔서 전화를 드렸다고 설명한 뒤 상반기 검진이 6월 30일 이신데, 가능하신지 여쭤보고 미리 예약해서 검진을 꼭 받아보시라고 말씀드리는 전화드리는 업무를 하였다. 그리고 간암 검진을 포함한 다른 검진도 있다는 것과 검진기관이 어디인지 여쭤보시면 안내문보고 설명해드리는 업무를 하였다.

4. 오늘 실습을 통해 새롭게 배운 것들이 향후 간호실무에 어떻게 적용될 수 있겠는가?

어제 이후로, 건강보험공단에서 부담해주는 암 검진이 6가지이고 그 중 2가지는 고위험군 위주라 보통 일반 성인은 4가지를 하여야 한다는 것을 알게 되었고 연령대가 다르다는 것과 출생연도 홀수/짝수 순으로 나뉜다는 것을 알게되었다. 대상자에게 검진대상자인 경우, 검진을 받을 수 있도록 안내하는 것이 중요하다고 생각이 들었다. 그리고 다른 부분에서 궁금한 점이 있다면 자세히 친절하게 설명 드려야겠다고 생각했다.

5. 오늘 실습에서 좋았던 점과 아쉬웠던 점은 무엇인가?

확실히 병원보다 분위기가 밝고 좋다는 느낌이 들었고, 간식도 챙겨주시며 다들 친절하셨다. 단지, 주변에 아무것도 없어서 점심 먹으러 좀 멀리나가야한다는 점 빼고는 근무환경이 좋아보였다. 취업을 병원쪽만 생각했었는데, 다른 방향으로도 취업을 할 수 있다는 점을 알게되어서 뜻깊은 경험이 된 것 같다.

#의사소통

#진로선택

1. 오늘 실습경험의 의미 있는 점은 무엇인가?

첫 OT를 통해 보건소내에 진행되는 다양한 부서의 프로그램과 각 부서의 활동영역 등을 접하면서 그 동안 알지 못했던 부분들을 알 수 있어 새로웠던 경험이었습니다.

2. 오늘 실습을 하는 동안 나의 생각과 느낌은 어떠했는가?

다양한 프로그램이 각 부서마다 시행되며, 그 전에는 알지 못했던 보건소 내에 많은 업무들을 접할 기회가 생겨 좋았습니다.
사실 간호사로 입사 할 때 내가 과연 보건소에서 일할 수 있는 여건이 조성이 될까 싶어 고민했던 역할은 크게 아니었지만 국민(나를 포함해) 많은 혜택과 이익을 받고 있음을 알 수 있는 기회였습니다.

3. 오늘 실습을 통해 새롭게 알게 된 것은?

- 진드기 매개 감염병 (라임병)

진드기가 사람을 무는 과정에서 나선형의 보렐리아 균이 신체에 침범하여 여러기관에 병을 일으키는 감염질환이다.
<진단검사> 병력 / 신체검진 / 효소면역법, 조직 배양검사
<원인> 배양이 어려운 나선형의 그람 음성균 보렐리아가 원인

4. 오늘 실습을 통해 새롭게 배운 것들이 향후 간호실무에 어떻게 적용될 수 있겠는가?

계절별 바이러스 질환 및 감염병 역학조사 등을 진행하니 예방측면과 다양한 프로그램을 적용하여 대상자 건강증진에 이바지 할 수 있을 것이라 생각한다.

예방활동

5. 오늘 실습에서 좋았던 점과 아쉬웠던 점은 무엇인가?

선생님들께서 친절하게 각 부서마다 시행되는 다양한 교육 및 프로그램들을 소개해주시고 이끌어주셔서 많은 것을 보고 배울 수 있는 기회였습니다.

CamScanner로 스캔하기

인식

1. 오늘 실습경험의 의미 있는 점은 무엇인가?

치매안심센터라는 부서에서 시행되어야 하는 프로그램과 다양한 사업진행 사항들을 새롭게 알수 있는 기회였으며 평소 쉽게 접할수 없었던 것들을 알아 볼수 있는 기회였습니다.

2. 오늘 실습을 하는 동안 나의 생각과 느낌은 어떠했는가?

치매안심센터라는 사업부서가 어떤 일을 기획하고 이행하는지 지역사회간호학 과제를 접하면서만 배웠지 현장은 어떤 상황인지 무지했습니다. 하지만 오늘 실습을 통해 어르신들을 위해 프로그램을 기획하고 운영하는 등의 모습을 보며 미처 알지 못했던 부분까지 접할 수 있는 좋은, 기회였습니다.

3. 오늘 실습을 통해 새롭게 알게 된 것은?

• 치매안심센터 주요 서비스
- 치매예방교육 : 치매의 원인, 증상, 예방
- 조기발견 검사 : 치매조기 발견검사
- 사례관리 : 치매환자와 가족의 삶의 질을 향상시키기 위해 사례관리 제공
- 돌봄 / 교육 / 홍보

4. 오늘 실습을 통해 새롭게 배운 것들이 향후 간호실무에 어떻게 적용될 수 있겠는가?

대상자에게 적용되는 사항들을 정확히 숙지하기 위해 반복적인 학습이 필요로 요구되며 그들과 그들의 지인 · 가족 들의 삶의 질향상에 도모할 수 있는 의료인으로 성장 할 수 있어야함 생각합니다.

5. 오늘 실습에서 좋았던 점과 아쉬웠던 점은 무엇인가?

프로그램 실행 날짜가 아니라 대상자에게 적용되는 사항들을 직접적으로 관찰하지 못해 아쉬웠지만 궁금한 사항이 있거나 해당 부서에 전반적인 내용에 관련하여 최대한 학생들에게 알려주시려는 모습을 보며 너무 감사했습니다.

CamScanner로 스캔하기

P13

1. 오늘 실습경험의 의미 있는 점은 무엇인가?

방문간호 현장을 선생님과 함께하며 대상자를 사정하고 의사소통을 통해 그들을 이해하고 관리하는 모습을 보며 미처 알지 못했던 영역에 여러 사항들을 관찰하고 학습 할 수 있는 기회였습니다.

지역사회 간호사 활동
사정

2. 오늘 실습을 하는 동안 나의 생각과 느낌은 어떠했는가?

혈당측정 방법에 관련하여 제대로 인지하지 못하고 미숙한 대상자 분이 계셨습니다. 그들은 같은 말을 반복하고 이해하고 있지 못하는 상태에서도 차분히 한걸음 뒤에서 그들을 기다리며 그들이 느끼는 불안과 스트레스 등을 사정하고 정서적 지지간호를 하는 모습을 관찰 할 수 있었습니다 이를 통해 여태껏 실습을 하면서 미처 관찰하지 못 한 다양한 영역을 학습할 수 있었던 뜻깊었던 기회였습니다.

3. 오늘 실습을 통해 새롭게 알게 된 것은?

방문간호를 통해 신체기능관리 (활동범위가 제한된 이들의 운동이나 스트레칭을 도와줌으로써 관절의 굳음을 예방하고, 근력을 강화시키는 운동을 진행 및 영양관리, 안심콜 등의 관리) 등을 다방면으로 시행하며 방문간호에 대해 정확히 알지 못했던 부분을 학습 할 수 있었습니다.

4. 오늘 실습을 통해 새롭게 배운 것들이 향후 간호실무에 어떻게 적용될 수 있겠는가?

대상자가 필요로 요구하는 것들이 무엇인지, 그것들이 적용되는 이유들을 알아보기 위해서는 정확한 대상자 파악을 통해 건강증진 도모에 이바지 할 수 있으리라 생각합니다.

사정

5. 오늘 실습에서 좋았던 점과 아쉬웠던 점은 무엇인가?

오전/오후 총 5번의 방문간호를 통해 각기 다른 성향을 가진 대상자를 사정하고 그들이 필요로 요구하는 사항들을 충족하며 삶의 질을 높이는데 기여하시는 여러 선생님들을 보며 많은 것을 보고 배울 수 있는 기회여서 좋았습니다.

CamScanner로 스캔하기

1. 오늘 실습경험의 의미 있는 점은 무엇인가?

국민건강보험공단에 출근하여 검진이 필요한 대상자들을 위주로 전화상담 등을 하는 실습을 통해서 공단에서 진행하고 있는 다양한 사업과 여러 업무들을 접할 수 있어 좋았던 경험이었습니다.

2. 오늘 실습을 하는 동안 나의 생각과 느낌은 어떠했는가?

부서에서 진행하는 업무중 간단한 전화응대 정도만 진행했는데도 불구하고 어려운 점들이 있었지만 거기계신 선생님들은 수월하게 진행하시는 모습을 보며 나는 아직 성장할 길이 멀구나 생각이 들었습니다.

3. 오늘 실습을 통해 새롭게 알게 된 것은?

· 국민건강보험 공단은 보험자인 위탁집행형 준정부기관과 그 외 나머지 4대보험의 수납도 담당하고 있다. / 국민의 질병 부상에 대한 예방 진단 · 치료 재활과 출산에 대하여 보험급여를 실시함으로써 국민보건을 향상시키고 사회보장을 증진한다.

4. 오늘 실습을 통해 새롭게 배운 것들이 향후 간호실무에 어떻게 적용될 수 있겠는가?

국민들이 적용받는 다양한 혜택들을 알아 볼 수 있었으며 생을 살아가면서 병원진료, 다양한 의료혜택이 우리의 삶의 얼마나 많은 부분을 차지하는지 알 수 있었던 기회였습니다.

5. 오늘 실습에서 좋았던 점과 아쉬웠던 점은 무엇인가?

지금껏 실습을 해보지 못했던 부서들을 돌아가며 각 부서별로 어떤업무를 진행하는지 관찰하고 경험해서 많은 것을 알수 있었던 기회였습니다.

CamScanner로 스캔하기

1. 오늘 실습경험의 의미 있는 점은 무엇인가?

금연상담센터에 방문하여 (흡연, 음주) 등으로 일상생활이 어려운 이들의 삶의 질 향상을 위한 올바른 가치관 성립과 다양한 물품 사용 및 프로그램 운영을 통해 다방면으로 대상자를 고려하는 모습을 보며 새로운 점을 배울 수 있어 좋았습니다.

2. 오늘 실습을 하는 동안 나의 생각과 느낌은 어떠했는가?

흡연으로 힘들어하시는 60-70대 여성분이 방문하셨는데, 흡연을 하는 본인의 모습이 창피하다며 어디 말할 곳도 없다는 식으로 자책하는 모습을 보았습니다. 대상자분들의 삶과 상황을 직접적으로 알아볼 수 있었던 계기였습니다.

3. 오늘 실습을 통해 새롭게 알게 된 것은?

· 금연은 왜 필요한가?

흡연은 심장질환 (협심증, 심근경색) 등뿐만 아니라, 뇌졸중 / 뇌경색 폐기종 같은, 천식 만성폐쇄성 질환에 원인이 된다.

4. 오늘 실습을 통해 새롭게 배운 것들이 향후 간호실무에 어떻게 적용될 수 있겠는가?

이러한 이론적 사항들을 사전에 학습하여 대상자가 필요로 할 때 적용하여 그들의 삶의 질 개선에 큰 도움이 되리라 생각합니다.

5. 오늘 실습에서 좋았던 점과 아쉬웠던 점은 무엇인가?

다양한 프로그램 활동을 함께 하지는 못했지만 대상자들이 어려움을 직면하여 함께 나은 방법을 모색하려는 선생님들의 모습을 보며 감명 받았습니다.

CamScanner로 스캔하기

1. 오늘 실습경험의 의미 있는 점은 무엇인가?

오늘은 건강검진 사업팀으로 보건소에서 진행하는 다양한 프로그램과 사업에 대해 알게 되었다. 병원에서 일하는게 아니라 보건소라서 국가 사업관련 알수 있는 기회가 되었는 많이 알게 되었다.

2. 오늘 실습을 하는 동안 나의 생각과 느낌은 어떠했는가?

건강증진 사업 팀으로 군보건에서 진행하는 군산시 국민 체력 증진 걷기로 대상자를 모집해서 헬스를 정해진 시간에 제공하여 헬스로 하고 은파호수 공원에 오전에 10시에 모여서 체조나 산책로 진행하는 프로그램이 있다. 보국가에서 생각보다 많은 사업을 진행한다는걸 알게되었다.

3. 오늘 실습을 통해 새롭게 알게 된 것은?

국가 건강검진 : 20세이상 2년마다 진행하고, 6대 암검진을 제공한다

자궁경부암검사 : 20세이상 여성 2년마다, 유방암검사 40세 이상 여성 2년마다.

위암검사 : 남여 40세이상 2년마다. ~~대장~~암 : 40세이상 남여 2년마다

간암 : 40세 이상 남녀 (고위험군) 6개월마다. 폐암 : 54~74세 남.녀 (고위험군) 2년마다 건강검진이 진행된다고 한다.

4. 오늘 실습을 통해 새롭게 배운 것들이 향후 간호실무에 어떻게 적용될 수 있겠는가?

병원이 아니라 다양한 사업종류를 알게 되었고. 향후 국가 관련 기관에서 일을 한다면 이런 국가사업이 도움이 많이 될거 같다고 생각한다.

5. 오늘 실습에서 좋았던 점과 아쉬웠던 점은 무엇인가?

새로운 사업관련해서 알게되었고 헬스로 직접 참여서 재미있었다. 아쉬웠던 점은 대상자가 많지는 않았던거 빼고는 좋았습니다.

CamScanner로 스캔하기

실습일자 : 2023년 8 월 29일

1. 오늘 실습경험의 의미 있는 점은 무엇인가?

백신의 금기사항은 백신을 접종받은 사람에게 심각한 부작용이 발생할 가능성이 아주 높은 경우이며, 이는 백신자체의 문제가 아니라 백신을 접종받는 사람의 상태를 말하며 금기 사항이 있는 경우에는 백신을 접종 하면 안된다. 또는 임신, 면역저하로 해당됨을 알게 되었다.

2. 오늘 실습을 하는 동안 나의 생각과 느낌은 어떠했는가?

보건소에서 유료접종, 무료접종 등 많은 예방접종 사업을 하는것으로 알게되었고 군산시에 거주하는 사람을 대상으로 진행되며 백신에 따라 연령이 포함되는 사람에게 백신을 제공한다. 보건소에 생각보다 많은 사람들이 백신을 맞으러 온다는걸 알게됨.

3. 오늘 실습을 통해 새롭게 알게 된 것은?

백신에 의해 알레르기 반응이 생길 수 있는데 이는 백신에 포함된 물질인 세포배양재료, 안정제, 보존제, 세균의 증식을 막기 위한 항생제 등에 의해 발생할 수 있고, 심한 알레르기 반응은 생명을 위협할 수 있지만 빈도는 드물다.

의료진들은 모든 알레르기 반응, 아나필락시스 등에 발생 시 대처할 수 있어야한다.

4. 오늘 실습을 통해 새롭게 배운 것들이 향후 간호실무에 어떻게 적용될 수 있겠는가?

백신의 종류도 많이 알게되었고, 시에서 운영하는 무사업으로 무료 백신, 유료백신도 알게되었고 백신에 따른 부작용 발생에 대해 알게되었다. 추후에 보건소 같은 국가 기관으로 가게 된다면 도움이 될거 같다는 생각을 했다.

5. 오늘 실습에서 좋았던 점과 아쉬웠던 점은 무엇인가?

보건소 안에 예방접종 관련 사업을 알게되었고 생각보다 업무는 간단하고 괜찮았던거 같다. 그 외에 아쉬웠던 점은 없고 잘 알려 주셔서 좋았습니다.

CamScanner로 스캔하기

1. 오늘 실습경험의 의미 있는 점은 무엇인가?

군산보건소에서 오늘은 치매 안심센터로 출근 하였다. 군산시에서는 치매 등록 관리서비스, 치매조기 검진서비스, 치매환자 서비스, 치매가족 지원서비스 치매 예방서비스, 치매 친화적 지역사회등 다양하고 많은 사업을 추진하고 있으며 다양한 프로그램을 진행 한다는걸 알게되었다

2. 오늘 실습을 하는 동안 나의 생각과 느낌은 어떠했는가?

군산에서는 치매 관련 사업을 많이 추진하는것도 알게되었고 대상자들이 직접찾아와 등록하고, 검진을 진행하였다. 군산시에 치매를 가지고 계신 분들도 않았고 육안으로 봤을때 전혀 예상하지 못할 대상자들도 많았다. 찾아보니 전북에서 치매율이 증가함을 알게되었고, 많이 알게되었다.

3. 오늘 실습을 통해 새롭게 알게 된 것은?

지역사회에 거주하는 어르신의 인지건강 상태에 따라 요구되는 치매 조기검진을 통해 발견하고, 관리함으로서 어르신의 삶의 질을 높이고자하고, 조기검진을 실시하며 치매 및 치매 고위험을 관리하고 가족들의 삶의 질 향상을 도모하며 스트레스 해소 및 정서적 고통과 부담을 경감하도록 프로그램을 진행하는 사업을 한다.

4. 오늘 실습을 통해 새롭게 배운 것들이 향후 간호실무에 어떻게 적용될 수 있겠는가?

나중에 관련 업무를 하게된다면 프로그램 계획을 하고 프로그램에 참여하고 어느정도 실무에 적용할 수 있을거라 생각한다. 보건소에서 치매 안심센터에 보내주어서 더 다양하게 알고 갈 수 있어서 좋았고, 실무에 적용할 수 있을거 같다.

5. 오늘 실습에서 좋았던 점과 아쉬웠던 점은 무엇인가?

보건소 뿐아니라 치매안심센터로 경험할 수 있어서 좋았고 프로그램에 직접 참여하여 배울 수 있어서 좋았다. 그리 아쉬웠던 점은 없다.

CamScanner로 스캔하기

1. 오늘 실습경험의 의미 있는 점은 무엇인가?

오늘은 모자보건센터에서 일을 진행했다. 보건소에서 매번 다른 부서로 배치해줘서 그 부서마다 특징과 하는일에 대해 알수 있어서 좋았고 모자보건 관련해서도 많이 알게되었다.

2. 오늘 실습을 하는 동안 나의 생각과 느낌은 어떠했는가?

군산시에서 모자보건 사업을 다양하게 추진하고 있어서 많은 분들이 찾아왔고, 출산·육아지원 제도라 에서 출산전 후 휴가급여, 육아기 근로시간 단축급여, 배우자 출산 휴가 급여, 육아 휴직급여, 출산 육아기 고용안정 장려금 등 많은 사업을 추진하고 우리나라 출산율 저하때문인지 혜택도 많이 느낀거 같다.

3. 오늘 실습을 통해 새롭게 알게 된 것은?

모자보건중, 고위험산모 가형아 검사비지원, 산모 특실이 지원, 산모 산전 산후 초음파지원, 산후키트 지원, 산모·신생아 산후관리 교육 및 상담, 신생아 난청지원, 혈액형 검사비 지원, 엽산제, 철분제 지원, 산모, 신생아 건강관리 지원, 출산 진료비 지원, 난임부부 시술비 지원 등 아이를 키우는데, 아기를 임신·출산 제도가 다양함을 알게됨.

건강증진 활동

4. 오늘 실습을 통해 새롭게 배운 것들이 향후 간호실무에 어떻게 적용될 수 있겠는가?

관련된 곳에서 일하게 된다면 그래도 어느정도의 사업이 추진되고 어떤 혜택과 지원방법등 설명하고 홍보할 수 있을거 같고 산부인과 같은 곳에 일하게 된다면 지원 하는 제도에 대해 잘 알고 알려줄 수 있을거 같다.

5. 오늘 실습에서 좋았던 점과 아쉬웠던 점은 무엇인가?

다양한 제도에 대해 알게되었고, 일하는걸 관찰할 수 있었다. 아쉬웠던 점은 없었고, 보건소는 좋은 사업들을 하는거 같아 좋았습니다.

CamScanner로 스캔하기

1. 오늘 실습경험의 의미 있는 점은 무엇인가?

오늘은 센터에서 방문간호 담당하는 부서에서 일하게 되었다
군산 나운동 지정을 담당하는 분과 방문간호를 직접 나가게 되었다.
처음 접해보는 방문간호여서 의미 있었던거 같았다.

2. 오늘 실습을 하는 동안 나의 생각과 느낌은 어떠했는가?

방문간호시 대상자의 혈당관리, 혈압관리등을 진행했고 개인
방문도 하지만 아파트내에 경로당에 들러 단체로
건강확인도 하고 건강한 식습관등을 안내도 진행하였다.
방문간호는 개인만 해당인줄 알았는데 단체로가능 하다는걸 알게되

3. 오늘 실습을 통해 새롭게 알게 된 것은?

오늘은 방문간호를 통해 대상자 관리를 진행했고,
방문간호를 혼자하기에는 위험할 수도 있다는 생각도 들었다.
국가에서 많은 사업을 진행하고 있는데 좀 더
홍보가 많이되어서 다양한 사람이 접했으면 했다.

4. 오늘 실습을 통해 새롭게 배운 것들이 향후 간호실무에 어떻게 적용될 수 있겠는가?

추후 방문간호업무와 관련된 업무를 하게 된다면
처음하는거 보다는 어떤 형식으로 사업이 진행되는지
먼저 알고 향후 간호실무에 적용하는데 도움이 될
수 있을거라 생각한다. 방문간호는 좋은 사업 같다.

5. 오늘 실습에서 좋았던 점과 아쉬웠던 점은 무엇인가?

실제로 집을 방문해서 하는 간호라 처음 접하기도
해서 신기했고 좋았지만 혼자하게된다면 조금은
위험할 수도 있다는 생각을 하게 되었다.

CamScanner로 스캔하기

1. 오늘 실습경험의 의미 있는 점은 무엇인가?

군산시보건소에 관련된 OT와 국민건강보험공단, 치매안심센터, 진드기감염병예방관리에 대해 교육을 들었던 것이 의미있었다. 국민건강보험공단에서 노인장기보험에 대해 자세히 교육받은 것이 수업시간에 배운 내용도 복습되면서 의미있었다. 또 국민건강보험공단에서 주관하는 업무 중 국가건강검진에 대해 교육을 들어 암 검진에 대한 내용을 자세히 알게되어서 의미있었다. 그리고 진드기 감염병에 대해서는 잘 모르고 있었는데 이번 진드기 감염병 예방관리 교육을 통해 진드기감염병으로 SFTS(중증열소혈소판감소증후군), 쯔쯔가무시, 라임병, 진드기뇌염이 있다는 것을 알게 되었고 보통 날씨가 따뜻해져 야외활동이 많아지는 4월부터 가을까지 많이 발생한다는 것을 알게되어서 의미있었다. 그리고 치매파트너에 대해 알게되고 치매파트너로 가입한것이 의미있었다.

2. 오늘 실습을 하는 동안 나의 생각과 느낌은 어떠했는가?

국민건강보험공단에서 건강보험뿐만 아니라 노인장기요양보험, 국가건강검진 등 다양한 업무를 시행하고 있다는 것을 느꼈고 모든사람들의 건강을 위해 의료보험체계가 잘 이루어져있다고 생각하였다. 그리고 진드기감염병은 요즘 많이 발생하지 않을것이라고 생각했는데 매년 발생률이 증가하고 있다는 말을 듣고 놀랐다. 4월부터 날씨가 따뜻해지면 시골에서 농사일들도 많이 하고 잔디에도 누워서 쉬기 때문에 많이 발생한다는 것을 알고 감염위험이 높은 사람들에게 철저하게 교육하는 것이 중요하다고 생각하였다. 60세 이상 관내 어르신들이 인지선별검사를 하기 위해 많이 찾아와서 검사를 시행하는 것을 보며 60세 이상 어르신들이 치매를 조기발견하기 위해 노력하신다고 느꼈으며 치매가 어르신들에게 두려운 질환이라는 것이라고 생각했다. 더 많은 60세 이상 어르신들이 할 수 있도록 홍보가 잘 되어야 하는 것이 중요하다고 생각했다. 우리나라가 치매발생률이 높아 국가에서 무료로 시행하는 검사와 프로그램, 지원제도가 많다고 느꼈다.

3. 오늘 실습을 통해 새롭게 알게 된 것은?

치매안심센터에서 하는 업무 중 조기검진에 대해 새롭게 알게 되었다. 치매조기검진의 대상자는 60세이상 관내 어르신으로 첫번째로 인지선별검사(CIST)를 시행하도록 한다. 학년, 연세에 따른 기준점수에 의하여 <정상/인지저하>로 나뉘어진다. 검사결과가 정상으로 나왔으면 1년마다 인지선별검사(CIST)를 시행하도록 한다. 검사결과가 인지저하로 나왔으면 정밀검사(SNSB) 안내 및 연계해드린다. 두번째로 정밀검사(SNSB)는 인지선별 실시 결과 <인지저하>가 나오신 분들을 대상으로 실시한다 기억력, 지남력, 주의력, 시공간기능, 집행기능, 언어기능 등의 영역검사를 문답식으로 시행한다. 검사결과 경도인지장애로 나온경우 1년마다 정밀검사(SNSB)를 시행한다. 검사결과가 치매의심으로 나온경우 감별검사를 연계한다. 세번째로 감별검사이다. 감별검사는 병원(신경과)에서 실시하는 치매진단을 위해 MRI 또는 혈액검사를 진행하는것이다. 이 감별검사는 중위소득 120% 이하의 대상자에게는 감별검사비를 지원해준다. 치매예방수칙 3·3·3에 대해서도 새롭게 알게되었다.

4. 오늘 실습을 통해 새롭게 배운 것들이 향후 간호실무에 어떻게 적용될 수 있겠는가?

치매안심센터 업무의 메뉴얼대로 치매 대상자를 관리할 것이다. 일단 조기검진을 시행하여 정상인지, 경도인지장애인지, 치매인지 구분하여 진단받을 수 있도록 도울 것이다. 치매를 진단받는 60세 이상의 대상자, 보호자를 상담할 것이다. 상담 후 대상자를 등록하고 서비스를 연계할 것이다. 치매치료비 지원서비스(중위소득120% 이하 치매환자에게 치매진료비/약제비 지원), 조호물품지원서비스(거동이 불편하신 분에게 기저귀, 물티슈 등의 조호물품 평생 1회 1년 제공), 사례관리서비스(방문, 전화 상담을 통해 대상자의 약 관리 및 필요서비스 연계), 실종예방서비스(지문등록, 배회인식표 배부, 배회감지기 배부)를 연계할 것이다. 그리고 관내노인시설(경로당, 복지관) 및 실외에서 60세 이상 어르신들을 대상으로 치매에 대한 기본 정보 제공 및 인식개선 교육을 실시할 것이다. 프로그램① 치매환자쉼터(미술치료, 원예치료, 운동치료 병행) ② 치매예방교실 ③ 인지강화교실 ④ 치매가족지원교실을 시행할 것이다. 치매예방수칙 3·3·3을 교육하고 직접 수행할 수 있도록 도울것이다.

5. 오늘 실습에서 좋았던 점과 아쉬웠던 점은 무엇인가?

군산시보건소에 대한 OT를 들어 보건소에서 하는 전반적인 일, 각 센터의 업무에 대해 알게 되어서 좋았다. 국민건강보험공단에서 하는 일, 국가건강검진, 국가보험 등에 대해 알게 되어서 좋았다. 또 진드기 감염병의 종류를 알게 되고 감염병의 예방방법과 매개체에 대해 자세히 배우게 되어서 좋았다. 그리고 치매안심센터에서 실습을 하면서 치매안심센터업무메뉴얼을 통해 치매 진단을 위해 시행하는 인지선별검사(CIST), 정밀검사(SNSB), 감별검사에 대해 알게 되어서 좋았다. 또 인지선별검사(CIST)를 실시하는 모습을 직접 볼 수 있어서 좋았다. 치매 대상자를 위한 다양한 지원 프로그램을 알게되고, 치매예방법에 대한 내용도 알게 되어서 좋았다. 아쉬웠던 점은 없었다.

CamScanner로 스캔하기

1. 오늘 실습경험의 의미 있는 점은 무엇인가?

오늘 구강보건센터에서 실습을 하면서 구강보건센터에서 하는 일과 구강센터에서 불소용액을 무료로 지원해주고 있다는 것을 알게 되어 의미있었다. 또 구강보건센터에서 무료로 제공하는 불소용액을 만드는 과정을 직접 관찰하고 불소용액통에 직접 불소용액을 담는 일을 해본것이 의미있었다. 그리고 만성질환에 대해 교육하는 프로그램에 참석하여 당뇨병, 고혈압에 대해 교육을 들으면서 성인간호학 수업 때 들었던 내용도 복습되고 의미있었다. 구강병 예방법의 내용으로 충치예방법, 잇몸병예방법, 시린이 예방법에 대해 알게 되어서 의미있었다. 또 일반인의 구강관리에 대한 내용도 알게 되어서 의미있었다.

2. 오늘 실습을 하는 동안 나의 생각과 느낌은 어떠했는가?

구강보건센터라는 곳을 처음 알게되고 처음 실습을 해보는 곳이라서 궁금했었다. 이번에 구강보건센터에서 실습을 해보면서 감염과 구취를 예방하는 생활 속 구강 관리 수칙, 충치예방법, 잇몸병 예방법, 시린이 예방법, 올바른 칫솔질법등 다양한 매체와 도구를 이용하여 교육을 진행하고 있다고 느꼈다. 그리고 구강보건센터에서 불소양치용액을 무료로 배부해주고 있다는 것을 듣고 나니 아이들과 지역주민분들의 충치예방을 위해 좋은 활동이라고 생각했다. 하지만 아직 많은 사람들이 이용하지 않은 것 같아 불소양치용액을 무료로 제공해준다는 것을 홍보하여 많은 사람들이 불소양치용액을 무료로 받아갈 수 있도록 해야된다고 느꼈다. 구강보건센터에서 실습해보니 지역주민들의 구강건강 및 관리를 위해 보건소에 필요한 센터라는 것을 느꼈으며 다양한 업무를 진행하고 있다는 생각이 들었다.

3. 오늘 실습을 통해 새롭게 알게 된 것은?

구강보건센터에서 대표적으로 하는 일이 노인의치보철사업, 학교구강보건사업 /어린이 충치예방사업, 노인불소도포/스켈링 사업, 장애인 및 취약계층 무료진료, 불소용액양치사업, 구강보건교육/양치체험교실 운영, 미취학어린이구강관리사업이라는 것을 새롭게 알게되었다. 또 3대 구강병인 치아우식증(치아가 삭아 구멍이 생기는병), 부정교합(치아가 가지런하지 못하고 삐뚤삐뚤하거나 한쪽 턱이 너무 나온것), 치주병(치아를 둘러싸고 있는 치아주위조직에 병이 생기는것)에 대해 새롭게 알게 되었다. 그리고 구강병의 특성(①널리퍼져있다. ② 만성병이다. ③누진적이다. ④ 예방이 가능하다.)에 대해 알게 되었고, 충치예방법으로 불소도포(매년 한 두번씩 불소를 치아에 발라준다.), 치아 홈 메우기(어금니의 충치가 생길만한 홈을 치과재료로 미리 메워버리는 방법), 올바른 칫솔법에 대해 새롭게 알게되었고 잇몸병 예방법인 치면세마(스케일링)에 대해 알게 되었다.

4. 오늘 실습을 통해 새롭게 배운 것들이 향후 간호실무에 어떻게 적용될 수 있겠는가?

지역주민들이 구강보건센터에서 하는 업무와 무료로 지원해주는 일들을 알 수 있도록 홍보하며 많은 주민들이 이용할 수 있도록 할 것이다. 지역주민들의 구강건강을 위하여 3대 구강병인 치아우식증, 부정교합, 치주병에 대해 설명하고 충치예방법에 대해 교육하여 잘 관리할 수 있도록 도와줄 것이다. 학교구강보건사업/어린이충치예방 사업을 통해 어린이와 청소년들에게 올바른 양치질과 구강 관리방법을 교육하고 치아우식증이 생기지 않도록 할 것이다. 장애인 및 취약계층은 일반인들보다 구강관리가 더 어려우므로 무료진료 사업에 협조하여 구강관리를 도울 것이다. 치아뿐만 아니라 잇몸병도 예방할 수 있도록 잇몸병에 대해 교육하고 스케일링의 중요성을 알려드려 1년이나 2년에 한번씩 정기적으로 받도록 도울 것이다. #건강형평성

5. 오늘 실습에서 좋았던 점과 아쉬웠던 점은 무엇인가?

구강보건센터에서 하루동안 실습을 하면서 구강보건센터에서 하는 일에 대해 알게 되어서 좋았다. 또 직접 불소용액을 만드는 과정을 관찰하고 불소양치용액 통에 담아 무료로 나눠주는 불소양치용액을 직접 준비해봐서 좋았다. 그리고 구강병, 잇몸병, 구강병의 특성, 올바른 칫솔질에 대해 몰랐던 부분을 자세히 알게 되어서 좋았다. 스케일링의 중요성에 대해서도 알게 되어서 좋았다. 하지만 구강보건센터에 방문하는 사람들이 한명도 있지않고 홈메우기, 불소양치용액을 받으러 오는 사람이 없어 홈메우기를 보지 못하고 불소양치용액을 직접 전달해보지 못해서 아쉬웠다.

CamScanner로 스캔하기

P15

1. 오늘 실습경험의 의미 있는 점은 무엇인가?

오늘은 건강증진실에서 실습하면서 모바일헬스케어에 가입하기 위해 건강증진실에 방문하여 키, 몸무게, 혈압, 혈당, 콜레스테롤, 인바디, 영양 설문을 하고 검사 결과에 따라 영양사와 운동전문가와 상담을 하고 3개월마다 관리되도록 등록하는 것을 직접 관찰한 것이 의미있었다. 또 모바일헬스케어에 대해 알게 된 것이 의미있었다. 그리고 오늘 유치원어린이들이 구강보건센터에 방문하여서 양치하는 것을 도와주고 치과의사선생님이 검진하는 것을 도와주고 충치예방교육을 도와준 것이 의미있었다. 그리고 지역사회조사기간이라서 안내문을 준비한 것도 의미있었다.

2. 오늘 실습을 하는 동안 나의 생각과 느낌은 어떠했는가?

건강증진실에서 진행하고 있는 모바일헬스케어, 화목하게 둘레길 걷기, 헬스, 요가 등 지역주민들의 건강관리를 위한 다양한 프로그램들이 많다고 생각했고 비만, 고혈압, 당뇨 등을 진단받지 않도록 많은 노력을 하고 있고 지역주민들에게 너무 좋은 활동이라고 느꼈다. 나도 모바일헬스케어에 가입하여 주기적으로 키, 몸무게, 혈압, 혈당, 콜레스테롤, 영양 섭취 등을 관리받고 싶다고 느꼈다. 그리고 유치원어린이들에게 애니메이션으로 충치예방 교육을 하고 직접 양치교실에서 올바른 양치법으로 양치를 하도록 하고 치아 검진을 하고 불소까지 도포해주는 것을 보면서 유치원어린이들에게 큰 도움이 될 것이라고 느꼈고 집에서도 실천할 수 있도록, 치실, 불소양치용액, 치약, 양치컵을 선물로 챙겨주는 것을 보면서 좋은 활동이라고 생각했다.

3-3

3. 오늘 실습을 통해 새롭게 알게 된 것은?

모바일헬스케어에 대해 새롭게 알게 되었다. 모바일헬스케어는 지역사회에서 보건소 모바일 헬스케어 플랫폼을 통해 ICT를 활용한 공공형 건강관리 서비스를 제공함으로써 지역주민의 건강수준 향상을 도모하고자 하는 것이다. ICT를 활용한 공공형 건강관리서비스 활성화와 지역주민의 자가 건강관리 능력을 향상하고자 하는 것이다. 만성질환예방을 통한 경제적 손실 최소화와 건강관리의 효율성 제고 필요와 수혜자(주민) 중심의 서비스 건강관리사업으로 전환을 위해 ICT를 활용한 모바일 기반의 건강관리 서비스 제공 필요로 모바일헬스케어를 추진하게 된 것이다. 코디네이터, 의사, 간호사, 영양사, 운동전문가 등 전문인력 5인이 참여한다. 간호사는 수집된 건강정보 및 서비스 이용 확인을 통한 건강 상담 및 관리 목표 설정, 건강수치 이상치 상시 모니터링 및 의사 연계 역할을 한다. 영양사는 온라인 영양 상담·교육, 맞춤정보를 제공한다. 운동전문가는 온라인 신체활동 상담, 교육, 맞춤정보를 제공한다.

팀활동

~~[illegible]~~

다각적

4. 오늘 실습을 통해 새롭게 배운 것들이 향후 간호실무에 어떻게 적용될 수 있겠는가?

모바일헬스케어 사업이 활성화되도록 드시고 있는 약이 없는지 확인하고 사업에 가입할 수 있도록 키, 몸무게, 인바디, 혈압, 혈당, 콜레스테롤 검사를 적극적으로 시행할 것이다. 또 모바일헬스케어에 대해 홍보를 진행할 것이다. 그리고 수집된 건강정보 및 서비스 이용 확인을 통한 건강상담과 대상자와 관리 목표 설정을 할 것이다. 또 건강수치 이상치 상시 모니터링을 하고 대상자를 의사선생님에게 연계해줘서 의사선생님과 상담할 수 있도록 할 것이다. 모바일헬스케어를 통해 지역주민들이 고혈압, 당뇨 등 대사증후군을 진단받지 않고 꾸준히 식단과 운동을 할 수 있도록 전문인력인 의사, 영양사, 운동 전문가, 코디네이터와 협력하여 노력할 것이다.

5. 오늘 실습에서 좋았던 점과 아쉬웠던 점은 무엇인가?

건강증진실에서 실습을 하면서 모바일헬스케어라는 사업을 처음 알게되고 어떤 절차로 어떻게 진행되는지 알게되어서 좋았다. 직접 검사하고 진행하는 모습을 관찰해서 좋았다. 그리고 구강보건센터에 가서 유치원어린이들 대상으로 하는 충치예방 하는 교육을 도와주고 양치하는 것도 도와준 것이 좋았다. 또 치과의사선생님이 구강검진하는 것을 직접 관찰해서 좋았다. 모바일헬스케어 사업에 필요한 전문인력에 대해 알고 전문인력인 의사, 간호사, 영양사, 운동전문가, 코디네이터의 각 역할에 대해 알게 되어서 좋았다. 또 지역사회조사에 대한 내용을 알게 되어서 좋았고 국가암검진에 대해 자세히 알게 되어서 좋았다. 아쉬웠던 점은 없다.

CamScanner로 스캔하기

1. 오늘 실습경험의 의미 있는 점은 무엇인가?

국민건강보험공단에 실습하러 가서 국민건강보험에서 어떤 일을 하는 지 알게 되어서 의미있었다. 그리고 국민건강보험공단에 일하시는 직원분들이 어떤 일을 하는 지 전반적으로 알게되어서 의미있었다. 또, 군산지사 국가건강검진기관들에 대해 알게된 것이 의미있었다. 국가 6대 암 검진에 대해 자세히 알게되어서 의미있었다. 대장암 검진 방법과 자궁경부암 검진, 유방암 검진, 위암 검진에 대하여 알게 되어서 의미있었다. 고위험군 기준 해당 시 검진하는 간암검진, 폐암검진에 대해 알게 되고 자궁경부암 대상자에게 전화해서 건강검진을 격려한 것이 의미있었다.

2. 오늘 실습을 하는 동안 나의 생각과 느낌은 어떠했는가?

많은 국민들이 국가건강검진을 하지 않는다고 느꼈다. 또 국가암검진율도 낮다고 느꼈다. 그래서 국가건강검진과 국가암검진을 받도록 많이 알리고 전화해서 격려하는 것이 중요하다고 생각하였다. 그리고 국민 사망원인1위가 암이므로 해당되는 국민들은 국가암검진을 꼭 받아 미리예방하거나 조기발견, 조기예방하는 것이 중요하다고 생각하였다. 내가 오늘 실습하면서 올해 자궁경부암 대상자인 20대, 30대 여성들에게 자궁경부암 검진 받으시라고 전화하면서 대부분 사람들이 연말에 받을거라고 답하는 것을 보며 연말에는 검진기관에 검진들이 밀려서 검진이 어려울 수 있으므로 미리 예약하시고 검진을 받으시라고 설명해드리는 것이 중요하다고 느꼈다.

예방중심사업

3. 오늘 실습을 통해 새롭게 알게 된 것은?

국가 암검진에 대해 자세한 내용을 새롭게 알게 되었다. 국가 암검진의 검진비용은 국민건강보험공단이 90%, 수검자가 10%를 부담한다는 것을 새롭게 알게 되었다. 건강보험료 하위 50%, 의료급여 수급권자, 대장암, 자궁경부암 검진 대상자는 본인 부담금이 없다는 것을 새롭게 알게 되었다. 2년 주기 암 검진인 자궁경부암, 유방암, 위암 검진은 전년도 미수검자도 공단 신청 시 수검 가능하다는 것을 알게 되었다. 1년 주기로 검사하는 대장암 검진은 50세 이상 남녀가 대상자이며 분변잠혈검사(FOBT)를 통해 검진을 진행한다. 2년 주기로 시행하는 암 검진으로는 자궁경부암, 위암, 유방암이 있다. 자궁경부암은 20세이상 여성이 대상자이고 유방암은 40세 이상 여성이 대상자이다. 위암은 40세 이상 남성, 여성이 대상자이다. 고위험군 기준에 해당 시 간암은 6개월 주기로 검진하고 폐암은 2년주기로 검진을 진행한다.

#건강형평성

4. 오늘 실습을 통해 새롭게 배운 것들이 향후 간호실무에 어떻게 적용될 수 있겠는가?

일반건강검진과 국가 암검진 대상자들이 검진을 받도록 홍보, 안내, 격려할 것이다. 자궁경부암 대상자에게 자궁경부세포검사를 통해 검진을 하는 것이기 때문에 자궁적출술을 받았거나 성경험이 없는 대상자는 검진 대상자에서 제외된다고 안내할 것이다. 그리고 2년 주기 암검진 항목 중 전년도에 검진을 받지 않은 대상자에게는 공단 신청 시 수검이 가능하다고 설명할 것이다. 대장암 검사는 분변잠혈검사를 통해 검진하고 자궁경부암은 자궁경부세포 검사, 유방암검진은 유방촬영, 위암검진은 위내시경검사나 위장조영검사를 통해 검진한다고 안내드릴것이다. 폐암 검진은 54~74세 폐암발생 고위험군이 대상자라고 설명할것이다.

5. 오늘 실습에서 좋았던 점과 아쉬웠던 점은 무엇인가?

국가암검진의 검진비용 부담은 어떻게 되는것인지 알게 되어서 좋았다. 그리고 1년 주기, 2년 주기에 해당하는 암검진들에 대해 알게 되어서 도움이 되고 좋았다. 또 자궁경부암 대상자인 20세이상의 여성에게 직접 전화해서 자궁경부암 검진 받아보시라고 전화통화하고 그에 대한 궁금사항을 답해준것이 재미있고 좋았다. 그리고 간암검진, 폐암 검진에 해당하는 고위험군 기준에 대해 자세히 알게 되어서 좋았다. 국민건강보험공단에서 어떤 일을 주로 하는 지 직접 보고 들어서 신기하고 좋았다. 다양한 업무를 접해보지는 못해서 아쉬웠다.

CamScanner로 스캔하기

1. 오늘 실습경험의 의미 있는 점은 무엇인가?

국민건강보험에 입사하는 방법과 간호학과를 졸업해 간호사 면허가 있는 상태에서 지원할 수 있는 부분에 대해 알게되어서 의미있었다. 그리고 건강직과 요양직 업무의 차이에 대해 자세히 알게 되어서 의미있었다. 마취과 간호사로 5년 일하시다가 국민건강보험공단으로 입사해서 일하시는 선생님과 이야기를 나누면서 간호사로써 병원에서 일하는 것과 국민건강보험공단에서 일하는 것의 장점, 단점과 차이에 대해 알게되어서 의미있고 나에게 도움이 되었다. 간호사가 국민건강보험공단, 연금보험공단 등 다양한 곳에서 일할 수 있다는 것을 알게되어서 의미있었다. 노인 장기요양보험에 대해 알게되어서 의미있었다.

2. 오늘 실습을 하는 동안 나의 생각과 느낌은 어떠했는가?

간호사 면허증을 가지고 일할 분야가 많다는 것을 느꼈다. 그래서 대학병원 간호사뿐만 아니라 간호사로써 일할 수 있는 다양한 분야에 대한 정보를 알아보고 미리 준비하는 것이 중요하다고 생각했다. 요양직은 노인장기요양보험서비스로 인해 출장이 많다는 것을 느꼈다. 의료보험을 위해 건강직, 요양직으로 나눠어져서 업무를 분담하여 체계적으로 업무를 시행한다고 느꼈다. 우리나라가 초고령화 사회가 되고 있고 군산시에 65세 이상 어르신들이 많이 살고 있어서 노인장기요양보험 대상자가 많고 장기요양급여를 받고 계시는 분들이 많다고 느꼈다. 장기요양등급판정위원회에서 6개월 이상 동안 혼자서 일상생활을 수행하기 어렵다고 인정하는 경우 심신상태 및 장기요양이 필요한 정도를 등급판정기준에 따라 장기요양 1~5등급 중 등급을 선정하여 그 등급에 따라 적절한 서비스를 제공해준다는 것을 알고나니 노인들에게 꼭 필요한 서비스라고 느꼈다.

3. 오늘 실습을 통해 새롭게 알게 된 것은?

오늘 실습을 통해 노인장기요양보험에 장기요양등급의 구분, 장기요양급여 종류에 대해 새롭게 알게 되었다. 그리고 치매전담형 장기요양기관에 대해 새롭게 알게 되었다. 장기요양 1등급은 심신의 기능상태 장애로 일상생활에서 전적으로 다른사람의 도움이 필요한 자로서 장기요양인정점수가 95점 이상인 자이다. 장기요양 2등급은 심신의기능상태 장애로 일상생활에서 상당부분 다른사람의 도움이 필요한 자로서 장기요양인정점수가 75점 이상 95점 미만인 자이다. 장기요양 3등급은 심신의 기능상태 장애로 일상생활에서 부분적으로 다른사람의 도움이 필요한 자로서 장기요양 인정점수가 60점 이상 75점 미만인 자이다. 장기요양 4등급은 심신의 기능상태장애로 일상생활에서 일정부분 다른사람의 도움이 필요한 자로서 장기요양인정 점수가 51점 이상 60점미만인 자이다. 장기요양 5등급은 치매환자로서 장기요양인정점수가 45점 이상 51점 미만인 자이다. 장기요양 인지지원등급은 치매환자로서 장기요양 인정 점수가 45점 미만인 자이다. 장기요양급여는 크게 재가급여, 시설급여, 특별현금급여로 구분되며 중복하여 이용할 수 없으나, 특별현금급여(가족요양비) 지급 대상자의 경우에는 기타재가급여(복지용구)는 추가로 이용할 수 있다. 치매전담형 장기요양기관은 치매 어르신이 안정감을 느낄 수 있는 시설환경을 제공하고 치매전문교육을 받은 인력을 배치하여 맞춤형서비스를 제공하는 것이다.

4. 오늘 실습을 통해 새롭게 배운 것들이 향후 간호실무에 어떻게 적용될 수 있겠는가?

65세 이상의 노인 또는 '치매·뇌혈관질환 등 노인성질병이 있는 65세 미만의 자'가 6개월 이상 동안 혼자서 일상생활을 수행하기 어려워 수급자로 판정받은 경우, 장기요양 기관으로부터 신체활동 또는 가사활동, 인지활동 지원 등의 장기요양급여를 받을 수 있도록 안내해드릴 것이다. 장기요양급여를 이용할 수 있도록 장기요양급여(서비스) 이용절차를 알려드릴 것이다. ① 필수서류수령(장기요양인정서와 개인별장기요양이용계획서, 복지용구 급여확인서), 장기요양기관 현황을 제공받고 수급자에게 맞는 서비스를 이용할 수 있도록 안내해 줄 것이다. ② 장기요양기관 선택 및 급여 계약 ③ 장기요양급여 이용에 대한 설명을 해드릴 것이다. 장기요양 인정 및 유효기간 및 신청에 대해 안내해드릴 것이다. 인지활동형 프로그램을 이용하는 인지활동형 방문요양에 대해 알려드려 인지기능이 저하된 치매수급자의 인지자극활동 및 남아있는 신체·인지 기능의 유지 향상을 위한 훈련을 제공받을 수 있도록 도울 것이다.

5. 오늘 실습에서 좋았던 점과 아쉬웠던 점은 무엇인가?

국민건강보험에 입사하는 방법과 간호사 면허가 있는 상태에서 지원할 수 있는 부분에 대해 알게 되어서 좋았다. 그리고 국민건강보험에서 일하는 건강직과 요양직의 업무 차이에 대해 알게 되어서 좋았다. 간호사로써 병원이 아니라 국민건강보험공단에서 일할 때의 장점에 대해 알게되어서 의미있고 좋았다. 노인장기요양보험에 장기요양등급의 구분, 장기요양급여 종류(재가급여 - 방문요양, 방문목욕, 방문간호, 주·야간보호, 단기보호, 인지활동형 방문요양, 기타 재가급여, 시설급여 - 노인요양시설, 노인요양공동생활가정, 특별현금급여 - 가족요양비)에 대해 자세히 알게되어서 좋았다. 장기요양급여(서비스)의 이용절차에 알게 되어서 좋았고 치매전담형 장기요양기관에 대해 새롭게 알게 되어서 좋았다. 아쉬운 점은 없다.

취업분야
간호전문직
경력
진로
보건소

1. 오늘 실습경험의 의미 있는 점은 무엇인가?

오늘은 예방접종실에서 하는 일인 다양한 예방접종 종류에 대해 알게 되어서 의미있었다. 무료예방접종과 유료예방접종들의 종류를 새롭게 알게 되어서 의미있었다. 만 12세 이하 아동에게 국가필수예방접종을 무료로 접종하고 있고 만 12세 여성 청소년에게 자궁경부암 예방접종을 6개월 간격으로 2회 접종을 지원해준다는 것을 알게 되어서 의미있었다. 또 다른 예방접종으로 어르신폐렴이 있다는 것을 알게 되었다. 1958년 이전 출생 어르신에게 무료 접종을 시행하고 있다는 것을 알게 되어서 의미있었다. 유료접종으로는 추가 B형간염(0.1, 6개월 간격), 장티푸스(만 5세이상, 1회 접종으로 3년간 유효), 신증후군출혈열(0-1-12개월 간격), A형간염(소아용, 성인용)이 있다는 것을 알게 되어서 의미있었다. 기타 예방접종 궁금증에 대해 알게 되어서 의미있었다.

2. 오늘 실습을 하는 동안 나의 생각과 느낌은 어떠했는가?

무료로 시행하는 국가 예방접종의 종류가 많다고 느꼈다. 영유아의 경우 결핵균에 노출되면 결핵 뇌수막염, 파종성 결핵 등 중증 결핵이 발생할 가능성이 높기 때문에 BCG 예방접종을 통해 예방하는 것이 중요하다고 생각했다. 13가지 폐렴구균 백신 프리베나 13으로 침습성 질환, 급성중이염, 폐렴을 예방할 수 있으므로 예방접종을 시행하는 것이 중요하다고 느꼈다. 또 영유아는 면역력이 약해 감염성에 취약하므로 출생 후 국가가 지정한 필수예방접종을 시행하는 것이 중요하다고 생각했다. 조혈모 세포 이식대기자가 2014년 이후 계속 증가하고 있는데 제대혈 기증 및 이식현황이 계속 줄어드는 것을 보면서 산모를 대상으로 제대혈, 제대혈 기증에 대해 안내해드리고 홍보하는 것이 필요하다고 느꼈다. 1958년도에 태어난 사람들이 보건소에서 폐렴구균 예방접종 대상자로 예방접종 하러 오시라는 문자를 보낸 것을 보고 많은 사람들이 방문하시는 것을 보며 문자를 통해 폐렴구균 예방접종을 안내하고 예방접종을 시행하는 것은 좋다고 느꼈다.

3. 오늘 실습을 통해 새롭게 알게 된 것은?

오늘 실습을 하면서 제대혈 기증에 대해 새롭게 알게 되었다. 제대혈이란 출산시 단 한번만 얻을 수 있는 탯줄과 태반에 존재하는 혈액이다. 기증된 제대혈은 조건이 일치하는 환자에게 이식하여 백혈병, 재생불량성 빈혈 등 난치성 혈액질환을 치료하는 데 사용된다. 제대혈을 보관하는 방법에는 가족제대혈과 기증제대혈이 있다. 기증제대혈은행은 조혈모세포 이식용 기증제대혈 보관·공급하는 곳으로 제대혈이식이 필요한 모든 국민에게 공급하고 보관비용은 국가에서 지원하기 때문에 비용이 없다. 가족제대혈은행은 본인 조혈모세포 이식용 가족제대혈 보관, 공급하는 곳으로 공급대상은 제대혈 이식이 필요한 본인 및 가족이다. 보관비용은 본인이 부담해야한다. 제대혈은 제대혈기증동의 및 채취동의를 한 산모를 대상으로 분만 시 의료진에 의해 채취된다. 산모의 탯줄 및 태반으로부터 채취하며, 신생아 및 산모에게 위험하지 않다는 것을 새롭게 알게 되었다. 폐렴구균은 소아에서 균혈증, 수막염, 폐렴 및 중이염의 주요 원인균이다. 3~5개월 소아에게는 수막염이 많이 발생하고 6~12개월 소아에게는 중이염이 많이 발생하고 13~18개월 소아에게는 폐렴이 많이 발생한다. 결핵(BCG) 예방접종 후 나타날 수 있는 이상반응으로 국소농양, 림프절염, 무통성궤양이나 켈로이드와 같은 국소 이상반응이 나타날 수 있다는 것을 알게되었다.

다양한 활용 #

4. 오늘 실습을 통해 새롭게 배운 것들이 향후 간호실무에 어떻게 적용될 수 있겠는가?

출산 시 제대혈 기증에 대해 설명해 줄 것이다. 아기성장 단계별 빈번한 폐렴구균 질환이 다르므로 3~5개월, 6~12개월, 13~18개월에 빈번한 폐렴구균 질환에 대해 안내해드릴 것이다. 프리베나 13 폐렴구균 백신을 접종하여 침습성질환, 급성중이염, 폐렴을 예방할 수 있도록 도울 것이다. 결핵(BCG) 예방접종 종류에 대해 설명해드려 피내접종 또는 경피접종 두 종류 중 한 종류를 선택하도록 할 것이다. 그리고 BCG (피내접종) 예방접종 후 정상 경과 과정에 대해 설명해드려 접종부위가 부풀어오르는 것에 대해 놀라지 않도록 할 것이다. 결핵(BCG) 예방접종 후 나타날 수 있는 이상반응인 국소농양, 림프절염, 접종부위 무통성 궤양이나 켈로이드와 같은 국소 이상반응 등에 대해 설명해드릴 것이다. 국소농양은 대부분 자연치유 되므로, 병변을 깨끗이 닦아주고, 연고, 항생제, 결핵약 투약, 수술은 필요하지 않다고 설명해드려 병변을 깨끗이 닦을 수 있도록 할 것이다.

5. 오늘 실습에서 좋았던 점과 아쉬웠던 점은 무엇인가?

예방접종실에서 실습하면서 국가에서 지원하는 필수예방접종, 무료 예방접종, 유료예방접종 등 다양한 예방접종의 종류와 기간, 주사방법에 대해 자세히 알게 되어서 좋았다. 아기성장 단계별 빈번한 폐렴구균 질환에 대해 알게 되어서 좋았다. 제대혈의 정의, 기증된 제대혈이 어디에 사용되는지, 기증제대혈은행과 가족제대혈은행의 차이, 제대혈 기증 및 이식현황, 제대혈 채취 방법 등 제대혈에 대한 내용을 자세히 알게 되어서 좋았다. 그리고 폐렴구균 예방접종을 접종하시러 오신 분들이 만 65세 이상 폐렴구균 예방접종 대상자인지 다시 한번 확인하고 예방접종 예진표 작성하는 것을 도와드리고 체온을 측정해드리고 기록한 것이 좋았다. 예방접종실을 방문하시는 분들을 응대하고 안내해드려서 좋았다. 폐렴구균예방접종밖에 보지 못해서 아쉬웠다.

CamScanner로 스캔하기

1. 오늘 실습경험의 의미 있는 점은 무엇인가?

오늘 방문건강관리실에서 실습을 하여 방문간호사 선생님과 함께 방문간호서비스를 제공하려고 나간 것이 의미있었다. 전립선암으로 항암치료를 마치고 집에 계신 대상자를 만나고 요즘은 어떤지? 어디 불편하신 곳은 없는지? 식사는 잘 하시는 지 확인하는 모습을 직접 관찰하고 혈압, 혈당을 측정하고 불편한 부위에 대한 간호와 교육을 제공하시는 모습을 직접 관찰해서 의미있었다. 두번째로 방문한 집에 계신 대상자에게 직접 혈압, 혈당을 측정 해봐서 의미있었다. 직접 대상자들을 만나고 이야기 나눠 본 것이 의미있었다.

방문간호

2. 오늘 실습을 하는 동안 나의 생각과 느낌은 어떠했는가?

방문간호서비스 대상자들이 집에 방문하고 이야기 나누는 것을 별로 안 좋아하시고 불편해하실 것 같다고 생각했었는데 오늘 직접 방문간호사선생님과 대상자의 집에 방문해보니 반갑게 맞이해주시고 엄청 협조적인 모습을 보면서 방문간호서비스의 필요성과 중요성에 대해 다시 한번 깨닫게 되었다. 건강관리는 잘 하고 계신지, 식사는 잘 하시는 지, 아프신 곳은 없는 지 주기적으로 확인하고 방문하였을 때 혈압, 혈당을 측정하여 대상자의 현상태를 확인하는 것이 중요한 일이라고 생각했다. 또 매일 방문하는 것이 아니기 때문에 저번 방문 이후에 어떻게 지내셨는지, 혹시 무슨 일 있으셨는지 물어보고 기록하는 일도 중요한 일이라고 느꼈다. 연락이 안 되시는 분들도 꽤 있으셔서 미리 연락해보고 방문해야겠다고 느꼈다.

방문 간호

3. 오늘 실습을 통해 새롭게 알게 된 것은?

방문간호는 방문간호지시서에 따라 간호(조무)사 또는 치과위생사가 수급자의 가정 등을 방문하여 간호 및 처치, 교육, 상담, 구강위생 등을 제공하는 것이라는 것을 새롭게 알게 되었다. 방문간호 이용 대상자는 간호 및 처치, 예방관리 등이 필요한 수급자, 방문요양 또는 방문목욕을 이용하는 1등급부터 5등급까지의 수급자 중 간호처치가 필요한 수급자는 월 1회에 한하여 월 한도액과 관계없이 예방관리 등을 위한 방문간호를 이용할 수 있다. 최초로 장기요양등급을 받은 1~5등급 치매수급자는 등급을 받고 60일 이내 최대 4회(월 2회)까지 방문간호서비스를 본인부담 없이 이용할 수 있다. 방문간호 급여비용에는 처치에 사용된 재료비나 검사료가 포함되어 있으므로 재료비에 대한 본인부담금은 없다. 방문간호서비스를 이용하기 위해서는 방문간호지시서를 발급받고 방문간호기관과 급여계약을 한 후 방문간호 급여를 이용하는 절차를 진행해야한다. 방문간호 급여는 동일한 날 의료기관에서 제공하는 가정간호와 함께 이용할 수 없다. 방문간호에 대한 전반적인 내용에 대해 새롭게 알게 되었다.

4. 오늘 실습을 통해 새롭게 배운 것들이 향후 간호실무에 어떻게 적용될 수 있겠는가?

방문간호를 이용할 수 있는 대상자인지 확인하고 방문간호서비스를 이용할 수 있는 대상자인 경우 대상자에게 방문간호 이용하기 위해 방문간호지시서를 발급받고 방문간호기관과 급여계약을 한 후 이용할 수 있다고 설명해 줄 것이다. 방문요양 또는 방문목욕을 이용하는 1등급부터 5등급까지의 수급자 중 간호처치가 필요한 수급자는 월 1회에 한하여 월 한도액과 관계없이 예방관리 등을 위한 방문간호를 이용할 수 있음을 안내해드릴 것이다. 방문간호급여비용에는 처치에 사용된 재료비나 검사료가 포함되어 있으므로 재료비에 대한 본인부담금은 없다고 설명해줄 것이다. 방문간호는 방문간호지시서에 따라 간호(조무)사 또는 치과위생사가 수급자의 가정 등을 방문하여 간호 및 처치, 교육, 상담, 구강위생 등을 제공하는 것이라고 설명해주고 많은 사람이 이용할 수 있도록 홍보할 것이다.

5. 오늘 실습에서 좋았던 점과 아쉬웠던 점은 무엇인가?

방문간호사선생님과 방문간호 대상자의 집에 방문하여 대상자를 만나 직접 만나 대화를 나누고 혈압, 혈당을 측정해봐서 좋았다. 암 환자를 직접 만나 암 환자의 고민 등 대화를 나누고 심적 간호를 제공해 본 것이 좋았다. 그리고 혼자 계신 대상자의 식생활, 생활습관, 약 복용 여부에 대해 물어보고 기록한 것을 직접 관찰한 것이 좋았다. 방문간호에 대한 전반적인 내용과 이용절차에 대해 알게 된 것이 좋았다. 요양원과 더 많은 대상자의 집을 방문하지 못해서 아쉬웠다.

CamScanner로 스캔하기

p15

1. 오늘 실습경험의 의미 있는 점은 무엇인가?

오늘 방문건강관리실에서 실습하면서 방문간호사 선생님들의 주 업무에 대해 알게된 점이 의미있었다. 그리고 방문건강관리의 목표에 대한 내용을 알게 된 것도 의미있었다. 방문건강관리실에서 시행하는 레드서클 캠페인에 대해서도 알게 되어서 의미있었다. 또 심근경색증 바로알기라는 사업을 시행하여 심근경색의 정의, 발생과정, 증상, 진단검사, 치료방법 등을 교육하는 내용을 알게된 것이 의미있었다. 그리고 진드기매개감염병의 예방수칙과 관리에 대해 자세히 알게 되어서 의미있었다.

예방사업

2. 오늘 실습을 하는 동안 나의 생각과 느낌은 어떠했는가?

방문건강관리실에서 방문간호 대상자의 집에 방문하여 혈압, 혈당을 측정하여 관리하는 일 말고도 심뇌혈관질환 예방 사업과 같은 다양한 사업을 하고 있다고 느꼈다. 방문건강대상자를 관리하기 위해 방문건강관리의 목표를 크게 건강형태 개선과 건강문제 관리로 나눠서 방문간호 대상자를 관리한다는 것을 알고나니 대상자의 상태에 따라 체계적으로 건강관리가 될 수 있도록 노력하고 있다고 생각했다. 심뇌혈관 질환을 예방하기 위해 레드서클 캠페인을 진행하는데 대상자에게 자신의 혈관 숫자를 알려주고 심뇌혈관질환의 예방수칙에 대해 알려주기 때문에 대상자들에게 많은 도움이 될 것 같다고 느꼈다.

3. 오늘 실습을 통해 새롭게 알게 된 것은?

방문건강관리의 목표에 대해 새롭게 알게 되었다. 방문건강관리의 목표는 2가지로 나눠진다. 첫번째는 건강형태 개선으로 방문간호 대상자가 본인의 건강상태를 인식하고 건강생활을 실천할 수 있도록 유도하며 건강지식을 향상시키는 것이다. 두번째는 건강문제 관리로 방문간호 대상자의 건강문제를 정기적으로 스크리닝을 하고 증상을 조절해주고 치료의 순응도를 향상시키는 것이다. 레드서클 캠페인이란 자기혈관 숫자 알려주고 담배는 반드시 끊도록 교육하고 술은 하루에 한두 잔 이하로 줄이도록 교육하는 것이다. 또 음식은 싱겁게 골고루 채소와 생선을 충분히 섭취하고 가능한 매일 30분이상 적절한 운동을 할 수 있도록 교육하는 것이다. 진드기매개감염병의 예방수칙은 옷을 제대로 입고 기피제 뿌리고 풀숲 피하고 야외활동 후 전신샤워를 하고 야외복은 분리해서 세탁을 하는 것이다.

간호전문직

4. 오늘 실습을 통해 새롭게 배운 것들이 향후 간호실무에 어떻게 적용될 수 있겠는가?

방문간호대상자들의 건강관리를 위해 방문건강관리의 목표에 따라 건강형태 개선과 건강문제를 관리하여 방문간호대상자들의 건강관리가 될 수 있도록 노력할 것이다. 심뇌혈관질환예방 사업을 진행하여 대상자들이 현재 본인의 혈관 상태를 알고 심뇌혈관질환의 예방수칙을 스스로 실천할 수 있도록 담배 끊기, 술 한두잔으로 줄이기, 하루 30분이상 적절한 운동하기 등 예방수칙을 쉽고 자세히 교육할 것이다. 그리고 진드기매개감염병의 예방수칙을 안내하여 대상자 스스로 진드기매개감염병의 예방수칙을 실천할 수 있도록 할 것이다. 또 진드기매개감염병의 증상에 대해 교육하여 빨리 치료할 수 있도록 할 것이다.

5. 오늘 실습에서 좋았던 점과 아쉬웠던 점은 무엇인가?

방문건강관리의 목표에 대해 자세히 알게 되어서 좋았다. 심뇌혈관질환을 예방하기 위해서 방문건강관리실에서 시행하는 레드서클 캠페인에 대해 새롭게 알게 되어서 좋았다. 또 심근경색증 바로알기라는 사업을 시행하여 지역주민들에게 심근경색의 정의, 발생과정, 증상, 진단검사, 치료방법 등 교육하는 내용에 대해 알게 되어서 좋았다. 방문건강관리실에서 방문간호뿐만 아니라 다양한 사업과 업무에 대해 알게 되어서 좋았다. 그리고 진드기매개감염병의 증상과 예방수칙에 대해 알게 되어서 좋았다. 방문간호를 가지 않아 대상자를 만나지 못해서 아쉬웠다.

CamScanner로 스캔하기

1. 오늘 실습경험의 의미 있는 점은 무엇인가?

충치와 잇몸병을 예방하기 위한 칫솔질에 대해 알게 되어서 의미있었다. 충치가 생기는 과정에 대해 자세히 알게 되어서 의미있었다. 치간칫솔법에 대해 알게 되고 불소를 사용해야하는 이유에 대해 알게 되어서 의미있었다. 단 음식과 담배가 왜 구강건강에 해로운지에 대해 알게 된 것이 의미있었다. 소아와 청소년은 6개월에 한번씩 불소도포를 받아 충치를 예방할 수 있고, 어르신이나 성인은 불소도포를 받아 치아 뿌리의 충치를 예방하고, 치아가 시린 증상을 완화할 수 있다는 것을 알게 된 것이 의미있었다.

2. 오늘 실습을 하는 동안 나의 생각과 느낌은 어떠했는가?

불소가 치아를 튼튼하게 만들어주고 충치로부터 치아를 보호해주기 때문에 꼭 불소가 들어가 있는 치약을 사용하여 양치질을 해야하고 주기적으로 보건소 또는 치과 병의원에 방문하여 불소도포를 받아야한다고 느꼈다. 특별한 증상이 없어도 치과에서 구강검진을 받고 치아 홈 메우기(실런트)로 첫번째, 두번째 큰 어금니의 충치를 예방하고 치석제거(스케일링)을 받아 잇몸병을 예방하는 것이 중요하다고 생각했다. 칫솔질을 할 때 치아의 바깥쪽면(입술·볼 부분), 안쪽 면(혓바닥 부분), 씹는 면과 혀를 모두 닦고 치아와 잇몸경계, 씹는 면, 치아 사이는 칫솔모가 닿지 않으므로 세균이 더 많으니까 더 꼼꼼하게 닦을 필요가 있다고 느꼈다.

3. 오늘 실습을 통해 새롭게 알게 된 것은?

유치도 구강검진을 받아야 하는 이유와 영유아 구강검진은 언제 받는지에 대해 새롭게 알게 되었다. 치실과 치간 칫솔의 사용방법에 대해 새롭게 알게 되었다. 유치는 빠지는 치아인데 구강검진을 받아야하는 이유는 유치가 건강한 어린이는 음식을 잘 씹어서 섭취하고 정확하게 발음하며 웃을 수 있다. 그러나 충치가 있어 유치가 기능을 제대로 하지 못할 경우, 아이의 성장과 발육은 물론 심리상태에도 영향을 미칠 수 있기 때문이다. 영유아 구강검진은 18~29개월(2세) 때, 42~53개월(4세) 때, 54~65개월(5세) 때, 66~71개월(6세) 때 문진, 시진, 구강보건 교육을 시행한다. 치간칫솔사용방법은 치간 칫솔의 심지가 치아나 잇몸에 닿지 않는 크기인 것으로 선택하여 치아 사이의 공간에 치간칫솔을 넣은 후 안쪽으로 5번 정도 움직여서 세균을 닦아준다. 혀 쪽에서 바깥쪽으로도 치간칫솔을 넣어 같은 방법으로 닦아준다.

4. 오늘 실습을 통해 새롭게 배운 것들이 향후 간호실무에 어떻게 적용될 수 있겠는가?

유치는 곧 빠질텐데 구강검진을 받아야하냐고? 묻는 대상자에게 영유아 구강검진을 받아야하는 이유에 대해 설명해드리고 영유아 구강검진을 받아야 할 시기에 받을 수 있도록 할 것이다. 치간칫솔사용방법과 치실의 사용방법을 교육하여 칫솔질을 한 후 치아 사이에 남아 있는 음식물을 제거할 수 있도록 할 것이다. 불소는 치아를 구성하는 수산화인회석 성분과 결합하여 치아를 튼튼하게 만들고, 충치로부터 치아를 보호해주는 것이기 때문에 불소가 들어있는 치약을 사용하여 양치질을 해야한다고 교육할 것이다. 잠을 자는 동안, 입속의 세균을 닦아주는 침의 분비가 줄어들고 세균의 활동이 활발해지기 때문에 잠자기 전 칫솔질로 입속의 세균 수를 최대한 줄여야한다고 설명할 것이다.

5. 오늘 실습에서 좋았던 점과 아쉬웠던 점은 무엇인가?

구강건강을 위한 생활수칙인 ① 하루 2번 이상 칫솔질 하기 ② 불소치약을 이용하기 ③ 단 음식을 줄이고, 담배를 피우지 않기 ④ 정기적으로 치과 방문하기에 대해 알게 되어서 좋았다. 유치도 구강검진을 받아야 하는 이유, 영유아 구강검진은 언제 받는지, 또 치실과 치간 칫솔 사용방법에 대해 몰랐던 내용을 알게 되어서 좋았다. 불소가 무엇인지, 양치질할 때 불소치약을 사용해야 하는 이유, 불소도포가 소아·청소년에게는 어떤 점이 좋고 성인과 어르신에게는 어떤 점이 좋은 지 알게 되어서 좋았다. 아쉬운 점은 없었다.

CamScanner로 스캔하기

1. 오늘 실습경험의 의미 있는 점은 무엇인가?

오늘 접수실에서 실습하면서 보건소에서 할 수 있는 일인 보건증 만들기, 결핵검사, A,B형 예방접종, 치매검사, 채용건강검진, 폐렴 예방접종, 코로나 양성률 조사, 인바디 검사, 운전면허적성 검사 등을 어디로 가서 어떻게 시행하는 지에 대해 알게 되어서 의미있었다. 그리고 암, 난임 희귀난치, 영유아 건강검진 지원 관련, 병·의원 관련 신고, 전염병, 방역 관련 보건사업, 금연 프로그램, 영양플러스 사업, 건강클리닉 운영, 아토피 지원, 산후도우미 신청, 유축기 대여, 산전검사, 엽산제, 철분제 지원 등 다양한 보건소 사업에 대해 알게되어서 의미있었다.

2. 오늘 실습을 하는 동안 나의 생각과 느낌은 어떠했는가?

보건소에 다양한 업무 때문에 대상자들이 많이 방문한다고 느꼈다. 또 보건소에서 하는 업무와 사업이 많다고 느꼈다. 암, 난임 희귀난치, 영유아건강검진 지원 사업, 아토피 지원 사업, 영양플러스 사업, 산후도우미 신청, 유축기 대여, 산전 검사, 엽산제, 철분제 지원 사업은 지원을 받을 수 있는 대상자에게 도움이 되며 좋은 사업이라고 생각했다. 보건소에서 사전연명의료의향서 신청이 가능한 지 몰랐었는데 사전연명의료의향서를 작성하려 왔다고 어디로 가야하냐고 물어봐서 알게되었으며 사전연명의료의향서 작성을 통해 연명의료 중단 결정을 하시는 분들이 꽤 많다고 느꼈으며 요즘 연명의료중단에 대한 관심이 많이 늘어났다고 생각했다.

3. 오늘 실습을 통해 새롭게 알게 된 것은?

연명의료에 관련된 내용들을 새롭게 알게 되었다. 사전연명의료의향서는 19세 이상 성인이 향후 자신이 임종과정에 있는 환자가 되었을 때를 대비하여 연명의료 중단 등 결정 및 호스피스에 관한 의사를 문서로 작성하는 것이다. 연명의료계획서는 말기 또는 임종과정에 있는 환자가 본인의 연명의료 유보 또는 중단에 관한 의사를 남겨놓는 것이다. 사전연명의료의향서는 본인이 직접 작성하는 것이고 연명의료계획서는 환자의 요청에 의해 담당의사가 작성하는 것이다. 중단할 수 있는 연명의료 시술에는 심폐소생술, 혈액투석, 항암제 투여, 인공호흡기 착용, 체외생명유지술, 수혈, 혈압상승제 투여가 있다. 단, 연명의료중단 결정 및 이행 시 통증 완화를 위한 의료 행위와 영양분 공급, 물 공급, 산소의 단순공급은 시행하지 않거나 중단되지 않는다. 연명의료는 임종과정에 있는 환자에게 의학적 시술로서 치료효과 없이 임종과정의 기간만을 연장하는 것으로 연명의료결정제도는 환자가 존엄하게 삶을 마무리 할 수 있도록 하는 것을 목적으로 하고 있다. 연명의료 유보는 임종과정에 있는 환자에게 연명의료를 처음부터 시행하지 않는 것이다.

4. 오늘 실습을 통해 새롭게 배운 것들이 향후 간호실무에 어떻게 적용될 수 있겠는가?

오늘 알게된 사전연명의향서, 연명의료계획서, 연명의료 유보, 연명의료 중단, 호스피스·완화 의료 등 연명의료결정제도의 주요 용어의 정의를 정확하게 설명해 줄 것이다. 또 사전연명의향서와 연명의료계획서의 차이를 정확하게 알고 차이점에 대해 안내할 것이다. 작성방법, 작성 시 주의할 점 등을 알려주고 언제든지 변경및 철회를 할 수 있다고 설명해드릴 것이다. 연명의료계획서에 대해 자세히 알고 임종간호를 제공하거나 고령환자의 사망을 빈번하게 겪을 때 보호자와 담당의사 사이에서 의견을 조율하고 중재하는 역할을 할 것이다. 후견인의 옹호자이자 사전연명의료의향서에 대해 설명할 수 있는 상담원으로 사전연명의료의향서에 대해 설명할 것이다. 환자와 보호자가 사전연명의료의향서 작성 시 최선의 결정을 내리도록 도울 것이다.

5. 오늘 실습에서 좋았던 점과 아쉬웠던 점은 무엇인가?

접수실에서 실습하면서 보건소에서 하는 업무, 사업을 어느 부서에서 하는 지 알게되고 보건소에 방문한 대상자의 민원에 직접 응대한 것이 좋았다. 그리고 사전연명의향서를 작성하려고 오신 대상자 덕분에 연명의료와 관련된 사전연명의향서, 연명의료계획서, 연명 의료 유보, 연명의료 중단, 중단 할 수 있는 연명의료 시술에 대한 내용을 찾아보고 연명의료에 관한 내용 중 몰랐던 부분에 대해 새롭게 알게 되어서 좋았다. 다양한 업무를 안내하지 못하고 거의 보건증, 예방접종에 관한 문의에 대해서만 안내해서 아쉬웠다.

CamScanner로 스캔하기

1. 오늘 실습경험의 의미 있는 점은 무엇인가?

지역사회인 보건소에 나와서 치매관련 사업에 대해 설명을 듣고 실제 캠페인에 직접 참여해서 홍보물과 소정의 선물을 전달하는 시간을 가져볼 수 있던 점이 의미있었다.

2. 오늘 실습을 하는 동안 나의 생각과 느낌은 어떠했는가?

생각보다 치매관련 사업이 세분화되어 있고 기준도 까다롭지 않은 점을 알게되며 신기하다는 생각이 들었다. 그리고 군산시 보건소의 규모가 좀 크다는 것을 느꼈다.

3. 오늘 실습을 통해 새롭게 알게 된 것은?

치매관련 대상자들은 1년에 한번 소득분위 산정을 통해 약값에 대해 지원금이 나온다는 것과 살면서 한번은 치매 진단 시 생필품 보조를 받을 수 있다는 것을 알게 되었다.

4. 오늘 실습을 통해 새롭게 배운 것들이 향후 간호실무에 어떻게 적용될 수 있겠는가?

주변에 치매와 관련이 있는 노인이나 지인이 있다면 보건소를 소개해주고 이를 통해 정부의 보조를 받을 수 있도록 안내해줄 수 있다.

5. 오늘 실습에서 좋았던 점과 아쉬웠던 점은 무엇인가?

보건소의 치매관련 사업에 대해 자세하게 설명을 들어보고 실제 캠페인에 참여해볼 점이 좋았다.

CamScanner로 스캔하기

1. 오늘 실습경험의 의미 있는 점은 무엇인가?

보건소 내에 있는 건강증진센터에서 시행하는 사업에 대해 알게되고 교수님과 컨퍼런스 시간을 통해 익산시 사례보고서의 부족했던 부분을 알고 다른 학생들의 간호과정도 확인했던 점이 의미있었다.

2. 오늘 실습을 하는 동안 나의 생각과 느낌은 어떠했는가?

보건소 내에는 여러분야로 나누어 사업을 운영하고 있고 각 팀에 여러 분야의 담당자를 배정하여 서로 상호보완 하며 업무를 처리할 수 있도록 되어 있다는 점을 알게 되며 효율적이다 라는 생각을 했다.

3. 오늘 실습을 통해 새롭게 알게 된 것은?

보건소에서 시행하는 보건소 모바일 헬스케어란 → 건강위험요인이 있는 사람에게 모바일앱을 통해 보건소전문가(의사, 코디네이터, 간호사, 영양사, 운동전문가)가 언제 어디서나 맞춤형 건강 상담을 제공하는 서비스 임을 알게되었다. 자가건강관리는 디바이스 연동 모니터링, 운동이력/소모칼로리 확인, 일일 실생활 미션 실천, 식사내용 입력/영양평가로 이루어져 있다.

협력

4. 오늘 실습을 통해 새롭게 배운 것들이 향후 간호실무에 어떻게 적용될 수 있겠는가?

건강정보 분석 및 상담은 매주 건강정보 콘텐츠 발송, 월 1회 건강실천 리포트 발송 및 분야별 전문가 상담, 보건소별 커뮤니티 운영및실시간 상담이 가능하다.

이 점을 알고 주변 건강관리가 잘 되지 않는 어르신이나 시민을 연동시켜 줄 수 있도록 적용할 수 있다.

5. 오늘 실습에서 좋았던 점과 아쉬웠던 점은 무엇인가?

보건소 사업 중 하나인 헬스케어 대상자분들을 직접 만나보며 지역사회에도 많은 사람이 건강에 관심이 있어 보건소에 방문하는 인이 상당하다는 것을 알게되어 좋았다.

CamScanner로 스캔하기

1. 오늘 실습경험의 의미 있는 점은 무엇인가?

건강보험 공단으로 실습을 나와서 공단 공무원의 업무를 조금 이나마 경험해 볼 수 있던 점이 의미있었다. 업무 수행을 하며 공단의 암검진에 대한 정확한 정보로 알게되어 의미있었다.

2. 오늘 실습을 하는 동안 나의 생각과 느낌은 어떠했는가?

항상 공기업에서 전화를 받는 입장에서 전화를 거는 입장이 되는 터니 긴장되었다. 혹여나 만 실수하지는 않을까, 전화 받는분이 화를 내지는 않을까, 등 여러상황에 대한 생각에 긴장하며 실습을 했다.

3. 오늘 실습을 통해 새롭게 알게 된 것은?

보통 6대 암검진의 위암, 간암, 유방암, 대장암, 폐암, 자궁경부은 각각의 2년, 6개월, 2년, 1년, 2년, 2년의 주기로 검사를 받게되고 건강보험료 하위 50% 건강보험 가입자, 의료급여수급권자는 본인부담금(10%)가 (할 [illegible]) 없다. 검진항목 중 2년 주기 항목은 전년도 미수검자로 공단 신청시 수검 이 가능하다.

4. 오늘 실습을 통해 새롭게 배운 것들이 향후 간호실무에 어떻게 적용될 수 있겠는가?

내가 나중에 나이가 더 들었을때, 그리고 나의 부모님에게 암검진의 주기와 검사방법을 정확히 안내 수행하거나 안내해줄 수 있을것 같다.

5. 오늘 실습에서 좋았던 점과 아쉬웠던 점은 무엇인가?

담당자분께서 전반적인 업무 설명할 때, 궁금한 부분을 해결해 주실때 너무나 친절하게 지도 해 주셔서 좋았다. 아쉬웠던점은 없었다.

CamScanner로 스캔하기

1. 오늘 실습경험의 의미 있는 점은 무엇인가?

실습은 4일차이지만 첫날처럼 단체 OT를 받았던 점이 의미있었다. 치매안심센터를 통해 치매의 증상과 원인을 알고 국가 정책도 알수 있었다. 그리고 진드기 매개 감염병, 건강보험공단의 간호사 역할을 알게되어 의미있었다.

2. 오늘 실습을 하는 동안 나의 생각과 느낌은 어떠했는가?

평소 잘 알지 못했던 치매와 진드기 매개 감염병(쯔쯔가무시, SFTS <중증열성혈소판감소증>)에 대해 설명을 듣고 예방법에 대해 교육을 받을 수 있어 감사하다는 생각을 했다.

3. 오늘 실습을 통해 새롭게 알게 된 것은?

SFTS는 참진드기에 물려 발생하는 질환으로 소화기증상(식욕저하, 구역, 구토, 설사, 복통)이 주증상이며 두통, 근육통, 신경증상(의식장애, 경련, 혼수), 림프절 종창, 호흡기증상(기침) 출혈증상(자반증, 하혈)을 일으킨다.

4. 오늘 실습을 통해 새롭게 배운 것들이 향후 간호실무에 어떻게 적용될 수 있겠는가?

진드기에 물리지 않도록 야외활동하는 사람에게 긴소매 긴바지, 다리를 완전히 덮는 신발을 착용하여 피부노출을 최소화하도록 교육합니다. 그리고 옷에 진드기 기피제를 도포하고 수행하도록 교육합니다.

교육자 역할

5. 오늘 실습에서 좋았던 점과 아쉬웠던 점은 무엇인가?

첫날에 받을 수 있는 OT를 받을 수 있어 실습에 대한 이해도를 높힐 수 있어 좋았다. 하지만 건강증진실에서 경험한게 없어 아쉽게 느껴졌다.

CamScanner로 스캔하기

1. 오늘 실습경험의 의미 있는 점은 무엇인가?

보건소 담당자분을 따라 방문간호를 직접 경험해본 점이 의미 있었다. 직접 혈당과 혈압도 측정해 드리고 간단한 말씀도 나누어 볼 수 있어 의미있었다.

2. 오늘 실습을 하는 동안 나의 생각과 느낌은 어떠했는가?

생각보다 우리나라의 많은 사람들이 보건소나 공단에 도움을 받고 있다는 점에 놀라고 또 그 만큼 연계 되어 있는 점이 신기하다고 생각이 들었다.

[illegible]

3. 오늘 실습을 통해 새롭게 알게 된 것은?

보통 질환을 진단 받고 5년은 보건소에서 연에 3~4회 정도 방문하여 관리하게 된다. 또한 요양등급에 따라 공단에서 보조할지 센터에서 보조할지 보건소에서 보조할지가 정해진다는 것을 알게되었다.

4. 오늘 실습을 통해 새롭게 배운 것들이 향후 간호실무에 어떻게 적용될 수 있겠는가?

대상자의 상태와 상황을 고려하여 지원받을 수 있는 지역사회프로그램을 연계해 줄 수 있다. 그리고 간호사가 어느방향으로 나아가서 지역사회에 도움이 될수 있을지 고려해봐야 할 것 같다.

5. 오늘 실습에서 좋았던 점과 아쉬웠던 점은 무엇인가?

직접 차량을 타고 개인건강방문 간호를 해볼 수 있어 좋았다. 하지만 더 많은 인원을 만나기 [illegible] 우리나라에 대상자가 많다는 점이 아쉬웠다.

1. 오늘 실습경험의 의미 있는 점은 무엇인가?

- 실습부서 (군산보건소 - 건강관리과) 관련 OT
- 치매인식 개선, 치매 파트너 교육
- 진드기 감염 예방 관리 (쯔쯔가무시, SFTS)
- 접수처에서 보건소 방문한 대상자들 위치 안내, 접수

2. 오늘 실습을 하는 동안 나의 생각과 느낌은 어떠했는가?

- 접수실에서 실습을 진행하였다. 보건소에는 보건증을 만들기 위해 오는 사람, 모자보건, 치매 안심 센터에 오는 사람이 많았으며 종종 구강검진센터, 금연클리닉 등 건강관리를 위해서 보건소를 찾는 사람이 많으며 보건소 내에는 굉장히 다양한 부서가 있다는 것을 알게되었다.
- 4월에는 진드기 감염이 빈번히 발생한다. 진드기는 작고 잘 눈에 띄지 않기 때문에 논사등을 할때 진드기가 몸에 들어올수 없도록 장갑, 긴양말등을 착용하며 피부가 노출되지 않도록 하는것이 중요하다는 것을 알게되었다.

3. 오늘 실습을 통해 새롭게 알게 된 것은?

실습을 하며 치매안심센터에 방문하는 사람들이 많음안다. 자신이 치매인지 의심되어 혼자 또는 친구들과 함께 오시거나 자식들과 함께 치매안심센터를 찾는 사람들이 많은것을 보고 치매에걸리는 것이 두렵다고 생각하는 사람이 많다는 생각을 하였고 치매에 대해 불안해하는 사람들이 많기에 치매에 관련된 교육을 진행하며 치매는 예방이되고 조기에 발견하면 진행을 늦출수 있다는것 등을 교육한다는것을 [illegible] 늘리면 좋겠다는 생각을 했다.

4. 오늘 실습을 통해 새롭게 배운 것들이 향후 간호실무에 어떻게 적용될 수 있겠는가?

- 쯔쯔가무시 등의 진드기에 관련된 올바른 내용을 사람에게 교육할 수 있다.
- 보건소, 병원등에 방문한 대상자들이 원하는 부서를 안내해 줄 수 있다.
- 치매와 관련하여 올바른 내용을 환자, 방문객에게 교육할 수 있다.

5. 오늘 실습에서 좋았던 점과 아쉬웠던 점은 무엇인가?

보건소에는 다양한 부서가 있으며 그 부서들의 위치를 알수있었고 사람들이 그부서를 찾는 빈도를 파악할 수 있어 좋았다.

CamScanner로 스캔하기

1. 오늘 실습경험의 의미 있는 점은 무엇인가?

◦ 불소 양치 용액 만드는것 관찰, 용액을 통에 담아 뚜껑을 닫아 시민들에게 제공 되는 불소 양치 용액을 만든 것이 의미 있었다.

◦ 만성질환(당뇨, 고혈압)에 대한 교육을 받는것이 의미있었다.

2. 오늘 실습을 하는 동안 나의 생각과 느낌은 어떠했는가?

• 실습을 진행하며 보건소에서 구강관련한 사업을 진행한다는 것을 알게되어 신기했으며 구강센터에서 여러가지 사업을 진행함에도 불구하고 사람들이 오지 않아 않은 사람들이 사업을 모르고 있을 가능성이 있다는 생각이 들었고 홍보가 필요하다는 생각이 들었다.

• 불소용액을 만들면서 불소용액의 효과와 사용방법 에 대해 처음보아 1분동안 가글해야 하는것 용액 가글 후 30분 정도는 입을 헹구거나 음식물을 섭취하지 않아야 하는것 등을 알수있어 좋았다.

시민참여

전문적? 보건교육

3. 오늘 실습을 통해 새롭게 알게 된 것은?

◦ 군산보건소 구강보건 센터에서는 노인의치보철 사업, 학교구강보건사업, 노인 불소도포, 스케일링, 사회복지 시설 구강건강 관리 사업, 불소용액 양치 사업, 미취학 어린이 구강건강검진 사업등을 진행한다.

◦ 충치 예방방법에는 불소도포(불소를 치아에 발라주어 산에 잘 견디게 함), 치아 홈메우기(어금니의 충치가 생길만한 홈을 치과재료로 메꾸는방법으로 90% 충치 예방의 효과가 있다.), 올바른 칫솔질법을 교육하여 평생 습관화 하는 방법이 있다.

◦ 잇몸병을 예방하기 위하여 1년에 2회 정도 정기적으로 치아와 잇몸사이에 부착된 치석을 제거하고 치아표면을 매끈하게 닦아준다. 적절한 구강위생용품을 선정하고 사용지도를 한다.

전문적 보건교육

4. 오늘 실습을 통해 새롭게 배운 것들이 향후 간호실무에 어떻게 적용될 수 있겠는가?

실습을 통하여 구강보건센터에서 진행하는 다양한 사업들을 관찰하고 배우게 되었다. 이러한점들은 임상에서 구강간호를 할때, 대상자에게 구강에 대한 교육을 할때 정확한 내용, 정보를 전달할 수 있는것으로 기대된다.

구강병은 널리퍼져있고, 만성적이고, 누진적이고 예방이 확실히 가능하다는 특성을 알고 대상자들에게 일단 발생된 구강병은 조기에 치료할수록 유리함을 교육할 수있다.

진로선택

진로선택

5. 오늘 실습에서 좋았던 점과 아쉬웠던 점은 무엇인가?

• 불소양치용액을 직접만들어 볼수있어 좋았다. 구강보건센터에서 어떤일을하는거 알고, 다양한 사업들을 진행하는 것들을 알게된 점이 좋았다.

◦ 시민분들이 많이 방문하지 않아 구강보건센터에서 진행하는 사업이 어떤식으로 적용 되는지 관찰할수 없었던 점이 아쉬웠다.

시민참여

CamScanner로 스캔하기

[illegible] 의미 있는 점은 무엇인가?

◦ 건강증진실에서 대상자 혈압측정, 간단한 혈액검사 (BST, 콜레스테롤 등), 인바디 검사를 바탕으로 의사선생님과 상담하는 과정 보조

◦ 구강보건센터 보조 (어린이집에서 구강교육과 양치방법 교육, 불소도포, 충치검진하는 과정 보조를 함) ◦ 지역사회 건강조사 우편보내야할 스티커 작업, [illegible] 안내문이 동봉 [illegible]

2. 오늘 실습을 하는 동안 나의 생각과 느낌은 어떠했는가?

◦ 선생님께서 치아교육을 하실때 치아 단면을 보여주며 사과 단면과 비교하시는 것을 관찰하였다. 평소에 실습을 하며 대상자가 이해하기 쉬운 언어를 사용해야 한다고 배우긴 했지만 평소 적용을 잘 못했는데 이런 예시를 들어 설명해 주어야 겠다는 생각을 했다.

#전문직 (교육)

◦ 건강증진실에서 모바일헬스케어라는 사업을 진행하고 있는데 이 사업은 스마트기기를 가지고 보건소에 직접방문하지 않아도 건강서비스를 받을 수 있다. 평소 이러한 서비스를 보건소에서 진행하는 것을 모르고 있었는데 실습을 통해 '모바일 헬스케어'라는 사업이 너무 좋은것 같다는 생각이 들어 선생님께 저 사는 지역에도 이 사업을 진행하는지 물어보았는데 생각보다 많은 지역에서 진행중인 사업이라 많이 놀랐다.

3. 오늘 실습을 통해 새롭게 알게 된 것은?

실습을 진행하며 건강증진실에서 여러가지 사업을 한다는 것을 알게되었다. 현재 공식적으로 시행을 시작한 사업은 '모바일 헬스케어'라는 사업이다. 이 사업의 추진배경은 만성질환 예방을 통하여 경제적 손실을 최소화하고 건강관리의 효율성을 제공하고 건강관리를 기기를 통하여 제공하여 직접방문의 불편함을 줄인다. 이 사업을 진행하기 위해서 코디네이터, 의사, 간호사, 영양사, 운동전문가의 5인이 필요하며 건강증진실에서 간호사는 수집된 건강정보 및 서비스 이용 확인을 통하여 건강관리 상담 및 관리의 목표를 설정하고 검사결과 정상수치 이상치를 살펴 모니터링하여 의사에게 연계해주는 역할을 한다는 것을 새롭게 알게 되었다.

#보건사업 이해.

#팀활동

4. 오늘 실습을 통해 새롭게 배운 것들이 향후 간호실무에 어떻게 적용될 수 있겠는가?

• 혈압측정, 간단한 혈액검사, 인바디검사를 통해 전반적인 대상자의 건강상태를 파악한 뒤 대상자에게 필요한 간호를 제공할 수 있다. 또한 새롭게 시작하는 사업에 대해 알고 적합한 대상자에게 권유하여 참여를 유도할 수 있다.

#의뢰자 (crossed out) 자기

◦ 아이들에게 무언가를 교육해야 할때 예시를 드는등 알아듣기 쉬운 언어를 사용할 수 있으며 양치방법 교육, 불소도포, 불소용액 사용법 등을 정확히 교육할수 있는것으로 기대된다.

5. 오늘 실습에서 좋았던 점과 아쉬웠던 점은 무엇인가?

#전문직 (교육)

여러 대상자를 만나고 대상자의 연령대에 맞춰 교육하는 방법을 알게된점, 불소도포 후 관찰, 불소는 먹어도 되며 최소 2시간은 유지해야 하는점을 알게된점, 건강증진실에서 여러가지 사업을 진행하는것을 알게되어 좋았다.

1. 오늘 실습경험의 의미 있는 점은 무엇인가?

건강보험 공단에서 실습을 진행하였으며 국민 건강검진 중 자궁경부 암 검진을 아직 받지 않은 대상자들에게 유선으로 자궁경부암 검진 대상자 임을 설명하고 올해안에 건강검진을 받도록 독려하는 것을 실습함.

임신중인경우, 자궁적출술을 받았거나 치료중인경우, 성경험이 없는경우에는 검진 제외할수 있으며
자궁경부암 검진은 2년단위로 진행되기에 전년도 미수검자들도 공단에 신청하여 수검할수 있다는것을 알게된점이 의미있었다.

2. 오늘 실습을 하는 동안 나의 생각과 느낌은 어떠했는가?

실습을 진행하며 건강검진 중 자궁경부암검진을 아직 받지 않은 대상자들에게 전화를하여 자궁경부암 검진을 독려하는 활동을했는데 처음에는 내가 잘 모르는 부분이 많기에 긴장도 많이 했다. 독려를하면서 자궁경부암에 대하여 모르시는 분도 있고 자신이 이번년도에 건강검진을 받는 사람인지 모르는 분들도 있기 검진을 받는분들이 적다는 사실을 알게되었고 임신중인경우 검사연기가 가능하기는 하지만 검진을 미룰수있다, 자궁적출술을 한경우 검진제외처리 후 하면 된다 등 올바르고 정확한 정보를 전달하기위해 노력했다. 하지만 전화로 비대면으로 해야하다보니 중간중간 당황하는 부분이 있어서 암검진에대해 더 많은 지식을 쌓아야 겠다는 생각을 하게 되었다.

효과적 의사소통
전문직
가-2

3. 오늘 실습을 통해 새롭게 알게 된 것은?

실습을 진행하며 건강보험공단에는 크게 장기요양팀과 보험급여 팀으로 나뉘어져 장기요양팀에는 등급판정, 인정신청, 의사소견서, 이용지원 등으로 세부적으로 나뉘어져 보험급여 팀은 건강검진, 대사증후군, 만성질환, 혈압계·혈당기 대여, 건강검진 (암, 일반, 영유아), 상해요인 · 기타징수금 등 세부적으로 나뉜다.
6가지 암 (대장암, 자궁경부암, 유방암, 위암, 간암, 폐암)는 국가 건강검진으로 검사받을 수 있으며 대장암과 자궁경부암은 본인부담금이 없다. 자궁경부암은 2년마다 자궁경부 세포를 검사하는 것으로 자궁적출술을 받았거나, 성경험이 없는경우 검진의사와 상의하여 진행을한다. 자궁경부암검사 최소 48시간 전부터는 질내에 어떠한 물질도 넣지않아야 하며 성관계, 탐폰, 질세척, 질내 약물, 윤활제 사용, 질내피임약 사용금지이다.

#

4. 오늘 실습을 통해 새롭게 배운 것들이 향후 간호실무에 어떻게 적용될 수 있겠는가?

오늘 실습을 통하여 자궁경부암 검진에 대한 내용을 알수 있었으며 건강보험공단에서 어떠한 업무를 진행하는지 알게되었다. 이를 토대로 암검강검진에는 6가지가 있으며 각각 검사별 어떤 주의사항이 있는지, 검진시기는 언제 인지, 어떤검사인지 안내할 수 있다.

5. 오늘 실습에서 좋았던 점과 아쉬웠던 점은 무엇인가?

여러 대상자에게 전화를 하여 자궁경부암 검진 대상자라는 것을 알려주고 검사를 독려하여 많은 사람들이 자궁경부암을 미리 예방할수 있도록 한것 같아 좋았다.
하지만 오늘 첫실습이었기에 초반에는 정확한 설명을 못해드린것 같아 아쉬웠다.

효과적 의사소통
느낌
설명 X

CamScanner로 스캔하기

1. 오늘 실습경험의 의미 있는 점은 무엇인가?

P17

① 지역사회 간호과정 사례보고서, CR보고서 발표에 대한 컨퍼런스를 진행하였는데 같이 실습하는 조원들의 발표를 들으며 군산시에서 발생하고 있는 문제점들에 대해 알수 있었으며 정확한 수치를 통한 객관적인 정보를 얻을수 있었다. 또한 CR보고서 발표를 통하여 군산시 보건소내의 각각 부서에서 어떠한 일을 하는지 자세히 알수 있었던것은 알수있기 의미있었다.

보험공단에 입사하려면 어떤것들을 준비해야 하는지 알수있었으며 간호사의 다양한 진로에 대해 이야기해볼수 있었던점에 의미있었다.

지역사회 문제 파악

2. 오늘 실습을 하는 동안 나의 생각과 느낌은 어떠했는가?

실습을 진행하고 지도해주시는 선생님과 대화를 하며 간호사면허를 가지고 할수있는 일이 많다는것을 느꼈다. 지금은 다들 임상으로 취업을 한다고하니 나도 그냥 그렇게 해야할것 같아 다른분야를 알아보지 않았는데 임상은 정말 많이 힘들기 때문에 나중에 내가 어떤것을 할지 생각해보고 미리미리 준비해야 겠다고 생각하는 계기가 되었다.

간호전문직 진로선택

② •컨퍼런스를 진행하여 군산시의 문제점들을 알수있는 기회였는데 내가 평소에 관심이 없어 생각하지 못하던 것들이 있었으며 각부서에서는 이문제를 해결하기위해 여러가지 계획을 세우고 프로그램을 진행하며 군산시 건강이 발전하기위하여 노력하고 있구나 하는 생각을 하게되었다.

지역문제 파악/사정

3. 오늘 실습을 통해 새롭게 알게 된 것은?

3-1

•간암검진은 40세이상 간암발생 고위험군이 대상이며, 초음파(간), 혈청알파태아단백검사로 진행된다. 간암발생 고위험군의 기준은 ① 간경변증 ② B형간염 바이러스 항원 양성 ③ C형간염바이러스 항체 양성 ④ B형 또는 C형간염바이러스에 의한 만성간질환 환자 이다. 간암은 6개월 주기로 검진을 실시한다.

• 폐암검진은 54세~74세 폐암발생고위험군을대상으로 검사하며 저선량흉부CT검사, 검진후 사후상담, 금연상담을실시한다. 폐암의 고위험군은 국가건강검진 대상자중 해당년도 전 2년 내에 국가건강검진 시 작성하는 문진표로 현재 흡연 중이며, 흡연력이 하루에 한갑기준 30년이상으로 확인되는자, 건강보험 금연치료 참여자.(해당년도 전 2년 내 금연치료 사업 참여위해 작성하는 문진표로 현재흡연중이며 흡연력이 하루 한갑기준 30년이상으로 확인되는자) 이다. 국가폐암 검진을 받는 사람은 금연을 하더라도 금연 15년 이내, 74세 이하라면 검진대상이 되므로 검사를 받아야한다. 검진실시기간은 해당년도의 12월31일까지이며 연말은 수검자의 40%가 저중되어 예약이 불가능하니 조기검진을실시하는것이 좋다.

4. 오늘 실습을 통해 새롭게 배운 것들이 향후 간호실무에 어떻게 적용될 수 있겠는가?

오늘 배운것들은 국가검진이 대상인 사람들에게 대장암은 50세 이상대상자, 1년주기로 분변잠혈검사로 진행되며 자궁경부암은 2년주기로 자궁경부세포검사, 20세이상 여성 대상으로 실시되며, 유방암은 40세 이상여성을 대상으로 유방촬영을 검사를 진행하며 2년주기 (출생연도 짝·홀수로 기준으로 적용한다.) 위암검진의경우 40세 이상이 대상자 이며 위내시경검사로 실시한다. 위내시경 검사가 어려운경우 위장조영 검사를 실시하고 필요시 조직검사를 실시한다(위암 의심자의 경우). 간암검진은 40세이상 간암발생 고위험군을 대상으로 간초음파와 혈청태아 단백검사를 실시한다. 폐암검진은 2년주기 이며 54세~74세 고위험군을 대상으로 실시하며 CT로 검사를 진행한다. 위의 국가6대 암검진의 대상, 주기, 검사방법을 알고 국가암검진을 필요로하는 대상자 에게 정확한 정보를 전달하고 검사를 독려할수 있을것이다.

5. 오늘 실습에서 좋았던 점과 아쉬웠던 점은 무엇인가?

실습을 진행하면서 간호사면허를 가지고 있으면 갈수있는 직업이 간호사이외에도 건강보험공단, 연금공단 등 다양한 국가기관에 취업할 수 있으며 전문간호사로 산업간호사등이 있다는것을 다시 생각해 보아 나중에 내가 무엇을 하고싶은지 생각해볼수있었던 시간이라서 좋았다.

국가암검진에는 어떤 종류가 있으며 각각의 대상자는 누구인지 검사주기와 각 암에 따른 고위험자가 누군지 알수있어 좋았다.

진로 선택

1. 오늘 실습경험의 의미 있는 점은 무엇인가?

보건소에서 접종하는 예방접종은 무료접종과 유료접종으로 나뉘어진다. 무료접종의 대상은 국가필수예방접종은 만 12세 미만아동 이며 폐렴구균, 뇌수막염은 생후 59개월이내, A형간염은 2012년이후 출생자가 대상이다. 자궁경부암은 만 12세 청소년을 대상으로 6개월 간격으로 2회 접종을 실시한다. 가다실4 와 서바릭스라는 약이 있지만 보통은 가다실 4를 많이 맞는다. 폐렴구균은 만 65세 이전의 만성질환자, 면역저하자등 위험군, 65세 이상일경우 면역저하자가 권장대상이다.
유료접종에는 추가 B형간염, 장티푸스, A형간염, 신증후군 출혈열이 있다는것을 이를 통하여 알게된점이 오늘실습경험중 의미있었다. A형간염, B형간염 동시접종 가능, 20의경우 생백신이 아니라면 4개까지접종이 가능하나 보통 2개만 접종한다.

2. 오늘 실습을 하는 동안 나의 생각과 느낌은 어떠했는가?

• 예방접종을 위해 보건소를 찾는 군산시민들이 있었는데 보건소에서 진행되고있는 예방접종의 수가 적어 중반정도의 사람들은 예방접종을 하러 왔다고 돌아가는것을 보며 보건소내의 예방접종의 개수를 늘릴수있다면 늘리면 좋을것 같다는 생각을 하였으며 착오가 생기지 않도록 이런것을 실행되고있는지 문자등으로 알려주면 좋을것같다는 생각이 들었다.

사업[illegible] 개선 필요

• 조혈모세포 이식 대기자는 계속해서 증가하고있지만 제대혈 기증및 이식현황은 점점 감소하고있다 아무래도 사전에 협약병원 여부를 확인하거나 국가 지정 기증제대혈은행에서 미리 알아봐야 하기 때문인것으로 생각이되고 제대혈 기증을 많이하도록 유도하려면 교육을 진행하거나 임신중에 이사실을 알리고 분만전에도 알리는등 적극적인 자세를 보여야할 것으로 생각된다.

3. 오늘 실습을 통해 새롭게 알게 된 것은?

실습을 통해 여러예방접종에 대하여 알게 되었다. 예방접종외에 제대혈 기증이라는것에대해 알수있었다.
제대혈이란 출산시에 단한번만 얻을수 있는, 탯줄과 태반에 존재하는 하는 혈액을 말한다. 제대혈을 보관하는 방법에는 '가족제대혈'과 '기증제대혈'이 있다. 가족제대혈이란 가족제대혈 은행에 비용을 지불하고 제대혈을 보관하는 것으로 향후 제대혈이식이 필요한경우 보관해둔 본인 제대혈을 이식할수있다.
'기증 제대혈'이란 기증제대혈은행에 제대혈을 기증하는것으로 제대혈이식이 필요한 국민누구나 사용할 수있다. 제대혈속에는 혈액을 만드는 줄기세포인 조혈모세포가 들어있다. 조직이 일치하는 환자에게 이식하여 백혈병, 재생불량성빈혈, 난치성 혈액질환을 치료하는데 사용된다. 제대혈은 분만시에 의료진을 통하여 채취되며 산모의 탯줄 및 태반에서 채취하기에 신생아와 산모에게 위험하지 않다. 채취된 제대혈은 36시간 내에 기증제대혈은행으로 수거되며 검사진행후 이식에 적합한 제대혈은 보관되나 부적합한 제대혈은 폐기되거나 연구용으로 사용된다.

4. 오늘 실습을 통해 새롭게 배운 것들이 향후 간호실무에 어떻게 적용될 수 있겠는가?

• B형간염 주산기감염은 임신 29주 ~ 생후 1주에 B형간염 산모의 체액이 신생아에게 노출되어 B형간염 바이러스가 엄마로부터 아이에게 전파되는 것을 의미한다. 주산기감염은 신생아 10명중 9명이 B형간염 만성보유자가 되어 성인에도위험할수 있기 때문에 위험하다는 것을 교육하고 B형간염 주산기감염을 예방하기 위해서 출생 12시간 이내에 B형간염 면역글로불린, 1차 백신을 같이투여하고 일정에맞춰 2, 3차를 완료하면 97% 예방이 가능하기에 예방접종이 필요함을 설명하고 예방접종을 진행할수있다.
또한 산전이나 분만후 7일 이내에 검사비를 지원하며 지원을 받을수있음을 알리고 적극적으로 예방할수있도록 교육할수 있을것으로 생각된다.

5. 오늘 실습에서 좋았던 점과 아쉬웠던 점은 무엇인가?

실습을통하여 보건소에서 실시하는 무료접종과 유료접종에 대하여 알게 되어 좋았으며 각 주사별 특징과 주의사항등 자세한내용을 알수있었다. 또한 제대혈 기증이 무엇인지, 어떻게 하는지, 어디에 도움이 되는지 알게되어 좋았으며 ~~약물~~ B형간염주산기감염이 무엇이며 어떻게 대처해야 하는지, 무엇을 지원받을수 있도록 도와주어야하는지 알게되어 좋았다.

CamScanner로 스캔하기

1. 오늘 실습경험의 의미 있는 점은 무엇인가?

ㅇ방문건강사업이 무엇인지, 방문간호의 대상은 누구이며 방문건강관리의 목표가 무엇인지 알수 있었던 점이 의미있었다.
ㅇ방문건강 간호사 선생님과 함께 가정방문 간호가 필요한 대상자들을 직접 만나러 집을 찾아간후 혈압과 혈당을 측정한후 지난번 결과와 비교하여 조절이 잘되고 있는지 아니면 조절이 잘안되고있어 다른 방식의 조절이 필요한지 등을 파악한후 대상자들에게 설명한 후 어떻게하면 건강관리를 더 효과적으로 할수있는지에대해 교육하는등의 실습을하고 관찰하고 교육한것이 오늘 실습경험에서 의미가 있었다.

2. 오늘 실습을 하는 동안 나의 생각과 느낌은 어떠했는가?

ㅇ방문간호 서비스 대상자들을 직접만나고 혈압과 혈당을 측정해드리면서 대화를 나눠보았는데, 일단 집앞에 방문하여 초인종을 눌렀을때 한번에 나오시는 분이 없어 청력이 많이 안좋아졌을수있다는 생각을 하였으며 방문을 했을때 파킨슨병을 앓고있어 거동이 어렵고 혈당조절이 잘 안되시는 분은 운동을 통해 혈당을 낮추는것이 어렵기때문에 음식조절과 약을통해서 조절이 가능하며 빵, 떡, 단것등의 음식은 갑작스럽게 혈당을 상승시킬수 있음을 설명드리고 하루의 목표일아라 [illegible]에 혈당을 측정하고 기록해두시라는 설명을 해드리는것을 보며 간호사는 교육자의 역할이 중요하다는 생각이 들었다.
ㅇ 약을먹은후 건강이 더 안좋아졌다며 양약을 다 끊고 한약을 드신다고 하시는 분이 계셨는데 이분은 혈압, 혈당모두 괜찮은수치여서 괜찮겠다고 생각을 하고 있었는데 선생님께서 저런분은 약을 안드시고 계시니까 더 신경쓰고 더 많이 방문해야 겠다고 하셔서 우리는 처방을 잘이행하는지 개인의 건강, 위생이 잘지켜지고 있는지 봐야한다는것을 다시한번 생각하게 되었다.

3. 오늘 실습을 통해 새롭게 알게 된 것은?

ㅇ방문건강관리 사업이란 보건소에 소속된 방문간호사가 각 가정을 방문하여 가족과 건강문제를 가진 구성원을 발견하여 질병 예방 및 관리, 건강증진을 위하여 건강 서비스를 제공하는것임을 알게되었다.
ㅇ 방문간호의 대상은 기초수급자, 독거노인, 차상위계층, 장애인, 다문화가족 등 의뢰한 건강문제가 있는 대상자, 재가암 환자관리, 재가 장애인, 집단서비스 (경로당 등)이 대상자로 방문간호를 실시하게 됨을 알게되었다.
ㅇ방문건강관리의 목표는 건강행태 개선과 건강문제관리이다. 건강행태개선에는 건강상태인지, 건강생활 실천 유도, 건강지식 향상 건강문제 관리에는 건강문제 정기적 스크리닝, 증상조절, 치료순응 향상이 포함된다.
ㅇ방문간호는 의료기관에서 방문간호 지시서를 발급받은후 장기요양기관 현황 또는 노인장기요양 보험 홈페이지를 통해 방문간호기관을 확인한 한후 계약을 한다. 이후 방문간호서비스를 이용한 후 이용중 궁금한사항은 공단담당자에게 문의한다.
방문간호에서의 발급비용은 수급자가 의료기관을 방문하는경우 (의료기관, 보건기관), 의사가 가정을방문하는경우(의료기관, 보건기관)다르다는 것을 새롭게 알게되었다.

4. 오늘 실습을 통해 새롭게 배운 것들이 향후 간호실무에 어떻게 적용될 수 있겠는가?

ㅇ방문요양또는 방문목욕을 이용하는 1등급부터 5등급까지의 수급자중 간호처치가 필요한수급자들에게, 예방관리 등을위해 방문간호를 이용할수 있음을 알려줄 수있다. 최초로 장기요양등급을받는 1~5등급 치매수급자는 등급을 받고 60일 이내에 최대 4회까지 방문간호서비스를 본인부담없이 이용할수 있으며 이때 방문간호는 치매교육을 이수한 간호사 또는 간호조무사가 건강행동을 ~~알려 대상자가~~ 방문간호 서비스를 받을수있도록 도와줄수 있는것으로 기대된다.
ㅇ방문간호서비스를 제공할때는 대상자의 상황을 고려하여 방문시간을 결정해야 하며 대상자에게 건강관리에 대하여 설명해 주어야할 경우 올바른 정보를 전달하도록 노력하고 대상자가 알아듣기 쉬운단어를 사용하여 대상자가 이해했는지 확인할수있도록 신경을 쓰는 등의 노력을 해서 이런것들을 간호실무에 적용할수 있을것 같다.

5. 오늘 실습에서 좋았던 점과 아쉬웠던 점은 무엇인가?

실습을 진행하면서 보건소내에서 인원들만 보았기 때문에 이번에 처음 실질적인 현지에 대해 관찰할수 있어 좋았으며 여러 집단의 대상자를 관찰하면서 각각 대상자별로 필요한 건강관리의 특성이 다다른것을 보고 건강관리에 대한 여러 피드백들을 얻을수있어 좋았다.
원래 오는 요일에 진행되는 경로당 방문간호에 대해서 관찰할수 없어 아쉬웠지만 내일 갈수있다는 생각에 아쉬운마음이 조금은 사라졌다.

#전문적

#건강 형평성?

#전문적

#맞춤형 건강관리

2군

1. 오늘 실습경험의 의미 있는 점은 무엇인가? P(1)

• 뇌졸중이 무엇이며 뇌졸중의 증상 발생시 바람직한 응급조치와 바람직하지 못한 응급조치에 대해 비교하여 알수 있게된 점이 의미 있었다. (평평한 바닥에 눕히기, 호흡을 방해하는 토사물이나 틀니 등 제거하기, 베개, 수건 등을 사용하여 기도확보하기, 넥타이 벨트 등을 풀어 호흡과 혈액순환을 원활하게 하기)

• 지역사회 조사를 위하여 여러가지 예방내용(팜플렛)을 포함하여 지역사회조사에대한 내용을 동봉하여 미리 알린후 지역사회 조사를 진행한다는 점. 요즘은 농경기라 쯔쯔가무시 등의 진드기 질환에 대하여 알게된점이 의미있었다.

2. 오늘 실습을 하는 동안 나의 생각과 느낌은 어떠했는가?

• 방문간호를 나가기전 대기를 하며 실습생 대기실안에 있는 여러가지 자료들을 읽어보며 다른 이유도 있을수 있지만 단순히 노인 이기 때문에 발생하는 질환, 발생위험이 높아지는 질환들이 많다는 생각을 했다. 하지만 노인분들은 정보와 가까이 하기 힘들고 새로 받아들일수 있는 내용이 많지 않기 때문에 방문간호를 통하여 지금가지고 있는 증상, 건강(혈압, 혈당)은 잘 조절이 되는지 확인을하고 잘조절이 되지 않는다면 건강관리 내용을 교육하고 더 자주 방문하여 건강상태를 사정하여 건강증진이 되도록. 조절이잘 되고있는거를 파악해야 한다는 생각도 한다.

• 방문건강 간호사는 건강만을 두고 여러 사람을 관찰, 관리해야하는 직업으로 스케줄 잡기, 여러 대상자 특성 기억하기 이사가면 정보 다시 습득 등 정말 다양한 부분을 신경써야 한다는것을 알게되어 새롭다는 느낌을 받았다.

3. 오늘 실습을 통해 새롭게 알게 된 것은?

「지역보건법」 제 4조 (지역사회 건강실태조사)에 의거하여 지역사회건강 실태조사 단면 안내문을 봉고 발송하기 위한 작업을 하면서 안내문에 들어가는 내용중에 진드기를 예방하기 위해서는 옷을 제대로 착용해야하는것. 모자, 목수건, 긴팔 긴바지, 장갑, 목이긴양말, 등산화를 착용하여 피부가 노출되지 않도록 해야한다. 기피제를 뿌리면 완전히 진드기가 오지 않는것은 아니지만 어느정도 예방할수있고 풀숲도 피해야 한다. 풀숲에 들어갔다가 나왔다면 에어건을 사용하여 진드기를 털어내고 전신을 샤워하여 진드기의 유무를 확인한다. 옷을 세탁할 경우 야외복을 분리하여 세탁해야한다. 진드기는 흔적을 남기기 때문에 증상(고열/오한, 두통, 근육통, 오심/구토/설사)의 유무를 확인하고 이 증상은 위험활동 1~3주 후 발생한다. 두번째로는 물린흔적을 찾아본다. 검은딱지가 있거나 빨간 자국이 대표적이다. 증상(고열/오한, 두통, 근육통, 오심(구토/설사)가 나타났거나 검은딱지, 물린자국을 발견했다면 가능한 빨리 병원에가서 치료 받아야한다. 2013~2017년 기준 SFTS가 5명중 1명이 사망한것으로 통계가 나타났기 때문 군산시에 농사일을 하시는 분들이 많아 이러한 안내문을 같이 동봉하여 보낸다는 사실을 새롭게 알게되었다.

4. 오늘 실습을 통해 새롭게 배운 것들이 향후 간호실무에 어떻게 적용될 수 있겠는가?

• 뇌졸중이 무엇인지, 위험요인은 무엇이며 뇌졸중의 증상이 어떤것인지 알고 증상발생시 바람직한 응급처치를 실시할수있다. 빠른 치료의 중요성을 알며 뇌졸중후 조기 재활치료 가 중요함을 설명하고 재활치료를 빠르게 실시하여 합병증을 예방하고, 기능적 독립성을 최대한으로 회복하고, 사회생활로 복귀할 수 있도록 도울수있을것이다.

• 방문건강 간호사가 하는 일을 알며, 방문건강간호사의 다양한 역량을 파악하여 방문건강 간호사가 되었을 경우 대상자의 특성에 맞는, 대상자가 필요로하는 것을 파악하여 대상자가 가정에서 건강수준을 유지하고 증진 시킬수 있도록 옆에서 도움을 줄 수있을것으로 기대된다.

5. 오늘 실습에서 좋았던 점과 아쉬웠던 점은 무엇인가?

• 오후에 경로당으로 실습을 나가 여러 대상자를 만나고 혈압. 혈당 측정 등의 활동을 할수 있을것으로 생각하고 기대하고 있었으나 갑자기 보건교육실에서 지역사회 건강조사 우편보낼 우편물을 봉투에 넣고 스티커 작업을 하라고 하여서 경로당에 나가보지 못해 아쉬웠다. ?

노인 간호

방문

지역 특성

진로선[illegible]

2-4

CamScanner로 스캔하기

1. 오늘 실습경험의 의미 있는 점은 무엇인가?

•구강보건센터에서 치아홈메우기를 하기위해 방문하는 분들이 있다. 치아홈메우기란 충치가 생기기 쉽고, 칫솔이 잘 닿지 않는 어금니 치아의 씹는 면의 좁고 깊은 골짜기 부분을 재료로 메워 세균이나 음식물의 찌꺼기가 홈에 끼지 못하게 함으로써 충치를 예방하는 치료이며 만 18세 이하 소아·청소년을 대상으로 대상치아는 충치가 없는 첫번째 또는 두번째 어금니이다.
• 치석제거(스케일링)은 잇몸병의 가장 큰 원인인 치아표면에 붙어있는 치석과 세균을 제거하고 치아표면을 매끄럽게 하여 세균부착을 방지하기위한 목적의 예방조치이며 만 19세 이상을 대상으로 연간 1회 유효이다.
우리나라에서 보험급여가 적용되는 치아홈메우기, 치석제거에 대해서 알 수 있어 의미 있었다.

2. 오늘 실습을 하는 동안 나의 생각과 느낌은 어떠했는가?

• 오늘실습을 진행하며 우리나라에서는 국민의 구강질환을 예방하기위해서 '치아홈메우기(실런트)', '치석제거(스케일링)'부분을 보험급여를 적용하고있어 국민들이 조금더 저렴한가격으로 구강검진을하고 구강건강에 대해 관리할수 있게 하여 좋은 사업이라는 생각을 하게 되었고 이러한 사업을 잘알고 이용해야겠다는 생각을 하게 되었다.
• 구강검진을 통하여 치아우식증, 결손치아, 아말감, 잇몸병, 치석, 톱니상태, 구강 암 및 구강연조직 이상에 대한 검사를 진행하며 구강건강상담을 통하여 의료이용 및 주관적 상태, 구강관련습관, 구강기능 상태 등을 검진받을수 있다. 나는 평소 구강검진을 필요치로 2년마다 돌아오는 구강검진을 받지 않았었는데 내게 많은 도움이 되고 내가 혹시라도 가지고 있을수 있는 나쁜습관들을 검진을 통해 찾고 이를 고쳐나갈수 있도록 해야겠다는 생각을 하게 되었다.

본인의 건강관리

3. 오늘 실습을 통해 새롭게 알게 된 것은?

• 충치균이 치아표면에 남아있는 당과 탄수화물을 먹고 충치균을 분해하여 산을 만들어낸다. 치아에서 연약한 부분은 산의 공격으로 충치가 생기기 때문에 치실을 사용해야한다. 치실을 45cm 정도로 자른후 한쪽 치아면을 감싸 C형태로 만들고 반복한다. 치간 칫솔을 사용할 때에는 치간 칫솔의 삽처가 치아나 잇몸에 닿지않는 크기인 것으로 선택하여 90°로 구부린후 사용한다.
• 칫솔을 보관할때는 칫솔모 사이에 남아있는 치약이 없도록 깨끗하게 헹궈 낸다음 물기를 제거하고 칫솔모가 위를 향하도록 보관해야 하며 다른 칫솔의 칫솔모와 닿지않게 보관해야 한다. 치약은 불소성분이 함유되어있는 것을 사용하는것이 좋다. 불소는 치아를구성하는 '수산화인회석' 성분과 결합하여 치아를 더 튼튼하게 만들고 충치로부터 치아를 보호해 줄수 있다. 성인의 경우 불소도포를 받아 치아의뿌리가 충치가 생기는 것을 예방하고, 치아가 시린증상을 완화할수 있으며 소아·청소년은 6개월에 한번씩 불소도포를 받아 충치를 예방할수 있다.
• 영유아는 유치를 가지고있지만 구강검진을 받아야한다. 18~29개월, 42~53개월, 54~65개월, 66~71개월일때 구강검진을 받으며 검진을통하여 충치유무, 부정교합, 구강위생상태, 연조직질환 등 구강 내 이상소견이 있는지 확인할수 있다.

4. 오늘 실습을 통해 새롭게 배운 것들이 향후 간호실무에 어떻게 적용될 수 있겠는가?

오늘 실습을 통해서 보험급여가 적용되는 건강검진에는 '치석제거(스케일링)', '치아홈메우기(실런트)'가 있다는 것과 이 두 사업이 어떻게 시행되는지, 대상은 누구인지 알게되었기에 이 사업이 필요하고, 적용되는 사람들에게 사업을 알려주고 검진을 받을 수 있도록 독려할수 있을 것으로 기대된다.
충치균에 대해, 칫솔 보관법, 불소 관련된 내용에 대해 알게되었는데 나중에 뒤돌아 보니 이러한 간단하고 지나칠수 있는 내용들을 자기관리가 부족한 사람들에게 알려줄수있고 이러한 것들을 알려줌으로써 대상자 스스로가 자신의 구강위생을 챙겨서 더 건강한 삶을 살수있도록 도와줄수 있을것으로 기대된다.

2-3

간호역량 강화
전문직: 교육

5. 오늘 실습에서 좋았던 점과 아쉬웠던 점은 무엇인가?

•원래는 오늘 이동진료 실습이었으나 오늘은 출장이 없다고 하여서 구강보건 센터에서 실습을 하게 되었다.
이동진료를 가지못해 경로당 등에서 실시하는 간호를 관찰하지 못하여 많은 아쉬움들이 있지만 구강보건 센터에서 실습을 하며 다양한, 새로운, 여기서 실습하지 못했다면 그냥 지나쳤을 내용들을 알게된 점이 좋았다.

CamScanner로 스캔하기

1. 오늘 실습경험의 의미 있는 점은 무엇인가?

• 치매안심센터에서 어떠한 업무를 하는지 알게된 점이 의미 있었다. 60세 이상 어르신들을 대상으로 조기검진을 실시하고, 60세이상 어르신, 치매환자, 보호자를 대상으로 상담. 등록 후 치매치료비 지원, 조호물품지원, 사례관리 서비스, 실종예방 서비스를 제공한다. 또한 어르신들을 대상으로 치매 인식개선 개선한 교육을 진행하며 치매 파트너, 파트너 양성교육을 진행한다. 치매 안심센터에서 진행하는 프로그램에는 치매환자 쉼터, 치매 예방교실, 인지강화교실, 치매 가족 지원 교실이 있다는 것을 알게되었다.

2. 오늘 실습을 하는 동안 나의 생각과 느낌은 어떠했는가?

• 치매분소에서 실습을 진행하며 생각보다 많은 사업(상담·등록, 조기검진, 인식개선 프로그램)들을 진행한다는것을 알게 되었으며 치매 분소에서 조기검진을 받기위해 방문하시는 어르신 분들이 생각보다 많아 좀 놀랐다. 또한 치매 등록을 하게되면 지원 되는 서비스가 있는데 중위소득 120% 이하 치매환자에게 진료비/약제비 3만원을 지원해주며 물품지원서비스(거동이 불편한 분들에게 기저귀, 물티슈 지원)가 있는데 1년치를 1회만 지급이되며 수급자, 의료급여 수급자의 경우 재신청이 가능하다. 하지만 이러한 지원이 치매치료를 받고있는 분들에게는 너무 부족하다는 생각이 들어서 지원 금액등을 더 높이면 좋겠다는 생각을 하게되었으며 생각보다 치매관련 사업이 많아 치매가있는 대상자들이 많이 이용하면 좋을것 같다는 생각이 들었다. # 노인간호 치매 검진 # 노인. 치매 지원

3. 오늘 실습을 통해 새롭게 알게 된 것은?

• 조기검진은 60세 이상 관내 어르신들이 대상이며, 먼저 인지선별검사를 진행한다. 인지선별검사(CIST)는 학력, 나이에 따라 <정상/인지저하>로 나뉘지며 인지저하로 검사결과가 나올경우 정밀검사에 대해 안내해주고 연계해준다. 정밀검사(SNSB)는 인지선별검사에서 인지저하가 나오신분들을 대상으로 실시하며 기억력, 지남력, 주의력, 시공간기능, 집행기능, 언어기능 등의 영역검사를 문답식으로 진행한다. 만약 검사결과가 경도인지장애라고 판정된다면 1년마다 재검사를 실시하며 치매가 의심된다면 다른기관에 연계한다. 보건소는 의사가 없기때문에 보건소에서는 진단내릴수 없다. 병원에서 실시하는 감별검사는 병원(협력병원)에서 실시하는 치매 진단을 위해 MRI 또는 혈액검사를 진행하여 치매를 진단할수 있으며, 중위소득 120% 이하의 대상자에게는 감별검사비를 지원해 준다는 사실을 알게되었다.

4. 오늘 실습을 통해 새롭게 배운 것들이 향후 간호실무에 어떻게 적용될 수 있겠는가?

오늘실습을 통하여 치매 조기검진을 필요로하는 어르신들에게 CIST(인지선별검사)를 권할수있으며 치매 대상자들에게 인지활동교육(미술치료, 원예치료, 운동치료)를 권하며, 치매 환자 및 경도인지 장애 환자를 돌보는 가족 및 보호자를 대상으로 돌봄교육 및 스트레스 해소프로그램을 진행할수 있으며 치매진단을 받지 않은 60세 이상의 어르신들을 대상으로 치매예방을 위한 인지활동교육을 권할수 있을것이다. 또한 치매 인식개선을 위하여 경로당이나 복지관 실외에서 60세 이상 어르신 들을 대상으로 치매 인식에 대한 기본정보를 제공한후 인식개선교육을 실시할수 있을것으로 기대된다.

5. 오늘 실습에서 좋았던 점과 아쉬웠던 점은 무엇인가?

• 치매분소에서 실습을 하며 치매 안심센터에서는 어떠한 업무를 진행하는지에 대해 알게되어 좋았으며 치매환자쉼터, 치매예방교실, 인지강화교실, 치매 가족 지원교실, 치매 상담·등록, 치매인식 개선교육등을 사업을 진행 한다는것을 알게된점이 좋았다.
• 치매 안심센터가 아닌 치매분소이다보니 하는일이 조금 제약적이라서 치매 안심센터도 가보면 좋았을텐데 하는 아쉬움이 들었다.

1. 오늘 실습경험의 의미 있는 점은 무엇인가?

오늘 처음으로 군산시 보건소에서 실습을 진행한 점이 의미 있었고 간단한 OT 및 보건소 내부 부서를 알게된 점이 의미있었다.

2. 오늘 실습을 하는 동안 나의 생각과 느낌은 어떠했는가?

오늘은 방문건강관리실로 출근하였는데 직접 방문간호를 진행하게 되어 좋았고, 대상자의 집에 방문하여 오랫동안 "오늘 건강" 프로그램 참여를 하지 않는 분이어서 혈당, 혈압 측정을 도와드렸고 말씀을 나누어 어떤 과거력과 가족력이 있는지 듣고 파악할 수 있어서 좋았다.

3. 오늘 실습을 통해 새롭게 알게 된 것은?

AI-IOT 기반 어르신 건강관리사업 스크리닝 도구는 450명 기준으로 "오늘 건강" 프로그램을 하기 전 사전조사를 통해 대상자를 사정하는 도구이다. 항목으로는 신체활동 실천현황, 식생활 실천현황, 만성질환 이환여부, 만성질환 관리현황, 허약평가, 보행(TUG), 3가지 단어 회상하기, 시계 그리기 등이 있다. 이를 통해 대상자를 파악 후 "오늘건강" 앱을 설치하여 혈압, 혈당 등을 스스로 측정할 수 있게 한다. 결과가 보건소에 옴으로써 대상자를 쉽고 간편하게 파악할 수 있으며 질병에 대해 미리 대비할 수 있다.

4. 오늘 실습을 통해 새롭게 배운 것들이 향후 간호실무에 어떻게 적용될 수 있겠는가?

향후 간호사가 되어 실습을 바탕으로 대상자와 공감하여 개방형 질문을 통해 소통할 것이고, 혈당 및 혈압 측정을 정확하게 진행할 것이다.

의사소통 역량 ?

5. 오늘 실습에서 좋았던 점과 아쉬웠던 점은 무엇인가?

좋았던 점은 직접 방문간호를 나가서 대상자와 소통할 수 있어서 좋았고 보건소 내부의 부서, 치매 교육, 진드기 매개 감염병 교육, 한국보험공단 특강을 듣게 되어 좋았다.

CamScanner로 스캔하기

1. [illegible]

예방접종실로 출근하여 유료 예방접종 종류 (A형간염, 신증후군 출혈열, 장티푸스, 추가 B형 간염), 인유두종 바이러스 (HPV) 예방접종 지원백신 (HPV 2가 서바릭스, HPV 4가 가다실), 65세 어르신 폐렴구균 유료 예방접종 등 추가 보건소에서 어떤것이 접종가능한지 알게된 점이 의미있었다.

2. 오늘 실습을 하는 동안 나의 생각과 느낌은 어떠했는가?

이른 아침부터 HPV, B형간염 예방접종을 받으러 보건소로 방문하신 분들이 많다는 것을 알게 되었고 예진표 작성 및 체온측정을 도와 드리며 잠깐이나마 소통하고 예방접종 후에는 사우나 찜질방은 피하도록 하면 상기해달라고 안내해 드리며 교육한 점이 좋았고 뿌듯하였다.

3. 오늘 실습을 통해 새롭게 알게 된 것은?

결핵 (BCG) 예방접종의 종류로는 BCG 피내 접종(주사형), BCG 경피접종 (도장형)이 있는데 피내접종은 피부에 약 15도 각도로 바늘 사면을 완전히 삽입한 후 백신을 주입하는 것이다. 경피접종은 피부에 주사액을 바른 후 9개의 바늘을 가진 주사도구를 이용하여 두번에 걸쳐 강하게 눌러 접종하는 것이다.
피내접종은 BCG 접종량이 상대적으로 일정하고 정확하지만 경피접종은 접종량이 일정하지 않을 수 있는 특징이 있다는 것을 알게되었다.

4. 오늘 실습을 통해 새롭게 배운 것들이 향후 간호실무에 어떻게 적용될 수 있겠는가?

향후 간호사가 되어 실습한 걸 바탕으로 결핵(BCG) 예방접종 후 나타날 수 있는 이상반응(국소농양, 림프절염, 기타 이상반응)에 대해 교육할 것이고, 예방접종 후 관리 (깨끗한 옷으로 갈아입고 접종부위를 깨끗하게 해준다 등)에 대해 교육할 것이다.

5. 오늘 실습에서 좋았던 점과 아쉬웠던 점은 무엇인가?

좋았던 점은 보건소에서 어떤 것이 접종 가능한지에 대해 알고 가능한 예방접종은 어떤 것들이 있는지를 알게되어 좋았다.
아쉬웠던 점은 직접 예방접종 하는 모습을 관찰할 수 없는 것이 아쉬웠다.

CamScanner로 스캔하기

1. 오늘 실습경험의 의미 있는 점은 무엇인가?

오늘은 건강보험공단에서 실습을 하여 전화를 통해 암검진 및 일반검진 유선독려하는 업무를 하게 된 점이 의미있었다.

2. 오늘 실습을 하는 동안 나의 생각과 느낌은 어떠했는가?

전화를 해야되기에 많이 긴장되고 떨렸지만 여러번 하다보니 점차 익숙해져가는 나의 모습을 보며 뿌듯하였다. 가끔 "제가 왜 검진을 받아야되요?", "검진 받고 안받는 저의 자유 아닌가요" 등의 말을 들으며 당황하기도 했지만 새로운 경험이 된 것 같아서 좋았고 실습하여 대처능력을 키울수 있어서 좋았다.

3. 오늘 실습을 통해 새롭게 알게 된 것은?

국가 6대 암검진으로는 위암, 대장암, 간암, 폐암, 유방암, 자궁경부암이 있다. 위암은 2년 주기로 40세 이상 남·여가 대상자며 검진비용 10%의 본인부담이 발생한다. 간암으로는 40세 이상 간암발생 고위험군 남·여가 대상이며 검진 비용 10%의 본인부담이 발생한다. 간암발생 고위험군 기준은 해당연도 전 2년간 보험급여내역 중 간암발생 고위험 질병으로 진료 받은 내역이 있는자, 과거연도 B형간염표면항원 또는 C형간염항체 검사결과가 양성으로 확인된자가 해당된다는 것을 알게 되었다.

4. 오늘 실습을 통해 새롭게 배운 것들이 향후 간호실무에 어떻게 적용될 수 있겠는가?

향후 간호사가 되어 실습에 경험했던 것을 바탕으로 시시각각 변화하는 환자 상태를 자세히 관찰하여 올바른 간호로 대처능력을 키울 것이다.
또한, 환자에게 교육시킬 때 정확한 지식을 바탕으로 올바르게 교육할 것이다.

5. 오늘 실습에서 좋았던 점과 아쉬웠던 점은 무엇인가?

좋았던 점은 전화업무를 하며 대처능력을 키우고 소통할 수 있었던 점이 좋았고, 국가 암검진, 일반검진에는 어떤 것들이 있고 대상으로 어떤 나이가 해당되는지를 알 수 있어서 좋았다.

CamScanner로 스캔하기

1. 오늘 실습경험의 의미 있는 점은 무엇인가?

오늘 구강보건센터에서 실습을 진행하며 불소양치 용액 배부 및 예약자 (치아 홈메우기) 를 안내하는 역할을 한 점이 의미있었다.

2. 오늘 실습을 하는 동안 나의 생각과 느낌은 어떠했는가?

지역사회 사례보고서 프로그램을 구강보건쪽으로 계획을 세워 만들었기에 과제를 통해 알은 정보를 습득하였는데 실제로 군산시 보건소에서도 시행하고 있음을 알게 되어 신기하였고 미리 알고 있어서 뿌듯하였다.

치아홈메우기 사업은 어린이를 대상으로 의사선생님이 직접 관찰하여 홈메우기가 필요한 부분은 진행하고 불소도포가 필요한 경우도 선택적으로 진행하는 모습을 관찰할 수 있어서 좋았다.

3. 오늘 실습을 통해 새롭게 알게 된 것은?

구강보건센터란 기존 보건소 구강보건실을 확대, 전환하여 인적, 물적 자원을 강화함으로써 보건지소와 보건소로 분산되어 있는 진료체계를 구강보건센터 중심의 조직적이고 효율적인 구강보건진료 사업구조로 변화, 취약계층의 구강진료 및 예방활동 접근성을 확보하기 위해 만들어졌음을 알게 되었고 시설로 구강진료실, 구강보건교육실, 양치 체험교실, 구강보건 사업실로 구성되어 있음을 알게 되었다.

? 책자

4. 오늘 실습을 통해 새롭게 배운 것들이 향후 간호실무에 어떻게 적용될 수 있겠는가?

향후 간호사가 되어 실습 때 경험했던 것을 바탕으로 정보를 미리 습득함으로써 올바른 간호를 제공할 것이고, 환자에게 교육시킬 때 정확한 지식을 바탕으로 올바르게 교육할 것이다.

△

5. 오늘 실습에서 좋았던 점과 아쉬웠던 점은 무엇인가?

좋았던 점은 보건소에 방문하시는 분들과 직접 소통하여 안내해드릴 수 있어서 좋았고 오늘은 부담이 없음 편하게 질문하라고 해주셔서 긴장하지 않고 편하게 실습하게 임할 수 있어서 좋았다.

의사소통

1. 오늘 실습경험의 의미 있는 점은 무엇인가?

오늘은 접수실에서 실습을 진행하여 민원에 대한 안내를 해드린 점이 의미 있었다.

2. 오늘 실습을 하는 동안 나의 생각과 느낌은 어떠했는가?

코로나 19 검사, 적성검사, 결핵검사, 치매상담, 검사, 사전연명의료의향서 신청, 임신부 상담, [illegible] 어르신 AT건강관리 상담 등 어느 과에서 실시하고 있는지 안내해드리며 몇 층에 위치하고 있는지 안내를 도와드리며 많은 분들이 [illegible] 다양한 이유로 보건소에 방문하시는 구나를 깨닫게 되었고 잠깐이나마 소통할 수 있어서 좋았다.

3. 오늘 실습을 통해 새롭게 알게 된 것은?

관산시 보건소 1층엔 접수실, 진료실, 예방접종실, 건강검진실, 금연상담실, 영양플러스실, 적성검사실, 결핵관리실, 모자보건 상담실, 검사실, 치매안심센터가 배치되어 있다. 또한 2층엔 한방실, 물리치료실, 재활치료실, 구강보건센터, 방문건강실이 배치되어 있다. 3층엔 보건행정과, 건강관리과, 감염병관리과가 배치되어 있음을 알게 되었고, 보건소 방문이유 중 가장 많은 이유가 보건증 발급이라는 것을 알게 되었다.

4. 오늘 실습을 통해 새롭게 배운 것들이 향후 간호실무에 어떻게 적용될 수 있겠는가?

향후 간호사가 되어 실습때 경험했던 것을 바탕으로 개방형 질문을 통해 소통할 것이고, 정확한 정보만을 제공할 것이다. 모르거나 정확하지 않은 정보는 질문을 통해 안전한 간호를 제공할 것이다.

의사소통

5. 오늘 실습에서 좋았던 점과 아쉬웠던 점은 무엇인가?

좋았던 점은 사람들과 소통하여 [illegible] 알고 있는 정보를 바탕으로 안내해드릴 수 있어 뿌듯하였고, 선생님들끼리 상호협력적인 관계를 유지하여 질문을 통해 민원을 해결하려고 하는 모습을 관찰하게 되어 향후 나 또한 상호협력적인 관계를 유지해야겠음을 깨달았다. 부족한 점은 [안내들] 민원하러 와셔서 편하게 실습할 수 있어 좋았다.

CamScanner로 스캔하기

1. 오늘 실습경험의 의미 있는 점은 무엇인가?

보건소에서 무료접종이 가능한 예방접종의 종류, 대상 등을 정확히 알 수 있었으며 유료 예방접종의 종류에 대해서도 알 수 있었습니다

→ ① 유아 B형 간염
- 3회 접종 (0-1-6개월 간격)
- 성인 : 5800원 (만 10세까지 3400원)

② 장티푸스
- 1회 접종으로 3년간 유효
- 항체형성 기간 2주 소요
- 접종비 : 8000원

③ 신증후군 출혈열
- 3회 접종 (0-1-12개월 간격)
- 접종비 : 8200원
(단 2014년 이전 접종자 및 기초생활수급자는 무료)

④ A형 간염
- 2회 접종 (0-6개월 간격)
- 성인 : 33000원 (만 15세까지 11000원)
(단, 40세 이상인 경우 항체검사 결과지 지참 필수)

2. 오늘 실습을 하는 동안 나의 생각과 느낌은 어떠했는가?

3. 오늘 실습을 통해 새롭게 알게 된 것은?

• 인유두종 바이러스 (자궁경부암, HPV) 예방접종

접종 대상
- 만 12~17세 여성 청소년
: 2005.1.1. ~ 2011.12.31. 출생자
- 만 18~26세 저소득층 여성
: 1996.1.1. ~ 2004.12.31. 출생자

지원 백신
- HPV 2가 (서바릭스)
- HPV 4가 (가다실)
*HPV 9가 (가다실9) 백신 미지원

지원 내용
- HPV 예방접종
→ 1차 만 12~14세 접종 : 총 2회 접종
(0-6~12개월 간격)
→ 1차 만 15세 이상에서 접종 : 총 3회 접종
→ HPV 2가 (0-2-6개월 간격), HPV 4가 (0-1-6개월 간격)
- 건강상담 : 사춘기 성장발달 및 [illegible] 관련 상담
→ 만 12세 여성 (2010.1.1. ~ 2011.12.31. 출생) [illegible]

• 65세 어르신 폐렴구균 무료 예방접종

접종대상 1958년 이전 출생 어르신 중 미접종자 (평생 1회 무료 지원)
지원백신 폐렴구균 23가 (다당질 백신)
접종기관 [illegible] 보건소
(평일 오전 9시~오후 5시, 점심시간 12시~13시 제외)
접종기간 연중
준비사항 신분증 지참 (주민등록증, 여권 등)

4. 오늘 실습을 통해 새롭게 배운 것들이 향후 간호실무에 어떻게 적용될 수 있겠는가?

5. 오늘 실습에서 좋았던 점과 아쉬웠던 점은 무엇인가?

각각의 예방접종에 대해 상세히 설명을 들었고 예방접종을 하러 온 대상자를 직접 안내하고 체온측정 및 예진표 작성 돕기 등을 해보아서 좋았습니다

1. 오늘 실습경험의 의미 있는 점은 무엇인가? 보건소 내 금연 클리닉 안내

<금연의 절차>

① [illegible]하기

-나의 금연동기 구체적으로 적어보기

② 정하기

-금연시작일 : 흡연과 이별하는 날 [illegible]

<12개월 금연 상담 프로그램>

-니코틴 대체요법

니코틴이 포함된 제재로 패치 껌 [illegible] 피우는 구강흡연을 [illegible] 감소

종류: 니코틴 패치, 니코틴 껌, 니코틴 캔디

2. 오늘 실습을 하는 동안 나의 생각과 느낌은 어떠했는가?

③ 알리기

-"나 오늘부터 금연하기로 했어."

-"앞으로는 내게 담배를 권하지 않아주면 좋겠어."

④ 예상하기

-금연을 방해하는 요인들 : 금단증상.

-실패에 대한 두려움

-대체 행동요법

담배를 피우고 싶은 욕구는 대부분의 경우 3~5분 정도 지속되며 계속되지 않는다. 그러니 그 욕구가 사라질 때까지 기다려보세요.

* 욕구를 버티는 방법들

(입으로 담배 잊기) (머리에서 담배 잊기) (신체활동하며 담배 잊기) (몸으로 담배 잊기)

- 물을 마신다
- 심호흡을 마신다.
- 과일을 먹는다.
- 껌을 씹는다.
- 무가당 사탕을 먹는다
- 비타민을 섭취한다
- 차가운 [illegible] 마신다

- 일단 하던 일을 멈추고 다른 일을 해본다.
- 눈을 감고 흡연 욕구가 사라질 때까지 기다린다.
- 명상을 한다
- 아무 [illegible] 누워서 [illegible]

- 숫자 [illegible] 세면서 [illegible]
- [illegible]
- [illegible]

- 샤워를 한다
- [illegible]
- 이 [illegible]
- [illegible]

3. 오늘 실습을 통해 새롭게 알게 된 것은?

-흡연의 결과

⑤ 치우기

-담배, 흡연관련 물품들, 집과 사무실, [illegible]

⑥ 멀리하기

-더 이상 담배를 사지 않기

4. 오늘 실습을 통해 새롭게 배운 것들이 향후 간호실무에 어떻게 적용될 수 있겠는가?

<금연 상담 서비스 기관>

① 등록

-금연클리닉 등록카드에 등록

-흡연자 자가 실시 → 금연계획 수립

② 내소별 내용

-니코틴 의존도 및 대체요법, 약물 제공

-12개월 상담 + 전화 상담 (6개월간 총 9회 이상)

5. 오늘 실습에서 좋았던 점과 아쉬웠던 점은 무엇인가?

③ 기간

-6개월 상담서비스 제공

④ [illegible]

-[illegible] 4주, 6주, 12주

⑤ [illegible]

-[illegible]

-6개월 금연 [illegible] 제공 [illegible]

CamScanner로 스캔하기

1. 오늘 실습경험의 의미 있는 점은 무엇인가?

[illegible]

[illegible]

[illegible]

[illegible]

2. 오늘 실습을 하는 동안 나의 생각과 느낌은 어떠했는가?

[illegible]

1) [illegible]

2) [illegible]

3) [illegible]

[illegible]

1) The [illegible] → [illegible] iN → [illegible]

→ [illegible]

3. 오늘 실습을 통해 새롭게 알게 된 것은?

[illegible] iN → [illegible]

[illegible]

[illegible]

[illegible]

1) [illegible]

[illegible] + [illegible]

4. 오늘 실습을 통해 새롭게 배운 것들이 향후 간호실무에 어떻게 적용될 수 있겠는가?

[illegible]

[illegible]

[illegible]

① [illegible]

② [illegible]

③ [illegible]

5. 오늘 실습에서 좋았던 점과 아쉬웠던 점은 무엇인가?

④ [illegible]

⑤ [illegible]

⑥ [illegible]

CamScanner로 스캔하기

1. 오늘 실습경험의 의미 있는 점은 무엇인가? [방문건강관리실]

실습 첫 날, 보건소 실습에 대한 오리엔테이션을 받았다. 치매인식개선 & 치매 파트너 교육, 건강보험관리공단, 감염병 예방관리(진드기 매개 감염병)에 대한 교육을 받았다.
"희망동 희망루 아파트"로 방문 간호를 다녀왔다. (5명)

2. 오늘 실습을 하는 동안 나의 생각과 느낌은 어떠했는가? 정확히 어떤 역할을 하는지 알지 못했다.

• 군산시 정신건강복지센터 실습을 하며 치매안심센터를 접하게 되었는데 오늘 OT와 교육을 통해 어떤 역할을 수행하고 왜 필요한지 등을 학습할 수 있어서 궁금증이 해결되었다.
• 국민건강보험공단을 간호관리학 이론 시간이나 지역사회간호학 이론 시간에 학습했었는데 직접 보험공단의 관계자분(팀장님)을 만나 학습할 수 있어서 신기하고 좋았다.
• 지역사회간호학2 레포트로 군산시 보건소의 조직도를 작성한 적이 있었다. 그때 정리하여 학습했던 내용보다 더 구체적으로 어떤 일을 하는지 알게 되었고, 이는 직접 [illegible]보지 않으면 모르는 사항이라 더 의미있게 느껴졌다.

3. 오늘 실습을 통해 새롭게 알게 된 것은?

우리나라는 전 세계에서 치매환자 증가속도가 가장 빠르고, 80세 이상 노인 중 1명이 치매환자이다. 치매안심센터는 치매조기검진, 치매예방관리, 치매환자쉼터, 맞춤형 사례관리, 치매치료 관리비 지원, 치매인식개선 교육·홍보·캠페인, 배회가능어르신 인식표 발급, 치매안심 마을, 조호물품 지원 등을 하고 치매환자 가족(보호자)상담, 힐링프로그램, 가족교실 등 치매환자의 가족에 대한 교육및 관리도 함께 한다. 치매 초기 환자 중에 복지 사각지대에 놓인 이들을 위해 치매환자 쉼터도 제공한다.

4. 오늘 실습을 통해 새롭게 배운 것들이 향후 간호실무에 어떻게 적용될 수 있겠는가?

- 일상생활에서 치매환자의 초기 증상과 중기 증상 (최근의 일기는 일 기억 못함, 적절한 단어 선택이 어려워 [illegible]) (혈관 비밀번호 잊음, 표현력·이해력 저하) 말기 증상을 (모든 언어능력상실, 의미있는 문장X) 구분할 수 있고, 치매 환자를 접했을 시 도움을 줄 수 있을 것이다.
- 치매 환자에 대해 흔히 오해하고 있는 사항들에 대해 알고 오해하는 사람들에게 설명할 수 있을 것이다.
<치매는 뇌의 질병이다. 다양한 증상이 동반된다. 앓은 것들을 느낄 수 있다. 치료관리를 통해 증상악화를 지연시킬 수 있다. 치매는 예방이 필요하다. (가능하다.)>

(+) (−)
5. 오늘 실습에서 좋았던 점과 아쉬웠던 점은 무엇인가?

(+). 실습 첫 날에 오리엔테이션을 구체적으로 들을 수 있어서 좋았다. (군산시보건소, 건강보험관리공단, 감염병예방관리(진드기 매개 감염병), 치매인식개선 & 치매 파트너 교육)
- 방문 간호를 가서 독거노인분들의 혈압과 혈당을 측정하고, 대상자의 이타의 건강상태를 사정하고, 개선방향을 교육하는 모습을 관찰할 수 있었다.
- 방문 간호 시, 공감과 경청을 기반으로 한 치료적 의사소통을 관찰할 수 있었다.
- 선생님들께서 계속 챙겨주시고 신경 써주셔서 좋고 감사했다.

간호사 역량

(−) 방문 간호시, 다른 집으로 이동할 때 손위생을 하지 않아서 조금 걱정이 되었다.

CamScanner로 스캔하기

1. 오늘 실습경험의 의미 있는 점은 무엇인가? [방문건강관리실]

관원 경로당으로 방문간호를 다녀왔다.

군산시 보건소에서 보건지소의 업무도 일부한다고 하셨다.

경로당 (전부하기엔 어려움이 있음).

교수님께서 순회지도를 나오셨다. 과제에 대해 궁금했던 사항에 대해 궁금증을 해소할 수 있었다.

2. 오늘 실습을 하는 동안 나의 생각과 느낌은 어떠했는가?

어제에 이어 오늘도 방문간호를 갔다. 어제와 달리 오늘은 경로당으로 갔다. 거리가 좀 있어서 30분 정도 걸려 도착했는데 농번기이고 연락드린 분이 이장님도, 경로당 회장님도 아니셔서 한 분도 안계셨다. 함께 가신 선생님께서도 이런 경우는 처음이라 당황스러워 하셨다. 다른 경로당에도 연락을 해봤으나 다들 바쁘셔서 뒤늦게 오신 세 분의 BP 및 BST를 측정하고 돌아왔다. 지역사회 간호에서는 지역사회 주민들의 일상을 이해하고 고려하여 간호를 제공해야 함을 배웠다.

지역사회 특성

3. 오늘 실습을 통해 새롭게 알게 된 것은?

• 방문건강 관리 사업이란?

: 보건소의 소속된 방문간호사가 각 가정을 방문하여 가족과 건강문제를 가진 가구원을 발견하여 질병예방 및 관리, 건강증진을 위하여 건강 서비스를 제공하는 것.

• 방문건강 관리의 목표.

① 건강 행태 개선 (건강상태 인식, 건강생활 실천 유도, 건강지식 향상)

② 건강문제 관리 (건강문제 정기적 스크리닝, 증상 조절, 치료 순응 향상)

• 방문간호 대상 : 기초수급자, 독거노인, 차상위계층, 장애인, 독거노인, 타기관에서 의뢰한 건강 문제가 있는 대상자, 지역사회 환자 관리, 재가 장애인 / 집단 서비스 - 경로당.

4. 오늘 실습을 통해 새롭게 배운 것들이 향후 간호실무에 어떻게 적용될 수 있겠는가?

~~방문 간호 등 지역사회 주민을 대상으로 한 간호를 계획할 시, 대상자들의 직업과 일과를 이해하고 그에 맞춰 계획을 세울 수 있을 것이다.~~

사회가 고령화될수록 방문간호 사업의 중요성이 높아지는데 방문 간호의 필요성과 중요성에 대해 알고 적절하게 설명할 수 있을 것이다.

5. 오늘 실습에서 좋았던 점과 아쉬웠던 점은 무엇인가?

(+) 가정이 아닌 경로당에서 하는 방문간호는 어떻게 하는지 학습할 수 있었다.

보건소에서 보건지소의 업무 중 일부를 나누어 할 수 있음을 배웠다. (예시)

지역사회 주민을 대상으로 하는 간호에서 고려할 사항을 학습했다.

(-) 방문간호를 하러 먼 길을 갔는데 대상자분들이 많이 계시지 않아 아쉬웠다.

(3명)

1. 오늘 실습경험의 의미 있는 점은 무엇인가? [이동진료팀].

이동진료팀에 배정받았으나 오후에 있던 출장이 취소되었다.
자습을 하다가 오후에 "지역사회 건강조사"에 대한 안내문을 발송하기 위해
우편물을 준비하러 갔다.

2. 오늘 실습을 하는 동안 나의 생각과 느낌은 어떠했는가?

이동진료와 방문간호의 차이를 알아볼 수 있을 것 같아 이동진료를 약간 기대했는데
가지 못해서 아쉬웠다. 우편물을 정리하는 일을 하러 가서 처음에는 일이 주어져서 좋았지만
몇 백 개를 만들다 보니 조금 힘들었다. 우편물에는 코로나 19 예방, 안내서, 지역사회건강조사
책자, 우울증·자살 예방, 국가암검진사업에 대한 내용이 포함됐다.

3. 오늘 실습을 통해 새롭게 알게 된 것은?

· 지역사회건강조사란? : 지역 건강통계를 산출하여 지역별로 꼭 필요한 근거 중심의 보건사업을
수행하기 위해 지역주민의 건강행태(흡연, 음주 등) 및 이환, 의료이용 등을 조사한다.

· 조사대상 : 표본가구 가구원 중 만 19세 이상 성인. · 조사단위 : 시·군·구 보건소 당 900명

· 조사 방법 : 조사원이 표본가구를 직접 방문하여 일대일 면접조사 실시 (조사원이 태블릿PC를 이용하여 응답내용 기록)

· 조사내용 : 가구조사 (세대유형, 기초생활수급 여부, 가구소득 등), 건강행태(흡연, 음주, 안전의식,
신체활동, 식생활, 비만 및 체중조절, 건강지식, 구강건강, 정신건강), 예방접종 및 검진 (인플루엔자 예방접종, 건강검진 및 암 검진 수진율 등.

4. 오늘 실습을 통해 새롭게 배운 것들이 향후 간호실무에 어떻게 적용될 수 있겠는가?

이환 (고혈압 진단 경험, 당뇨병 진단 경험 등), 의료이용 (미충족 의료 경험, 미충족 의료 이유 등), 사고 및 중독(낙상)
(낙상경험 및 건수, 사고·중독 경험 및 건수, 사고·중독 원인 등), 활동제한 및 삶의 질 (주관적 건강수준 등), 사회 물리적
환경 (지역사회 물리적 환경, 사회적 연결망 – 친척, 이웃, 친구, 사회활동 등), 개인위생 (식사 전·화장실 다녀온 후
외출 후 손씻기 실천 등), 교육 및 경제활동 (학력, 경제활동 참여, 혼인상태 등)

보건소에서 지역사회 건강조사도 담당하는 줄을 알게 되었고 지역사회건강조사를 어디서 주최하고 담당하는지
출처가 명확하지 않아 참여율이 저조함을 알게 되었다. 만약 대상에 선정될 시 성실히 응해야겠다고 생각했다.

5. 오늘 실습에서 좋았던 점과 아쉬웠던 점은 무엇인가?

(+) 지역사회건강조사란 무엇이고, 진행 방법과 목적에 대해 학습할 수 있었다.

(–) 이동진료팀인데 이동진료를 가지 못해 아쉬웠다.
오전에 자습을 하다보니 시간이 많이 안 가서 아쉬웠다.

CamScanner로 스캔하기

1. 오늘 실습경험의 의미 있는 점은 무엇인가?

오전에는 어제에 이어 지역사회건강조사 우편작업을 하였다.
건강증진실에 실습을 나갔다. 건강증진실에서 현재 진행하는 사업인 "모바일 헬스케어"의 참여 대상, 목적 등을 학습하고 접수절차를 확인할 수 있었다.
건강증진실의 역할을 학습할 수 있었다. 구강보건실에서 불소를 소분하고 포장하는 작업을 했다.

2. 오늘 실습을 하는 동안 나의 생각과 느낌은 어떠했는가?

거주지에 있는 건강증진실에 인바디를 하러 몇 번 가봤었는데 굉장히 비활성화 되어 있었다. 근데 군산시 보건소에서는 대표적으로 모바일 헬스케어가 인원 모집 중이고, 인바디 검사, 헬스장 운영을 하고 월·수·금은 운동수업, 화·목은 '한목하게 둘레길 걷기'라는 프로그램을 진행함을 알게 되었다. 지금까지 갔던 보건소 중에 가장 활발하다는 생각이 들었다. 6개월을 진행하게 되고, 활동 우수자에게는 활동량계를 지급하는 것 같은데 보상이 좋아 사람들의 흥미를 더 끈 것 같다.

3. 오늘 실습을 통해 새롭게 알게 된 것은?

. 모바일 헬스케어: 건강위험요인이 있는 사람에게 모바일 앱을 통해 보건소 전문가(의사, 코디네이터, 간호사, 영양사, 운동전문가)가 언제 어디서나 맞춤형 건강 상담을 제공하는 서비스.

· 대상자: 군산 거주(직장 포함), 만 19세 이상 성인, 건강위험요인 1개 이상 가지고 있는 사람 (질환자, 약물복용자 제외)

· 건강위험요인: BP 130mmHg↑, 85mmHg↑ / 공복혈당 100mg/dL↑ / 허리둘레 남 90cm↑, 여 85cm↑ / 중성지방 150mg/dL↑ / HDL 콜레스테롤 남 40mg/dL↓, 여 50mg/dL↓.

4. 오늘 실습을 통해 새롭게 배운 것들이 향후 간호실무에 어떻게 적용될 수 있겠는가?

· 나중에 보건소 건강증진실에서 근무할 경우, 건강을 향상시키기 위한 프로그램을 구상하고 진행할 수 있을 것이다.

. 건강위험요인을 벗어난 ~~[illegible]~~ 몸에 이상이 있다고 판단될 시 의료기관에 연계하고 추가검진의 필요성을
선영 할 수 있을 것이다.

5. 오늘 실습에서 좋았던 점과 아쉬웠던 점은 무엇인가?

(+) 건강증진실에서 진행하는 사업이 어떻게 진행되는지 관찰하고, 학습할 수 있었다.
영양, 금연, 뇌졸중 예방(근기 강화), 대사증후군 등 건강을 향상시키기 위한 내용을 학습할 수 있었다.
lipid test라는 혈액검사를 통해 지질 수치를 측정하는 모습을 관찰할 수 있었다.

(-) 다른 곳으로 출장 나가신 선생님은 어떤 내용으로 어디로 가신 건지 궁금했는데 알지 못해서 아쉬웠다.

CamScanner로 스캔하기

1. 오늘 실습경험의 의미 있는 점은 무엇인가? [접수실, 컨퍼런스]

오전 9시부터 오후 1시까지 컨퍼런스를 진행했다. 오전에 통증관리의 술기평가를 진행했다. 통증관리는 생리간호·기본간호 등 많이 배웠지만 술기평가로는 처음이라 좀 걱정이 많이 됐다. 이후 사례보고서를 간호진단까지 작성했다. 컨퍼런스 후 보건소 1층 접수실에서 민원인들의 안내를 도왔다.

2. 오늘 실습을 하는 동안 나의 생각과 느낌은 어떠했는가?

• 통증관리의 술기평가는 처음이라 걱정이 많이 됐는데 생각보다 수월하게 끝났다.
• 사례보고서 컨퍼런스를 진행했는데 사례보고서를 작성하면서 생겼던 물음표들이 컨퍼런스를 진행하며 많이 해소되었다. 적절하게 준비해오지 못해 아쉬웠지만 (오늘 잘 못한 만큼) 다음 컨퍼런스까지 준비해와야 겠다고 생각했다.
. 컨퍼런스 후, 접수실에서 민원인들의 안내를 도왔다. 접수실 자리에는 "민원 안내"라는 내용에 설명이 자세하게 적혀있었다. 여기서 한 눈에 보건소의 업무를 볼 수 있어 좋았다. 보건소에서 일하도 했던 적이 있어서 잘 안다고 생각했는데 군산시 보건소가 훨씬 크고, 새로운 내용도 많아 재미있었다.

3. 오늘 실습을 통해 새롭게 알게 된 것은?

보건소에서 가장 많이 하는 검사는 보건증 발급과 결핵검사로 오른쪽 뒤에 있는 결핵관리실과. 번호표 뽑고 접수를 해야 된다는 말을 가장 많이 한 것 같다. 보건소에서는 사무업무 외에도 민원인들을 대상으로 응대하고 프로그램 수행 및 검사를 진행하는데 그 항목에는 3층 건강관리과에서 암, 난임, 희귀난치, 영유아-건강증진 지원 관련 업무 / 3층 보건사업과 의약계: 병,의원 관련 신고 / 3층 전염병 관리계: 전염병, 방역관련 보건사업과 / 2층 정신건강실: 정신건강상담 / 2층 구강보건센터: 어린이 홈메우기, 어르신(저소득층 읍면동신청 대상자)·1.2.3급 장애인 진료 / 2층 한방진료실: 한방진료 / 2층 물리치료실 / 1층 치매상담센터 / 결핵관리실 / 자동차 적성검사 / 각종 검사 / 건강 클리닉 운영 / 금연 프로그램 / 예방접종 / 산후 도우미 등 다양한 일을 한다.

4. 오늘 실습을 통해 새롭게 배운 것들이 향후 간호실무에 어떻게 적용될 수 있겠는가?

민원인들을 응대할 때 정확하게 설명할 수 있을 것이다. 민원인으로 오게될 때, 보건소에서 할 수 있는 업무를 알고 할 수 있을 것이다. 보건소에서 진행하는 업무 중 몰라서 지원 대상자에도 혜택을 받지 못하는 경우가 많은 것 같다. 그래서 만약 일하게 된다면 사업의 대상자들이 주로 보는 곳을 분석하고 홍보할 것이다.
외상이 두창 감염 예방을 위해 손등·손바닥에 피부질환이 있는지 확인할 수 있을 것이다.

5. 오늘 실습에서 좋았던 점과 아쉬웠던 점은 무엇인가?

(+) 사례보고서에 대해 막막했는데 교수님께서 해주시는 피드백을 듣고 감을 조금이나마 잡을 수 있었다. 보건소에서 하는 업무들을 어떻게 생각하고 진행하는 지 흐름을 볼 수 있게 되었다. 다양한 업무를 한 눈에 볼 수 있었다. 접수실 선생님께서 친절하게 설명을 해주셨다.
(-) 사례보고서를 작성하는 방향이 부족했던 것 같아 아쉬움이 남는다. 접수실을 오전부터 나서 민원 안내에 대한 내용을 미리 읽어보고 접수시간에 위치를 간단하게 나마 파악했다면 응대할 때 좀 더 쉽게 설명할 수 있었을 것 같다.

CamScanner로 스캔하기

1. 오늘 실습경험의 의미 있는 점은 무엇인가? [구강보건센터]

실습 2주차 첫 날이고 구강보건센터에서 실습을 진행했다. 호원대학교 치위생과 교수님께서 오셔서 행사에 필요한 물품을 지원받으셨고, 미군부대에서 군산 시장님께 표창장을 수여받으러 오셨다가 보건소도 방문하셨다. 치과의사 선생님께서 즉석에서 영어로 통역 및 설명하시는 모습을 볼 수 있었다. 보건소 직원분들이 편하게 오셔서 치아 상담을 하셨다.

의사소통

2. 오늘 실습을 하는 동안 나의 생각과 느낌은 어떠했는가?

구강보건센터에서 불소용액을 만들어 소분하는 일을 도왔었는데 오늘 정식으로 출근하여 실습하게 되었다. 구강보건센터에서 하는 일에 대해 전반적으로 알게 되었고, 지역주민분들께 불소용액을 나누어주는 일을 하였다. 생각보다 많은 분들이 알고 찾아오셔서 복지혜택 등을 누리는 것을 보고 나도 지역의 보건소 홈페이지를 둘러보고 해당되는 프로그램이 있다면 참여해보도록 해야겠다는 생각이 들었다.

미군부대에서 몇 분이 오셔서 영어로 구강보건센터에 대해 설명하는 모습을 관찰하였는데 처음에 다짜고짜 우르르 들어오셔서 당황했지만 취약계층이 무료로 누린다는 말에 감탄을 하는 모습을 보고 내가 한 일은 아니지만 뿌듯했다.

3. 오늘 실습을 통해 새롭게 알게 된 것은?

• 구강보건센터에서 하는 일 : 노인의치보철 사업, 장애인 및 취약계층 무료진료, 학교구강보건사업, 어린이 충치 예방사업, 불소용액 양치 사업, 구강보건 교실 사업, 구강건강관리 사업 등.

⇒ 주로 "예방 + 관리"에 중점을 두고 있음.

• 구강 건강 생활수칙
① 하루 2번 이상 칫솔질 하기
② 불소치약 이용하기
③ 단 음식 ↓, 담배 피우지 않기
④ 정기적으로 치과 이용하기

4. 오늘 실습을 통해 새롭게 배운 것들이 향후 간호실무에 어떻게 적용될 수 있겠는가?

구강보건센터에서 하는 일을 알고 설명할 수 있을 것이다.

구강보건센터에서 하는 일의 대상자를 알고 설명할 수 있을 것이다.

구강 건강 생활수칙 4가지를 알고 설명할 수 있을 것이다.

칫솔질의 중요성, 치실 사용 방법 및 중요성, 칫솔 관리방법, 불소가 들어있는 치약 확인방법, 불소를 사용해야 하는 이유, 구강건강에 해로운 식습관, 유아 구강검진의 중요성에 대해 알고 설명할 수 있을 것이다.

5. 오늘 실습에서 좋았던 점과 아쉬웠던 점은 무엇인가?

(+) 구강보건센터에서 불소만 만들고 소분하여 어떤 일을 하는지 잘 몰랐는데 선생님께서 친절하게 설명해주셔서 어떤 일을 수행하고 어떻게 관리하는지 알게되었다.
미군부대분들을 보고 치과의사 선생님께서 즉석으로 통역을 하시는 모습을 볼 수 있어서 보건소에서 다양한 활동을 하는구나 싶었고 좋은 일 있었다는 생각을 했다.

(−) 우리가 하는 일이 많지 않아 조금 지루했다.

1. 오늘 실습경험의 의미 있는 점은 무엇인가? [국민건강보험공단, 순회지도]

국민건강보험공단에 출근하여 실습을 진행하였다. 국가 암검진에는 6가지가 있으며 암검진 독려 안내 전화를 하였다. (약 200명) 국민건강보험공단 채용 공고에 대한 설명을 해주셨다. 국민건강보험공단을 포함한 공기업에는 정량평가가 진행되고 학생 때부터 준비할 수 있는 직업교육, 자격증 등에 대해 학습할 수 있었다.

2. 오늘 실습을 하는 동안 나의 생각과 느낌은 어떠했는가?

국민건강보험공단에서 자궁경부암 검진 대상자에게 전화로 검진을 독려하는 안내 전화를 했는데 생각보다 많은 분들이 안 받으셨지만, 받으시는 분들은 친절하게 답해주시고, 또 '감사합니다' '좋은 하루 되세요.' 라며 인사를 해주셔서 나도 다음에 이런 전화가 오면 감사인사를 더 신경써서 해야겠다고 생각했다. 그리고 건강보험공단 채용 공고를 프린트 해주시고 필요한 항목에 대해 자세하게 설명을 해주셔서 원래 공기업에 관심이 없었지만 공기업 취업에 대해서도 관심이 생겼다.

3. 오늘 실습을 통해 새롭게 알게 된 것은?

국가 6대 암 검진: 대장암, 자궁경부암, 유방암, 간암, 위암, 폐암.
대장암: 1년 주기, 50세 이상 대상자 / 간암: 6개월 주기, 40세 이상 · 고위험군.
유방암: 2년 주기, 40세 이상 여성 / 자궁경부암: 2년주기, 20세 이상 여성.
위암: 2년 주기, 40세 이상 / 폐암: 2년 주기, 54~74세 폐암발생 고위험군.
- 자궁경부암 검진 시 주의사항: 임신 중인 경우 가능은 하지만 검진을 미룰 수 있음.
자궁적출술은 검진 제외가 가능, 성경험이 없는 경우 검진 제외, 검진 후 한 달 정도 병원에서 공단에 신고까지 시간이 걸림.

4. 오늘 실습을 통해 새롭게 배운 것들이 향후 간호실무에 어떻게 적용될 수 있겠는가?

대상자들에게 건강 검진의 중요성과 검진 주기를 설명할 수 있을 것이다.
건강 검진 시 주의사항을 설명할 수 있을 것이다.
국민건강보험공단과 같은 공기업 취업 시 준비해야 할 서류를 준비할 수 있을 것이다.
건강검진이 가능한 병원을 안내할 수 있을 것이다. (국민건강보험공단 홈페이지)
[illegible] 직업교육은 인터넷을 통해 수료할 수 있는 것을 알았고, 정량평가라 미리 해놓을 수록 좋으며 본인에게 혜택이 있는 것을 알아 적절히 적용할 수 있을 것이다.

5. 오늘 실습에서 좋았던 점과 아쉬웠던 점은 무엇인가?

(+) 선생님께서 친절하게 알려주셔서 좋았다. 국가 건강검진에 대해 자세히 알 수 있어 좋았다. 채용공고에 대해 하나하나 설명해주셔서 좋았다. 독려 전화 시 친절하게 응대해주셔서 좋았다. 나도 그런 전화가 오면 친절하게 답변해야겠다 생각했다.
(-) 하루밖에 국민건강보험공단을 가지 못해서 공단의 분위기라던가 하는 일을 세세하게 알아보기 어려웠다.

CamScanner로 스캔하기

1. 오늘 실습경험의 의미 있는 점은 무엇인가? [예방접종실]

예방접종실에서 실습을 했다. 예방접종실의 주 타깃층은 영유아, 산모 or 임신 준비 중 여성, 노인 등이 있고 이들을 대상으로 결핵(BCG)예방접종, B형간염 예방접종, 사람유두종 바이러스(HPV) 예방접종, 폐렴구균 예방접종 등을 진행하며 자궁경부암 검진표 등을 통해 여성 건강에 힘을 쓰는 모습을 관찰할 수 있었다.
[예진표 작성 → 예진 의사 상담 → 예방접종] 하는 모습을 관찰할 수 있었다.
⊕ tdap, A형간염, 유행성 출혈열.

2. 오늘 실습을 하는 동안 나의 생각과 느낌은 어떠했는가?

예방접종실에 총 성인 12명, 아이 3명이 예방접종을 맞으러 왔었다. 지금까지 중에 가장 많은 민원인을 대했던 것 같다. 예방접종 외에도 예방접종 증명서, 병원에 백신공급, 예방접종 후 교육 등 다양한 활동을 하는 모습을 볼 수 있었다. 계속해서 민원이 오지는 않았지만 무슨 일을 하려고만 하면 계속 와서 묘하게 바쁘다는 느낌을 받았다.
투석을 받는 분이 왔는데 투석을 받는 경우 B형 간염 4회까지 맞을 수도 있다는 것을 알게 되어 신기했고 폐렴구균에 2가지 종류가 있으며 2가지를 다 맞아야 효과가 좋다는 사실을 알게 되었다. 보건소에 있더라도 간호사가 지식을 가져야 함은 변치 않는다는 걸 다시 깨달았다.

3. 오늘 실습을 통해 새롭게 알게 된 것은?

· 투석을 받는 환자의 경우 대상자에 따라 B형간염 백신을 4회까지 접종할 수 있다는 것을 알았다.

· 임신 중 주의해야 하는 의약품: 여드름 치료제(이소트레티노인), 경련 치료제(발프로산), 고혈압 치료제(안지오텐신 수용체 차단제, 안지오텐신 전환효소 저해제), 항생제(테트라사이클린), 비스테로이드성 소염진통제(NSAIDs) 등.

· 임신 초기 혈당이 높은 경우 태아 기형 발생 확률이 증가하므로, 임신 전 혈당관리가 중요하다. 필요시 고용량의 엽산제를 복용하는 것도 권장된다.

4. 오늘 실습을 통해 새롭게 배운 것들이 향후 간호실무에 어떻게 적용될 수 있겠는가?

· 대상자가 폐렴구균 무료 예방접종 대상자인지 확인하고 설명할 수 있을 것이다. (65세이상, 58년생 이전 출생자)
· 임신 중에 약물을 조심해야 함을 대상자에게 설명할 수 있을 것이다.
· 예방접종 받기 전 주의사항을 알고 설명할 수 있을 것이다.
· 예방접종 받은 후 주의사항을 알고 설명할 수 있을 것이다.

5. 오늘 실습에서 좋았던 점과 아쉬웠던 점은 무엇인가?

좋았던 점: 예방접종실에서 수행하는 일을 알고, 예방접종을 받는 과정을 배울 수 있었다. 아이뿐이 아닌 어르신들도 예방접종을 받고 내가 해당되지는 않더라도 많은 예방접종을 관리함을 알 수 있었다.

아쉬웠던 점: 일을 안 하면 계속 와서 편안하게 앉지 [illegible] 서있었다.

CamScanner로 스캔하기

1. 오늘 실습경험의 의미 있는 점은 무엇인가? [치매분소], 컨퍼런스

치매분소에서 실습을 하게 되었고, 치매분소는 처음이라 OT를 해주셨다. 치매분소는 치매안심센터보다 작은 규모로 운영되는 데 생각보다 많은 인원이 오시는 것을 알 수 있었다. 치매분소는 의료기관이 아니므로 치매를 확진할 수 없고 의심소견이 있다면 의료기관에 연계하는 역할을 함을 알게 되었다. CIST 종이를 번호로 접는 일을 하였다.

2. 오늘 실습을 하는 동안 나의 생각과 느낌은 어떠했는가?

이전에 경로당으로 봉사활동을 나갔을 때 CIST (인지선별검사)로 검사하니 어려워하시는 어르신들을 도와 검사를 도운 경험이 있는데 실습에 나와 다시 보게되어 반가웠다.
60세 이상을 대상으로 CIST로 1차 검사를 하고 이 검사결과 상 인지 저하가 나타날 경우 정밀검사 (SNSB)를 수행함을 알았다. 검사하는 곳과 분리되어 있었지만 검사 내용이 들렸는데 내가 지금 하더라도 약간 헷갈릴 것 같은 내용이 있어서 나도 경각심을 가져야겠다는 생각을 했다. 그리고 오신 분들이 "인지 저하"로 거의 없으셔서 좀 다행이었다.

3. 오늘 실습을 통해 새롭게 알게 된 것은?

조기검진 대상 : 60세 이상 관내 어르신

1. 인지선별검사 (CIST) : 학력, 연세에 따른 기준점수에 의하여 <정상/인지저하>로 나누어지고 인지저하 시, 정밀검사 안내 및 연계.

2. 정밀검사 (SNSB) : 인지선별검사 실시 결과 <인지저하>가 나오시는 분들을 대상으로 실시
* 기억력, 지남력, 주의력, 시공간기능, 집행기능, 언어기능 등의 영역 검사를 문답식으로 시행
⇒ 결과 : 정상인지 장애 (1년 마다 재검사), 치매의심 (감별검사 연계)

3. 감별검사 : 병원(신경과)에서 실시하는 치매 진단을 위해 MRI 또는 혈액검사
* 중위소득 120% 이하의 대상자에게 감별검사비 지원.

4. 오늘 실습을 통해 새롭게 배운 것들이 향후 간호실무에 어떻게 적용될 수 있겠는가?

· 치매 관련 프로그램에는 치매환자 쉼터, 치매예방 교실, 인지 강화 교실, 치매 가족 지원 교실 등이 있으며 어르신들에게 설명할 수 있을 것이다.
. 치매 환자 등록 시 : 치매치료비 지원 서비스, 조호물품 지원 서비스, 사례관리 서비스, 실종예방 서비스가 있음을 설명하고 연계할 수 있을 것이다.
- 실종예방 서비스 중에서 치매환자를 대상으로 지문등록, 배회인식표 배부, 배회감지기 배부를 함을 알고 설명하여 연계할 수 있을 것이다.

5. 오늘 실습에서 좋았던 점과 아쉬웠던 점은 무엇인가?

(+) 정신실습 때도 치매안심센터 위에 있는 정신건강복지센터에서 실습을 하여 치매안심센터에 대해 궁금했어 있었는데 오늘 선생님께 설명을 들으며 궁금증을 해소할 수 있어서 좋았다.

(-) 검사를 하는 방과 분리되어 있어 소리로만 검사방법을 유추해야해서 약간 아쉬웠다.

CamScanner로 스캔하기

1. 오늘 실습경험의 의미 있는 점은 무엇인가?

OT 당시 국민 건강 보험공단, 감염병 대응과, 치매 관리과, 군산시 보건소에 대해 다양한 OT와 설명을 들을 수 있었는데. 그게 정말 좋은 정보와, 도움이 되었다고 생각이 들어서 가장 의미 있었던 활동이었다.

2. 오늘 실습을 하는 동안 나의 생각과 느낌은 어떠했는가?

보건소에서 X-ray 같은 간단한 처치만 진행하는 줄 알았는데 생각보다. 지역 사회에서 다양한 활동을 진행하고 있다는 것을 알게 되었다. 정신 실습 때. 세종시 보건소에서 했던 것과 다르게 실제로 방문을 한다던가, 어떤 과에서는 어떤 업무를 하는지에 대해 자세하게 알게 되어서 좋은 실습이었던 것 같다.

3. 오늘 실습을 통해 새롭게 알게 된 것은?

방문건강 관리 사업이란 보건소에 소속된 방문 간호사가 각 가정을 방문하여 가족과 건강문제를 가진 가구원을 발견하여 질병 예방 및 관리, 건강증진을 위하여 건강서비스를 제공하는 것이다. 방문 간호 대상은 기초 생활 수급자, 독거노인, 차상위 계층, 장애인, 독거노인, 타기관에서 의뢰한 건강문제가 있는 대상자, 재가 암관리, 재가 장애인 등이 대상이다.

4. 오늘 실습을 통해 새롭게 배운 것들이 향후 간호실무에 어떻게 적용될 수 있겠는가?

방문간호사들이 어떤식으로 근무를 하는지, 어떤 일을하고, 대상자들은 누구이며, 어떤 사업들을 실시 하는지 에 대해 배웠는데, 후에 방문 간호사가 되었을 때 지식으로 사용할 수 있을 것이라는 생각이 들고, 방문간호사가 되어서 어떻게 해야 하는지에 대해 ~~[illegible]~~ 더 자세하게 알게 되면, 더 나은 간호를 제공할수 있을것이다.

진로 선택?

5. 오늘 실습에서 좋았던 점과 아쉬웠던 점은 무엇인가?

처음나간 실습에서 실제 방문 간호를 따라가서 자세하게 보고 배울수 있어서 좋았다. 다양한 과를 전체적으로 체험하고, 실습하며 다양한 대상자를 만나게 된것도 좋았던 점 같았다. 아쉬웠던 점은 없었다.

CamScanner로 스캔하기

1. 오늘 실습경험의 의미 있는 점은 무엇인가?

방문 간호를 위해 대상자들의 집이나 경로당에 방문하여 정서적 지지를 제공하고 활력 징후를 측정하거나 암 환자의 경우 영양제를 2달에 한 번씩 제공하며 이야기를 듣고, 필요한 간호를 제공한 점이 의미 있었다.

2. 오늘 실습을 하는 동안 나의 생각과 느낌은 어떠했는가?

방문 간호라는 말에 솔직히 만성적인 환자가 많아 제공해야 한다거나, 상태가 많이 좋지 못한 대상자가 많으면 어떡하지라는 걱정이 많았는데, 생각보다 정정하신 분들이 많았고, 요새는 제도의 개편으로 요양등급이 나온 대상자는 요양시설에서, 다른 등급을 받으면 또 다른 기관에서 관리가 되고 있어서 중증도가 높은 환자를 간호하지는 않는다고, 말씀하셨다.

3. 오늘 실습을 통해 새롭게 알게 된 것은?

방문건강관리의 목표는 건강 행태를 개선하는 것과 건강문제를 관리하는 것이다. 건강행태 관리 개선을 위해 건강상태를 인식하고 건강생활 실천을 유도하며, 건강지식을 향상시키는 목표를 지향한다. 또한 건강문제 관리를 위해 건강문제 스크리닝을 정기적으로 실시하며 증상을 조절하고, 치료 순응도 향상시키기 위해 다양한 활동을 하고 있음을 알게 되었다.

4. 오늘 실습을 통해 새롭게 배운 것들이 향후 간호실무에 어떻게 적용될 수 있겠는가?

건강을 주제로 대상자들과 이야기를 하다보면 잘못된 의료지식을 사실인 것처럼 생각하시는 분들도 계셨고, 건강에 대해 깊은 관심이 없으신 분들도 계셨는데, 그런 대상자 모두와 건강을 주제로 2달에 한 번씩 이야기를 나누면서 경각심을 일깨운다면 대상자들은 작든 크든 나름대로의 행동을 실천할 것이기에 자주 이야기 할 것이다.

의사소통
교육자

5. 오늘 실습에서 좋았던 점과 아쉬웠던 점은 무엇인가?

방문간호를 하면서 환자들에게 건강에 대해 인식을 심어주고, 관리를 위한 교육을 진행하면서 조금 더 대상자들에게 경각심을 일깨워 준 것 같아 좋았던 것 같다.
아쉬웠던 점은 특별하게 없었던 것 같다.

CamScanner로 스캔하기

1. 오늘 실습경험의 의미 있는 점은 무엇인가?

혈압과 혈당검사를 해드리면서 정상범위나, 검사의 목적에 대해 질문하시는 분들에게 쉽게 설명을 드리려고 설탕물에 비유한다거나, 호스관에 대해 비유하는 방법으로 설명을 드렸더니 대상자 분들께서 '공부 많이 했네, 이해가 잘 돼'라고 말씀해주셔서 정말 기뻤고, 의미 있었던 것 같다.

2. 오늘 실습을 하는 동안 나의 생각과 느낌은 어떠했는가?

생각보다 연세가 있으신 대상자 분들의 기초 건강검진 결과 평균혈압이 높아서 놀랐다. 여태까지는 연세가 있으셔도 혈압이 평균정도였는데, 외부지역으로 나오니까 관리를 하지만 입원은 하지 않는 대상자가 많아서 신기하기도 했던 것 같다. 대상자 분들은 보건소에서 방문하며 관리를 하는 것에 만족해하시는 것 같았다. 검사가 끝나면 '감사합니다'라고 이야기 해주시는데 그게 참 기쁘고 좋았다.

보건소역할

3. 오늘 실습을 통해 새롭게 알게 된 것은?

예방활동.

군산시 보건소의 이동진료팀은 의료취약지를 찾아가는 의료서비스를 실시하는데, 이는 3월부터 12월까지 실시하며 개별적으로 가정에 방문해서 실시하는 것이 아니라, 의료취약지의 경로당에서 실시하며 어르신들을 상대로 기초건강검진과 보건교육을 실시한다. 한방진료도 보는데 아직 본적은 없었고, 생각보다 많은 어르신들이 혈압과 혈당을 재기 위해 방문하신다는 것을 알게되었다. 중간에 환자분들께서 건강에 대해 질문을 하시면 그것도 알려드리거나 병원에 방문해보실 수 있도록 격려하는 역할도 해서 병원과 환자를 연결하는 징검다리 같은 역할을 한다.

4. 오늘 실습을 통해 새롭게 배운 것들이 향후 간호실무에 어떻게 적용될 수 있겠는가?

생각보다 많은 분들께서 기초 건강검진을 받기 위해 경로당에 방문하고 계셨는데, 왜 혈압과 혈당이 중요한지에 대해서는 모르시는 분들이 많았다. 어르신들이 알아듣기 쉽게 설명해드리기 위해 고민하고 실제로 적용해봄으로써 대상자에게 최선의 간호를 제공하는 간호 실무에 더 이해시키기 쉽도록 접근할 수 있을 것이다.

5. 오늘 실습에서 좋았던 점과 아쉬웠던 점은 무엇인가?

의사소통.
맞춤형.

혈압의 중요성에 대해 묻는 대상자에게 최대한 쉽게 설명을 드리게 되었는데, 선생님께서 답변을 듣고는 알아듣기 쉽게 설명을 잘하는 것 같다고 칭찬해주셨다. 선생님께 칭찬을 받은 것도 기뻤지만 환자가 내 답변을 듣고 쉽게 이해를 하셨다는게 더 기뻤던 것 같다. 아쉬웠던 점은 없었다.

CamScanner로 스캔하기

P21

1. 오늘 실습경험의 의미 있는 점은 무엇인가?

오늘은 건강증진팀을 방문해서 실습을 진행 했는데 이런 세부적인 팀에서 하는 업무를 다양하게 볼 수 있어서 좋았다. 환자 분들께서 운동 요일을 궁금해 하시는 경우가 많은데 운동요일을 제대로 숙지해드리거나 지질혈증 검사를 해볼수 있어서 좋았다.

2. 오늘 실습을 하는 동안 나의 생각과 느낌은 어떠했는가?

건강증진팀은 대부분이 간호사일거라고 생각 했는데 운동 선생님이나 영양사, 간호사 등으로 다양하게 이루어져 있어서 다학제간으로 환자 간호를 실시할 수 있는 것 같아서 흥미로웠다. 기본적인 걷기부터 운동 선생님께서 자세를 잡아주신다거나, 영양 상태를 돕는 등의 활동을 한 팀에서 진행할 수 있어 효율적이라는 생각이 들었다.

협력

3. 오늘 실습을 통해 새롭게 알게 된 것은?

건강 증진 팀에서는 간단한 건강검진을 실시하는데, 혈당, 혈압 뿐만아니라 지질혈증 관련 검사를 실시한다 모바일 헬스케어 앱을 통해 예약을 하면 인바디 검사부터 실시할 수 있는데 그렇게 3~6개월마다 검진을 하는 사업을 하고, 헬스장이 붙어 있어서. 월. 수. 금은 운동선생님과 운동을 진행하고, 화. 목은 월명산을 걷거나 필요하신 분들은 헬스장을 이용하는 방법으로 진행되고 있었다.

4. 오늘 실습을 통해 새롭게 배운 것들이 향후 간호실무에 어떻게 적용될 수 있겠는가?

생각보다 보건소에서는 다양한 방법으로 대상자나 고위험 환자들을 관리했는데, 수치가 너무 위험한 환자들은 병원에서 관리가 되어야 하기 때문에, 만약 수치가 너무 높다는 판단이 되면 병원으로 갈 수 있도록 독려할 필요가 있다. 만약 꾸준한 관리로만 관리할 필요가 있는 사람이라면 보건소에서 꾸준하게 관리할 수 있도록 독려한다.

예방활동

5. 오늘 실습에서 좋았던 점과 아쉬웠던 점은 무엇인가?

건강 증진 팀을 방문해서 보건소에서 진행하는 다양한 활동에 대해 알 수 있어서 좋았다. 인바디라던가 헬스장이 있을 거라고는 생각을 못했는데, 지역사회에서의 보건소의 역할이 다양해지는 것 같아 재미있었고 흥미로웠다. 아쉬운 점은 비가와서 월명산 걷기운동에 참여할 수 없었다는 점이었다.

CamScanner로 스캔하기

1. 오늘 실습경험의 의미 있는 점은 무엇인가?

오늘은 치매안심센터에서 선생님들이 하시는 업무를 볼수 있었다. 확인해보니. 오늘은 정밀검진(SNSB)가 있는 날이라서 더 신기한 장면을 볼수 있었다. 대상자분들이 불편해 하시는 않으실까 걱정 됐는데 선생님들께서 학생 선생님이니까 불편해 하지 않으셔도 된다고 이야기 해주셔서 기뻤다.

2. 오늘 실습을 하는 동안 나의 생각과 느낌은 어떠했는가?

치매안심센터에서 아르바이트를 했었을 때에는 75세 이상의 어르신만을 대상으로 기억력 검사 전화를 드렸는데, 군산시에서는 60세 이상이 대상이라고 말씀하셔서 신기했다. / 요새는 노인분들께서도 (CIST, SNSB) 같은 검사 방법을 다 알고 오셔서 관악에서 새로 기억력 검사 조사표를 다시 만들었다는 것을 듣고 신기했다.

3. 오늘 실습을 통해 새롭게 알게 된 것은?

치매안심센터에서는 60세 이상의 관내 어르신들을 대상으로 인지선별검사를 실시하는데, 학년, 연세에 따른 기준점수에 의해 정상 / 인지저하로 나뉘어 진다. 만약 이 단계에서 인지저하로 나타나면 정밀검사 단계로 넘어가서 기억력, 지남력, 주의력, 시공간기능, 집행기능, 언어기능 등으로 나누어 검사를 진행하고 결과가 경도인지 장애가 나오면 1년마다 재검사를, 치매가 의심된다면 감별검사로 연계한다.

4. 오늘 실습을 통해 새롭게 배운 것들이 향후 간호실무에 어떻게 적용될 수 있겠는가?

예방중심.

치매는 미리 예방하는 것도 중요하지만, 조기 진단을 하는 것도 만만치 않게 중요하다. 평소에 기억력에 이상이 있다고 느낀다면 지체없이 검사를 받아볼 필요가 있고, 보건소나 치매안심센터에서 상당히 전문적으로 검사를 진행하고 있기 때문에 이런 내용을 환자들에게 전달하면 좋은 것 같다.

5. 오늘 실습에서 좋았던 점과 아쉬웠던 점은 무엇인가?

겨울 방학때 ~~[illegible]~~ 관공서 아르바이트로 치매 안심센터에서 근무를 했는데, 이렇게 다른지역의 치매 안심센터로 오게 돼서 신기했다. 당시에는 환자들의 검사나 진찰같은 것은 어떻게 진행되는지 몰랐었지만, 오늘 치매안심센터에서는 자세하게 알려주셔서 유익한 것들이 많아 감사했다.

CamScanner로 스캔하기

1. 오늘 실습경험의 의미 있는 점은 무엇인가?

선생님을 도와서 불소용액을 만들고 병에 옮겨 담고, 박스에 담는 일을 도맡아 하게 됐다. 아이들 방문을 위해 어린이집에 불소용액을 넣고, 모래시계와, 팜플렛을 넣어 포장했는데 선생님들께서 너무 고맙다고 이야기 해주셨다.

2. 오늘 실습을 하는 동안 나의 생각과 느낌은 어떠했는가?

불소용액은 사서 나누어 주는 것인줄 알았는데, 가루에다가 물같은 액체를 특정비율로 섞고 기계로 돌리면 불소용액이 만들어 지는 것이었다. 신기했고, 생각보다 불소용액을 받기 위해서 다양한 분들이 방문하고 계셔서 불소 나눔사업이 중요하겠구나 하는 생각이 들었다.

3. 오늘 실습을 통해 새롭게 알게 된 것은?

구강 보건센터는 기존 구강 보건실 (보건소내)을 확대, 전환 하여 인력, 물적 자원을 강화함으로써 보건지소와 보건소로 분산되어있는 진료체계를 구강보건센터 중심의 조직적이고 효율적인 구강보건진료 사업구조로 변화시켜 취약계층의 구강진료 및 예방활동 접근성을 높는 곳이다. 하지만 일반 치과처럼 진료는 하지 않고 대부분은 불소 해주나. 예방사업이 주인것 같았다.

4. 오늘 실습을 통해 새롭게 배운 것들이 향후 간호실무에 어떻게 적용될 수 있겠는가?

실습도중 미군부대에서 중사, 소령정도 되시는 분들께서 보건소에 방문하셨는데 한국의 구강 보건센터에서 진행하는 사업에 대해서 여쭙고, 모든 사업들이 대부분 무료라는 것이 미국도 그런제도가 있다면 좋겠다고 말씀하셨다. 한국에는 정말 많은 사업으로 다양한 활동을 하는데, 그 사업들이 조금 더 대중화 될수 있도록 노력할 필요가 있어보였다. 보건소 인식

5. 오늘 실습에서 좋았던 점과 아쉬웠던 점은 무엇인가?

구강 보건센터에서 하는 일을 자세하게 볼수 있어서 신기하고 좋았고, 불소용액을 만들고 포장하면서 뿌듯함도 느낄수 있었던 것같다. 아쉬웠던 점은 아직 불소용액이나, 구강보건센터에서 하는 일에 대해 모르시는 분들이 많은 것 같아 아쉬웠던 것 같다.

CamScanner로 스캔하기

1. 오늘 실습경험의 의미 있는 점은 무엇인가?

처음으로 보건소가 아닌 공기업으로 실습을 나가게 됐다. 국민건강보험공단에서 실습을 했는데. 공기업으로 실습을 나가서, 설명을 듣고, 실습을 진행하면서 다양한 진로 분야로 눈을 뜰수 있게 돼서 정말 좋았다고 생각한다. 학생들에게 간호학과의 진로가 간호사, 병원만 있는것이 아니라고 알려주면 좋을 것 같다.

2. 오늘 실습을 하는 동안 나의 생각과 느낌은 어떠했는가?

공기업인 건강보험 공단에서, 검진 독려 전화와 함께. 검진 가능한 병원까지 알려주는 등의 관리 방법을 실시하는 것이 대단했다. 한국의 건강보험에 대해. 외국에서도 좋은제도라고 말하면서 자국으로 배워가길 원한다고 OT때 들었었는데 실제 진행되고 있는 것을 보니 더 대단하고 국민에게 위한 제도라고 생각했다.

3. 오늘 실습을 통해 새롭게 알게 된 것은?

자궁경부암과 유방암검진은 2년주기로 검사를 받는다. 유방암 검진은 40세 이상, 자궁경부암의 경우 20세 이상 여성이 대상이다. 자궁경부암의 경우. 최소 검사 48시간 전부터는 질 내 어떠한 조작도 넣지 않아야 하며. 성관계, 탐폰, 질세척, 질내 약물 & 윤활제, 질내 피임약 등이 다 금지된다. 2년마다 자궁경부 세포검사를 진행하며 특이사항이 있는경우 의사와 상담한다.

4. 오늘 실습을 통해 새롭게 배운 것들이 향후 간호실무에 어떻게 적용될 수 있겠는가?

국민건강보험공단은 공기업으로서 국민의 건강 관리에 대한 기초적인 검사를 잘 담당한다. 대상자들에게 당연히 누릴수 있는 권리에 대해 설명드리고, 기초적이지만 중요한 암 검진에 대해 진행할 수 있도록 도우면서. 국민의 건강 향상에 이바지 할 수 있을 것이다.

5. 오늘 실습에서 좋았던 점과 아쉬웠던 점은 무엇인가?

자궁경부암 검진에 대해 자세하게 알게 되었고, 대상자들에게 전화로 전달하는 과정에 있어서 다양한 사례를 접할 수 있어 좋았다. 다만 아쉬웠던 점은 다양한 분들께서 건강보험공단의 전화를 수신 거절 했을때 대처에 대해 몰라서, 아쉬웠던 것 같다.

CamScanner로 스캔하기

1. 오늘 실습경험의 의미 있는 점은 무엇인가?

건강검진시 대상항 검사를 위해 분변통을 만드는 일을 했다. 아우래로 대장암 검진이라고 적힌 부분의 종이가 드러나는것이 대상자들의 참여를 용이하게 할것같아 해당 방법으로 만들었는데 선생님께서 훨씬 좋다고 해주셨다.

2. 오늘 실습을 하는 동안 나의 생각과 느낌은 어떠했는가?

2일간 건강보험공단에서 실습하면서 다양한 민원업무도 보고, 사증경우맞추려 전화도 하고, 분변통도 만들고 하면서 생각보다 건강보험공단이 다양한 일을 한다는 것을 알게됐다. 간호사로서의 길은 임상 밖에 없다고 생각한 나에게 견문과 시각이 넓어지게 된 계기가 되어 정말 좋았다.

3. 오늘 실습을 통해 새롭게 알게 된 것은?

건강보험공단에서는 모바일 앱으로 건강관리가 가능하도록 서비스를 만들었는데. 그중 대사증후군의 건강관리를 위해서. 최근 5년간 국가 건강검진을 받은 사람 중 누구나 대사증후군 24주 건강 관리를 받을 수 있다. 모바일 앱에서는 식단 관리를 할수 있는데. 이는 대사증후군의 예방이 복부비만 해소와 칼로리 섭취 감소와 연관성이 있기 때문이다.

4. 오늘 실습을 통해 새롭게 배운 것들이 향후 간호실무에 어떻게 적용될 수 있겠는가?

실습도중 민원실이 어딘 사무실로 민원을 넣으러 오신 분이 계셨는데, 우리가 있는 실습지는 사무실이라서 대상자들의 개인정보가 유출될 우려가 있어 민원인이 들어오지 못하도록 막았다가, 휴게실로 안내해 대상자들의 고충을 자세히 듣는 선생님의 모습을 볼수 있었다. 무턱대고 막는 것이 아니라 대상자의 입장을 충분이해하는 자세가 필요하다고 생각했다.

5. 오늘 실습에서 좋았던 점과 아쉬웠던 점은 무엇인가?

분변통을 제작하고, 선생님과 공단의 비전, 그리고, 입사하는 방법과 간호사의 다양한 진로를 이야기 할수 있어 좋았다. 특히 선생님께서 간호사셔서 더 이해도 잘되고, 설명도 잘해주신 것 같았다. 아쉬웠던 점은 없었다.

CamScanner로 스캔하기

1. 오늘 실습경험의 의미 있는 점은 무엇인가?

예방접종실에서 하는 어린이 접종은 관리번호 발급 대상에게만 실시한다. 그 말인 즉슨, 주민번호가 없는 내국인이나, 외국인 등록번호가 없는 장기 체류 외국인이라는 뜻이다. 실제 실습 당시 생각보다 많은 외국인들이 방문해서 신기했어서 찾아보니 모르는 어린이에게 하는 접종이 아니었어서 재미있었다.

2. 오늘 실습을 하는 동안 나의 생각과 느낌은 어떠했는가?

실습을 하면서 보건소의 예방접종실이 생각보다 체계가 잘잡혀 있고, 지역 병원과도 잘 연결이 되어있다는게 신기했다. 우리는 매우 이런 시스템 속에서 살아서 잘 몰랐지만, 타국의 잡혀있지 않는 의료체계를 기자로나, 공부로나 접하게 되면서 정말 많은 발전을 일구어 냈구나 하는 생각이 들어서 뿌듯했다.

3. 오늘 실습을 통해 새롭게 알게 된 것은?

국가에서는 1995년부터 예방접종 후 이상반응이 발생하면 예방접종과의 관련성을 심의해 진료비 등을 보상하는 '예방접종 피해 국가보상제도'를 운영하고 있다. 사람유두종바이러스 감염증 예방접종 후 이상반응이 발생하여 진료를 받은 경우 예방접종 피해 국가보상 신청이 가능하며, 보상 신청 방법 등 자세한 사항은 주소지 관할 보건소에 문의하거나 홈페이지에 방문하면 된다.

4. 오늘 실습을 통해 새롭게 배운 것들이 향후 간호실무에 어떻게 적용될 수 있겠는가?

보건소에서 실시하는 예방접종 사업에 대해 자세하게 알게 됐고, 각 접종들의 시기와, 접종 전, 후의 관리 방법들에 대해 배우면서. 실제 대상자들의 나이나. 관련 질병들에 대해 자세하게 알수 있게 되었고 보건소의 중심업무에 대해서도 자세하게 알게되었던것 같다.

5. 오늘 실습에서 좋았던 점과 아쉬웠던 점은 무엇인가?

예방접종실에서 생각보다 다양한 연령대의 사람들을 볼수 있어서 좋았다. 특히 영유아가 80%로 많았는데 아동간호학을 다시 배우는 것 같아 재미있었고, 생각보다 주사를 무서워하지 않는 아기도 많다는 것을 알게 됐다.

아쉬웠던 점은 책상이 너무 좁다..

CamScanner로 스캔하기

1. 오늘 실습경험의 의미 있는 점은 무엇인가?

접수실 대신 적성검사실에서 운전면허증 갱신, 신규와 관련된 업무를 했다. 면허증이 있지만 보건소에서 이렇게 신체 검진과 관련되어서 검사를 했었나 하는 생각이 들었는데 생각보다 재미있고, 알게 되는 것들도 많아서 더 의미있게 근무했던 것 같다.

2. 오늘 실습을 하는 동안 나의 생각과 느낌은 어떠했는가?

지난 열흘간 실습을 보건소에서 진행하면서 다양한 과를 경험했던 것 같다. 처음에는 계속 옮겨야 하고, 적응할 것 같으면 새로운 곳으로 가게 돼서 조금 힘들었는데 10일간 그만큼 다양한 과를 볼수 있어서 정말 좋았다고 생각한다. 간호학생으로서 조금더 미래에 대해 깊게 생각하게 되었던 계기가 된것 같아서 좋았다.

3. 오늘 실습을 통해 새롭게 알게 된 것은?

적성검사실에서는 자동차 운전 면허증을 갱신하거나 신규 발급해주는 곳이었는데, 그러다 보니 면허증별 적합 기준이라는 것이 있었다. 2종보통의 경우 양안 0.5 이상, 한쪽눈만 있는 경우 0.6 이상 이어야하며, 1종보통의 경우 시력 0.8 이상, 한쪽눈만 있는경우 0.8 이상, 수평시야 120°, 수직 시야 20° 이상이며 중심시야 20° 이내 암점 / 반맹이 없어야 한다.

4. 오늘 실습을 통해 새롭게 배운 것들이 향후 간호실무에 어떻게 적용될 수 있겠는가?

보건소에서는 생각보다 다양한 일을 한다. 분명 그건 주민과 국민의 건강 증진과 관련이 있는 일이겠지만, 나는 보건소에서 하는 일에 대해서 정확하게 알지 못했었다. 실제로 실습하면서 병원만큼 바쁘게 돌아가지 않는 보건소를 보면서 보건소가 하는 일에 대해 자세하게 알수 있다면 참 좋을 것 같다는 생각을 했다.

보건소 인식

5. 오늘 실습에서 좋았던 점과 아쉬웠던 점은 무엇인가?

접수실 인원이 두명이라서 솔직히 공간도 크지 않고. 많이 걱정했는데 선생님들께서 배려해 주셔서 적성검사실에서 근무를 할수 있게 되어 좋았다. 아쉬웠던 점은 없었다.

CamScanner로 스캔하기

P 22

1. 오늘 실습경험의 의미 있는 점은 무엇인가?

오늘 보건소 OT와 건강보험공단의 OT를 받게되었는데 중점을 두고 있는 사업에 대해 알게되었고 일부 업무에 참여하게되어 업무파악과 실습을 하는데 좋은 기회가 된것 같다. 보건소와 공단의 업무를 한눈에 파악이 가능하도록 설명해 주셔서 비교가 가능했고 치매와 진드기 등의 설명도 이해를 돕는데 도움이 되었다.

2. 오늘 실습을 하는 동안 나의 생각과 느낌은 어떠했는가?

간암 미검진 대상자들에게 독려전화를 돌리는 일을 하게되었는데 생각보다 일반 대상자들이 검진에 대한 인지가 높다는 생각이 들었다. 하지만 개인적인 일이나 업무 때문에 시간 내시기 어려워하신 분들이 많으셨다. 바쁘신 와중에도 시간을 내서 검진을 받으시는 분들을 보며 건강보험공단에서 건강검진에 대한 홍보가 잘 이루어졌고 대상자분들도 중요성을 잘 알고계시다는 것을 느끼게되었다.

3. 오늘 실습을 통해 새롭게 알게 된 것은?

국가 6대 암검진으로 1년주기 대장암 검진 (50세 이상 대상자), 2년주기 자궁경부암, 유방암 검진 (40세 이상 여성), 위암검진 (40세 이상 대상자), (20세이상 여성)
→ 40세 이상 간암발생 고위험군
고위험군 기준 해당 시 간암 6개월 주기 / 폐암 2년 주기 → 54~74세 폐암발생 고위험군 건강검진을 받을 수 있다. 간암은 간 초음파검사 + 혈청 알파태아 단백검사를 실시한다.

4. 오늘 실습을 통해 새롭게 배운 것들이 향후 간호실무에 어떻게 적용될 수 있겠는가?

암검진 주기와 검사항목, 고위험군에 해당하는 기준에 대해 배울 수 있었고 검진 독려를 수행할 수 있도록 해야겠다는 생각이 들었다. 1년에 2회 검진 받을 수 있는 간암 검진의 기간이 끝나감에도 아직 많은 분들이 받지 않으셨다는 것을 알게되었고 건강검진에 대해 잘 모르시는 분들이나 잊어버리고 계신 분들에게 정보를 알려드리고 독려하는 것이 중요하다는 것을 알게되었다.

5. 오늘 실습에서 좋았던 점과 아쉬웠던 점은 무엇인가?

독려전화를 하는 중에 깜박하셨다며 시간 맞춰서 예약하시겠다고 말씀하시는 분들이 계셨는데 내 연락으로 인해 제날짜에 검진을 받으실 수 있다는 것에 감사하기도하고 뿌듯한 마음이 들었다.

CamScanner로 스캔하기

[redacted]

#진로선택

1. 오늘 실습경험의 의미 있는 점은 무엇인가?

오늘 선생님께서 공단에 취업하기 위한 조건들과 경험담을 말씀해 주시는 시간이 있었는데 직접 공단에 취업해 계신 간호사 선생님의 생생한 경험담을 들을 수 있어 값진 시간이 였다. 인터넷에 돌아다니는 알수 없는 정보들보다 믿을 수 있는 정보를 들려주셨고 이곳에 취업할 수도 있겠구나 하는 새로운 길이 생긴것같다.

2. 오늘 실습을 하는 동안 나의 생각과 느낌은 어떠했는가?

3-2 #전문직

오늘은 건강검진을 받지 않으신 대상자 분들에게 전화를 걸어 독려하는 일을 했는데 대면이 아닌 전화통화를 꺼려하시는 분들도 계시고 이렇게 전화 걸어주셔서 감사하다는 분들도 계셨다. 전화실습을 통해서 대본의 중요성과 그때그때 하시는 질문에 답을 드리려면 그에 맞는 지식과 대처능력이 필요하다는 것을 느끼게 되었다.

3. 오늘 실습을 통해 새롭게 알게 된 것은?

• 자궁경부암 검진은 2년 주기(출생연도 짝·홀수 기준 적용)로 검진하고 자궁경부 세포검사를 시행한다. 이때 자궁적출술을 받았거나 성경험이 없으신 분은 검사 전에 반드시 검진의사와 상담해야한다.

• 유방암 검진은 2년주기(출생연도 짝 홀수 기준 적용)로 검진하고 유방 촬영을 시행한다. 유방 촬영술은 유방 질환의 진단에 있어 무증상 조기 유방암을 발견하는 가장 기본적인 방법으로 유방 압박으로 인한 고통이 수반될 수 있다.

4. 오늘 실습을 통해 새롭게 배운 것들이 향후 간호실무에 어떻게 적용될 수 있겠는가?

• 자궁경부암은 최소 검사 48시간 전부터 질 내 어떠한 물질도 넣지 않아야 하며, 다음 사항을 지켜야 한다. ① 성관계 금지 ② 탐폰 사용금지 ③ 질세척 금지 ④ 질 내 약물 및 윤활제 사용 금지 ⑤ 질 내 피임약 사용 금지

• 유방검진은 수검자는 검사 중 어떤 시기에라도 검사를 멈출 수 있으며, 검사자는 수검자가 검사를 거부하였을 때 그에 대해 인정하고 권리를 존중해야 한다.

5. 오늘 실습에서 좋았던 점과 아쉬웠던 점은 무엇인가?

바빠서 검진 받지 못하시는 분과 지금 질병 치료 중이셔서 검진을 받지 못하시는 분들을 보며 마음이 아팠고 지속적인 관심과 독려를 통해 잠깐이라도 남는 시간에 검진을 받으셔서 질병을 예방 하셨으면 좋겠다는 생각을 했다.

CamScanner로 스캔하기

오늘은 보건소 캠페인을 하는 날이었다. 선생님들을 도와 부스를 설치하고 몇가지 부스를 돌아가며 일손을 도왔는데 일반 시민분들을 많이 만나게 된것이 좋았고 보건소에서 각 부서별 추진하는 사업과 중점적으로 다루고 있는 일들에 대해서도 알게되어 좋았다.

2. 오늘 실습을 하는 동안 나의 생각과 느낌은 어떠했는가?

공원에서 부스를 설치했는데 근처 어린이집에서 어린이들이 단체로 와서 순간적으로 어린이 공원에 와있는 것 같은 착각이 들었다. 홍보가 잘 될까 하는 생각도 들었고 어린아이들이 게임과 체험들에 적극적으로 참여하는 모습이 보기 좋았다. 어릴 때부터 건강한 습관을 만드는 것이 중요한데 보건소에서 그것을 돕는 활동이 잘 이루어지고 있는 것 같았다.

① #보건[illegible] 인식 1-3

3. 오늘 실습을 통해 새롭게 알게 된 것은?

· 진드기 매개 감염병 이란 세균이나 바이러스에 감염된 진드기에 물려 발생하는 감염병이다. 주로 텃밭이나 농작업등을 하거나 등산, 나물채취 등의 야외작업을 하는 경우 감염위험이 있다. 쯔쯔가무시증, 중증열성혈소판감소증(SFTS), 라임병 등이 걸릴 수 있다. 증상으로는 발열, 근육통, 설사 등 감기 몸살 증상과 유사하므로 빠른 진단을 위해서 병원 진료시 농작업 또는 야외활동력 의료진에게 알려야한다.

4. 오늘 실습을 통해 새롭게 배운 것들이 향후 간호실무에 어떻게 적용될 수 있겠는가?

· 환자 5명 중 1명이 사망하는 치명적인 질병으로 아프면 의료기관에 방문을 권하고 진드기에 물리지 않도록 예방 수칙을 지키도록 교육한다. 예방수칙을 지키면 충분히 예방이 가능하다. 증상을 살피고 몸전체를 살핀 후 이상이 있으면 의료기관 방문하도록 교육한다. (→ 야외활동을 한 후)

5. 오늘 실습에서 좋았던 점과 아쉬웠던 점은 무엇인가?

여러가지 분야의 캠페인을 보게되어 다양한 경험을 쌓을 수 있어 좋았다. 특히 평소에는 접해보지 않았었는데 낯설었지만 홍보하시는 내용에 대해서도 공부할 수 있는 기회가 되어 좋았다.

1-3 # → 구강보건사업

1. 오늘 실습경험의 의미 있는 점은 무엇인가?

#[illegible] 인식

오늘은 금연 캠페인 중 금연 저금통 만들기 부스에서 어린아이들을 대상으로 저금통 만드는 것을 도왔다. 아이들이 금연의 필요성과 중요성에 대해 다시 한번 배울수 있고 부모님에게도 권유할 수 있게 된다는 점이 의미 있었다.

2. 오늘 실습을 하는 동안 나의 생각과 느낌은 어떠했는가?

아이들에게 부모님이나 가족 중 담배를 피는 분이 계시냐고 물어봤는데 아빠, 할아버지 등 각 가구마다 1명씩은 담배를 피우신다는 것을 알수있었고 저금통을 채워서 금연하시는 분에게 전달해 주겠다는 생각을 가진 친구들도 있어서 캠페인의 취지에 맞게 잘 따라준 것 같다.

3. 오늘 실습을 통해 새롭게 알게 된 것은?

• 혈액순환이 안될때 증상은?
1. 손발저림. 다른 질환에 의해 생기기도 하지만 혈관에 문제가 있으면 손발저림과 시림 증상이 가장 흔하게 나타난다.
2. 기억력 감퇴. 뇌로 가는 혈액공급이 원활하지 않으면 뇌기능이 서서히 떨어지게 되고 그 결과 기억력 감퇴가 생기기 시작한다. 3. 뒷목 뻐근함, 어깨 결림 → 뇌로가는 혈류량 충분X 4. 잦은 피로감이나 무기력증 → 혈액순환X면 혈액 속 젖산이 쌓이므로 피로함, 무기력증 느끼게된다. 5. 변비나 불면증, 몸에 멍 잘 듦, 부종

4. 오늘 실습을 통해 새롭게 배운 것들이 향후 간호실무에 어떻게 적용될 수 있겠는가?

<예방 방법> ?

1. 금연 → 급성심근경색 발병요인 중 흡연이 65%를 차지해 가장 높은 원인으로 지적된다. 니코틴은 혈관탄력성 유지시키는 내피세포를 파괴하고 혈전 생성 촉진시키는 동맥경화를 유발하는 위험물질로 알려져 있다. 2. 금주 → 도수가 높은 술을 갑자기 많이 마시면 1시간 이내 심근경색 발생위험은 72%나 높아진다. 3. 장시간 앉지 않기

5. 오늘 실습에서 좋았던 점과 아쉬웠던 점은 무엇인가?

아이들과 함께 저금통을 만드는게 재미도 있었고 귀여운 모습을 보며 힘이 났었는데 오전이 어린이집 현장실습이 끝나고 더 이상 아이들을 볼수 없어 아쉬웠다.

CamScanner로 스캔하기

1. 오늘 실습경험의 의미 있는 점은 무엇인가?

오늘은 방문보건 팀에서 하시는 일을 관찰하였는데 암환자에게 영양제를 나눠주시고 경로당에 가셔서 혈압과 혈당을 측정하고 운동프로그램이 잘 이루어지고 있는지 관리하는 일을 도와드렸다.

2. 오늘 실습을 하는 동안 나의 생각과 느낌은 어떠했는가?

경로당에 들어가 본 것은 처음이었는데 운동기구와 악기 등이 많이 보였고 외부 강사님들이 일정에 맞춰 프로그램을 진행한다는 것을 알게 되었다. 광산은 시스템이 잘되어 있다는 생각이 들었다. 어르신 분들이 집에 계시는 것보다 나오셔서 활동하시도록 시스템이 잘 구축되어 있다는 생각이 들었다.

3. 오늘 실습을 통해 새롭게 알게 된 것은?

방문건강 맞춤형 방문관리
건강서비스 이용이 어려운 사회문화·경제적 건강취약계층을 대상으로 찾아가는 맞춤형 건강관리 서비스 제공 및 보건소 내외 연계를 통해 대상자의 건강상태 유지 및 수준 향상에 기여하는 사업이다.

4. 오늘 실습을 통해 새롭게 배운 것들이 향후 간호실무에 어떻게 적용될 수 있겠는가?

- 집단시설 건강관리는 연중으로 관내 경로당 이용 어르신들을 대상으로 집단시설(경로당) 기초검진 및 보건교육을 실시한다. (V/S, BST)
- 취약 가구 방문관리는 연중으로 기초생활수급자 및 건강관리 서비스 이용이 어려운 사회, 문화, 경제적 취약계층 및 65세 이상 독거노인 가구, 75세 이상 노인부부 가구 중심으로 방문간호사 지역담당제로 방문간호사 서비스를 제공한다.

5. 오늘 실습에서 좋았던 점과 아쉬웠던 점은 무엇인가?

방문간호를 가야하는 다른 기관이 있었는데 사무실공사문제 인해 복귀해야 했다. 가지 못했던 것이 아쉬움이 남는다.

CamScanner로 스캔하기

1. 오늘 실습경험의 의미 있는 점은 무엇인가?

오늘은 치매분소에서 실습이 있는 날이었다. 어르신들이 예약을 하시거나 의심이 되셔서 방문하셨다. 오셔서 10분 남짓한 테스트를 받으시는데 정상이하의 점수가 나오시에는 1시간 걸리는 테스트를 하고 의심시 서류를 받아 병원에 방문하는 시스템이였다. 보건소에서 다양한 경험을 할 수 있어 의미있는 것같다.

2. 오늘 실습을 하는 동안 나의 생각과 느낌은 어떠했는가?

한 어르신이 자꾸 생각을 떠올리는게 느리고 힘들다고 하시며 방문을 하셨는데 테스트 전에 있었던 일들을 들려주셨다. 이야기를 듣는 와중에 나도모르게 몰입이 되어 걱정되는 마음이 들었고 노화라는 것이 참 슬프다는 생각이 들었다. 많은 분들이 건강하셨으면 좋겠고 혹시라도 증상이 있을 때 조기발견하셔서 증상을 늦출수 있었으면 좋겠다는 생각을 했다.

노인간호 슬프다.

3. 오늘 실습을 통해 새롭게 알게 된 것은?

치매예방 수칙 333 3권, 3금, 3행

3권 운동: 일주일에 3번 이상 걸으세요.
식사: 생선과 채소를 골고루 챙겨 드세요
독서: 부지런히 읽고 쓰세요

3금 절주: 술은 한번에 3잔보다 적게 마시세요
금연: 담배 x
뇌손상예방: 머리 다치지 않도록 조심.

3행 건강검진: 혈압, 혈당, 콜레스테롤 3가지 정기적으로 체크
· 소통: 가족과 친구들 자주 연락하고 만나세요
치매조기발견: 매년 보건소에서 치매조기검진 받으세요.

4. 오늘 실습을 통해 새롭게 배운 것들이 향후 간호실무에 어떻게 적용될 수 있겠는가?

위의 치매예방 수칙 333을 대상자에게 주기적으로 설명한다.
식사시에는 거르지 마시고 기름진 음식은 피하고 싱겁게 드시고, 5층이하는 걸어다니시고, 버스 한 정거장 정도는 걸어가셔도 좋습니다, 틈날 때마다 책이나 신문을 읽으시고 글쓰기를 하세요. 운동하실 땐 보호장구를 반드시 착용하고 머리는 부딪혔을 땐 바로 검사를 받으세요.

5. 오늘 실습에서 좋았던 점과 아쉬웠던 점은 무엇인가?

정신간호 실습때 옆에 있던 치매안심센터에 대해 궁금함이 있었는데 이번 실습으로 경험해 볼 수 있어 좋았다.

CamScanner로 스캔하기

1. 오늘 실습경험의 의미 있는 점은 무엇인가?

간호전문직

폐렴 접종을 하러오신 대상자 분들에게 예방접종을 하시는 과정을 도와드렸다. 문진표 작성을 도와드리고 체온을 측정하고 선생님들이 접종하시는 모습과 안내하시는 모습을 보았는데 폐렴주사가 아픈축에 속하는 주사라 아프실 수 있다고 미리 말씀드리고 접종부위에 신경써서 최대한 통증없도록 주사하시는 모습에서 노하우가 있으시다는 생각이 들었고,

2-1

2. 오늘 실습을 하는 동안 나의 생각과 느낌은 어떠했는가? 이런점을 배워야 겠다고 생각했다.

OT를 들으면서 그동안 알지못했던 예방주사 항목에 대해 알게되어 좋았고 많이듣고 배워서 알고있지만 자세히 알지못하는 내용에 대해서도 이번 기회에 개념을 정립하게되어 유익한 시간이었다.

3. 오늘 실습을 통해 새롭게 알게 된 것은?

결핵(BCG) 예방접종 후 나타날 수 있는 이상반응으로는 국소농양, 림프절염, 기타 이상반응이 나타난다. 국소농양은 백신을 피하층 주입시 나타나고 접종부위에 궤양이 생기고 고름이 생길 수 있다. 대부분 자연치유 되므로 병변을 깨끗이 닦아주고, 연고, 항생제, 수술은 필요하지 않다.

림프절염은 겨드랑이나 목의 림프절 비대가 BCG 예방접종 후 2주~1년 이내에 발생할 수 있으며 통증은 없다. 대개 수개월 이내 자연 치유된다.

기타이상반응으로는 접종부위 마농성 궤양이나 켈로이드 같은 국소이상반응과 매우 드물게 전신 파종성 BCG 감염증, 골수염 등이 나타날 수 있다.

4. 오늘 실습을 통해 새롭게 배운 것들이 향후 간호실무에 어떻게 적용될 수 있겠는가?

2-1

BCG 예방접종 후 정상 경과과정에 대해 설명한다. 접종직후 접종 부위가 부풀어 올랐다가 10~15분이 지나면 없어집니다. 2~4주에는 접종부위 붉은 점이 나타나고 몽우리가 점점 커지고 단단한 결절이(10mm) 됩니다. 4~6주에는 농주머니 달고 있는 피부를 뚫고 고름이 나오기도 하여 궤양을 형성합니다. 6~9주에는 궤양이 아물며 딱지가 형성되고 이때 딱지를 누르면 고름이 나오며 점차 나오지 않게 됩니다. 9~12주에는 딱지가 떨어지고 2~3mm 크기의 반흔을 남기며 아물게 됩니다.

5. 오늘 실습에서 좋았던 점과 아쉬웠던 점은 무엇인가?

아이들의 접종모습이 궁금했지만 집단회로 오전 관찰이 불가능 하였는데 경험할 수 있는 기회가 있었으면 좋았겠다라는 아쉬움이 남았다.

CamScanner로 스캔하기

P22

1. 오늘 실습경험의 의미 있는 점은 무엇인가?

오늘은 6월 9일 구강의 날이었다. 어제 학생들이 만들어 놓았던 불소를 나누어드리고 대상자분들을 안내하는 역할을 맡았었다. 불소의 효과와 치아 우식증을 예방할 수 있는 법을 알게되었고 홍보활동에 필요한 준비물을 준비하며 구강보건에 대해 알게되어 의미있었다.

2. 오늘 실습을 하는 동안 나의 생각과 느낌은 어떠했는가?

안내데스크에 구강과 관련된 책이 많이 있었는데 대상자가 오지 않을때는 시간이는 책을 읽게되었다. 어린이를 대상으로 한 책들이 대부분이 였지만 치아와 구강관리에 대해서 설명이 되어있어 도움이 되었다. 어릴때부터 이런 서적들을 자연스럽게 접하고 구강관리에 신경쓰는 것이 참 바람직하고 보건소에서 하시는 일들이 의미가 깊었을 생각이 들었다.

구강보건 사업.

3. 오늘 실습을 통해 새롭게 알게 된 것은?

•치아우식증의 주원인은 치면세균막(프라그)이다. 치면세균막은 치아면의 음식물 찌꺼기와 세균의 혼합물로, 제거되지 않은 상태로 오래 방치되면 치아우식증을 일으킨다. 충치예방을 위한 치아홈메우기란 치아의 홈이 충치가 발생하기 전에 플라스틱과 유사한 치과용 재료로 미리 막아 음식물이 끼지 않고 세균이 자라지 않게 하는 방법을 치아홈메우기라고 한다. 올바른 칫솔질과 치아홈 메우기를 통해 실천하면 충치의 약 65~90% 예방 효과가 있는 것으로 나타난다.

4. 오늘 실습을 통해 새롭게 배운 것들이 향후 간호실무에 어떻게 적용될 수 있겠는가?

치아우식증의 예방법에 대해 설명한다. 올바른 칫솔질을 하고 설탕이 함유되고 끈적끈적한 음식물은 충치를 잘 일으키므로 섭취를 줄이고 과일이나 야채 같은 음식을 섭취해야합니다. 치아홈메우기고 불소를 사용하여 제공해줍니다. 불소는 치아를 단단하게 하여 치아우식증이 생기지 않도록 합니다. 그리고 6개월에서 1년에 한번씩 정기 구강검사를 받아 치아우식증을 예방해야 합니다.

5. 오늘 실습에서 좋았던 점과 아쉬웠던 점은 무엇인가?

평소 충치가 잘 생기는 편이였는데 구강관리방법에 대해 알게되어 앞으로 배운것을 바탕으로 관리해야겠다는 의지가 들었다. 나 뿐만 아니라 주위 사람들, 대상자분에게도 구강 관리가 필요하신 분들에게 도움을 드릴 수 있을것 같아 유익한 시간이였던 것 같다.

본인의 구강관리 교육.

CamScanner로 스캔하기

P23

1. 오늘 실습경험의 의미 있는 점은 무엇인가?

오늘 실습 중 금연클리닉에서 실습을 하게 되었는데 금연을 하기 위해 스스로 찾아와서 의지를 보이는 것이 의미있었다. 지인의 소개로 찾아온 대상자를 정확히 확인하고 흡연을 얼마나 하는지 확인하는 점이 의미있었다.

2. 오늘 실습을 하는 동안 나의 생각과 느낌은 어떠했는가?

실습을 하는 동안 대상자가 스스로 금연클리닉에 찾아오는 것 자체가 신기했고, 금연의 의지를 가지고 그 자리에서 가지고 있던 담배를 버리고 패치를 가져가는 모습이 새로웠다. 또한, 일주일마다 점검을 하기 위해 오는 대상자도 있었는데 마찬가지로 본인이 열심히 줄이고 있었지만 눈 앞에 담배가 보여서 한개피를 피웠다고 말하는 것을 보고 금연이 어렵구나 하는 생각이 들었다.

금연, 건강증진 토론

3. 오늘 실습을 통해 새롭게 알게 된 것은?

니코틴 패치의 단계가 있고 숫자가 작을 수록 니코틴 함량이 더 많다는 것을 알게 되었다. 또한, 니코틴 패치를 붙이고 있을 때 담배를 핀다면 X2가 된다는 것도 설명해주셔서 알 수 있었다.

금단증상을 대상자가 호소하였는데 쉽게 알 수 있는 손떨림, 불안 이런것이 아니라 변비를 호소하여 변비도 생길 수 있구나 하는 생각이 들었다.

4. 오늘 실습을 통해 새롭게 배운 것들이 향후 간호실무에 어떻게 적용될 수 있겠는가?

향후 흡연을 하는 대상자에게 지역 금연클리닉을 소개해줄 수 있고,

~~금연클리닉에~~ 금연에 중요성·필요성과 장점등을 교육할 수 있다.

5. 오늘 실습에서 좋았던 점과 아쉬웠던 점은 무엇인가?

-좋았던 점 = 보건소 실습이 처음이라 보건소의 업무에 대해 알게되어 좋았고 금연클리닉에 업무와 환경을 실습할 수 있어서 좋았다.

-아쉬운 점 = 건강보험공단을 가보지 못하여 아쉽다.

CamScanner로 스캔하기

1. 오늘 실습경험의 의미 있는 점은 무엇인가?

오늘은 방문간호를 시행하였다. 나운 1동 담당 선생님과 함께 재택 방문 간호를 하러 갔는데 독거노인 할머니였는데 고혈압, 당뇨를 가지고 계셨고 더운데 에어컨도 없이 여름을 보내고 계시다는게 놀랐고 입맛이 없어서 식사를 잘 안하신다는것에 또 놀랐다. 열악한 환경

방문간호를 처음 가봤는데 방문해서 그날동안에 상태를 파악하고 정기적 관찰을 하는것이 의미있었다.

2. 오늘 실습을 하는 동안 나의 생각과 느낌은 어떠했는가?

방문간호는 처음 해보았는데 어르신들이 너무 친절하게 맞이해주셔서 좋았고 선생님이 대상자 한분 한분 몸 상태 변화를 기억하고 물어보고 챙기는게 대단하다는 생각이 들었다. 또한 방문 간호사 혈압이 너무 높게 나와서 병원을 가라고 한 대상자에게 병원 다녀왔냐고 물어보고 약 복용 여부를 확인하고 조절이 잘 되는지까지 확인하는 것을 보고 방문간호의 필요성을 느끼게 되었다.

3. 오늘 실습을 통해 새롭게 알게 된 것은?

방문간호라고 해서 개인의 가정에만 방문하는 줄 알았는데 또 경로당에도 방문하여 단체로 관리하는 것을 보고 새롭게 알게 되었다.

또한 고혈압, 당뇨 뿐 아니라 암환자도 관리할 수 있다.

기본적인 건강관리와 교육, 상담등을 제공한다.

암투병중 식단관리 방법 제공, 혈당과, 혈압 수치 검사 뿐 아니라 암 치료 중 필요한 소모품, 영양제 등 물품지원도 이루어 진다.

4. 오늘 실습을 통해 새롭게 배운 것들이 향후 간호실무에 어떻게 적용될 수 있겠는가?

향후 간호실무에서 방문간호사가 된다면 대상자를 정확하게 파악하고 생활환경도 보며 올바른 교육과 상담, 건강관리를 도울수 있을 것이다.

5. 오늘 실습에서 좋았던 점과 아쉬웠던 점은 무엇인가?

- 좋았던 점 = 방문간호를 처음으로 경험하게 되어서 좋았고 경로당에 방문하게되어 신기하고 좋았다.

- 아쉬운 점 = 너무 더웠고 대상자를 찾지 않고 없으면 어쩔 수 없이 안하는 것이 아쉽다고 느껴졌다.

CamScanner로 스캔하기

1. 오늘 실습경험의 의미 있는 점은 무엇인가?

[illegible] 결핵예방접종 BCG에 대해 알게 된 점이 가장 의미있었다.
우리나라는 OECD 국가 중 가장 높은 결핵 사망률을 보이고 있다.
결핵에 감염을 예방하기 위해 모든 영유아 (만5세 미만)을 대상으로 접종을 한다는 것을
알게 되었고 생후 4주 이내에 1회 접종하는것이 권장된다는 것을 알게 된 점이 의미 있었다.

2. 오늘 실습을 하는 동안 나의 생각과 느낌은 어떠했는가?

실습을 하는동안 결핵 (BCG) 예방접종이 BCG 경피접종 (도장형)인 줄 알았는데
BCG 피내접종 (주사형)도 있다는 것을 알게 되어 ~~실습~~ 신기했고 우리나라에서는 피내접종
방법으로 접종할 것을 권장하고 있으며 WHO 에서도 피내접종을 권장한다는 것을 알았다.
그러나 나는 경피접종으로 접종하여 팔에 아직도 자국이 남아있는데 ~~결핵~~ 피내접종이
더 권장된다는 것을 보고 조금 내가 결핵에 걸리지 않는것이 다행이라는 생각이 들었다.

3. 오늘 실습을 통해 새롭게 알게 된 것은?

BCG 예방접종 후 정상 경과 과정을 알게 되었는데 다른 예방접종과 달리
접종부위에 멍우리가 생기고 결절이 만들어지고 농주머니가 생긴다. 농주머니를 덮고 있는
피부를 뚫고 고름이 나오기도 하며 궤양을 형성한다. 궤양이 아물면서 딱지가 형성되고
딱지를 뜯으면 고름이 나오고 점차, 고름이 나오지 않게 된다. 이러한 고름이 ~~나오면~~ 나오면
예방접종이 잘못 된것이라 생각하게 되는데 결핵은 이 과정이 정상이라는 것을 새롭게 알게 되었다.

4. 오늘 실습을 통해 새롭게 배운 것들이 향후 간호실무에 어떻게 적용될 수 있겠는가?

향후 간호실무에서 결핵 접종하는 아이의 부모에게 결핵 접종 후 고름이 나오는 것은
비정상 소견이 아니라고 설명하며 경과과정이 올바른 상황이라는 것을 설명할
수 있다.

5. 오늘 실습에서 좋았던 점과 아쉬웠던 점은 무엇인가?

-좋았던점: 예방접종 하는것을 실제로 가까이에서 볼 수 있어서 좋았고
예방접종 ~~예방~~ 예진표 작성도 도울수 있어서 좋았다.
-아쉬운점: 결핵 BCG 예방접종 하는 것을 못 보게 되어 아쉬웠다.

CamScanner로 스캔하기

1. 오늘 실습경험의 의미 있는 점은 무엇인가?

오늘은 접수실에서 실습을 하였는데 각종 민원의 안내를 하는 업무를 하였다.
보건증이나 아토피 지원, 각종 검사 접수 및 수납 후에 안내하는 일을 한다는
것을 알게되어 의미있었다.
또한, 다른 곳에서는 어떤 일을 담당하고 민원 안내를 하는지 알 수 있어 좋았다.

2. 오늘 실습을 하는 동안 나의 생각과 느낌은 어떠했는가?

접수실은 평소 보건소를 방문하면 자주 오는 곳인데 수납, 접수 등을 하는 곳이라고는 알고 있었는데
전화 안내도 함께 하는지는 몰랐고 건강진단결과서(보건증)을 접수하러 자주 오는데
만15세부터 된다는 것을 알게 되어 신기했다.
또한, 보건증의 유효기간이 종류마다 다른 것을 새롭게 알게되어 좋았다.

3. 오늘 실습을 통해 새롭게 알게 된 것은?

보건증 유효기간
-식품1년, 학교급식 6개월, 유흥은 3개월의 유효기간이 있다.
사전연명의료의향서 작성과 관련된 업무들은 주로 보건행정과에서 담당한다고 알게 되었다.
군인 입소용 건강진단서는 입대하는 군인의 종류마다 다르고 의경은 보건증만 필요하다면
해경은 폐결핵, 소변, B형 간염, 매독 검사까지 해야한다는 것을 새롭게 알게되었다.

4. 오늘 실습을 통해 새롭게 배운 것들이 향후 간호실무에 어떻게 적용될 수 있겠는가?

향후 보건소에서 어떠한 일을 하는지 알고 환자나 보호자에게 설명할 수 있고
보건소에서 일한다면 어떤 부서에서 일을 담당하는지 알고 설명할 수 있다.

5. 오늘 실습에서 좋았던 점과 아쉬웠던 점은 무엇인가?

-좋았던 점 = 보건소 각 과마다 무슨일을 하고 민원인을 어디로 안내해야 하는지 알고 안내할 수 있어서 좋았다.

-아쉬운점 = 실습을 동들이 건강보험공단을 실습하지 못한 점이 가장 아쉽다.

훌륭해

CamScanner로 스캔하기

1. 오늘 실습경험의 의미 있는 점은 무엇인가?

•보건소 OT (현장지도자 OT. 치매안심센터 소개. 감염병관리계, 공단 소개)
•교수님 OT. 셀프리더십 자가평가
•방문보건 (재가암 대상자 BP, BST + 비타민 배급)

2. 오늘 실습을 하는 동안 나의 생각과 느낌은 어떠했는가?

취업 시기라서 그런가 셀프리더십 자가평가 점수가 되게 낮아서 놀라웠다.
조금 더 자신감을 가질 필요성을 느꼈다.
보건소 OT 중 부서의 업무를 간단히 보여주었는데 감염병 대응계와
감염병관리계의 업무 분류는 어떻게 되는 건지 궁금하였다.

3. 오늘 실습을 통해 새롭게 알게 된 것은?

치매에 대한 편견을 학습했다.
1. 치매는 노인병이 아니다. (일부)
2. 치매는 유전되지 않는다. (일부, 가족성 알츠하이머)
~~[illegible]~~ 공단에 대한 OT를 학습하며 취업 방법에 대해 안내해주셨는데
걱정했던 [illegible] 이러 [illegible] 조언이 와닿았고 시간내주어 설명해주심이 감사했다.

4. 오늘 실습을 통해 새롭게 배운 것들이 향후 간호실무에 어떻게 적용될 수 있겠는가?

대상자 방문보건시 간호사 선생님께서 대상자의 말을 줄이고 빨리 나가려는
태도를 취하셨다. 아무리 바쁘더라도 대상자에게 예의를 갖출 수 있는
간호사가 되어야겠다고 생각하였다. 의사소통

5. 오늘 실습에서 좋았던 점과 아쉬웠던 점은 무엇인가?

오리엔테이션이 있어 선생님께서 배려해주시어 오전에 방문보건을 다녀와
주셔서 감동을 받았다. 교수님의 말씀대로 대상자 정보에 대한 질문을
했어야 했는데 이를 놓쳐 아쉬운 마음이 든다.

1. 오늘 실습경험의 의미 있는 점은 무엇인가?

· 예방접종실 : 예방접종 안전인 1과 안내

· BCG 피내 접종 관찰

2. 오늘 실습을 하는 동안 나의 생각과 느낌은 어떠했는가?

보건소 예방접종실에는 생각보다 다양한 나라의 사람이 방문하여 다양한 종류의 예방접종을 시행한다는 것이다.
또한 15세 이상 여아 대상과 HPV 예방접종은 서바릭스와 가다실4가 중 택할 수 있는 것인지 묻는 경우 수요에 따라 결정되지만 주로 가다실4가는 접종한다고 하셨다.

3. 오늘 실습을 통해 새롭게 알게 된 것은?

결핵(BCG) 예방접종은 접종방법에 따라 피내접종과 경피접종으로 구분된다.
① BCG 피내접종(주사형): 피부에 15° 각도로 바늘 사면이 완전히 삽입한 후 백신 주입, 접종량이 상대적으로 일정하고 정확함. 안전O
② BCG 경피접종(도장형): 피부에 주사약 바르고 9개 바늘로 가진 주사를 이용해 2번에 걸쳐 강하게 눌러 접종, 접종량 일정 X 수 있음, 안전O

4. 오늘 실습을 통해 새롭게 배운 것들이 향후 간호실무에 어떻게 적용될 수 있겠는가?

관내시민 대상 독감 접종 예방주사는 무료로 해드리기 위해 최대한 노력하는 선생님의 모습을 보고, 대충 넘겨도 될 일이라고 생각한 일이라도 상황을 대상자를 위해 노력하는 간호사가 되어야겠다고 생각했다.

5. 오늘 실습에서 좋았던 점과 아쉬웠던 점은 무엇인가?

흔치 않은 BCG 피내접종을 2회 볼 수 있어 신기했고 선생님께서 직접 백신과 함께 설명해주셔서 감사했다

1. 오늘 실습경험의 의미 있는 점은 무엇인가?

◦ 건강보험공단 군산지사 1일차 실습

◦ 건강검진 유선독려 150명 진행

◦ [illegible] 진행

2. 오늘 실습을 하는 동안 나의 생각과 느낌은 어떠했는가?

국민건강보험공단 중 악성민원도 있다는 것은 알고 있었지만 [illegible] 놀랐다. 오늘 9:30부터 전화업무를 시작하며 이른 시간에 전화로 [illegible] 응답하며 [illegible].
이러한 민원인이 있긴 했지만 악성에 비해 순한 편이었다.

3. 오늘 실습을 통해 새롭게 알게 된 것은?

국민건강보험공단은 생애주기에 [illegible] 검진에 따라 상세한 [illegible] 할 수 있었다. 담당선생님께서는 간호학이시고 간호학에도 간호사와 영양사가 [illegible] 간호사와 [illegible] 있었다. 간호사가 [illegible] 않고 경력을 모두 인정해주어 좋은 직장이라고 설명해 주셨다.

4. 오늘 실습을 통해 새롭게 배운 것들이 향후 간호실무에 어떻게 적용될 수 있겠는가?

실습을 경험하고 현재 [illegible] 선생님께 [illegible] 할 수 있는 기회에 너무 감사했다. 나 또한 후배간호사에게 영향을 주는 간호사가 되어야 겠다.

5. 오늘 실습에서 좋았던 점과 아쉬웠던 점은 무엇인가?

교육을 경험할 수 있음에 감사했고 [illegible] 하러 오신분들에게 [illegible] 진행하여 감사하는 마음과 [illegible] 마음이 공존했다.
교육은 더 경험해보지 못함에 조금 아쉬움은 남는다.

CamScanner로 스캔하기

1. 오늘 실습경험의 의미 있는 점은 무엇인가?

군산시보건소 실습은 처음인데 첫날은 금연상담실로 출근하게 되었다.
금연을 결심하신분들이 찾아오셔서 등록을 하거나 금연 패치 스티커, 사탕을 받아가는 모습을 관찰하였다.

2. 오늘 실습을 하는 동안 나의 생각과 느낌은 어떠했는가?

건강증진활동

생각보다 다양한 연령대의 사람들이 금연을 결심하고 상담 받으러 온다는 것을 느꼈고 어린이집 선생님도 오셔서 금연구역 스티커와 꼭 붙여야 하는것을 나라에서 지정한 스티커들을 받아가는 것을 보고 새로웠다.

3. 오늘 실습을 통해 새롭게 알게 된 것은?

금연상담실에 등록 절차는 금연클리닉 카드에 등록하고 흡연자평가 실시 후 금연계획을 수립하고 내용 및 방법으로 니코틴 보조제와 대체용품을 제공하고 대면상담과 + 전화상담을 6개월간 총 9회 이상 실시하고 중간평가를 금연결심일로부터 4, 6, 12 주에 실시하고 최종평가는 금연결심일로 부터 24주째에 평가한다는 것을 알게되었다.

4. 오늘 실습을 통해 새롭게 배운 것들이 향후 간호실무에 어떻게 적용될 수 있겠는가?

보건소의 금연상담실에서 일하게 된다면 어떤 프로그램과 상담이 있는지 알 수 있고 능숙하게 금연 도움 물품들을 드리고 대상자분들에게 맞는 상담을 제공할 수 있다.

5. 오늘 실습에서 좋았던 점과 아쉬웠던 점은 무엇인가?

∨ 군산시 보건소 실습은 처음이었는데 금연상담실에서 어떤 것을 하는지 직접 볼 수 있어서 좋았고 선생님들께서도 잘해주셔서 좋았습니다.

CamScanner로 스캔하기

1. 오늘 실습경험의 의미 있는 점은 무엇인가?

오늘은 예방접종실로 출근하게 되었는데 담당 선생님께서 이곳에서 사용하고 있는 예방접종들을 알려주시고 어떤걸 하면 되는지 안내해주셔서 감사했고 백신 접종의 금기사항에 대해 공부하였다.

2. 오늘 실습을 하는 동안 나의 생각과 느낌은 어떠했는가?

베트남에서 살다오신분들이 고혈압병주사를 맞는다고 오셨는데 군산 보건소에서는 고혈압병 주사를 맞을 수가 없어서 선생님들께서 다른 병원을 추천해주셨는데 친절하다 느꼈고 생각보다 다양한 인종의 사람들이 보건소를 찾는다는 것을 느끼게 되었다.

3. 오늘 실습을 통해 새롭게 알게 된 것은?

예방접종 백신접종의 주의사항은 면역생성을 저하시킬 수 있는 상태.
예) 수혈을 받은 후 홍역에 대한 혈중 항체가 있는 사람에게 홍역 포함 백신을 투여하는 경우 등이고 백신접종의 금기사항은 백신을 접종 받은 사람에게 심각한 부작용이 발생할 가능성이 아주 높은 경우이며, 이는 백신 자체의 문제가 아닌 백신접종 받는 사람의 상태를 말하고 금기사항이 있는 경우 백신접종을 해선 안된다는 것을 알게 되었다.

4. 오늘 실습을 통해 새롭게 배운 것들이 향후 간호실무에 어떻게 적용될 수 있겠는가?

예방접종실에서 일하게 된다면 대상자분들에게 어떤것을 작성하고 와야하는지, 절차, 설명을 할 수 있고 백신과 예방접종 항목에 대해 알기쉽게 설명하고 접종해드릴 수 있을 것이다.

5. 오늘 실습에서 좋았던 점과 아쉬웠던 점은 무엇인가?

어제보다 할일이 많이 있어서 좋았고 선생님께서 하나하나 자세하게 설명해주시고 해야하는 일을 알려주시고 시켜주셔서 좋았습니다.

CamScanner로 스캔하기

지역사회활동

1. 오늘 실습경험의 의미 있는 점은 무엇인가?

오늘은 외부의 치매안심센터에 오게되어 선생님들과 함께 수산시장에 가서 치매예방 캠페인과 심혈관질환 예방 캠페인을 진행하고 선물을 나눠드리면서 설명드리고 사인받는것을 하였다.

2. 오늘 실습을 하는 동안 나의 생각과 느낌은 어떠했는가?

치매안심센터에 처음와보게 되었는데 보건소와 비슷한 느낌이 들었고 쾌적하다고 생각되었다. 또한 수산시장에서 상인분들에게 캠페인을 하며 소통하는 것이 재밌다는 생각이 들었고 보건소에서 이런 ~~[illegible]~~ 이벤트를 진행하는게 좋은 활동이라 느껴졌다.

3. 오늘 실습을 통해 새롭게 알게 된 것은?

치매 예방센터에서는 직접 방문하여 상담을 진행하는 것도 있지만 어르신분들을 대상으로 기관 내에서 프로그램과 상담을 진행하여 직접 기관으로 찾아오시는분들도 많다는 것을 느꼈다. 또한 어르신들을 대상으로 장구공연을 하게되어 연습하는 것을 보았다.

4. 오늘 실습을 통해 새롭게 배운 것들이 향후 간호실무에 어떻게 적용될 수 있겠는가?

보건소에서 근무할 때 노인분들께 치매예방법과 관리방법 등을 설명드리고 프로그램을 진행할 수 있고 어르신 분들이나 상인분들을 대상으로 한 캠페인을 진행할 수 있다.

5. 오늘 실습에서 좋았던 점과 아쉬웠던 점은 무엇인가?

수산시장에 가서 캠페인을 해보며 상인분들과 소통할수 있어서 좋았고 치매예방 ~~[illegible]~~을 위한 프로그램과 활동 등이 어떤것들이 있는지 볼 수 있어서 좋았다.

CamScanner로 스캔하기

1. 오늘 실습경험의 의미 있는 점은 무엇인가?

오늘은 모자보건실 부서로 출근하게 되었는데 이곳은 난임 심리 · 의료 상담과 출산 · 육아지원제도, 엄마와 아가의 건강지원 서비스를 알려주고 도와주는 활동을, 하는곳이었다.

2. 오늘 실습을 하는 동안 나의 생각과 느낌은 어떠했는가?

출산을 앞둔 산모분께서 남편과 함께 이곳에 방문하셨는데 두분다 맞벌이를 하고있어서 이럴 때 지원받을 수 있는 것들을 이곳에 선생님들처럼 설명해주시는 분들이 없으면 알기 힘들겠다는 생각이 들었고 이런 사업이 더욱 확대되어야 출산율도 높아질것 같다는 생각이 들었다.

3. 오늘 실습을 통해 새롭게 알게 된 것은?

출산전후 휴가 기간에 매월 통상임금의 100%를 지급해주고 만 8세 이하 또는 초등학교 2학년 이하의 자녀를 가진 근로자가 그 자녀를 양육하기 위해 사업주에게 신청하면 1년의 육아기 근로시간 단축을 부여받을 수 있다는 것을 알게되었다.

제도안

4. 오늘 실습을 통해 새롭게 배운 것들이 향후 간호실무에 어떻게 적용될 수 있겠는가?

산모와 아이를 위한 혜택이 어떤 것들이 있는지 알고 도와주고 설명해줄수 있고 일하는 맞벌이 부부들을 위한 출산 · 육아지원 제도에 대해 설명할 수 있고 혜택을 받을수 있도록 도움을 줄 수 있다.

5. 오늘 실습에서 좋았던 점과 아쉬웠던 점은 무엇인가?

출산 · 육아 지원 제도와 아가와 엄마의 건강지원 서비스, 난임 · 심리 · 의료 상담 프로그램과 혜택에 대해 자세하게 알 수 있어서 좋았다.

CamScanner로 스캔하기

1. 오늘 실습경험의 의미 있는 점은 무엇인가?

오늘은 방문간호를 하는곳인 방문건강 관리실에서 실습을 하게되었는데 선생님을 따라 두 곳에 할머니 할아버지가 살고계신 곳으로 방문간호를 갔다왔다. 혈압과 BST를 재드리고 아프신곳은 없으신지 여쭤보는 것들을 하였다.

2. 오늘 실습을 하는 동안 나의 생각과 느낌은 어떠했는가?

아파트에 살고계신 분들과 노인정에 계신분들께 혈압을 재드리고 혈당을 체크하며 이야기들을 나눴는데 건강에 걱정이 많으셨고 조금 안좋다 얘기하면 너무 걱정하셔서 안쓰러운 느낌이 들었다. 방문간호사 선생님과 대화가 너무 잘 통해 많은 이야기들을 나누며 방문지를 이동하고 하면서 너무 즐거웠고 의미있는 시간이라 느껴져서 좋았습니다.

3. 오늘 실습을 통해 새롭게 알게 된 것은?

한명에 선생님과 한명의 학생이 따라가서 방문간호를 나간다는 것과 할아버지 할머니께 뭐드셨는지와 끼니를 잘 챙겨드시는지, 혈압, 혈당 수치들을 알려드리고 식단관리와 건강관리에 대해 설명해드리고 부족하신 부분이 어떤 것인지 알려드린다는 것을 알게 되었다.

4. 오늘 실습을 통해 새롭게 배운 것들이 향후 간호실무에 어떻게 적용될 수 있겠는가?

오늘 실습을 통해 방문간호는 어떤식으로 진행되는지, 가서 무엇을 하는지 알고 능숙하게 수행할수 있고 노인분들의 건강과 식습관 개선을 도와드리고 운동을 격려하는 수 있다.

5. 오늘 실습에서 좋았던 점과 아쉬웠던 점은 무엇인가?

오늘 방문건강관리실에서 실습을 하게되어 방문간호 선생님을 따라 여기저기 간호해드리러 나녔는데 너무 재밌고 보람찬 하루라 생각되어 좋았다.

CamScanner로 스캔하기

1. 오늘 실습경험의 의미 있는 점은 무엇인가?

군산보건소 실습을 하면서 이번에는 구강 보건 센터에서 실습을하게 되었는데 처음 치과에 실습을하게되어 생소한부분이 많았지만 선생님들이 하나씩 알려 주셔서 좋았다.

2. 오늘 실습을 하는 동안 나의 생각과 느낌은 어떠했는가?

치과에 대부분 10대 아이들도 70~80대 분들이 오셨고 대부분의 사람들은 불소도위해서 오셨기에는 가끔 노령의 어머니 들의 치아는 많이 상했고 관리를 잘 해야겠다고 생각하였습니다.

3. 오늘 실습을 통해 새롭게 알게 된 것은?

3대 치과 질병에는 부정 교합, 치아우식증, 그리고 치주질환인 것을 알게되었습니다. 또한 치아에는 신경이 있어 조심히 잘관리해야한다는것을 깨닫게 되었습니다.

4. 오늘 실습을 통해 새롭게 배운 것들이 향후 간호실무에 어떻게 적용될 수 있겠는가?

이번실습을 토대로 공부한것을 환자 또는 지인에게 교육할수 있을정도의 지식을 함양하며 노력해야겠다고 생각하게 되었습니다.

5. 오늘 실습에서 좋았던 점과 아쉬웠던 점은 무엇인가?

오늘 좋았던 점은 이번이 마지막 실습시작이며 보건소에 가고싶은입장으로 꼭 하고싶었는데 실습할수있어서 행복했다

1. 오늘 실습경험의 의미 있는 점은 무엇인가?

건강 보험공단 군산지사에서 건강보험공단의 일하는 일부분을 배우게 되었습니다. 처음에는 힘들었지만 건강보험공단의 일들의 일부분이지만 국민을위해 일하는 사람들의 모습을확인하고 도움을줄수있어서 좋았다

2. 오늘 실습을 하는 동안 나의 생각과 느낌은 어떠했는가?

전화로하면서 이상한 사람도 있고 착한 사람도 있는걸 보았습니다. 여기서 전화로 하시면서 다양한 사람들의 말을 받아주는게 하루이틀은 그게 하는거야 그럴수있지만 계속하는걸로 진짜 힘들겠다는 생각이 들었습니다

3. 오늘 실습을 통해 새롭게 알게 된 것은?

건강보험공단에서는 일반인들의 건강검진을 관리하는 것을 알게되었습니다. 특히 안할경우 독려까지 할정도로 국민을위해 노력하는 모습을 확인하며 나도 이런 모습에 반하지 않고 더 노력해야겠다고 생각했습니다.

4. 오늘 실습을 통해 새롭게 배운 것들이 향후 간호실무에 어떻게 적용될 수 있겠는가?

간호실무에서 이러한 사람을위해 노력하는 자세로의 모습을 관찰하여 나도 간호사가 된다면 환자분들을위해 노력하는 훌륭한 간호사가 되어야겠다고 생각했습니다

5. 오늘 실습에서 좋았던 점과 아쉬웠던 점은 무엇인가?

좋았던점은 다양한 실무를 배울수 있어서 좋았다

CamScanner로 스캔하기

1. 오늘 실습경험의 의미 있는 점은 무엇인가?

오늘 실습은 건강보험공단이라는 곳을 갔다오고 나서
치매안심센터라는 곳에 실습을하게되었는데 실습에서
의미있던점은 지역사회 간호사로 되게 다양한 일을하는구나라고
알게되었습니다.

2. 오늘 실습을 하는 동안 나의 생각과 느낌은 어떠했는가?

치매 예방을위해 여러가지 인지예방 프로그램을
한다는것을 느꼈었는데 아쉬웠던점은
약간 프로그램에 대처능력들이 부족한것에 대해
아쉬움을느꼈다

3. 오늘 실습을 통해 새롭게 알게 된 것은?

치매안심센터가 치매 진단을 해주면서
치매의 예방과 치매를 조기진단 받으면으로
치매의 유지를 도와주기위해 관리도하는것을 관찰
하였습니다.

4. 오늘 실습을 통해 새롭게 배운 것들이 향후 간호실무에 어떻게 적용될 수 있겠는가?

치매의 예방과 조기발견이 중요하다고 들었었고
어떻게해야하는지 알게되었습니다 사람들에게
정확히 교육할수 있도록 강화가 되어야겠다고 생각했습니다.

5. 오늘 실습에서 좋았던 점과 아쉬웠던 점은 무엇인가?

체계는 배운게 많이없어서 아쉬웠다고
생각했습니다.

CamScanner로 스캔하기

1. 오늘 실습경험의 의미 있는 점은 무엇인가?

방문간호를 실천하는 모습을 관찰 하였습니다

방문간호를 받으시는 노인분들과 간호사분들을 관찰하면서

방문간호에게 해 알게되었다

2. 오늘 실습을 하는 동안 나의 생각과 느낌은 어떠했는가?

방문간호는 취약계층을 더불어 시민 을대상으로 하면서

시민의 건강 을 직접 찾아가서 도와주는 간호임을 알게되었습니다.

3. 오늘 실습을 통해 새롭게 알게 된 것은?

방문간호를통해 환자분에게 적극적으로도울수있다는 것을알게되었습니다

방문간호를 생각보다 중요하다는것을 알게되었습니다.

4. 오늘 실습을 통해 새롭게 배운 것들이 향후 간호실무에 어떻게 적용될 수 있겠는가?

나중에 간호사가 된다면 방문간호의 중요성 을 잘 설명하고

환자분에게 더 잘 해주겠다고 생각 되었습니다.

5. 오늘 실습에서 좋았던 점과 아쉬웠던 점은 무엇인가?

방문간호를 할때 더 열심히 도와야겠다고 생각했습니다.

만약하게된다면 더 열심히 해서 도와드려야겠다고 생각했습니다

CamScanner로 스캔하기

1. 오늘 실습경험의 의미 있는 점은 무엇인가?

예방 접종 실을 가서 예방접종에 대해 배우게 되었습니다
예방접종에 종류와 예방접종의 시기 등 예방접종에 관한
내용들을 배우게 되어서 뜻깊은 시간이 되었던 것 같다.

2. 오늘 실습을 하는 동안 나의 생각과 느낌은 어떠했는가?

예방접종실에서 실습을 할 때 아기들이 많이 있었는데
아기들의 예방접종에 대해 교육을 받을 수 있었습니다.
아기들의 건강을 책임질 수 있는 예방접종은 꼭 해야겠다고 생각했습니다

3. 오늘 실습을 통해 새롭게 알게 된 것은?

폐렴구균의 연령은 65세 이상에서 무료로
예방접종을 할 수 있다는 것을 알게 되었습니다.

4. 오늘 실습을 통해 새롭게 배운 것들이 향후 간호실무에 어떻게 적용될 수 있겠는가?

여러가지 예방접종을 배워서 이 지식을 잘 활용하겠습니다
지식 부족인 사람들을 가르치면서 열심히
간호로 정진해야겠다고 생각했습니다.

5. 오늘 실습에서 좋았던 점과 아쉬웠던 점은 무엇인가?

마지막 실습실에서 너무 손이 안 쉽지만
즐거운 기분을 가지게 되었다.

CamScanner로 스캔하기

p27

1. 오늘 실습경험의 의미 있는 점은 무엇인가?

· 첫날이라서 교육담당 선생님께서 오리엔테이션을 해주셨다.
↳ 치매안심센터, 감염병관리계, 국민건강 보험공단 선생님들도 오셔서 하는 일과 어떤 곳인지 알려주셨다.

2. 오늘 실습을 하는 동안 나의 생각과 느낌은 어떠했는가?

· 첫 시작은 오리엔테이션 전에 출석을 불렀는데 우리 학교 이외에 타 학교에서 한 번에 모이는 장소를 전달 받지 못해 늦어졌고 방문간호팀이라 취약계층분들의 댁에 못 가는 상황이 발생해 정보 전달의 중요성을 다시 한 번 알게 되었다.

3. 오늘 실습을 통해 새롭게 알게 된 것은?

· 여름철이라 그런지 진드기 피해 사례가 급증하였으며 얼마 전 (전라도) 신안에서 진드기로 인해 돌아가신 일이 생겼었다. 환자 5명 中 2명이 사망하며 털진드기인 쯔쯔가무시는 잠복기가 10일 이내이며 검은 가피가 발견된다. 육안으로 확인이 가능하며 고열, 오한, 근육통, 구토가 일어나며 무리하게 잡아당기지 말고 핀셋을 이용해야 하며 병원으로 바로 갈수있게 안내해야한다.

4. 오늘 실습을 통해 새롭게 배운 것들이 향후 간호실무에 어떻게 적용될 수 있겠는가?

· 방문간호를 가기전에 AI - IOT 를 관찰하였는데 대상자에게 혈압, 혈당, 건강 상태를 수시로 측정하기 위해 손목시계, 체중계, 혈당측정기를 지급해 드리기 위해 인지검사, 고거력, 식생활, 신체활동 등을 물어 볼 수 있었다. 4차 혁명시대에서 AI로 건강상태를 확인할 수 있기에 이에 따른 사용방법을 정확하고 이해하기 쉽게 알려드려야 겠다고 생각했다.

5. 오늘 실습에서 좋았던 점과 아쉬웠던 점은 무엇인가?

· 보건소 이외에 다른 곳에 가면 똑같은 일, 관찰을 하게 되는데 여기서는 매일 다른 곳을 [illegible]가고 보게 되고 기대가 되고 최근에 시작한 사업 중 하나인 AI - IOT도 볼수 있어 뜻깊은 경험이었다.

CamScanner로 스캔하기

1. 오늘 실습경험의 의미 있는 점은 무엇인가?

- 매번 다른 부서를 가기에 오늘은 2일차로 예방접종실에 가게 되었다.
- 자궁경부암에 대한 예방접종도 보건소에서 하는 것을 관찰할 수 있었다.
- 대상자가 오시면 예진표 전부 작성 여부를 확인하고 열을 측정하는 역할을 하였다.

2. 오늘 실습을 하는 동안 나의 생각과 느낌은 어떠했는가?

- 이제는 월요일이라서 그런지 대상자분들이 꽤 오신듯 하였지만 오늘은 한가하다고 생각이 들었었다. 그래도 매일 이곳에 한명 이상이 온다는 것이 신기하였다. 내가 보건소에 갔을 때만 해도 혼자였기 때문에 다양한 이유로 온다는 것을 알 수 있었다.

3. 오늘 실습을 통해 새롭게 알게 된 것은?

- 다른 사람들은 어떻게 생각할지 모르겠지만 개인적으로는 예방접종실이 보건소에서 메인이라고 생각했던 과여서 그런지 해외에서 예방접종을 맞은 기록도 한국으로 이전을 시키는 역할을 하기도 한다는 사실을 새롭게 알게 되었다.

4. 오늘 실습을 통해 새롭게 배운 것들이 향후 간호실무에 어떻게 적용될 수 있겠는가?

- 법정 행정 관련 정보체계, 연령별 예방접종 맞는 종류 및 횟수와 주사 놓는 실력까지 갖춰야 하는 모습을 보고 마냥 쉽게 한다고 생각한 것을 반성하고 잘 알아야겠다고 생각했다.

5. 오늘 실습에서 좋았던 점과 아쉬웠던 점은 무엇인가?

- 아쉬웠던 부분보다는 당황스러웠던 점이 건너 들었던 보건소 직원분이 아직 의사는 아니셨지만 그래도 그 분께 공의이~ 이렇게 말하신게 계속 생각이 났다.

?

1. 오늘 실습경험의 의미 있는 점은 무엇인가?

- 국민건강관리 공단 에서 암검진 유선 독려 안내 전화 응대를 하였다.
- 예상 밖의 다양한 케이스가 있었고 모를 경우 돌려주기를 통해 담당 선생님께 안내를 해줄 수 있었다. (↳당겨받기)

2. 오늘 실습을 하는 동안 나의 생각과 느낌은 어떠했는가?

- 첫날에 오리엔테이션을 들었을 때 부터 공단에서는 전화응대를 해야 한다는 것에 두려웠고 도착하고나서 케이스를 설명받는데 소화해낼 자신이 없어서 걱정되었지만 다행히도 case 안의 내용이나 선생님이 알려주셔서 해낼수 있었고 대본이 없었다면 힘들었을 것 같았다.

3. 오늘 실습을 통해 새롭게 알게 된 것은?

- 외국인 분들은 성별 부분에 5(남), 6(여) 으로 표기하고 전화는 따로 돌리지 않으며 올해는 홀수년도생이 건강검진 해야지만 내년에 받고 싶으신 분들은 공단에 연락주도록 말하고 수검을 이미 하신분은 검진일자, 검진병원, 통화시간 (수화기 내린시간) 을 기입함을 알게 되었다.

4. 오늘 실습을 통해 새롭게 배운 것들이 향후 간호실무에 어떻게 적용될 수 있겠는가?

- 병동에서 선생님들이 전화응대를 하시는 모습을 보고 많이 힘들어 보인다고 생각하였고 물론 공단에서 하는 일과는 많이 다르겠지만 전화도 많이 받고 응대 하다보면 노티 하는데에도 노련해질 것이라고 생각하였다.

5. 오늘 실습에서 좋았던 점과 아쉬웠던 점은 무엇인가?

- 모르는 상황이 발생하였을 때 서로 돌려주기라는 버튼을 통해서 내선전화로 담당자 연결해드리겠습니다. 라고 하고 너무 초반에 일 하는데 걱정할 게 없는 것 같고 분위기가 너무 좋아서 왜 다들 공단에 취업하고 싶어 하는지 알게 된 계기가 되었다. (⊕ 설레임 (아이스크림), 핫도그(명랑) ⊕ 선물)

CamScanner로 스캔하기

1. 오늘 실습경험의 의미 있는 점은 무엇인가?

- 보건소에 과는 3개로 이루어졌다고 했는데 이 중 건강관리과에서 시민건강계라는 팀에서 학생 실습을 관리해주셔서 안내를 받았다.
- 구강검진센터에서 초등학생들의 홈메우기, 치아 우식증 확인을 볼 수 있었다.

2. 오늘 실습을 하는 동안 나의 생각과 느낌은 어떠했는가?

- 치과에 많이 아이들이 갈 줄 알았는데 생각보다 보건소로 찾아오는 아이들이 있어서 놀랐고 구강용품 봉투함 되어있는 것도 가져가서는 구나 싶었다.

3. 오늘 실습을 통해 새롭게 알게 된 것은?

- 치과 책은 옆에 두고 22개의 유치 ~~영구치~~로 빠지고 그리고 다시 나 영구치가 생기고 사랑니도 생김을 다시 기억할 수 있었다.

4. 오늘 실습을 통해 새롭게 배운 것들이 향후 간호실무에 어떻게 적용될 수 있겠는가?

- 치과 쪽에는 치위생사님이 있지만 보건소에서는 어떨지 모르기에 기본적인 사항은 알고 있어야 겠다.

5. 오늘 실습에서 좋았던 점과 아쉬웠던 점은 무엇인가?

- 좋았던 점은 오랜만에 아이들을 봐서 좋았지만 보건소보단 치과에 가서 치료받는 게 좋을 것 같다라는 생각이 들었다

CamScanner로 스캔하기

1. 오늘 실습경험의 의미 있는 점은 무엇인가?

• 치매 안심센터의 오리엔테이션을 들었다. (치매환자 전국 3위 / 양가 부모님(4명) 中 1명은 겪는다)
↳ 뇌의 병 ⊕ 일상생활의 어려움, 알츠하이머유형이 74%로 제일 비중이 크다.

• 치매 인지기능 검사를 하는 모습과 이에 맞는 상담을 하는 것을 볼 수 있었다.

• ~~[illegible]~~ 귀가 잘 안들리셔서 한자 한자 글씨를 써가며 검사를 하셨다.

2. 오늘 실습을 하는 동안 나의 생각과 느낌은 어떠했는가?

• 치매 환자가 생각보다 많이 겪으시고 증상으로는 인지기능에 있어 기억력 저하, 언어기능 저하, 수행능력 저하, 시간· 지남력 저하 / 시·공간 능력↓ 가 되는 것.

초기 치매 (1~3년)
↳ 약간 주변 도움이 필요

중기치매 (2~10년)
↳ 일상생활에 대한 상당한 정도의 도움 필요

말기치매 (8~12년)
↳ 대부분의 기억상실

중에 여기로는 중기치매분이 대부분 오시는듯 했다.

3. 오늘 실습을 통해 새롭게 알게 된 것은?

• 치매 Vs 건망증

- 치매: 문제에 대해 힌트를 줬을 때 답을 하지 못하는 것이다.
- 건망증: 문제에 대해 힌트를 줬을 때 답을 할 수 있는 것이다.

• 조기검진

① 인지선별검사 (CIST): 학년, 연세에 따른 기준점수에 의하여 <정상/인지저하>로 나눠짐

② 정밀검사 (SNSB): 인지저하시 실시 [경도인지장애 → 1년마다 재검, 치매의심 → 감별검사] → MRI or 혈액검사

4. 오늘 실습을 통해 새롭게 배운 것들이 향후 간호실무에 어떻게 적용될 수 있겠는가?

• 감별검사 시행 시 치매치료비 지원 서비스라고 돈을 지불하는데 어려움이 있을 시 중위소득 120% 이하 치매환자에게 치매 진료비라는 명목하에 제공하고 약제비를 제공한다.

• 조호물품 지원, 사례관리 서비스 or 실종예방 서비스를 이용할 수 있는 방안을 제공할 수 있다.
↳ 지문등록, 스마트워치 이용한 배회감지기, 배회인식표 배부

5. 오늘 실습에서 좋았던 점과 아쉬웠던 점은 무엇인가?

• 아쉬웠던 점으로는 치매관련 실인데 교구 자재들은 별로 안 보이고 딱딱하게 검사지들과 안내책자들만 있어서 도움이 될 수 있는 것은 보기가 힘들었다.

CamScanner로 스캔하기

P 28

1. 오늘 실습경험의 의미 있는 점은 무엇인가?

군산 보건소에서 OT를 들으며 실습 간에 경험해볼 곳의 관리자의 강의를 들을 수 있었다.

치매안심센터는 보건소에 포함되었었지만 시설면이 커지며 독립하였다.

2. 오늘 실습을 하는 동안 나의 생각과 느낌은 어떠했는가?

만 60세 이상 노인 중 96만명이 치매 환자이며

2023. 100만명 / 2038 — 200만명 추정으로 빠른 속도로

치매환자가 증가하고 있습니다.

~~주변에 있는 분들이~~ 치매환자가 많지 않아 놀라게 되었다.

3. 오늘 실습을 통해 새롭게 알게 된 것은?

치매 100명 중 5~10명 치료가능함을 알게 되었고

치매 또한 조기 발견의 중요성을 알게 되었다.

고혈압·당뇨 관리로 삶의 질을 높이며

가성 치매증, 갑상선 저하 등의 원인은 치료가 가능합니다.

4. 오늘 실습을 통해 새롭게 배운 것들이 향후 간호실무에 어떻게 적용될 수 있겠는가?

뇌손상은 치매위험이 2.4배, 음주는 2.2배, 흡연은 1.6배로

치매 예방을 위해 교육을 제공할 수 있을 것이며

치매의 조기발견과 치료가 완치되고 유의미함을 교육할 것 입니다.

5. 오늘 실습에서 좋았던 점과 아쉬웠던 점은 무엇인가?

CamScanner로 스캔하기

1. 오늘 실습경험의 의미 있는 점은 무엇인가?

국민건강보험 공단으로 실습을 하였다.

건강검진 대상자가 검진을 받지 않아 국민의 검진을 돕고

본인 부담액이 적음을 설명해주어 국민의 삶의 질 향상에 도움을 주는 업무를 하였다.

2. 오늘 실습을 하는 동안 나의 생각과 느낌은 어떠했는가?

제가 실습한 내용은 검진 관련이지만

다른 부서의 업무는 상해, 의료급여, 사후관리, 급여등을 조사하는

업무를 하는 모습을 볼 수 있었다.

사무실 인원들이 국민이 받을 권리를 받을 수 있도록 노력함에

새로움을 느끼고 보람을 느낄 수 있었다.

3. 오늘 실습을 통해 새롭게 알게 된 것은?

각종 암검사 중 간암검사는 간초음파 검사와 혈청 알파 태아 단백 검사로

간암을 검진하였다.

다른 암 검진 또한 그러하였으며

검진 정보를 모두 모을 수 있어 좋았지만 임신. 코로나. 암치료 등

개인의 사정이나 다의 정보는 공유되지 않아 업무의 효율성이 조금은 안타까웠다.

4. 오늘 실습을 통해 새롭게 배운 것들이 향후 간호실무에 어떻게 적용될 수 있겠는가?

공감적으로 ? 대화하여 상대를 존중하는 말하기를

몇 시간 동안 할 수 있어 간호 실무에서도 잘 활용하여

바람직한 간호를 할 수 있을 것이다.

5. 오늘 실습에서 좋았던 점과 아쉬웠던 점은 무엇인가?

일의 효율성이 조금은 떨어져 아쉬웠으나 ?

국민 검진을 통해 국민의 건강을 신경쓰고

삶의 질 향상에 도움을 줄 수 있어 좋았다.

1. 오늘 실습경험의 의미 있는 점은 무엇인가?

치매 안심 센터에서 실습을 하였다.
치매 검사를 위해 오시는 대상자들을 안내해드리고
치매를 예방하거나 뇌혈관 질환에 대해 설문지는 배포하는 행사를 했다.

2. 오늘 실습을 하는 동안 나의 생각과 느낌은 어떠했는가?

군산 수산시장에서 치매 안심센터와 뇌혈관 질환 관련
어깨에 두르고 대상인 사람에게 다가가 이야기하였다.
상인분들은 치매가 일을 하지않으면 생기는 질환으로
생각하시는 분들이 많았다.

3. 오늘 실습을 통해 새롭게 알게 된 것은?

보건소의 업무를 수행하며, 수행에 있어 불편한 점들을 알 수 있었다.
국가에서, 지원 받은 물품을 대상자에게 지급하기위해 몸을 써야하며.
물품의 수량 또한 맞춰야 해서 복잡하다는 생각이 들었다.

4. 오늘 실습을 통해 새롭게 배운 것들이 향후 간호실무에 어떻게 적용될 수 있겠는가?

실상을 경험하다가 공단 측은 공적으로 수행해야할 일들이
생기게 된다면 여러사람들이 혜택과 설명, 교육을 받을 수 있는
환경을 만들어두고 시작할 것이다. 물론, 내가 수행을 한들
문제점과 부족한 점이 있을 것이지만 누구하나 빠뜨리는 수행은 줄이고 싶다.

5. 오늘 실습에서 좋았던 점과 아쉬웠던 점은 무엇인가?

활동적인 수행을 할 수 있어 좋았으며
대상자들과 대화할 수 있는 분위기여서 좋았다.

CamScanner로 스캔하기

1. 오늘 실습경험의 의미 있는 점은 무엇인가?

방문간호를 수행하였다.
태블릿과 같은 전자기기를 대상자가 생활하는 곳에 가서 설치 및 교육을 하였다.
태블릿에는 혈압·혈당·몸무게·운동량, 등 다양한 컨텐츠를 수행할 수 있었다.

2. 오늘 실습을 하는 동안 나의 생각과 느낌은 어떠했는가?

태블릿에 있는 약 먹는 시간, 응급구조벨, 운동계획, 등.
여러가지 기능을 가진 위치까지 지원하는 등 보건소에서의 지원이
양질임을 알 수 있었다.

기술활용

물론, 지역별: 방문사의 교육 받은 내용의 차이가 있겠지만
공무원 분들의 성향과 일처리는 생각보다 더욱 여유가 흘러 보였다.

3. 오늘 실습을 통해 새롭게 알게 된 것은?

대상자들이 따로 원하지 않아도 지원을 받을 수 있었고.
지원의 배분이 필요함을 느낄 수 있었다.
그리고 [illegible] 어린, 젊은 사람들은 알 수 있는 혈압·혈당. 자가관리, 등을
잘 수행할 수 없는 노인 분들을 대상으로
교육이 필요함을 인지할 수 있었다.

교육. 맞춤형!

4. 오늘 실습을 통해 새롭게 배운 것들이 향후 간호실무에 어떻게 적용될 수 있겠는가?

향후, 실무에서 만나볼 대상자들, 보호자들에게 치료 뿐만이 아닌
교육의 목표 또한 이룰 수 있도록 노력할 것이며
전국, 전세계의 건강한 삶의 질 향상을 위해 힘을 쓰는
간호사가 될 것이다!

5. 오늘 실습에서 좋았던 점과 아쉬웠던 점은 무엇인가?

방문간호 상황에서 벌어지는 일에 대한
대처가 부족하여 아쉬운 생각이 들었다..

CamScanner로 스캔하기

1. 오늘 실습경험의 의미 있는 점은 무엇인가?

예방접종실 실습에서 생후 2주가된 태아가 방문하였다.
1주, 2주. 주차마다 성장할 아이를 예방접종을 위해 복지사업,
알림의 정책 ,등이 세세하게 설명이 잘 되어있는 것 같아 만족스러웠다.

2. 오늘 실습을 하는 동안 나의 생각과 느낌은 어떠했는가?

예방 접종을 시행하는 종류들에 대해 설명을 들을 수 있었고
예방 접종실에 근무하시는 분들이 모두 간호사였던 것 같다.
주사를 준비하고 주사를 놓음에 능숙함이 있어 멋져보였다.

3. 오늘 실습을 통해 새롭게 알게 된 것은?

태아의 BCG 주사는 피내 주사로 1cc 주사기로 주입하였다.
지금까지 관찰해온 피내 주사 중 가장 크게 낭포를 만들었는데.
이 주사는 길게는 5살 정도까지 흉이 남을 수 있으며
곪고 회복하고를 반복하는 결핵 예방 주사였다.

4. 오늘 실습을 통해 새롭게 배운 것들이 향후 간호실무에 어떻게 적용될 수 있겠는가?

소아과로 배정받아 임상을 수행할 경우 , 보건소에서 시행하는
예방접종 절차, 접종을 맞는 기간, 등을 자세히 알고 교육할 수 있을 것이다.
이를 통해 대상자들과 라포를 형성하고 그 이후 신뢰받는 간호사가
될 수 있을 것이다.

5. 오늘 실습에서 좋았던 점과 아쉬웠던 점은 무엇인가?

SN으로써 마지막 실습을 했다.
홀가분하면서 한편으로는 불편한 마음이 생긴다.
이제부터 내 행동과 결정에 강한 책임이 뒤따라 올 것이며
보다 전문적인 내용을 공부하고 멋진 간호사가 되도록 노력할 것이다.

CamScanner로 스캔하기

1. 오늘 실습경험의 의미 있는 점은 무엇인가?

오늘 처음으로 보건소 실습에 나왔다.

보건소에서는 각 부서에 다양한 경험을 해 볼 수 있도록 학생들의 실습 부서를 따로 배치해 주셨다.

2. 오늘 실습을 하는 동안 나의 생각과 느낌은 어떠했는가?

오늘은 결핵실 이라는 곳에 갔는데 학생이 할 수 있는 것이 아무것도 없었을 뿐더러 이전 간호관리학 실습지에 비해서 나에게 주어지는 일이 너무 없다 보니 좋기도 하면서 한편으로는 이래도 되나...라는 생각이 들었다.

3. 오늘 실습을 통해 새롭게 알게 된 것은?

오늘 실습을 통해서 잠복결핵 감염 치료에 대해서 알게 되었다.

치료는 결핵약 1종류 또는 2종류를 적게는 3개월, 많게는 9개월 동안 매일 1회 복용하는 방법으로 진행되며 치료 전에 치료자는 현재 앓고 있는 질환, 복용 중인 약제 등을 담당 의사에게 알리고, 진찰 및 혈액검사를 통해 건강상태를 확인한다.

치료 중 부작용을 최소화하고 안전하게 치료를 완료하기 위해 담당 의사의 진찰을 받고 혈액검사를 실시한다는 것을 알게되었다.

4. 오늘 실습을 통해 새롭게 배운 것들이 향후 간호실무에 어떻게 적용될 수 있겠는가?

향후 1. 결핵균 감염의 위험성이 큰 경우

2. 결핵 발병의 위험성이 큰 경우

3. 결핵 발병 시 중증 결핵 발병의 위험이 큰 경우

4. 결핵 발병 시 전파 가능성이 큰 경우(집단시설 종사자)등

5. 오늘 실습에서 좋았던 점과 아쉬웠던 점은 무엇인가?

오늘 실습하면서 내가 할 수 있는 일이 아무것도 없었다는 점에서 아쉬웠다.

CamScanner로 스캔하기

1. 오늘 실습경험의 의미 있는 점은 무엇인가?

오늘은 방문건강관리실에서 실습을 하게 되었다.

2. 오늘 실습을 하는 동안 나의 생각과 느낌은 어떠했는가?

대상자분께 직접 찾아가 불편함을 도와드리고 AI를 직접 설치하여 대상자분께 맞춤 설명을 해주셨다. 처음 접해 본 나도 어려움을 느꼈는데, 어르신 분들께서는 더욱더 어려울 것 같다는 생각이 들었으며 동시에 사회가 많이 변화했다는 것을 느꼈다.

지역사회의 기술적용

3. 오늘 실습을 통해 새롭게 알게 된 것은?

AI를 도모하여 앱을 깔고 위치에 연동을 시킨 후 대상자가 혈압을 재고, 체중을 재는 등 미션을 클리어 하면 보건소에서 대상자의 현 상태를 알 수 있어 보다 바르게 이상이 생길 시 처치할 수 있다는 점을 알게 되었다.

4. 오늘 실습을 통해 새롭게 배운 것들이 향후 간호실무에 어떻게 적용될 수 있겠는가?

혈압과, 체중을 꾸준히 재야 하는 분들께 이를 적용해 볼 수 있을 것이다.

5. 오늘 실습에서 좋았던 점과 아쉬웠던 점은 무엇인가?

직접 방문을 하여 간호를 수행할 수 있어 좋았다.

CamScanner로 스캔하기

1. 오늘 실습경험의 의미 있는 점은 무엇인가?

오늘은 치매안심센터에서 실습을 하게 되었다.

2. 오늘 실습을 하는 동안 나의 생각과 느낌은 어떠했는가?

치매조기 검진을 통해서 조기에 치매를 발견하여 적절한 치료로 완치 또는 중증

상태로의 진행을 억제하기 위해 검진을 한다는 점에서 인상 깊었다.

3. 오늘 실습을 통해 새롭게 알게 된 것은?

치매공공후견 사업 → 의사결정 능력이 저하된 치매노인이 자력으로 후견인을
선임하기 어려운 경우, 후견인의 도움을 받을 수 있도록
지원하여, 인간으로서의 존엄성을 보장하기 위한 사업

이라는 것을 알게 되었다.

4. 오늘 실습을 통해 새롭게 배운 것들이 향후 간호실무에 어떻게 적용될 수 있겠는가?

- 만 60세 이상의 치매환자
- 기초생활 수급자, 차상위자 등 저소득자 및 기초연금수급자
- 권리를 대변해 줄 가족이 없는 경우 등

5. 오늘 실습에서 좋았던 점과 아쉬웠던 점은 무엇인가?

오늘 실습을 통해서 치매안심센터는 무엇을 제공하는지,
어떤 일을 하는지에 대해 알 수 있어 좋았다.

1. 오늘 실습경험의 의미 있는 점은 무엇인가?

오늘은 건강증진 센터에서 실습을 하게 되었다.

2. 오늘 실습을 하는 동안 나의 생각과 느낌은 어떠했는가?

건강증진 센터에서 비만 또는 저체중을 관리하고 인바디를 측정하여
지금 현재 대상자에게 무엇이 필요한지를 상세하게 설명을 해주시는 등
실습관 또한 관리를 해준다는 점에서 놀라웠다.

3. 오늘 실습을 통해 새롭게 알게 된 것은?

오늘 실습을 하면서 성별 별로 적정 체중은 무엇이며 (정상체중)
근력, 칼로리 등등 정상범위 & 표준이 어느정도인지에 대해
알 수 있게 되었다.

4. 오늘 실습을 통해 새롭게 배운 것들이 향후 간호실무에 어떻게 적용될 수 있겠는가?

향후 보건교 또는 관리가 필요하는 대상자에게
이를 교육하는데에 적용해 볼 수 있을 것이다.

5. 오늘 실습에서 좋았던 점과 아쉬웠던 점은 무엇인가?

오늘 실습을 통해서 대상자 분들과 오래 있어 보지는 못했지만
(코로나가 있어서) 짧은 시간 동안 많은 것을 배우고 깨닫게 되는
시간을 가질 수 있어 좋았다.

CamScanner로 스캔하기

1. 오늘 실습경험의 의미 있는 점은 무엇인가?

오늘은 국민건강보험공단에서 실습을 하게 되었다.

2. 오늘 실습을 하는 동안 나의 생각과 느낌은 어떠했는가?

건강보험료 하위 50% 건강보험 가입자, 의료급여 수급자는 본인부담금(10%)이 없으며 검진 항목 중 2년 주기 항목은 전년도 미수검자도 공단 신청 시 수검이 가능하다는 점에서 놀라웠다.

3. 오늘 실습을 통해 새롭게 알게 된 것은?

연말에는 수검자의 40% 이상이 집중되어 예약불가, 장시간 대기 등의 불편함을 느낄 수 있다는 것과 폐암 고위험군 기준에 검진 후 금연을 하더라도 금연 15년 이내, 74세 까지 검진 대상이라는 것을 알게 되었다.

4. 오늘 실습을 통해 새롭게 배운 것들이 향후 간호실무에 어떻게 적용될 수 있겠는가?

향후 조기검진 대상이나 폐암 고위험군 대상에게 이를 적용해 볼 수 있을 것이다.

5. 오늘 실습에서 좋았던 점과 아쉬웠던 점은 무엇인가?

오늘 실습을 통해서 대상자에게 누릴 수 있는 혜택을 알려드리면서 내심 뿌듯하여 좋았다.

CamScanner로 스캔하기
